# Supplementary material for: Comparative analysis of shared and unique mechanisms important for diverse strains of Pasteurella multocida to cause systemic infection in mice
Source: PLoS Pathog. 2025 Dec 22;21(12):e1013398. doi: 10.1371/journal.ppat.1013398 (PMC12721544; doi:10.1371/journal.ppat.1013398)
Supplement: S1 Text — (DOCX) [file ppat.1013398.s001.docx]

## S1 Text for Comparative analysis of shared and unique mechanisms important for diverse strains of *Pasteurella multocida* to cause systemic infection in mice

Sequencing and assembly of the *P. multocida* strain M1404 genome

Prior to TraDIS analysis we performed whole-genome sequencing of *P. multocida* strain M1404 via Illumina and Nanopore sequencing as described in the materials and methods. The M1404 genome was assembled as previously described [1], with minor modifications. Briefly, Nanopore data were processed using High Accuracy Calling, and then further processed using Filtlong to remove reads < 1 kb in length and 5% of reads that had the lowest quality score. The processed M1404 nanopore reads were *de novo* assembled using Flye and then polished with Nanopore reads using Medaka. Illumina M1404 reads were trimmed using Trimmomatic, then used to polish the Nanopore assembly using Polypolish and then POLCA. The genome was then annotated using Prokka. The M1404 genome assembled into two contigs, a 2.35 Mbp contig (M1404_1) and a 66 kb contig (M1404_2), despite having 360x depth across the genome. Most publicly available complete *P. multocida* genomes have under 200x depth, indicating that 360x depth should be enough depth to generate a closed genome.

Given strain M1404 has previously been shown to contain several prophages [2], we searched the genome for prophage regions using PHASTER, identifying six different prophage regions (Table S8). Five of the prophage regions were identified on the 2.35 Mbp contig, and one was identified on the 66 kb contig. The prophage region on the 66 kb contig and three prophage regions on the 2.35 Mbp contig shared >99% nucleotide identity, and were homologs of bacteriophage Mu, suggesting that the same prophage has inserted three times into the genome. The region flanking the prophage on the 66 kb contig matched genomic DNA from several other *P. multocida* strains. Mauve alignments showed the region flanking the prophage on the 66 kb contig was present as an intact segment in several other complete *P. multocida* genomes (Figure S9). Alignment of the 2.35 Mbp contig with the same genomes showed a gap where the expected genomic DNA sequence should be in the M1404 chromosome at the assembly gap (Fig S9). Furthermore, mapping and individual read-level analysis of the nanopore and Illumina reads to the 2.35 Mbp contig and the 66 kb contig showed that no single reads went further than assembly junction for either contig. Together these data suggest that the 66 kb contig is likely an excised prophage that has captured genomic DNA and may exist as a separate replicon in M1404. The 66 kb region contained six genes identified as essential for growth in rich media, which suggests this region cannot be lost. As we could not generate a closed genome, the M1404 assembly in two contigs was used for TraDIS analysis, with the 2.35 Mbp contig made to start at *dnaA*.

**Modification of pAL953 to target genes of interest**

Mutants were generated in this study using a modified version of the ClosTron system [3]. pAL953, which contains a group II intron harbouring the kanamycin resistance gene *aph3* [4], was used to generate mutants in *P. multocida*. The ClosTron Intron Design Tool (https://clostron.com/intron-design-tool) was used to identify potential intron targeting sites in selected genes, and to obtain modified IBS, EBS1d and EBS2 oligonucleotide sequences, which are listed in S7 Table. The intron targeting region of the group II intron on pAL953 was then modified by splice overlap extension polymerase chain reaction (PCR), followed by cloning. For each gene, the first round PCR was performed using pAL953 as the template DNA with either BAP6544 and the gene specific IBS oligonucleotide or the gene specific EBS1d and EBS2 oligonucleotides. The first-round products were purified, and a second-round PCR setup with equal molar ratio of the two first-round PCR products and the gene specific IBS and EBS1d oligonucleotides. This generats an intron targeting region specific to a gene of interest. The second-round product was purified, digested with *Hin*dIII and *Bsr*GI and cloned into similarly digested pAL953. The modified intron targeting sequence was then confirmed by Sanger sequencing using BAP8243 and the modified pAL953 as the template DNA. Correctly retargeted plasmids were then used for mutagenesis.

## References

1. Smallman TR, Perlaza-Jiménez L, Wang X, Korman TM, Kotsanas D, Gibson JS, et al. Pathogenomic analysis and characterization of *Pasteurella multocida* strains recovered from human infections. Microbiol Spectr. 2024;12(4):e0380523. doi: 10.1128/spectrum.03805-23.

2. Moustafa AM, Seemann T, Gladman S, Adler B, Harper M, Boyce JD, et al. Comparative genomic analysis of asian haemorrhagic septicaemia-associated strains of *Pasteurella multocida* identifies more than 90 haemorrhagic septicaemia-specific genes. PLoS One. 2015;10(7):e0130296. doi: 10.1371/journal.pone.0130296.

3. Heap JT, Kuehne SA, Ehsaan M, Cartman ST, Cooksley CM, Scott JC, et al. The ClosTron: Mutagenesis in *Clostridium* refined and streamlined. J Microbiol Methods. 2010;80(1):49-55. doi: 10.1016/j.mimet.2009.10.018.

4. Harper M, St Michael F, John M, Vinogradov E, Steen JA, van Dorsten L, et al. *Pasteurella multocida* Heddleston serovar 3 and 4 strains share a common lipopolysaccharide biosynthesis locus but display both inter- and intrastrain lipopolysaccharide heterogeneity. J Bacteriol. 2013;195(21):4854-64. doi: 10.1128/jb.00779-13.
