# Supplementary material for: Comparative analysis of shared and unique mechanisms important for diverse strains of Pasteurella multocida to cause systemic infection in mice
Source: PLoS Pathog. 2025 Dec 22;21(12):e1013398. doi: 10.1371/journal.ppat.1013398 (PMC12721544; doi:10.1371/journal.ppat.1013398)
Supplement: S2 Text — (DOCX) [file ppat.1013398.s002.docx]

(10159:0.0010427327,((((((((((((10185:0.0000002499,11245:0.0000023663)100:0.0040985805,((32985:0.0000358746,33011:0.0000011761)100:0.0002279927,(FDAARGOS_216:0.0000057956,X73:0.0000002499)100:0.0001508622)100:0.0041823466)100:0.0008253571,(P1702:0.0033665925,P2723:0.0036229314)100:0.0014751944)99:0.0004570259,((10957:0.0034617560,(11020:0.0020255570,11205:0.0039824286)100:0.0006713814)100:0.0020967168,(39639:0.0008815384,(FDAARGOS_218:0.0000023658,P1059:0.0000002499)100:0.0007366332)100:0.0050020574)96:0.0007139167)89:0.0003356592,(((((((((((102426:0.0001083357,(((212516:0.0000394180,21275:0.0000313440)100:0.0000174138,((P030653_1:0.0000002499,P030653_2:0.0000002499)100:0.0000128011,(P5041881:0.0000023928,P504190:0.0000011761)100:0.0000139051)100:0.0000208938)100:0.0000208431,(HN06:0.0000908828,PM140:0.0000807035)100:0.0000424276)78:0.0000161366)100:0.0036141970,((((29135:0.0000104418,29792:0.0000069380)100:0.0000415847,LXSS001:0.0000912879)99:0.0000011352,((3480:0.0000717071,SD001:0.0000496350)100:0.0000012205,80176:0.0000266095)100:0.0001318114)91:0.0001213570,((40540:0.0000196723,((((618_90:0.0000000000,671_90:0.0000000000):0.0000000000,A0419:0.0000000000):0.0000002499,619_90:0.0000002499)97:0.0000011761,((A0757:0.0000000000,X1053:0.0000000000):0.0000002499,X0120:0.0000002499)98:0.0000011761)100:0.0000069380)86:0.0000003308,4407:0.0000173978)100:0.0002162165)100:0.0026533971)100:0.0024276447,(((((((((FCf15:0.0000093128,(PMWSG-4:0.0000011761,SAMN33747217:0.0000058272)100:0.0000265631)100:0.0000319688,(FCf76:0.0000150332,RCAD0259:0.0000115893)100:0.0000293127)99:0.0000069252,FCf45:0.0000593502)100:0.0000134932,(HuN001:0.0000277442,Q:0.0000404743)100:0.0000069227)100:0.0000069302,((FCf71:0.0000312202,FCf83:0.0000138693)100:0.0000185618,VP161:0.0000277164)100:0.0001991649)90:0.0000003344,Razi_0002:0.0000740771)89:0.0000160130,PMTB2_1:0.0000612245)100:0.0015014493,NCTC10382:0.0014822114)100:0.0037746903,NCTC10204:0.0044930949)100:0.0007818617)57:0.0002556109,((((((36502:0.0000116293,(HN07:0.0000173979,SD11:0.0000313104)100:0.0000127363)100:0.0001124306,(((9N:0.0004379324,Pm70:0.0000058067)100:0.0008513581,(Past3:0.0000035505,W16-1607_1:0.0000069044)100:0.0045380811)89:0.0000688482,(RCAD0726:0.0008941795,W22-2894-7:0.0012949597)90:0.0001034533)100:0.0000520405)100:0.0004723788,CIRMBP-0884:0.0002388389)100:0.0002558383,((CIRMBP-0873:0.0000069617,(((PF1:0.0000002499,PF6:0.0000002499)33:0.0000002499,((((PF10:0.0000047163,PF12:0.0000002499)87:0.0000002499,PF14:0.0000002499)66:0.0000002499,PF18:0.0000002499)12:0.0000002499,PF16:0.0000002499)15:0.0000002499)65:0.0000002499,PF9:0.0000011761)100:0.0000127349)100:0.0000578979,(((PF11:0.0000002499,PF19:0.0000002499)77:0.0000002499,PF17:0.0000002499)100:0.0000301044,((PF5:0.0000002499,PF7:0.0000002499)96:0.0000002499,PF8:0.0000002499)100:0.0000092883)100:0.0000637613)100:0.0000599593)100:0.0009115310,(LH06:0.0001613846,(PF13:0.0000002499,PF15:0.0000002499)100:0.0001435881)100:0.0008458341)100:0.0039369922,FCf147:0.0046479125)100:0.0011329895)45:0.0002246081,(38725:0.0045472405,Pm1621:0.0045059487)100:0.0008231869)45:0.0002608386,(((17BRD-035:0.0000002499,19BRD-057:0.0000035189)100:0.0013707043,CQ7:0.0016717621)100:0.0023356311,PM1463:0.0037465261)100:0.0017197031)75:0.0003941538,(PM-2:0.0051825780,Past33:0.0054966037)43:0.0006535873)43:0.0003452236,((((((((12601:0.0000289734,((IMT47951:0.0000058284,PS3536-1p:0.0000092863)95:0.0000023759,((((USDA-59962:0.0000011761,USDA-60224:0.0000002499)99:0.0000035221,(USDA-60213:0.0000002499,USDA-60717:0.0000002499)100:0.0000139316)61:0.0000002499,(USDA-60712:0.0000002499,USDA-60713:0.0000002499)100:0.0000116098)52:0.0000002499,(USDA-60380:0.0000069536,USDA-60675:0.0000209465)70:0.0000002499)69:0.0000002499)64:0.0000002499)100:0.0000300222,SAMN39110833:0.0000777073)100:0.0000345967,(((3358:0.0000035772,((3361:0.0000545830,36950:0.0000185499)100:0.0000002499,(USDA-60494:0.0000002499,USDA-60714:0.0000002499)100:0.0000092459)98:0.0000011761)94:0.0000045996,USDA-60248:0.0000220094)93:0.0000067759,(((ATCC_43137:0.0000002499,NCTC10322:0.0000002499)100:0.0000196988,((BS168:0.0000081058,(((EB104:0.0000104754,EB168:0.0000081021)86:0.0000002499,PM_8-6:0.0000011761)48:0.0000002499,(PM8-1:0.0000002499,TB168:0.0000002499)41:0.0000002499)54:0.0000002499)100:0.0000347142,HB03:0.0000300629)100:0.0000289122)100:0.0000835600,SAMD00436440:0.0002124571)99:0.0000922523)93:0.0000708626)94:0.0000248728,((((HB01:0.0000070702,SAMN04520874:0.0000127180)100:0.0000023767,Pm64:0.0000149655)100:0.0000024486,SAMN20842242:0.0000127736)100:0.0000092883,(USDA-59910:0.0000035231,(USDA-60215:0.0000002499,USDA-60381:0.0000047029)100:0.0000046996)100:0.0000058318)100:0.0002679636)75:0.0000909891,14424:0.0000612145)75:0.0000583150,PM22:0.0001007451)98:0.0002256789,(P1933:0.0000468243,USDA-60385:0.0000574192)100:0.0002106756)100:0.0047599534,(HN01:0.0009306083,HN02:0.0012182040)100:0.0076760763)66:0.0007954651)48:0.0003317787,(((31971:0.0000701233,Past29:0.0001915278)100:0.0040957110,OH1905:0.0009746032)100:0.0059555575,RCAD0730:0.0056499431)77:0.0012011209)39:0.0002910147,(((((Ban-PM4:0.0000002499,Ban-PM7:0.0000002499)100:0.0000556816,(CUL-TANUVAS_2020:0.0000244099,NCTC10323:0.0000174224)100:0.0000092964)100:0.0000011761,Razi_Pm0001:0.0000197813)100:0.0000035270,((SAMN17080908:0.0000047105,sample-B:0.0000058462)100:0.0000035317,Tibet-Pm1:0.0000162489)100:0.0000371605)100:0.0000901228,M1404:0.0001282082)100:0.0061744632)35:0.0002824633,(HS_Canada1:0.0042153034,W22-979_1:0.0052346493)99:0.0011174946)55:0.0002029825)57:0.0004487119,P2192:0.0077943329)69:0.0003945853,(41060:0.0016229364,P2095:0.0005616591)100:0.0058024586)90:0.0008537408,W22-3735_1:0.0066817199)100:0.0023471083,28606:0.0061036239)100:0.0121318269,((AKS2021-HT3:0.0012439332,AKS2022-HT5:0.0005772707)100:0.0005851647,GS2020-X2:0.0006768590)100:0.0095753859)100:0.0101401062,(21317:0.0000002573,35564:0.0000022823)100:0.0083889677)100:0.0072548284,(((((161215033201-1:0.0046564179,KVNON-213:0.0033372118)100:0.0019872946,20N:0.0037866755)84:0.0006860126,((34030:0.0045742414,NCTC11619:0.0039470873)86:0.0007306655,(P1581:0.0038163628,Past6:0.0036905108)99:0.0011037488)86:0.0005115830)92:0.0004582028,((FDAARGOS_384:0.0000002499,FDAARGOS_385:0.0000002499)100:0.0034705824,Past9:0.0047247381)98:0.0008852225)100:0.0012499162,FDAARGOS_644:0.0051399030)88:0.0013345014)100:0.0038047492,Pm1618:0.0009732352);
