# Supplementary material for: Comparative analysis of shared and unique mechanisms important for diverse strains of Pasteurella multocida to cause systemic infection in mice
Source: PLoS Pathog. 2025 Dec 22;21(12):e1013398. doi: 10.1371/journal.ppat.1013398 (PMC12721544; doi:10.1371/journal.ppat.1013398)
Supplement: S3 Text — (DOCX) [file ppat.1013398.s003.docx]

##gff-version 3

##sequence-region gnl|Prokka|M1404_1 1 2047962

##sequence-region gnl|Prokka|M1404_2 1 100922

##sequence-region gnl|Prokka|M1404_3 1 196688

##sequence-region gnl|Prokka|M1404_4 1 33969

##sequence-region gnl|Prokka|M1404_5 1 35329

##sequence-region gnl|Prokka|M1404_6 1 64605

gnl|Prokka|M1404_1 prokka gene 1 1356 . + . ID=M1404_00001_gene;Name=dnaA;gene=dnaA;locus_tag=M1404_00001

gnl|Prokka|M1404_1 Prodigal:002006 CDS 1 1356 . + 0 ID=M1404_00001;Parent=M1404_00001_gene;Name=dnaA;db_xref=COG:COG0593;gene=dnaA;inference=ab initio prediction:Prodigal:002006,similar to AA sequence:UniProtKB:P03004;locus_tag=M1404_00001;product=Chromosomal replication initiator protein DnaA;protein_id=gnl|Prokka|M1404_00001

gnl|Prokka|M1404_1 prokka gene 1370 2470 . + . ID=M1404_00002_gene;Name=dnaN;gene=dnaN;locus_tag=M1404_00002

gnl|Prokka|M1404_1 Prodigal:002006 CDS 1370 2470 . + 0 ID=M1404_00002;Parent=M1404_00002_gene;Name=dnaN;db_xref=COG:COG0592;gene=dnaN;inference=ab initio prediction:Prodigal:002006,similar to AA sequence:UniProtKB:P0A988;locus_tag=M1404_00002;product=Beta sliding clamp;protein_id=gnl|Prokka|M1404_00002

gnl|Prokka|M1404_1 prokka gene 2488 3564 . + . ID=M1404_00003_gene;Name=recF;gene=recF;locus_tag=M1404_00003

gnl|Prokka|M1404_1 Prodigal:002006 CDS 2488 3564 . + 0 ID=M1404_00003;Parent=M1404_00003_gene;Name=recF;db_xref=COG:COG1195;gene=recF;inference=ab initio prediction:Prodigal:002006,similar to AA sequence:UniProtKB:P0A7H0;locus_tag=M1404_00003;product=DNA replication and repair protein RecF;protein_id=gnl|Prokka|M1404_00003

gnl|Prokka|M1404_1 prokka gene 3570 4325 . - . ID=M1404_00004_gene;Name=COQ5_1;gene=COQ5_1;locus_tag=M1404_00004

gnl|Prokka|M1404_1 Prodigal:002006 CDS 3570 4325 . - 0 ID=M1404_00004;Parent=M1404_00004_gene;eC_number=2.1.1.163;Name=COQ5_1;gene=COQ5_1;inference=ab initio prediction:Prodigal:002006,protein motif:HAMAP:MF_01813;locus_tag=M1404_00004;product=2-methoxy-6-polyprenyl-1%2C4-benzoquinol methylase%2C mitochondrial;protein_id=gnl|Prokka|M1404_00004

gnl|Prokka|M1404_1 prokka gene 4457 5098 . - . ID=M1404_00005_gene;Name=crp;gene=crp;locus_tag=M1404_00005

gnl|Prokka|M1404_1 Prodigal:002006 CDS 4457 5098 . - 0 ID=M1404_00005;Parent=M1404_00005_gene;Name=crp;db_xref=COG:COG0664;gene=crp;inference=ab initio prediction:Prodigal:002006,similar to AA sequence:UniProtKB:P0ACJ8;locus_tag=M1404_00005;product=cAMP-activated global transcriptional regulator CRP;protein_id=gnl|Prokka|M1404_00005

gnl|Prokka|M1404_1 prokka gene 5138 5356 . - . ID=M1404_00006_gene;locus_tag=M1404_00006

gnl|Prokka|M1404_1 Prodigal:002006 CDS 5138 5356 . - 0 ID=M1404_00006;Parent=M1404_00006_gene;inference=ab initio prediction:Prodigal:002006;locus_tag=M1404_00006;product=hypothetical protein;protein_id=gnl|Prokka|M1404_00006

gnl|Prokka|M1404_1 prokka gene 5378 5977 . - . ID=M1404_00007_gene;Name=slmA;gene=slmA;locus_tag=M1404_00007

gnl|Prokka|M1404_1 Prodigal:002006 CDS 5378 5977 . - 0 ID=M1404_00007;Parent=M1404_00007_gene;Name=slmA;db_xref=COG:COG1309;gene=slmA;inference=ab initio prediction:Prodigal:002006,similar to AA sequence:UniProtKB:Q9KVD2;locus_tag=M1404_00007;product=Nucleoid occlusion factor SlmA;protein_id=gnl|Prokka|M1404_00007

gnl|Prokka|M1404_1 prokka gene 5981 6436 . - . ID=M1404_00008_gene;Name=dut;gene=dut;locus_tag=M1404_00008

gnl|Prokka|M1404_1 Prodigal:002006 CDS 5981 6436 . - 0 ID=M1404_00008;Parent=M1404_00008_gene;eC_number=3.6.1.23;Name=dut;db_xref=COG:COG0756;gene=dut;inference=ab initio prediction:Prodigal:002006,similar to AA sequence:UniProtKB:P06968;locus_tag=M1404_00008;product=Deoxyuridine 5'-triphosphate nucleotidohydrolase;protein_id=gnl|Prokka|M1404_00008

gnl|Prokka|M1404_1 prokka gene 6452 7654 . - . ID=M1404_00009_gene;Name=coaBC;gene=coaBC;locus_tag=M1404_00009

gnl|Prokka|M1404_1 Prodigal:002006 CDS 6452 7654 . - 0 ID=M1404_00009;Parent=M1404_00009_gene;Name=coaBC;db_xref=COG:COG0452;gene=coaBC;inference=ab initio prediction:Prodigal:002006,similar to AA sequence:UniProtKB:P0ABQ0;locus_tag=M1404_00009;product=Coenzyme A biosynthesis bifunctional protein CoaBC;protein_id=gnl|Prokka|M1404_00009

gnl|Prokka|M1404_1 prokka gene 7833 8507 . + . ID=M1404_00010_gene;locus_tag=M1404_00010

gnl|Prokka|M1404_1 Prodigal:002006 CDS 7833 8507 . + 0 ID=M1404_00010;Parent=M1404_00010_gene;inference=ab initio prediction:Prodigal:002006,similar to AA sequence:UniProtKB:Q97QW0;locus_tag=M1404_00010;note=UPF0758 protein SP_1088;product=hypothetical protein;protein_id=gnl|Prokka|M1404_00010

gnl|Prokka|M1404_1 prokka gene 8750 8986 . + . ID=M1404_00011_gene;Name=rpmB;gene=rpmB;locus_tag=M1404_00011

gnl|Prokka|M1404_1 Prodigal:002006 CDS 8750 8986 . + 0 ID=M1404_00011;Parent=M1404_00011_gene;Name=rpmB;db_xref=COG:COG0227;gene=rpmB;inference=ab initio prediction:Prodigal:002006,similar to AA sequence:UniProtKB:P0A7M2;locus_tag=M1404_00011;product=50S ribosomal protein L28;protein_id=gnl|Prokka|M1404_00011

gnl|Prokka|M1404_1 prokka gene 8998 9168 . + . ID=M1404_00012_gene;Name=rpmG;gene=rpmG;locus_tag=M1404_00012

gnl|Prokka|M1404_1 Prodigal:002006 CDS 8998 9168 . + 0 ID=M1404_00012;Parent=M1404_00012_gene;Name=rpmG;db_xref=COG:COG0267;gene=rpmG;inference=ab initio prediction:Prodigal:002006,similar to AA sequence:UniProtKB:P0A7N9;locus_tag=M1404_00012;product=50S ribosomal protein L33;protein_id=gnl|Prokka|M1404_00012

gnl|Prokka|M1404_1 prokka gene 9236 10048 . + . ID=M1404_00013_gene;Name=mutM;gene=mutM;locus_tag=M1404_00013

gnl|Prokka|M1404_1 Prodigal:002006 CDS 9236 10048 . + 0 ID=M1404_00013;Parent=M1404_00013_gene;eC_number=3.2.2.23;Name=mutM;db_xref=COG:COG0266;gene=mutM;inference=ab initio prediction:Prodigal:002006,similar to AA sequence:UniProtKB:P05523;locus_tag=M1404_00013;product=Formamidopyrimidine-DNA glycosylase;protein_id=gnl|Prokka|M1404_00013

gnl|Prokka|M1404_1 prokka gene 10051 11076 . - . ID=M1404_00014_gene;locus_tag=M1404_00014

gnl|Prokka|M1404_1 Prodigal:002006 CDS 10051 11076 . - 0 ID=M1404_00014;Parent=M1404_00014_gene;inference=ab initio prediction:Prodigal:002006;locus_tag=M1404_00014;product=hypothetical protein;protein_id=gnl|Prokka|M1404_00014

gnl|Prokka|M1404_1 prokka gene 11079 12137 . - . ID=M1404_00015_gene;locus_tag=M1404_00015

gnl|Prokka|M1404_1 Prodigal:002006 CDS 11079 12137 . - 0 ID=M1404_00015;Parent=M1404_00015_gene;inference=ab initio prediction:Prodigal:002006;locus_tag=M1404_00015;product=hypothetical protein;protein_id=gnl|Prokka|M1404_00015

gnl|Prokka|M1404_1 prokka gene 12165 13007 . - . ID=M1404_00016_gene;locus_tag=M1404_00016

gnl|Prokka|M1404_1 Prodigal:002006 CDS 12165 13007 . - 0 ID=M1404_00016;Parent=M1404_00016_gene;inference=ab initio prediction:Prodigal:002006;locus_tag=M1404_00016;product=hypothetical protein;protein_id=gnl|Prokka|M1404_00016

gnl|Prokka|M1404_1 prokka gene 13064 13276 . - . ID=M1404_00017_gene;Name=rpmE;gene=rpmE;locus_tag=M1404_00017

gnl|Prokka|M1404_1 Prodigal:002006 CDS 13064 13276 . - 0 ID=M1404_00017;Parent=M1404_00017_gene;Name=rpmE;db_xref=COG:COG0254;gene=rpmE;inference=ab initio prediction:Prodigal:002006,similar to AA sequence:UniProtKB:P0A7M9;locus_tag=M1404_00017;product=50S ribosomal protein L31;protein_id=gnl|Prokka|M1404_00017

gnl|Prokka|M1404_1 prokka gene 13495 14337 . + . ID=M1404_00018_gene;Name=lex1;gene=lex1;locus_tag=M1404_00018

gnl|Prokka|M1404_1 Prodigal:002006 CDS 13495 14337 . + 0 ID=M1404_00018;Parent=M1404_00018_gene;eC_number=2.-.-.-;Name=lex1;db_xref=COG:COG3306;gene=lex1;inference=ab initio prediction:Prodigal:002006,similar to AA sequence:UniProtKB:Q03974;locus_tag=M1404_00018;product=Lipooligosaccharide biosynthesis protein lex-1;protein_id=gnl|Prokka|M1404_00018

gnl|Prokka|M1404_1 prokka gene 14881 17094 . + . ID=M1404_00019_gene;Name=priA;gene=priA;locus_tag=M1404_00019

gnl|Prokka|M1404_1 Prodigal:002006 CDS 14881 17094 . + 0 ID=M1404_00019;Parent=M1404_00019_gene;eC_number=3.6.4.-;Name=priA;db_xref=COG:COG1198;gene=priA;inference=ab initio prediction:Prodigal:002006,similar to AA sequence:UniProtKB:P17888;locus_tag=M1404_00019;product=Primosomal protein N';protein_id=gnl|Prokka|M1404_00019

gnl|Prokka|M1404_1 prokka gene 17232 17966 . + . ID=M1404_00020_gene;Name=ftsN;gene=ftsN;locus_tag=M1404_00020

gnl|Prokka|M1404_1 Prodigal:002006 CDS 17232 17966 . + 0 ID=M1404_00020;Parent=M1404_00020_gene;Name=ftsN;db_xref=COG:COG3087;gene=ftsN;inference=ab initio prediction:Prodigal:002006,similar to AA sequence:UniProtKB:P29131;locus_tag=M1404_00020;product=Cell division protein FtsN;protein_id=gnl|Prokka|M1404_00020

gnl|Prokka|M1404_1 prokka gene 18047 18682 . + . ID=M1404_00021_gene;locus_tag=M1404_00021

gnl|Prokka|M1404_1 Prodigal:002006 CDS 18047 18682 . + 0 ID=M1404_00021;Parent=M1404_00021_gene;db_xref=COG:COG1309;inference=ab initio prediction:Prodigal:002006,similar to AA sequence:UniProtKB:P44923;locus_tag=M1404_00021;product=putative HTH-type transcriptional regulator;protein_id=gnl|Prokka|M1404_00021

gnl|Prokka|M1404_1 prokka gene 18712 19923 . + . ID=M1404_00022_gene;Name=mdtA;gene=mdtA;locus_tag=M1404_00022

gnl|Prokka|M1404_1 Prodigal:002006 CDS 18712 19923 . + 0 ID=M1404_00022;Parent=M1404_00022_gene;Name=mdtA;gene=mdtA;inference=ab initio prediction:Prodigal:002006,protein motif:HAMAP:MF_01422;locus_tag=M1404_00022;product=Multidrug resistance protein MdtA;protein_id=gnl|Prokka|M1404_00022

gnl|Prokka|M1404_1 prokka gene 19951 20394 . + . ID=M1404_00023_gene;Name=msrC;gene=msrC;locus_tag=M1404_00023

gnl|Prokka|M1404_1 Prodigal:002006 CDS 19951 20394 . + 0 ID=M1404_00023;Parent=M1404_00023_gene;eC_number=1.8.4.14;Name=msrC;db_xref=COG:COG1956;gene=msrC;inference=ab initio prediction:Prodigal:002006,similar to AA sequence:UniProtKB:P76270;locus_tag=M1404_00023;product=Free methionine-R-sulfoxide reductase;protein_id=gnl|Prokka|M1404_00023

gnl|Prokka|M1404_1 prokka gene 20404 23502 . + . ID=M1404_00024_gene;Name=bepE;gene=bepE;locus_tag=M1404_00024

gnl|Prokka|M1404_1 Prodigal:002006 CDS 20404 23502 . + 0 ID=M1404_00024;Parent=M1404_00024_gene;Name=bepE;gene=bepE;inference=ab initio prediction:Prodigal:002006,similar to AA sequence:UniProtKB:Q8G2M6;locus_tag=M1404_00024;product=Efflux pump membrane transporter BepE;protein_id=gnl|Prokka|M1404_00024

gnl|Prokka|M1404_1 prokka gene 23560 24624 . - . ID=M1404_00025_gene;locus_tag=M1404_00025

gnl|Prokka|M1404_1 Prodigal:002006 CDS 23560 24624 . - 0 ID=M1404_00025;Parent=M1404_00025_gene;inference=ab initio prediction:Prodigal:002006;locus_tag=M1404_00025;product=hypothetical protein;protein_id=gnl|Prokka|M1404_00025

gnl|Prokka|M1404_1 prokka gene 24704 25054 . + . ID=M1404_00026_gene;Name=yfgD;gene=yfgD;locus_tag=M1404_00026

gnl|Prokka|M1404_1 Prodigal:002006 CDS 24704 25054 . + 0 ID=M1404_00026;Parent=M1404_00026_gene;Name=yfgD;db_xref=COG:COG1393;gene=yfgD;inference=ab initio prediction:Prodigal:002006,similar to AA sequence:UniProtKB:P76569;locus_tag=M1404_00026;product=putative protein YfgD;protein_id=gnl|Prokka|M1404_00026

gnl|Prokka|M1404_1 prokka gene 25127 25408 . + . ID=M1404_00027_gene;locus_tag=M1404_00027

gnl|Prokka|M1404_1 Prodigal:002006 CDS 25127 25408 . + 0 ID=M1404_00027;Parent=M1404_00027_gene;inference=ab initio prediction:Prodigal:002006;locus_tag=M1404_00027;product=hypothetical protein;protein_id=gnl|Prokka|M1404_00027

gnl|Prokka|M1404_1 prokka gene 25413 25706 . + . ID=M1404_00028_gene;locus_tag=M1404_00028

gnl|Prokka|M1404_1 Prodigal:002006 CDS 25413 25706 . + 0 ID=M1404_00028;Parent=M1404_00028_gene;inference=ab initio prediction:Prodigal:002006;locus_tag=M1404_00028;product=hypothetical protein;protein_id=gnl|Prokka|M1404_00028

gnl|Prokka|M1404_1 prokka gene 25931 29101 . + . ID=M1404_00029_gene;Name=cas9;gene=cas9;locus_tag=M1404_00029

gnl|Prokka|M1404_1 Prodigal:002006 CDS 25931 29101 . + 0 ID=M1404_00029;Parent=M1404_00029_gene;eC_number=3.1.-.-;Name=cas9;db_xref=COG:COG3513;gene=cas9;inference=ab initio prediction:Prodigal:002006,similar to AA sequence:UniProtKB:Q9CLT2;locus_tag=M1404_00029;product=CRISPR-associated endonuclease Cas9;protein_id=gnl|Prokka|M1404_00029

gnl|Prokka|M1404_1 prokka gene 29157 30071 . + . ID=M1404_00030_gene;Name=cas1_1;gene=cas1_1;locus_tag=M1404_00030

gnl|Prokka|M1404_1 Prodigal:002006 CDS 29157 30071 . + 0 ID=M1404_00030;Parent=M1404_00030_gene;eC_number=3.1.-.-;Name=cas1_1;gene=cas1_1;inference=ab initio prediction:Prodigal:002006,protein motif:HAMAP:MF_01470;locus_tag=M1404_00030;product=CRISPR-associated endonuclease Cas1;protein_id=gnl|Prokka|M1404_00030

gnl|Prokka|M1404_1 prokka gene 30064 30390 . + . ID=M1404_00031_gene;Name=cas2;gene=cas2;locus_tag=M1404_00031

gnl|Prokka|M1404_1 Prodigal:002006 CDS 30064 30390 . + 0 ID=M1404_00031;Parent=M1404_00031_gene;eC_number=3.1.-.-;Name=cas2;gene=cas2;inference=ab initio prediction:Prodigal:002006,similar to AA sequence:UniProtKB:G3ECR3;locus_tag=M1404_00031;product=CRISPR-associated endoribonuclease Cas2;protein_id=gnl|Prokka|M1404_00031

gnl|Prokka|M1404_1 minced:0.4.2 repeat_region 30624 30859 . . . note=CRISPR with 4 repeat units;rpt_family=CRISPR;rpt_type=direct;rpt_unit_seq=ATTGTAGCACTGCGAAATGAGAGAGGGAACTACAACTC

gnl|Prokka|M1404_1 prokka gene 30904 32277 . - . ID=M1404_00032_gene;Name=argH;gene=argH;locus_tag=M1404_00032

gnl|Prokka|M1404_1 Prodigal:002006 CDS 30904 32277 . - 0 ID=M1404_00032;Parent=M1404_00032_gene;eC_number=4.3.2.1;Name=argH;db_xref=COG:COG0165;gene=argH;inference=ab initio prediction:Prodigal:002006,similar to AA sequence:UniProtKB:P11447;locus_tag=M1404_00032;product=Argininosuccinate lyase;protein_id=gnl|Prokka|M1404_00032

gnl|Prokka|M1404_1 prokka gene 32293 33066 . - . ID=M1404_00033_gene;Name=argB;gene=argB;locus_tag=M1404_00033

gnl|Prokka|M1404_1 Prodigal:002006 CDS 32293 33066 . - 0 ID=M1404_00033;Parent=M1404_00033_gene;eC_number=2.7.2.8;Name=argB;db_xref=COG:COG0548;gene=argB;inference=ab initio prediction:Prodigal:002006,similar to AA sequence:UniProtKB:Q8ZA87;locus_tag=M1404_00033;product=Acetylglutamate kinase;protein_id=gnl|Prokka|M1404_00033

gnl|Prokka|M1404_1 prokka gene 33067 34074 . - . ID=M1404_00034_gene;Name=argC;gene=argC;locus_tag=M1404_00034

gnl|Prokka|M1404_1 Prodigal:002006 CDS 33067 34074 . - 0 ID=M1404_00034;Parent=M1404_00034_gene;eC_number=1.2.1.38;Name=argC;db_xref=COG:COG0002;gene=argC;inference=ab initio prediction:Prodigal:002006,similar to AA sequence:UniProtKB:Q8ZKL8;locus_tag=M1404_00034;product=N-acetyl-gamma-glutamyl-phosphate reductase;protein_id=gnl|Prokka|M1404_00034

gnl|Prokka|M1404_1 prokka gene 34110 35330 . + . ID=M1404_00035_gene;Name=argE;gene=argE;locus_tag=M1404_00035

gnl|Prokka|M1404_1 Prodigal:002006 CDS 34110 35330 . + 0 ID=M1404_00035;Parent=M1404_00035_gene;eC_number=3.5.1.16;Name=argE;db_xref=COG:COG0624;gene=argE;inference=ab initio prediction:Prodigal:002006,similar to AA sequence:UniProtKB:P23908;locus_tag=M1404_00035;product=Acetylornithine deacetylase;protein_id=gnl|Prokka|M1404_00035

gnl|Prokka|M1404_1 prokka gene 35416 36204 . - . ID=M1404_00036_gene;locus_tag=M1404_00036

gnl|Prokka|M1404_1 Prodigal:002006 CDS 35416 36204 . - 0 ID=M1404_00036;Parent=M1404_00036_gene;inference=ab initio prediction:Prodigal:002006;locus_tag=M1404_00036;product=hypothetical protein;protein_id=gnl|Prokka|M1404_00036

gnl|Prokka|M1404_1 prokka gene 36315 36390 . - . ID=M1404_00037_gene;locus_tag=M1404_00037

gnl|Prokka|M1404_1 Aragorn:001002 tRNA 36315 36390 . - . ID=M1404_00037;Parent=M1404_00037_gene;inference=COORDINATES:profile:Aragorn:001002;locus_tag=M1404_00037;product=tRNA-Val(tac)

gnl|Prokka|M1404_1 prokka gene 36455 36530 . - . ID=M1404_00038_gene;locus_tag=M1404_00038

gnl|Prokka|M1404_1 Aragorn:001002 tRNA 36455 36530 . - . ID=M1404_00038;Parent=M1404_00038_gene;inference=COORDINATES:profile:Aragorn:001002;locus_tag=M1404_00038;product=tRNA-Val(tac)

gnl|Prokka|M1404_1 prokka gene 36582 36657 . - . ID=M1404_00039_gene;locus_tag=M1404_00039

gnl|Prokka|M1404_1 Aragorn:001002 tRNA 36582 36657 . - . ID=M1404_00039;Parent=M1404_00039_gene;inference=COORDINATES:profile:Aragorn:001002;locus_tag=M1404_00039;product=tRNA-Val(tac)

gnl|Prokka|M1404_1 prokka gene 36699 36774 . - . ID=M1404_00040_gene;locus_tag=M1404_00040

gnl|Prokka|M1404_1 Aragorn:001002 tRNA 36699 36774 . - . ID=M1404_00040;Parent=M1404_00040_gene;inference=COORDINATES:profile:Aragorn:001002;locus_tag=M1404_00040;product=tRNA-Val(tac)

gnl|Prokka|M1404_1 prokka gene 36958 38400 . + . ID=M1404_00041_gene;Name=gltX;gene=gltX;locus_tag=M1404_00041

gnl|Prokka|M1404_1 Prodigal:002006 CDS 36958 38400 . + 0 ID=M1404_00041;Parent=M1404_00041_gene;eC_number=6.1.1.17;Name=gltX;db_xref=COG:COG0008;gene=gltX;inference=ab initio prediction:Prodigal:002006,similar to AA sequence:UniProtKB:P04805;locus_tag=M1404_00041;product=Glutamate--tRNA ligase;protein_id=gnl|Prokka|M1404_00041

gnl|Prokka|M1404_1 prokka gene 38489 38564 . + . ID=M1404_00042_gene;locus_tag=M1404_00042

gnl|Prokka|M1404_1 Aragorn:001002 tRNA 38489 38564 . + . ID=M1404_00042;Parent=M1404_00042_gene;inference=COORDINATES:profile:Aragorn:001002;locus_tag=M1404_00042;product=tRNA-Ala(ggc)

gnl|Prokka|M1404_1 prokka gene 38849 40993 . + . ID=M1404_00043_gene;Name=pnp;gene=pnp;locus_tag=M1404_00043

gnl|Prokka|M1404_1 Prodigal:002006 CDS 38849 40993 . + 0 ID=M1404_00043;Parent=M1404_00043_gene;eC_number=2.7.7.8;Name=pnp;db_xref=COG:COG1185;gene=pnp;inference=ab initio prediction:Prodigal:002006,similar to AA sequence:UniProtKB:P05055;locus_tag=M1404_00043;product=Polyribonucleotide nucleotidyltransferase;protein_id=gnl|Prokka|M1404_00043

gnl|Prokka|M1404_1 prokka gene 41188 41973 . + . ID=M1404_00044_gene;Name=nlpI;gene=nlpI;locus_tag=M1404_00044

gnl|Prokka|M1404_1 Prodigal:002006 CDS 41188 41973 . + 0 ID=M1404_00044;Parent=M1404_00044_gene;Name=nlpI;db_xref=COG:COG4785;gene=nlpI;inference=ab initio prediction:Prodigal:002006,similar to AA sequence:UniProtKB:P44585;locus_tag=M1404_00044;product=Lipoprotein NlpI ;protein_id=gnl|Prokka|M1404_00044

gnl|Prokka|M1404_1 prokka gene 42081 43913 . + . ID=M1404_00045_gene;Name=deaD;gene=deaD;locus_tag=M1404_00045

gnl|Prokka|M1404_1 Prodigal:002006 CDS 42081 43913 . + 0 ID=M1404_00045;Parent=M1404_00045_gene;eC_number=3.6.4.13;Name=deaD;db_xref=COG:COG0513;gene=deaD;inference=ab initio prediction:Prodigal:002006,similar to AA sequence:UniProtKB:P0A9P6;locus_tag=M1404_00045;product=ATP-dependent RNA helicase DeaD;protein_id=gnl|Prokka|M1404_00045

gnl|Prokka|M1404_1 prokka gene 44013 44861 . - . ID=M1404_00046_gene;Name=nadC;gene=nadC;locus_tag=M1404_00046

gnl|Prokka|M1404_1 Prodigal:002006 CDS 44013 44861 . - 0 ID=M1404_00046;Parent=M1404_00046_gene;eC_number=2.4.2.19;Name=nadC;db_xref=COG:COG0157;gene=nadC;inference=ab initio prediction:Prodigal:002006,similar to AA sequence:UniProtKB:P39666;locus_tag=M1404_00046;product=putative nicotinate-nucleotide pyrophosphorylase [carboxylating];protein_id=gnl|Prokka|M1404_00046

gnl|Prokka|M1404_1 prokka gene 44879 45478 . - . ID=M1404_00047_gene;Name=cysA;gene=cysA;locus_tag=M1404_00047

gnl|Prokka|M1404_1 Prodigal:002006 CDS 44879 45478 . - 0 ID=M1404_00047;Parent=M1404_00047_gene;eC_number=7.3.2.3;Name=cysA;db_xref=COG:COG1118;gene=cysA;inference=ab initio prediction:Prodigal:002006,similar to AA sequence:UniProtKB:P9WQM1;locus_tag=M1404_00047;product=Sulfate/thiosulfate import ATP-binding protein CysA;protein_id=gnl|Prokka|M1404_00047

gnl|Prokka|M1404_1 prokka gene 45480 46277 . - . ID=M1404_00048_gene;locus_tag=M1404_00048

gnl|Prokka|M1404_1 Prodigal:002006 CDS 45480 46277 . - 0 ID=M1404_00048;Parent=M1404_00048_gene;inference=ab initio prediction:Prodigal:002006;locus_tag=M1404_00048;product=hypothetical protein;protein_id=gnl|Prokka|M1404_00048

gnl|Prokka|M1404_1 prokka gene 46252 46989 . - . ID=M1404_00049_gene;locus_tag=M1404_00049

gnl|Prokka|M1404_1 Prodigal:002006 CDS 46252 46989 . - 0 ID=M1404_00049;Parent=M1404_00049_gene;db_xref=COG:COG0725;inference=ab initio prediction:Prodigal:002006,similar to AA sequence:UniProtKB:P71391;locus_tag=M1404_00049;product=Putative binding protein;protein_id=gnl|Prokka|M1404_00049

gnl|Prokka|M1404_1 prokka gene 47124 48767 . - . ID=M1404_00050_gene;Name=groL;gene=groL;locus_tag=M1404_00050

gnl|Prokka|M1404_1 Prodigal:002006 CDS 47124 48767 . - 0 ID=M1404_00050;Parent=M1404_00050_gene;Name=groL;db_xref=COG:COG0459;gene=groL;inference=ab initio prediction:Prodigal:002006,similar to AA sequence:UniProtKB:P46398;locus_tag=M1404_00050;product=60 kDa chaperonin;protein_id=gnl|Prokka|M1404_00050

gnl|Prokka|M1404_1 prokka gene 48795 48950 . - . ID=M1404_00051_gene;locus_tag=M1404_00051

gnl|Prokka|M1404_1 Prodigal:002006 CDS 48795 48950 . - 0 ID=M1404_00051;Parent=M1404_00051_gene;inference=ab initio prediction:Prodigal:002006;locus_tag=M1404_00051;product=hypothetical protein;protein_id=gnl|Prokka|M1404_00051

gnl|Prokka|M1404_1 prokka gene 49022 49312 . - . ID=M1404_00052_gene;Name=groS;gene=groS;locus_tag=M1404_00052

gnl|Prokka|M1404_1 Prodigal:002006 CDS 49022 49312 . - 0 ID=M1404_00052;Parent=M1404_00052_gene;Name=groS;db_xref=COG:COG0234;gene=groS;inference=ab initio prediction:Prodigal:002006,similar to AA sequence:UniProtKB:P0A6F9;locus_tag=M1404_00052;product=10 kDa chaperonin;protein_id=gnl|Prokka|M1404_00052

gnl|Prokka|M1404_1 prokka gene 49401 49871 . - . ID=M1404_00053_gene;locus_tag=M1404_00053

gnl|Prokka|M1404_1 Prodigal:002006 CDS 49401 49871 . - 0 ID=M1404_00053;Parent=M1404_00053_gene;inference=ab initio prediction:Prodigal:002006,similar to AA sequence:UniProtKB:P37147;locus_tag=M1404_00053;note=UPF0716 protein FxsA;product=hypothetical protein;protein_id=gnl|Prokka|M1404_00053

gnl|Prokka|M1404_1 prokka gene 50004 51575 . - . ID=M1404_00054_gene;Name=abgT;gene=abgT;locus_tag=M1404_00054

gnl|Prokka|M1404_1 Prodigal:002006 CDS 50004 51575 . - 0 ID=M1404_00054;Parent=M1404_00054_gene;Name=abgT;db_xref=COG:COG2978;gene=abgT;inference=ab initio prediction:Prodigal:002006,similar to AA sequence:UniProtKB:P46133;locus_tag=M1404_00054;product=p-aminobenzoyl-glutamate transport protein;protein_id=gnl|Prokka|M1404_00054

gnl|Prokka|M1404_1 prokka gene 51904 53322 . + . ID=M1404_00055_gene;Name=aspA;gene=aspA;locus_tag=M1404_00055

gnl|Prokka|M1404_1 Prodigal:002006 CDS 51904 53322 . + 0 ID=M1404_00055;Parent=M1404_00055_gene;eC_number=4.3.1.1;Name=aspA;db_xref=COG:COG1027;gene=aspA;inference=ab initio prediction:Prodigal:002006,similar to AA sequence:UniProtKB:P0AC38;locus_tag=M1404_00055;product=Aspartate ammonia-lyase;protein_id=gnl|Prokka|M1404_00055

gnl|Prokka|M1404_1 prokka gene 53458 55527 . - . ID=M1404_00056_gene;Name=glyS;gene=glyS;locus_tag=M1404_00056

gnl|Prokka|M1404_1 Prodigal:002006 CDS 53458 55527 . - 0 ID=M1404_00056;Parent=M1404_00056_gene;eC_number=6.1.1.14;Name=glyS;db_xref=COG:COG0751;gene=glyS;inference=ab initio prediction:Prodigal:002006,similar to AA sequence:UniProtKB:P00961;locus_tag=M1404_00056;product=Glycine--tRNA ligase beta subunit;protein_id=gnl|Prokka|M1404_00056

gnl|Prokka|M1404_1 prokka gene 55557 55937 . - . ID=M1404_00057_gene;locus_tag=M1404_00057

gnl|Prokka|M1404_1 Prodigal:002006 CDS 55557 55937 . - 0 ID=M1404_00057;Parent=M1404_00057_gene;inference=ab initio prediction:Prodigal:002006;locus_tag=M1404_00057;product=hypothetical protein;protein_id=gnl|Prokka|M1404_00057

gnl|Prokka|M1404_1 prokka gene 56068 56691 . - . ID=M1404_00058_gene;Name=yibF;gene=yibF;locus_tag=M1404_00058

gnl|Prokka|M1404_1 Prodigal:002006 CDS 56068 56691 . - 0 ID=M1404_00058;Parent=M1404_00058_gene;Name=yibF;db_xref=COG:COG0625;gene=yibF;inference=ab initio prediction:Prodigal:002006,similar to AA sequence:UniProtKB:P0ACA1;locus_tag=M1404_00058;product=putative GST-like protein YibF;protein_id=gnl|Prokka|M1404_00058

gnl|Prokka|M1404_1 prokka gene 56709 56972 . - . ID=M1404_00059_gene;locus_tag=M1404_00059

gnl|Prokka|M1404_1 Prodigal:002006 CDS 56709 56972 . - 0 ID=M1404_00059;Parent=M1404_00059_gene;inference=ab initio prediction:Prodigal:002006;locus_tag=M1404_00059;product=hypothetical protein;protein_id=gnl|Prokka|M1404_00059

gnl|Prokka|M1404_1 prokka gene 57003 57905 . - . ID=M1404_00060_gene;Name=glyQ;gene=glyQ;locus_tag=M1404_00060

gnl|Prokka|M1404_1 Prodigal:002006 CDS 57003 57905 . - 0 ID=M1404_00060;Parent=M1404_00060_gene;eC_number=6.1.1.14;Name=glyQ;db_xref=COG:COG0752;gene=glyQ;inference=ab initio prediction:Prodigal:002006,similar to AA sequence:UniProtKB:P00960;locus_tag=M1404_00060;product=Glycine--tRNA ligase alpha subunit;protein_id=gnl|Prokka|M1404_00060

gnl|Prokka|M1404_1 prokka gene 58187 59440 . + . ID=M1404_00061_gene;locus_tag=M1404_00061

gnl|Prokka|M1404_1 Prodigal:002006 CDS 58187 59440 . + 0 ID=M1404_00061;Parent=M1404_00061_gene;inference=ab initio prediction:Prodigal:002006;locus_tag=M1404_00061;product=hypothetical protein;protein_id=gnl|Prokka|M1404_00061

gnl|Prokka|M1404_1 prokka gene 59551 60408 . + . ID=M1404_00062_gene;Name=menB;gene=menB;locus_tag=M1404_00062

gnl|Prokka|M1404_1 Prodigal:002006 CDS 59551 60408 . + 0 ID=M1404_00062;Parent=M1404_00062_gene;eC_number=4.1.3.36;Name=menB;db_xref=COG:COG0447;gene=menB;inference=ab initio prediction:Prodigal:002006,similar to AA sequence:UniProtKB:Q7CQ56;locus_tag=M1404_00062;product=1%2C4-dihydroxy-2-naphthoyl-CoA synthase;protein_id=gnl|Prokka|M1404_00062

gnl|Prokka|M1404_1 prokka gene 60353 60466 . + . ID=M1404_00063_gene;locus_tag=M1404_00063

gnl|Prokka|M1404_1 Prodigal:002006 CDS 60353 60466 . + 0 ID=M1404_00063;Parent=M1404_00063_gene;inference=ab initio prediction:Prodigal:002006;locus_tag=M1404_00063;product=hypothetical protein;protein_id=gnl|Prokka|M1404_00063

gnl|Prokka|M1404_1 prokka gene 60491 60664 . + . ID=M1404_00064_gene;locus_tag=M1404_00064

gnl|Prokka|M1404_1 Prodigal:002006 CDS 60491 60664 . + 0 ID=M1404_00064;Parent=M1404_00064_gene;inference=ab initio prediction:Prodigal:002006;locus_tag=M1404_00064;product=hypothetical protein;protein_id=gnl|Prokka|M1404_00064

gnl|Prokka|M1404_1 prokka gene 60661 61674 . + . ID=M1404_00065_gene;Name=menC;gene=menC;locus_tag=M1404_00065

gnl|Prokka|M1404_1 Prodigal:002006 CDS 60661 61674 . + 0 ID=M1404_00065;Parent=M1404_00065_gene;eC_number=4.2.1.113;Name=menC;db_xref=COG:COG1441;gene=menC;inference=ab initio prediction:Prodigal:002006,similar to AA sequence:UniProtKB:P29208;locus_tag=M1404_00065;product=o-succinylbenzoate synthase;protein_id=gnl|Prokka|M1404_00065

gnl|Prokka|M1404_1 prokka gene 61745 62191 . + . ID=M1404_00066_gene;Name=aroQ;gene=aroQ;locus_tag=M1404_00066

gnl|Prokka|M1404_1 Prodigal:002006 CDS 61745 62191 . + 0 ID=M1404_00066;Parent=M1404_00066_gene;eC_number=4.2.1.10;Name=aroQ;db_xref=COG:COG0757;gene=aroQ;inference=ab initio prediction:Prodigal:002006,similar to AA sequence:UniProtKB:P43877;locus_tag=M1404_00066;product=3-dehydroquinate dehydratase;protein_id=gnl|Prokka|M1404_00066

gnl|Prokka|M1404_1 prokka gene 62346 62807 . + . ID=M1404_00067_gene;Name=accB;gene=accB;locus_tag=M1404_00067

gnl|Prokka|M1404_1 Prodigal:002006 CDS 62346 62807 . + 0 ID=M1404_00067;Parent=M1404_00067_gene;Name=accB;db_xref=COG:COG0511;gene=accB;inference=ab initio prediction:Prodigal:002006,similar to AA sequence:UniProtKB:P0ABD8;locus_tag=M1404_00067;product=Biotin carboxyl carrier protein of acetyl-CoA carboxylase;protein_id=gnl|Prokka|M1404_00067

gnl|Prokka|M1404_1 prokka gene 62909 64255 . + . ID=M1404_00068_gene;Name=accC;gene=accC;locus_tag=M1404_00068

gnl|Prokka|M1404_1 Prodigal:002006 CDS 62909 64255 . + 0 ID=M1404_00068;Parent=M1404_00068_gene;eC_number=6.3.4.14;Name=accC;db_xref=COG:COG0439;gene=accC;inference=ab initio prediction:Prodigal:002006,similar to AA sequence:UniProtKB:P43873;locus_tag=M1404_00068;product=Biotin carboxylase;protein_id=gnl|Prokka|M1404_00068

gnl|Prokka|M1404_1 prokka gene 64338 64589 . + . ID=M1404_00069_gene;Name=yhdT;gene=yhdT;locus_tag=M1404_00069

gnl|Prokka|M1404_1 Prodigal:002006 CDS 64338 64589 . + 0 ID=M1404_00069;Parent=M1404_00069_gene;Name=yhdT;db_xref=COG:COG3924;gene=yhdT;inference=ab initio prediction:Prodigal:002006,similar to AA sequence:UniProtKB:P45566;locus_tag=M1404_00069;product=putative membrane protein YhdT;protein_id=gnl|Prokka|M1404_00069

gnl|Prokka|M1404_1 prokka gene 64579 66012 . + . ID=M1404_00070_gene;Name=panF;gene=panF;locus_tag=M1404_00070

gnl|Prokka|M1404_1 Prodigal:002006 CDS 64579 66012 . + 0 ID=M1404_00070;Parent=M1404_00070_gene;Name=panF;db_xref=COG:COG4145;gene=panF;inference=ab initio prediction:Prodigal:002006,similar to AA sequence:UniProtKB:P16256;locus_tag=M1404_00070;product=Sodium/pantothenate symporter;protein_id=gnl|Prokka|M1404_00070

gnl|Prokka|M1404_1 prokka gene 66155 67036 . + . ID=M1404_00071_gene;Name=prmA;gene=prmA;locus_tag=M1404_00071

gnl|Prokka|M1404_1 Prodigal:002006 CDS 66155 67036 . + 0 ID=M1404_00071;Parent=M1404_00071_gene;eC_number=2.1.1.-;Name=prmA;db_xref=COG:COG2264;gene=prmA;inference=ab initio prediction:Prodigal:002006,similar to AA sequence:UniProtKB:P0A8T1;locus_tag=M1404_00071;product=Ribosomal protein L11 methyltransferase;protein_id=gnl|Prokka|M1404_00071

gnl|Prokka|M1404_1 prokka gene 67319 68317 . + . ID=M1404_00072_gene;Name=dusB;gene=dusB;locus_tag=M1404_00072

gnl|Prokka|M1404_1 Prodigal:002006 CDS 67319 68317 . + 0 ID=M1404_00072;Parent=M1404_00072_gene;eC_number=1.3.1.-;Name=dusB;db_xref=COG:COG0042;gene=dusB;inference=ab initio prediction:Prodigal:002006,similar to AA sequence:UniProtKB:P0ABT5;locus_tag=M1404_00072;product=tRNA-dihydrouridine synthase B;protein_id=gnl|Prokka|M1404_00072

gnl|Prokka|M1404_1 prokka gene 68298 68597 . + . ID=M1404_00073_gene;Name=fis;gene=fis;locus_tag=M1404_00073

gnl|Prokka|M1404_1 Prodigal:002006 CDS 68298 68597 . + 0 ID=M1404_00073;Parent=M1404_00073_gene;Name=fis;db_xref=COG:COG2901;gene=fis;inference=ab initio prediction:Prodigal:002006,similar to AA sequence:UniProtKB:P0A6R3;locus_tag=M1404_00073;product=DNA-binding protein Fis;protein_id=gnl|Prokka|M1404_00073

gnl|Prokka|M1404_1 prokka gene 68823 72716 . + . ID=M1404_00074_gene;Name=purL;gene=purL;locus_tag=M1404_00074

gnl|Prokka|M1404_1 Prodigal:002006 CDS 68823 72716 . + 0 ID=M1404_00074;Parent=M1404_00074_gene;eC_number=6.3.5.3;Name=purL;db_xref=COG:COG0046;gene=purL;inference=ab initio prediction:Prodigal:002006,similar to AA sequence:UniProtKB:P15254;locus_tag=M1404_00074;product=Phosphoribosylformylglycinamidine synthase;protein_id=gnl|Prokka|M1404_00074

gnl|Prokka|M1404_1 prokka gene 72796 73047 . + . ID=M1404_00075_gene;locus_tag=M1404_00075

gnl|Prokka|M1404_1 Prodigal:002006 CDS 72796 73047 . + 0 ID=M1404_00075;Parent=M1404_00075_gene;inference=ab initio prediction:Prodigal:002006;locus_tag=M1404_00075;product=hypothetical protein;protein_id=gnl|Prokka|M1404_00075

gnl|Prokka|M1404_1 prokka gene 73203 74291 . + . ID=M1404_00076_gene;locus_tag=M1404_00076

gnl|Prokka|M1404_1 Prodigal:002006 CDS 73203 74291 . + 0 ID=M1404_00076;Parent=M1404_00076_gene;inference=ab initio prediction:Prodigal:002006;locus_tag=M1404_00076;product=hypothetical protein;protein_id=gnl|Prokka|M1404_00076

gnl|Prokka|M1404_1 prokka gene 74592 75125 . + . ID=M1404_00077_gene;locus_tag=M1404_00077

gnl|Prokka|M1404_1 Prodigal:002006 CDS 74592 75125 . + 0 ID=M1404_00077;Parent=M1404_00077_gene;inference=ab initio prediction:Prodigal:002006;locus_tag=M1404_00077;product=hypothetical protein;protein_id=gnl|Prokka|M1404_00077

gnl|Prokka|M1404_1 prokka gene 75428 75889 . + . ID=M1404_00078_gene;locus_tag=M1404_00078

gnl|Prokka|M1404_1 Prodigal:002006 CDS 75428 75889 . + 0 ID=M1404_00078;Parent=M1404_00078_gene;inference=ab initio prediction:Prodigal:002006;locus_tag=M1404_00078;product=hypothetical protein;protein_id=gnl|Prokka|M1404_00078

gnl|Prokka|M1404_1 prokka gene 75949 76974 . + . ID=M1404_00079_gene;locus_tag=M1404_00079

gnl|Prokka|M1404_1 Prodigal:002006 CDS 75949 76974 . + 0 ID=M1404_00079;Parent=M1404_00079_gene;inference=ab initio prediction:Prodigal:002006;locus_tag=M1404_00079;product=hypothetical protein;protein_id=gnl|Prokka|M1404_00079

gnl|Prokka|M1404_1 prokka gene 77440 79869 . + . ID=M1404_00080_gene;locus_tag=M1404_00080

gnl|Prokka|M1404_1 Prodigal:002006 CDS 77440 79869 . + 0 ID=M1404_00080;Parent=M1404_00080_gene;db_xref=COG:COG1629;inference=ab initio prediction:Prodigal:002006,similar to AA sequence:UniProtKB:Q9JZN9;locus_tag=M1404_00080;product=putative TonB-dependent receptor;protein_id=gnl|Prokka|M1404_00080

gnl|Prokka|M1404_1 prokka gene 80015 80752 . - . ID=M1404_00081_gene;Name=hmuV;gene=hmuV;locus_tag=M1404_00081

gnl|Prokka|M1404_1 Prodigal:002006 CDS 80015 80752 . - 0 ID=M1404_00081;Parent=M1404_00081_gene;eC_number=3.6.3.-;Name=hmuV;gene=hmuV;inference=ab initio prediction:Prodigal:002006,similar to AA sequence:UniProtKB:O70014;locus_tag=M1404_00081;product=Hemin import ATP-binding protein HmuV;protein_id=gnl|Prokka|M1404_00081

gnl|Prokka|M1404_1 prokka gene 80764 81708 . - . ID=M1404_00082_gene;Name=hmuU;gene=hmuU;locus_tag=M1404_00082

gnl|Prokka|M1404_1 Prodigal:002006 CDS 80764 81708 . - 0 ID=M1404_00082;Parent=M1404_00082_gene;Name=hmuU;db_xref=COG:COG0609;gene=hmuU;inference=ab initio prediction:Prodigal:002006,similar to AA sequence:UniProtKB:Q56992;locus_tag=M1404_00082;product=Hemin transport system permease protein HmuU;protein_id=gnl|Prokka|M1404_00082

gnl|Prokka|M1404_1 prokka gene 81721 82509 . - . ID=M1404_00083_gene;Name=hmuT;gene=hmuT;locus_tag=M1404_00083

gnl|Prokka|M1404_1 Prodigal:002006 CDS 81721 82509 . - 0 ID=M1404_00083;Parent=M1404_00083_gene;Name=hmuT;db_xref=COG:COG4558;gene=hmuT;inference=ab initio prediction:Prodigal:002006,similar to AA sequence:UniProtKB:Q56991;locus_tag=M1404_00083;product=Hemin-binding periplasmic protein HmuT;protein_id=gnl|Prokka|M1404_00083

gnl|Prokka|M1404_1 prokka gene 82818 83372 . - . ID=M1404_00084_gene;locus_tag=M1404_00084

gnl|Prokka|M1404_1 Prodigal:002006 CDS 82818 83372 . - 0 ID=M1404_00084;Parent=M1404_00084_gene;inference=ab initio prediction:Prodigal:002006;locus_tag=M1404_00084;product=hypothetical protein;protein_id=gnl|Prokka|M1404_00084

gnl|Prokka|M1404_1 prokka gene 83853 84932 . + . ID=M1404_00085_gene;locus_tag=M1404_00085

gnl|Prokka|M1404_1 Prodigal:002006 CDS 83853 84932 . + 0 ID=M1404_00085;Parent=M1404_00085_gene;inference=ab initio prediction:Prodigal:002006;locus_tag=M1404_00085;product=hypothetical protein;protein_id=gnl|Prokka|M1404_00085

gnl|Prokka|M1404_1 prokka gene 85022 86677 . - . ID=M1404_00086_gene;locus_tag=M1404_00086

gnl|Prokka|M1404_1 Prodigal:002006 CDS 85022 86677 . - 0 ID=M1404_00086;Parent=M1404_00086_gene;eC_number=5.4.2.8;db_xref=COG:COG1109;inference=ab initio prediction:Prodigal:002006,similar to AA sequence:UniProtKB:Q57290;locus_tag=M1404_00086;product=putative phosphomannomutase;protein_id=gnl|Prokka|M1404_00086

gnl|Prokka|M1404_1 prokka gene 86895 87449 . - . ID=M1404_00087_gene;locus_tag=M1404_00087

gnl|Prokka|M1404_1 Prodigal:002006 CDS 86895 87449 . - 0 ID=M1404_00087;Parent=M1404_00087_gene;inference=ab initio prediction:Prodigal:002006;locus_tag=M1404_00087;product=hypothetical protein;protein_id=gnl|Prokka|M1404_00087

gnl|Prokka|M1404_1 prokka gene 87596 89353 . - . ID=M1404_00088_gene;Name=bacA;gene=bacA;locus_tag=M1404_00088

gnl|Prokka|M1404_1 Prodigal:002006 CDS 87596 89353 . - 0 ID=M1404_00088;Parent=M1404_00088_gene;Name=bacA;db_xref=COG:COG4178;gene=bacA;inference=ab initio prediction:Prodigal:002006,similar to AA sequence:UniProtKB:P9WQI9;locus_tag=M1404_00088;product=Vitamin B12 transport ATP-binding protein BacA;protein_id=gnl|Prokka|M1404_00088

gnl|Prokka|M1404_1 prokka gene 89538 91199 . + . ID=M1404_00089_gene;Name=aspT;gene=aspT;locus_tag=M1404_00089

gnl|Prokka|M1404_1 Prodigal:002006 CDS 89538 91199 . + 0 ID=M1404_00089;Parent=M1404_00089_gene;Name=aspT;gene=aspT;inference=ab initio prediction:Prodigal:002006,similar to AA sequence:UniProtKB:Q8L3K8;locus_tag=M1404_00089;product=Aspartate/alanine antiporter;protein_id=gnl|Prokka|M1404_00089

gnl|Prokka|M1404_1 prokka gene 91247 91537 . - . ID=M1404_00090_gene;locus_tag=M1404_00090

gnl|Prokka|M1404_1 Prodigal:002006 CDS 91247 91537 . - 0 ID=M1404_00090;Parent=M1404_00090_gene;inference=ab initio prediction:Prodigal:002006,similar to AA sequence:UniProtKB:P32162;locus_tag=M1404_00090;note=UPF0381 protein YiiS;product=hypothetical protein;protein_id=gnl|Prokka|M1404_00090

gnl|Prokka|M1404_1 prokka gene 91782 93107 . + . ID=M1404_00091_gene;locus_tag=M1404_00091

gnl|Prokka|M1404_1 Prodigal:002006 CDS 91782 93107 . + 0 ID=M1404_00091;Parent=M1404_00091_gene;db_xref=COG:COG2067;inference=ab initio prediction:Prodigal:002006,similar to AA sequence:UniProtKB:P80603;locus_tag=M1404_00091;product=47 kDa outer membrane protein;protein_id=gnl|Prokka|M1404_00091

gnl|Prokka|M1404_1 prokka gene 93170 93712 . + . ID=M1404_00092_gene;Name=ogt;gene=ogt;locus_tag=M1404_00092

gnl|Prokka|M1404_1 Prodigal:002006 CDS 93170 93712 . + 0 ID=M1404_00092;Parent=M1404_00092_gene;eC_number=2.1.1.63;Name=ogt;db_xref=COG:COG0350;gene=ogt;inference=ab initio prediction:Prodigal:002006,similar to AA sequence:UniProtKB:P11742;locus_tag=M1404_00092;product=Methylated-DNA--protein-cysteine methyltransferase%2C constitutive;protein_id=gnl|Prokka|M1404_00092

gnl|Prokka|M1404_1 prokka gene 93722 94390 . + . ID=M1404_00093_gene;Name=mutH;gene=mutH;locus_tag=M1404_00093

gnl|Prokka|M1404_1 Prodigal:002006 CDS 93722 94390 . + 0 ID=M1404_00093;Parent=M1404_00093_gene;Name=mutH;db_xref=COG:COG3066;gene=mutH;inference=ab initio prediction:Prodigal:002006,similar to AA sequence:UniProtKB:P44688;locus_tag=M1404_00093;product=DNA mismatch repair protein MutH;protein_id=gnl|Prokka|M1404_00093

gnl|Prokka|M1404_1 prokka gene 94456 95184 . + . ID=M1404_00094_gene;locus_tag=M1404_00094

gnl|Prokka|M1404_1 Prodigal:002006 CDS 94456 95184 . + 0 ID=M1404_00094;Parent=M1404_00094_gene;inference=ab initio prediction:Prodigal:002006,similar to AA sequence:UniProtKB:P67127;locus_tag=M1404_00094;note=UPF0053 inner membrane protein YgdQ;product=hypothetical protein;protein_id=gnl|Prokka|M1404_00094

gnl|Prokka|M1404_1 prokka gene 95238 98030 . - . ID=M1404_00095_gene;Name=polA;gene=polA;locus_tag=M1404_00095

gnl|Prokka|M1404_1 Prodigal:002006 CDS 95238 98030 . - 0 ID=M1404_00095;Parent=M1404_00095_gene;eC_number=2.7.7.7;Name=polA;db_xref=COG:COG0258;gene=polA;inference=ab initio prediction:Prodigal:002006,similar to AA sequence:UniProtKB:P00582;locus_tag=M1404_00095;product=DNA polymerase I;protein_id=gnl|Prokka|M1404_00095

gnl|Prokka|M1404_1 prokka gene 98277 99095 . + . ID=M1404_00096_gene;Name=hel;gene=hel;locus_tag=M1404_00096

gnl|Prokka|M1404_1 Prodigal:002006 CDS 98277 99095 . + 0 ID=M1404_00096;Parent=M1404_00096_gene;Name=hel;db_xref=COG:COG2503;gene=hel;inference=ab initio prediction:Prodigal:002006,similar to AA sequence:UniProtKB:P26093;locus_tag=M1404_00096;product=Lipoprotein E;protein_id=gnl|Prokka|M1404_00096

gnl|Prokka|M1404_1 prokka gene 99424 99648 . + . ID=M1404_00097_gene;locus_tag=M1404_00097

gnl|Prokka|M1404_1 Prodigal:002006 CDS 99424 99648 . + 0 ID=M1404_00097;Parent=M1404_00097_gene;inference=ab initio prediction:Prodigal:002006;locus_tag=M1404_00097;product=hypothetical protein;protein_id=gnl|Prokka|M1404_00097

gnl|Prokka|M1404_1 prokka gene 99689 100207 . - . ID=M1404_00098_gene;locus_tag=M1404_00098

gnl|Prokka|M1404_1 Prodigal:002006 CDS 99689 100207 . - 0 ID=M1404_00098;Parent=M1404_00098_gene;inference=ab initio prediction:Prodigal:002006;locus_tag=M1404_00098;product=hypothetical protein;protein_id=gnl|Prokka|M1404_00098

gnl|Prokka|M1404_1 prokka gene 100371 100655 . - . ID=M1404_00099_gene;locus_tag=M1404_00099

gnl|Prokka|M1404_1 Prodigal:002006 CDS 100371 100655 . - 0 ID=M1404_00099;Parent=M1404_00099_gene;inference=ab initio prediction:Prodigal:002006;locus_tag=M1404_00099;product=hypothetical protein;protein_id=gnl|Prokka|M1404_00099

gnl|Prokka|M1404_1 prokka gene 100634 101182 . - . ID=M1404_00100_gene;locus_tag=M1404_00100

gnl|Prokka|M1404_1 Prodigal:002006 CDS 100634 101182 . - 0 ID=M1404_00100;Parent=M1404_00100_gene;inference=ab initio prediction:Prodigal:002006;locus_tag=M1404_00100;product=hypothetical protein;protein_id=gnl|Prokka|M1404_00100

gnl|Prokka|M1404_1 prokka gene 101185 103404 . - . ID=M1404_00101_gene;locus_tag=M1404_00101

gnl|Prokka|M1404_1 Prodigal:002006 CDS 101185 103404 . - 0 ID=M1404_00101;Parent=M1404_00101_gene;inference=ab initio prediction:Prodigal:002006;locus_tag=M1404_00101;product=hypothetical protein;protein_id=gnl|Prokka|M1404_00101

gnl|Prokka|M1404_1 prokka gene 103407 104006 . - . ID=M1404_00102_gene;locus_tag=M1404_00102

gnl|Prokka|M1404_1 Prodigal:002006 CDS 103407 104006 . - 0 ID=M1404_00102;Parent=M1404_00102_gene;inference=ab initio prediction:Prodigal:002006;locus_tag=M1404_00102;product=hypothetical protein;protein_id=gnl|Prokka|M1404_00102

gnl|Prokka|M1404_1 prokka gene 103997 105064 . - . ID=M1404_00103_gene;locus_tag=M1404_00103

gnl|Prokka|M1404_1 Prodigal:002006 CDS 103997 105064 . - 0 ID=M1404_00103;Parent=M1404_00103_gene;inference=ab initio prediction:Prodigal:002006;locus_tag=M1404_00103;product=hypothetical protein;protein_id=gnl|Prokka|M1404_00103

gnl|Prokka|M1404_1 prokka gene 105064 105474 . - . ID=M1404_00104_gene;locus_tag=M1404_00104

gnl|Prokka|M1404_1 Prodigal:002006 CDS 105064 105474 . - 0 ID=M1404_00104;Parent=M1404_00104_gene;inference=ab initio prediction:Prodigal:002006;locus_tag=M1404_00104;product=hypothetical protein;protein_id=gnl|Prokka|M1404_00104

gnl|Prokka|M1404_1 prokka gene 105485 106033 . - . ID=M1404_00105_gene;locus_tag=M1404_00105

gnl|Prokka|M1404_1 Prodigal:002006 CDS 105485 106033 . - 0 ID=M1404_00105;Parent=M1404_00105_gene;inference=ab initio prediction:Prodigal:002006;locus_tag=M1404_00105;product=hypothetical protein;protein_id=gnl|Prokka|M1404_00105

gnl|Prokka|M1404_1 prokka gene 106065 106544 . - . ID=M1404_00106_gene;locus_tag=M1404_00106

gnl|Prokka|M1404_1 Prodigal:002006 CDS 106065 106544 . - 0 ID=M1404_00106;Parent=M1404_00106_gene;inference=ab initio prediction:Prodigal:002006;locus_tag=M1404_00106;product=hypothetical protein;protein_id=gnl|Prokka|M1404_00106

gnl|Prokka|M1404_1 prokka gene 106548 106916 . - . ID=M1404_00107_gene;locus_tag=M1404_00107

gnl|Prokka|M1404_1 Prodigal:002006 CDS 106548 106916 . - 0 ID=M1404_00107;Parent=M1404_00107_gene;inference=ab initio prediction:Prodigal:002006;locus_tag=M1404_00107;product=hypothetical protein;protein_id=gnl|Prokka|M1404_00107

gnl|Prokka|M1404_1 prokka gene 106920 107315 . - . ID=M1404_00108_gene;locus_tag=M1404_00108

gnl|Prokka|M1404_1 Prodigal:002006 CDS 106920 107315 . - 0 ID=M1404_00108;Parent=M1404_00108_gene;inference=ab initio prediction:Prodigal:002006;locus_tag=M1404_00108;product=hypothetical protein;protein_id=gnl|Prokka|M1404_00108

gnl|Prokka|M1404_1 prokka gene 107315 108679 . - . ID=M1404_00109_gene;locus_tag=M1404_00109

gnl|Prokka|M1404_1 Prodigal:002006 CDS 107315 108679 . - 0 ID=M1404_00109;Parent=M1404_00109_gene;inference=ab initio prediction:Prodigal:002006;locus_tag=M1404_00109;product=hypothetical protein;protein_id=gnl|Prokka|M1404_00109

gnl|Prokka|M1404_1 prokka gene 108689 110596 . - . ID=M1404_00110_gene;locus_tag=M1404_00110

gnl|Prokka|M1404_1 Prodigal:002006 CDS 108689 110596 . - 0 ID=M1404_00110;Parent=M1404_00110_gene;inference=ab initio prediction:Prodigal:002006;locus_tag=M1404_00110;product=hypothetical protein;protein_id=gnl|Prokka|M1404_00110

gnl|Prokka|M1404_1 prokka gene 110757 111680 . + . ID=M1404_00111_gene;locus_tag=M1404_00111

gnl|Prokka|M1404_1 Prodigal:002006 CDS 110757 111680 . + 0 ID=M1404_00111;Parent=M1404_00111_gene;inference=ab initio prediction:Prodigal:002006;locus_tag=M1404_00111;product=hypothetical protein;protein_id=gnl|Prokka|M1404_00111

gnl|Prokka|M1404_1 prokka gene 111667 112944 . + . ID=M1404_00112_gene;locus_tag=M1404_00112

gnl|Prokka|M1404_1 Prodigal:002006 CDS 111667 112944 . + 0 ID=M1404_00112;Parent=M1404_00112_gene;inference=ab initio prediction:Prodigal:002006;locus_tag=M1404_00112;product=hypothetical protein;protein_id=gnl|Prokka|M1404_00112

gnl|Prokka|M1404_1 prokka gene 112999 113081 . - . ID=M1404_00113_gene;locus_tag=M1404_00113

gnl|Prokka|M1404_1 Aragorn:001002 tRNA 112999 113081 . - . ID=M1404_00113;Parent=M1404_00113_gene;inference=COORDINATES:profile:Aragorn:001002;locus_tag=M1404_00113;product=tRNA-Phe(aaa)

gnl|Prokka|M1404_1 prokka gene 113144 113578 . + . ID=M1404_00114_gene;locus_tag=M1404_00114

gnl|Prokka|M1404_1 Prodigal:002006 CDS 113144 113578 . + 0 ID=M1404_00114;Parent=M1404_00114_gene;inference=ab initio prediction:Prodigal:002006;locus_tag=M1404_00114;product=hypothetical protein;protein_id=gnl|Prokka|M1404_00114

gnl|Prokka|M1404_1 prokka gene 113809 114834 . + . ID=M1404_00115_gene;locus_tag=M1404_00115

gnl|Prokka|M1404_1 Prodigal:002006 CDS 113809 114834 . + 0 ID=M1404_00115;Parent=M1404_00115_gene;inference=ab initio prediction:Prodigal:002006;locus_tag=M1404_00115;product=hypothetical protein;protein_id=gnl|Prokka|M1404_00115

gnl|Prokka|M1404_1 prokka gene 114834 115706 . + . ID=M1404_00116_gene;locus_tag=M1404_00116

gnl|Prokka|M1404_1 Prodigal:002006 CDS 114834 115706 . + 0 ID=M1404_00116;Parent=M1404_00116_gene;inference=ab initio prediction:Prodigal:002006;locus_tag=M1404_00116;product=hypothetical protein;protein_id=gnl|Prokka|M1404_00116

gnl|Prokka|M1404_1 prokka gene 115828 116247 . + . ID=M1404_00117_gene;locus_tag=M1404_00117

gnl|Prokka|M1404_1 Prodigal:002006 CDS 115828 116247 . + 0 ID=M1404_00117;Parent=M1404_00117_gene;inference=ab initio prediction:Prodigal:002006,similar to AA sequence:UniProtKB:P44228;locus_tag=M1404_00117;product=Mu-like prophage FluMu protein gp35;protein_id=gnl|Prokka|M1404_00117

gnl|Prokka|M1404_1 prokka gene 116247 116492 . + . ID=M1404_00118_gene;locus_tag=M1404_00118

gnl|Prokka|M1404_1 Prodigal:002006 CDS 116247 116492 . + 0 ID=M1404_00118;Parent=M1404_00118_gene;inference=ab initio prediction:Prodigal:002006;locus_tag=M1404_00118;product=hypothetical protein;protein_id=gnl|Prokka|M1404_00118

gnl|Prokka|M1404_1 prokka gene 116714 117226 . + . ID=M1404_00119_gene;locus_tag=M1404_00119

gnl|Prokka|M1404_1 Prodigal:002006 CDS 116714 117226 . + 0 ID=M1404_00119;Parent=M1404_00119_gene;inference=ab initio prediction:Prodigal:002006;locus_tag=M1404_00119;product=hypothetical protein;protein_id=gnl|Prokka|M1404_00119

gnl|Prokka|M1404_1 prokka gene 117226 117417 . + . ID=M1404_00120_gene;locus_tag=M1404_00120

gnl|Prokka|M1404_1 Prodigal:002006 CDS 117226 117417 . + 0 ID=M1404_00120;Parent=M1404_00120_gene;inference=ab initio prediction:Prodigal:002006;locus_tag=M1404_00120;product=hypothetical protein;protein_id=gnl|Prokka|M1404_00120

gnl|Prokka|M1404_1 prokka gene 117417 118880 . + . ID=M1404_00121_gene;locus_tag=M1404_00121

gnl|Prokka|M1404_1 Prodigal:002006 CDS 117417 118880 . + 0 ID=M1404_00121;Parent=M1404_00121_gene;inference=ab initio prediction:Prodigal:002006;locus_tag=M1404_00121;product=hypothetical protein;protein_id=gnl|Prokka|M1404_00121

gnl|Prokka|M1404_1 prokka gene 118891 119244 . + . ID=M1404_00122_gene;locus_tag=M1404_00122

gnl|Prokka|M1404_1 Prodigal:002006 CDS 118891 119244 . + 0 ID=M1404_00122;Parent=M1404_00122_gene;inference=ab initio prediction:Prodigal:002006;locus_tag=M1404_00122;product=hypothetical protein;protein_id=gnl|Prokka|M1404_00122

gnl|Prokka|M1404_1 prokka gene 119247 119630 . + . ID=M1404_00123_gene;locus_tag=M1404_00123

gnl|Prokka|M1404_1 Prodigal:002006 CDS 119247 119630 . + 0 ID=M1404_00123;Parent=M1404_00123_gene;inference=ab initio prediction:Prodigal:002006;locus_tag=M1404_00123;product=hypothetical protein;protein_id=gnl|Prokka|M1404_00123

gnl|Prokka|M1404_1 prokka gene 119720 121627 . + . ID=M1404_00124_gene;locus_tag=M1404_00124

gnl|Prokka|M1404_1 Prodigal:002006 CDS 119720 121627 . + 0 ID=M1404_00124;Parent=M1404_00124_gene;inference=ab initio prediction:Prodigal:002006;locus_tag=M1404_00124;product=hypothetical protein;protein_id=gnl|Prokka|M1404_00124

gnl|Prokka|M1404_1 prokka gene 121637 123001 . + . ID=M1404_00125_gene;locus_tag=M1404_00125

gnl|Prokka|M1404_1 Prodigal:002006 CDS 121637 123001 . + 0 ID=M1404_00125;Parent=M1404_00125_gene;inference=ab initio prediction:Prodigal:002006;locus_tag=M1404_00125;product=hypothetical protein;protein_id=gnl|Prokka|M1404_00125

gnl|Prokka|M1404_1 prokka gene 123001 123396 . + . ID=M1404_00126_gene;locus_tag=M1404_00126

gnl|Prokka|M1404_1 Prodigal:002006 CDS 123001 123396 . + 0 ID=M1404_00126;Parent=M1404_00126_gene;inference=ab initio prediction:Prodigal:002006;locus_tag=M1404_00126;product=hypothetical protein;protein_id=gnl|Prokka|M1404_00126

gnl|Prokka|M1404_1 prokka gene 123400 123768 . + . ID=M1404_00127_gene;locus_tag=M1404_00127

gnl|Prokka|M1404_1 Prodigal:002006 CDS 123400 123768 . + 0 ID=M1404_00127;Parent=M1404_00127_gene;inference=ab initio prediction:Prodigal:002006;locus_tag=M1404_00127;product=hypothetical protein;protein_id=gnl|Prokka|M1404_00127

gnl|Prokka|M1404_1 prokka gene 123772 124251 . + . ID=M1404_00128_gene;locus_tag=M1404_00128

gnl|Prokka|M1404_1 Prodigal:002006 CDS 123772 124251 . + 0 ID=M1404_00128;Parent=M1404_00128_gene;inference=ab initio prediction:Prodigal:002006;locus_tag=M1404_00128;product=hypothetical protein;protein_id=gnl|Prokka|M1404_00128

gnl|Prokka|M1404_1 prokka gene 124283 124831 . + . ID=M1404_00129_gene;locus_tag=M1404_00129

gnl|Prokka|M1404_1 Prodigal:002006 CDS 124283 124831 . + 0 ID=M1404_00129;Parent=M1404_00129_gene;inference=ab initio prediction:Prodigal:002006;locus_tag=M1404_00129;product=hypothetical protein;protein_id=gnl|Prokka|M1404_00129

gnl|Prokka|M1404_1 prokka gene 124842 125252 . + . ID=M1404_00130_gene;locus_tag=M1404_00130

gnl|Prokka|M1404_1 Prodigal:002006 CDS 124842 125252 . + 0 ID=M1404_00130;Parent=M1404_00130_gene;inference=ab initio prediction:Prodigal:002006;locus_tag=M1404_00130;product=hypothetical protein;protein_id=gnl|Prokka|M1404_00130

gnl|Prokka|M1404_1 prokka gene 125252 126319 . + . ID=M1404_00131_gene;locus_tag=M1404_00131

gnl|Prokka|M1404_1 Prodigal:002006 CDS 125252 126319 . + 0 ID=M1404_00131;Parent=M1404_00131_gene;inference=ab initio prediction:Prodigal:002006;locus_tag=M1404_00131;product=hypothetical protein;protein_id=gnl|Prokka|M1404_00131

gnl|Prokka|M1404_1 prokka gene 126310 126909 . + . ID=M1404_00132_gene;locus_tag=M1404_00132

gnl|Prokka|M1404_1 Prodigal:002006 CDS 126310 126909 . + 0 ID=M1404_00132;Parent=M1404_00132_gene;inference=ab initio prediction:Prodigal:002006;locus_tag=M1404_00132;product=hypothetical protein;protein_id=gnl|Prokka|M1404_00132

gnl|Prokka|M1404_1 prokka gene 126912 129131 . + . ID=M1404_00133_gene;locus_tag=M1404_00133

gnl|Prokka|M1404_1 Prodigal:002006 CDS 126912 129131 . + 0 ID=M1404_00133;Parent=M1404_00133_gene;inference=ab initio prediction:Prodigal:002006;locus_tag=M1404_00133;product=hypothetical protein;protein_id=gnl|Prokka|M1404_00133

gnl|Prokka|M1404_1 prokka gene 129134 129682 . + . ID=M1404_00134_gene;locus_tag=M1404_00134

gnl|Prokka|M1404_1 Prodigal:002006 CDS 129134 129682 . + 0 ID=M1404_00134;Parent=M1404_00134_gene;inference=ab initio prediction:Prodigal:002006;locus_tag=M1404_00134;product=hypothetical protein;protein_id=gnl|Prokka|M1404_00134

gnl|Prokka|M1404_1 prokka gene 129661 129945 . + . ID=M1404_00135_gene;locus_tag=M1404_00135

gnl|Prokka|M1404_1 Prodigal:002006 CDS 129661 129945 . + 0 ID=M1404_00135;Parent=M1404_00135_gene;inference=ab initio prediction:Prodigal:002006;locus_tag=M1404_00135;product=hypothetical protein;protein_id=gnl|Prokka|M1404_00135

gnl|Prokka|M1404_1 prokka gene 130109 130627 . + . ID=M1404_00136_gene;locus_tag=M1404_00136

gnl|Prokka|M1404_1 Prodigal:002006 CDS 130109 130627 . + 0 ID=M1404_00136;Parent=M1404_00136_gene;inference=ab initio prediction:Prodigal:002006;locus_tag=M1404_00136;product=hypothetical protein;protein_id=gnl|Prokka|M1404_00136

gnl|Prokka|M1404_1 prokka gene 130668 130892 . - . ID=M1404_00137_gene;locus_tag=M1404_00137

gnl|Prokka|M1404_1 Prodigal:002006 CDS 130668 130892 . - 0 ID=M1404_00137;Parent=M1404_00137_gene;inference=ab initio prediction:Prodigal:002006;locus_tag=M1404_00137;product=hypothetical protein;protein_id=gnl|Prokka|M1404_00137

gnl|Prokka|M1404_1 prokka gene 131146 132246 . + . ID=M1404_00138_gene;locus_tag=M1404_00138

gnl|Prokka|M1404_1 Prodigal:002006 CDS 131146 132246 . + 0 ID=M1404_00138;Parent=M1404_00138_gene;inference=ab initio prediction:Prodigal:002006;locus_tag=M1404_00138;product=hypothetical protein;protein_id=gnl|Prokka|M1404_00138

gnl|Prokka|M1404_1 prokka gene 132299 134047 . + . ID=M1404_00139_gene;Name=msbA_1;gene=msbA_1;locus_tag=M1404_00139

gnl|Prokka|M1404_1 Prodigal:002006 CDS 132299 134047 . + 0 ID=M1404_00139;Parent=M1404_00139_gene;eC_number=7.5.2.6;Name=msbA_1;db_xref=COG:COG1132;gene=msbA_1;inference=ab initio prediction:Prodigal:002006,similar to AA sequence:UniProtKB:P63359;locus_tag=M1404_00139;product=Lipid A export ATP-binding/permease protein MsbA;protein_id=gnl|Prokka|M1404_00139

gnl|Prokka|M1404_1 prokka gene 134079 135056 . + . ID=M1404_00140_gene;Name=lpxK;gene=lpxK;locus_tag=M1404_00140

gnl|Prokka|M1404_1 Prodigal:002006 CDS 134079 135056 . + 0 ID=M1404_00140;Parent=M1404_00140_gene;eC_number=2.7.1.130;Name=lpxK;db_xref=COG:COG1663;gene=lpxK;inference=ab initio prediction:Prodigal:002006,similar to AA sequence:UniProtKB:P27300;locus_tag=M1404_00140;product=Tetraacyldisaccharide 4'-kinase;protein_id=gnl|Prokka|M1404_00140

gnl|Prokka|M1404_1 prokka gene 135068 135250 . + . ID=M1404_00141_gene;locus_tag=M1404_00141

gnl|Prokka|M1404_1 Prodigal:002006 CDS 135068 135250 . + 0 ID=M1404_00141;Parent=M1404_00141_gene;inference=ab initio prediction:Prodigal:002006,similar to AA sequence:UniProtKB:P0AAZ7;locus_tag=M1404_00141;note=UPF0434 protein YcaR;product=hypothetical protein;protein_id=gnl|Prokka|M1404_00141

gnl|Prokka|M1404_1 prokka gene 135252 136028 . + . ID=M1404_00142_gene;Name=kdsB;gene=kdsB;locus_tag=M1404_00142

gnl|Prokka|M1404_1 Prodigal:002006 CDS 135252 136028 . + 0 ID=M1404_00142;Parent=M1404_00142_gene;eC_number=2.7.7.38;Name=kdsB;db_xref=COG:COG1212;gene=kdsB;inference=ab initio prediction:Prodigal:002006,similar to AA sequence:UniProtKB:P44490;locus_tag=M1404_00142;product=3-deoxy-manno-octulosonate cytidylyltransferase;protein_id=gnl|Prokka|M1404_00142

gnl|Prokka|M1404_1 prokka gene 136062 137888 . + . ID=M1404_00143_gene;Name=uvrC;gene=uvrC;locus_tag=M1404_00143

gnl|Prokka|M1404_1 Prodigal:002006 CDS 136062 137888 . + 0 ID=M1404_00143;Parent=M1404_00143_gene;Name=uvrC;db_xref=COG:COG0322;gene=uvrC;inference=ab initio prediction:Prodigal:002006,similar to AA sequence:UniProtKB:P0A8G0;locus_tag=M1404_00143;product=UvrABC system protein C;protein_id=gnl|Prokka|M1404_00143

gnl|Prokka|M1404_1 prokka gene 137984 138541 . + . ID=M1404_00144_gene;Name=pgsA;gene=pgsA;locus_tag=M1404_00144

gnl|Prokka|M1404_1 Prodigal:002006 CDS 137984 138541 . + 0 ID=M1404_00144;Parent=M1404_00144_gene;eC_number=2.7.8.5;Name=pgsA;db_xref=COG:COG0558;gene=pgsA;inference=ab initio prediction:Prodigal:002006,similar to AA sequence:UniProtKB:P0ABF8;locus_tag=M1404_00144;product=CDP-diacylglycerol--glycerol-3-phosphate 3-phosphatidyltransferase;protein_id=gnl|Prokka|M1404_00144

gnl|Prokka|M1404_1 prokka gene 138730 138805 . + . ID=M1404_00145_gene;locus_tag=M1404_00145

gnl|Prokka|M1404_1 Aragorn:001002 tRNA 138730 138805 . + . ID=M1404_00145;Parent=M1404_00145_gene;inference=COORDINATES:profile:Aragorn:001002;locus_tag=M1404_00145;product=tRNA-Gly(gcc)

gnl|Prokka|M1404_1 prokka gene 138832 138918 . + . ID=M1404_00146_gene;locus_tag=M1404_00146

gnl|Prokka|M1404_1 Aragorn:001002 tRNA 138832 138918 . + . ID=M1404_00146;Parent=M1404_00146_gene;inference=COORDINATES:profile:Aragorn:001002;locus_tag=M1404_00146;product=tRNA-Leu(taa)

gnl|Prokka|M1404_1 prokka gene 140720 141511 . + . ID=M1404_00147_gene;locus_tag=M1404_00147

gnl|Prokka|M1404_1 Prodigal:002006 CDS 140720 141511 . + 0 ID=M1404_00147;Parent=M1404_00147_gene;inference=ab initio prediction:Prodigal:002006;locus_tag=M1404_00147;product=hypothetical protein;protein_id=gnl|Prokka|M1404_00147

gnl|Prokka|M1404_1 prokka gene 141511 142920 . + . ID=M1404_00148_gene;Name=sctC;gene=sctC;locus_tag=M1404_00148

gnl|Prokka|M1404_1 Prodigal:002006 CDS 141511 142920 . + 0 ID=M1404_00148;Parent=M1404_00148_gene;Name=sctC;gene=sctC;inference=ab initio prediction:Prodigal:002006,protein motif:HAMAP:MF_02219;locus_tag=M1404_00148;product=Type 3 secretion system secretin;protein_id=gnl|Prokka|M1404_00148

gnl|Prokka|M1404_1 prokka gene 142917 143420 . + . ID=M1404_00149_gene;locus_tag=M1404_00149

gnl|Prokka|M1404_1 Prodigal:002006 CDS 142917 143420 . + 0 ID=M1404_00149;Parent=M1404_00149_gene;inference=ab initio prediction:Prodigal:002006;locus_tag=M1404_00149;product=hypothetical protein;protein_id=gnl|Prokka|M1404_00149

gnl|Prokka|M1404_1 prokka gene 143438 144544 . + . ID=M1404_00150_gene;locus_tag=M1404_00150

gnl|Prokka|M1404_1 Prodigal:002006 CDS 143438 144544 . + 0 ID=M1404_00150;Parent=M1404_00150_gene;inference=ab initio prediction:Prodigal:002006;locus_tag=M1404_00150;product=hypothetical protein;protein_id=gnl|Prokka|M1404_00150

gnl|Prokka|M1404_1 prokka gene 144565 145842 . + . ID=M1404_00151_gene;locus_tag=M1404_00151

gnl|Prokka|M1404_1 Prodigal:002006 CDS 144565 145842 . + 0 ID=M1404_00151;Parent=M1404_00151_gene;inference=ab initio prediction:Prodigal:002006;locus_tag=M1404_00151;product=hypothetical protein;protein_id=gnl|Prokka|M1404_00151

gnl|Prokka|M1404_1 prokka gene 145846 146730 . + . ID=M1404_00152_gene;locus_tag=M1404_00152

gnl|Prokka|M1404_1 Prodigal:002006 CDS 145846 146730 . + 0 ID=M1404_00152;Parent=M1404_00152_gene;inference=ab initio prediction:Prodigal:002006;locus_tag=M1404_00152;product=hypothetical protein;protein_id=gnl|Prokka|M1404_00152

gnl|Prokka|M1404_1 prokka gene 146727 147581 . + . ID=M1404_00153_gene;locus_tag=M1404_00153

gnl|Prokka|M1404_1 Prodigal:002006 CDS 146727 147581 . + 0 ID=M1404_00153;Parent=M1404_00153_gene;inference=ab initio prediction:Prodigal:002006;locus_tag=M1404_00153;product=hypothetical protein;protein_id=gnl|Prokka|M1404_00153

gnl|Prokka|M1404_1 prokka gene 147571 148341 . + . ID=M1404_00154_gene;locus_tag=M1404_00154

gnl|Prokka|M1404_1 Prodigal:002006 CDS 147571 148341 . + 0 ID=M1404_00154;Parent=M1404_00154_gene;inference=ab initio prediction:Prodigal:002006;locus_tag=M1404_00154;product=hypothetical protein;protein_id=gnl|Prokka|M1404_00154

gnl|Prokka|M1404_1 prokka gene 148351 148926 . + . ID=M1404_00155_gene;locus_tag=M1404_00155

gnl|Prokka|M1404_1 Prodigal:002006 CDS 148351 148926 . + 0 ID=M1404_00155;Parent=M1404_00155_gene;inference=ab initio prediction:Prodigal:002006;locus_tag=M1404_00155;product=hypothetical protein;protein_id=gnl|Prokka|M1404_00155

gnl|Prokka|M1404_1 prokka gene 148968 149513 . + . ID=M1404_00156_gene;locus_tag=M1404_00156

gnl|Prokka|M1404_1 Prodigal:002006 CDS 148968 149513 . + 0 ID=M1404_00156;Parent=M1404_00156_gene;inference=ab initio prediction:Prodigal:002006;locus_tag=M1404_00156;product=hypothetical protein;protein_id=gnl|Prokka|M1404_00156

gnl|Prokka|M1404_1 prokka gene 149534 151303 . + . ID=M1404_00157_gene;locus_tag=M1404_00157

gnl|Prokka|M1404_1 Prodigal:002006 CDS 149534 151303 . + 0 ID=M1404_00157;Parent=M1404_00157_gene;inference=ab initio prediction:Prodigal:002006;locus_tag=M1404_00157;product=hypothetical protein;protein_id=gnl|Prokka|M1404_00157

gnl|Prokka|M1404_1 prokka gene 151569 151937 . - . ID=M1404_00158_gene;locus_tag=M1404_00158

gnl|Prokka|M1404_1 Prodigal:002006 CDS 151569 151937 . - 0 ID=M1404_00158;Parent=M1404_00158_gene;inference=ab initio prediction:Prodigal:002006;locus_tag=M1404_00158;product=hypothetical protein;protein_id=gnl|Prokka|M1404_00158

gnl|Prokka|M1404_1 prokka gene 152013 154694 . - . ID=M1404_00159_gene;Name=gyrA;gene=gyrA;locus_tag=M1404_00159

gnl|Prokka|M1404_1 Prodigal:002006 CDS 152013 154694 . - 0 ID=M1404_00159;Parent=M1404_00159_gene;eC_number=5.6.2.2;Name=gyrA;db_xref=COG:COG0188;gene=gyrA;inference=ab initio prediction:Prodigal:002006,similar to AA sequence:UniProtKB:P37411;locus_tag=M1404_00159;product=DNA gyrase subunit A;protein_id=gnl|Prokka|M1404_00159

gnl|Prokka|M1404_1 prokka gene 154847 155575 . + . ID=M1404_00160_gene;Name=ubiG;gene=ubiG;locus_tag=M1404_00160

gnl|Prokka|M1404_1 Prodigal:002006 CDS 154847 155575 . + 0 ID=M1404_00160;Parent=M1404_00160_gene;eC_number=2.1.1.222;Name=ubiG;db_xref=COG:COG2227;gene=ubiG;inference=ab initio prediction:Prodigal:002006,similar to AA sequence:UniProtKB:P17993;locus_tag=M1404_00160;product=Ubiquinone biosynthesis O-methyltransferase;protein_id=gnl|Prokka|M1404_00160

gnl|Prokka|M1404_1 prokka gene 155669 156991 . - . ID=M1404_00161_gene;Name=aroA;gene=aroA;locus_tag=M1404_00161

gnl|Prokka|M1404_1 Prodigal:002006 CDS 155669 156991 . - 0 ID=M1404_00161;Parent=M1404_00161_gene;eC_number=2.5.1.19;Name=aroA;db_xref=COG:COG0128;gene=aroA;inference=ab initio prediction:Prodigal:002006,similar to AA sequence:UniProtKB:P0A6D3;locus_tag=M1404_00161;product=3-phosphoshikimate 1-carboxyvinyltransferase;protein_id=gnl|Prokka|M1404_00161

gnl|Prokka|M1404_1 prokka gene 157026 158123 . - . ID=M1404_00162_gene;Name=hisC_1;gene=hisC_1;locus_tag=M1404_00162

gnl|Prokka|M1404_1 Prodigal:002006 CDS 157026 158123 . - 0 ID=M1404_00162;Parent=M1404_00162_gene;eC_number=2.6.1.9;Name=hisC_1;gene=hisC_1;inference=ab initio prediction:Prodigal:002006,similar to AA sequence:UniProtKB:P34037;locus_tag=M1404_00162;product=Histidinol-phosphate aminotransferase;protein_id=gnl|Prokka|M1404_00162

gnl|Prokka|M1404_1 prokka gene 158187 159269 . - . ID=M1404_00163_gene;Name=serC;gene=serC;locus_tag=M1404_00163

gnl|Prokka|M1404_1 Prodigal:002006 CDS 158187 159269 . - 0 ID=M1404_00163;Parent=M1404_00163_gene;eC_number=2.6.1.52;Name=serC;db_xref=COG:COG1932;gene=serC;inference=ab initio prediction:Prodigal:002006,similar to AA sequence:UniProtKB:P23721;locus_tag=M1404_00163;product=Phosphoserine aminotransferase;protein_id=gnl|Prokka|M1404_00163

gnl|Prokka|M1404_1 prokka gene 159462 159803 . + . ID=M1404_00164_gene;locus_tag=M1404_00164

gnl|Prokka|M1404_1 Prodigal:002006 CDS 159462 159803 . + 0 ID=M1404_00164;Parent=M1404_00164_gene;inference=ab initio prediction:Prodigal:002006,similar to AA sequence:UniProtKB:P44117;locus_tag=M1404_00164;note=UPF0265 protein HI_1168;product=hypothetical protein;protein_id=gnl|Prokka|M1404_00164

gnl|Prokka|M1404_1 prokka gene 159849 161066 . - . ID=M1404_00165_gene;Name=yjhB;gene=yjhB;locus_tag=M1404_00165

gnl|Prokka|M1404_1 Prodigal:002006 CDS 159849 161066 . - 0 ID=M1404_00165;Parent=M1404_00165_gene;Name=yjhB;gene=yjhB;inference=ab initio prediction:Prodigal:002006,similar to AA sequence:UniProtKB:P39352;locus_tag=M1404_00165;product=Putative metabolite transport protein YjhB;protein_id=gnl|Prokka|M1404_00165

gnl|Prokka|M1404_1 prokka gene 161457 162431 . + . ID=M1404_00166_gene;Name=manX;gene=manX;locus_tag=M1404_00166

gnl|Prokka|M1404_1 Prodigal:002006 CDS 161457 162431 . + 0 ID=M1404_00166;Parent=M1404_00166_gene;eC_number=2.7.1.191;Name=manX;db_xref=COG:COG2893;gene=manX;inference=ab initio prediction:Prodigal:002006,similar to AA sequence:UniProtKB:P69797;locus_tag=M1404_00166;product=PTS system mannose-specific EIIAB component;protein_id=gnl|Prokka|M1404_00166

gnl|Prokka|M1404_1 prokka gene 162456 163259 . + . ID=M1404_00167_gene;Name=manY;gene=manY;locus_tag=M1404_00167

gnl|Prokka|M1404_1 Prodigal:002006 CDS 162456 163259 . + 0 ID=M1404_00167;Parent=M1404_00167_gene;Name=manY;db_xref=COG:COG3715;gene=manY;inference=ab initio prediction:Prodigal:002006,similar to AA sequence:UniProtKB:P69801;locus_tag=M1404_00167;product=PTS system mannose-specific EIIC component;protein_id=gnl|Prokka|M1404_00167

gnl|Prokka|M1404_1 prokka gene 163274 164110 . + . ID=M1404_00168_gene;Name=manZ;gene=manZ;locus_tag=M1404_00168

gnl|Prokka|M1404_1 Prodigal:002006 CDS 163274 164110 . + 0 ID=M1404_00168;Parent=M1404_00168_gene;Name=manZ;db_xref=COG:COG3716;gene=manZ;inference=ab initio prediction:Prodigal:002006,similar to AA sequence:UniProtKB:P69805;locus_tag=M1404_00168;product=PTS system mannose-specific EIID component;protein_id=gnl|Prokka|M1404_00168

gnl|Prokka|M1404_1 prokka gene 164290 164721 . + . ID=M1404_00169_gene;locus_tag=M1404_00169

gnl|Prokka|M1404_1 Prodigal:002006 CDS 164290 164721 . + 0 ID=M1404_00169;Parent=M1404_00169_gene;inference=ab initio prediction:Prodigal:002006,similar to AA sequence:UniProtKB:P67601;locus_tag=M1404_00169;note=UPF0266 membrane protein YobD;product=hypothetical protein;protein_id=gnl|Prokka|M1404_00169

gnl|Prokka|M1404_1 prokka gene 164733 165935 . + . ID=M1404_00170_gene;Name=manA;gene=manA;locus_tag=M1404_00170

gnl|Prokka|M1404_1 Prodigal:002006 CDS 164733 165935 . + 0 ID=M1404_00170;Parent=M1404_00170_gene;eC_number=5.3.1.8;Name=manA;db_xref=COG:COG1482;gene=manA;inference=ab initio prediction:Prodigal:002006,similar to AA sequence:UniProtKB:P00946;locus_tag=M1404_00170;product=Mannose-6-phosphate isomerase;protein_id=gnl|Prokka|M1404_00170

gnl|Prokka|M1404_1 prokka gene 165970 167292 . - . ID=M1404_00171_gene;Name=argA;gene=argA;locus_tag=M1404_00171

gnl|Prokka|M1404_1 Prodigal:002006 CDS 165970 167292 . - 0 ID=M1404_00171;Parent=M1404_00171_gene;eC_number=2.3.1.1;Name=argA;db_xref=COG:COG0548;gene=argA;inference=ab initio prediction:Prodigal:002006,similar to AA sequence:UniProtKB:P0A6C5;locus_tag=M1404_00171;product=Amino-acid acetyltransferase;protein_id=gnl|Prokka|M1404_00171

gnl|Prokka|M1404_1 prokka gene 167415 167678 . - . ID=M1404_00172_gene;Name=grxA;gene=grxA;locus_tag=M1404_00172

gnl|Prokka|M1404_1 Prodigal:002006 CDS 167415 167678 . - 0 ID=M1404_00172;Parent=M1404_00172_gene;Name=grxA;db_xref=COG:COG0695;gene=grxA;inference=ab initio prediction:Prodigal:002006,similar to AA sequence:UniProtKB:P68688;locus_tag=M1404_00172;product=Glutaredoxin 1;protein_id=gnl|Prokka|M1404_00172

gnl|Prokka|M1404_1 prokka gene 167838 168764 . + . ID=M1404_00173_gene;Name=rimK;gene=rimK;locus_tag=M1404_00173

gnl|Prokka|M1404_1 Prodigal:002006 CDS 167838 168764 . + 0 ID=M1404_00173;Parent=M1404_00173_gene;eC_number=6.3.2.-;Name=rimK;db_xref=COG:COG0189;gene=rimK;inference=ab initio prediction:Prodigal:002006,similar to AA sequence:UniProtKB:P0C0U4;locus_tag=M1404_00173;product=Ribosomal protein S6--L-glutamate ligase;protein_id=gnl|Prokka|M1404_00173

gnl|Prokka|M1404_1 prokka gene 168780 169319 . + . ID=M1404_00174_gene;locus_tag=M1404_00174

gnl|Prokka|M1404_1 Prodigal:002006 CDS 168780 169319 . + 0 ID=M1404_00174;Parent=M1404_00174_gene;inference=ab initio prediction:Prodigal:002006,similar to AA sequence:UniProtKB:P0A8E1;locus_tag=M1404_00174;note=UPF0227 protein YcfP;product=hypothetical protein;protein_id=gnl|Prokka|M1404_00174

gnl|Prokka|M1404_1 prokka gene 169356 169967 . - . ID=M1404_00175_gene;locus_tag=M1404_00175

gnl|Prokka|M1404_1 Prodigal:002006 CDS 169356 169967 . - 0 ID=M1404_00175;Parent=M1404_00175_gene;inference=ab initio prediction:Prodigal:002006;locus_tag=M1404_00175;product=hypothetical protein;protein_id=gnl|Prokka|M1404_00175

gnl|Prokka|M1404_1 prokka gene 170060 171454 . - . ID=M1404_00176_gene;Name=fumC;gene=fumC;locus_tag=M1404_00176

gnl|Prokka|M1404_1 Prodigal:002006 CDS 170060 171454 . - 0 ID=M1404_00176;Parent=M1404_00176_gene;eC_number=4.2.1.2;Name=fumC;db_xref=COG:COG0114;gene=fumC;inference=ab initio prediction:Prodigal:002006,similar to AA sequence:UniProtKB:P05042;locus_tag=M1404_00176;product=Fumarate hydratase class II;protein_id=gnl|Prokka|M1404_00176

gnl|Prokka|M1404_1 prokka gene 171705 172133 . + . ID=M1404_00177_gene;Name=holC;gene=holC;locus_tag=M1404_00177

gnl|Prokka|M1404_1 Prodigal:002006 CDS 171705 172133 . + 0 ID=M1404_00177;Parent=M1404_00177_gene;eC_number=2.7.7.7;Name=holC;db_xref=COG:COG2927;gene=holC;inference=ab initio prediction:Prodigal:002006,similar to AA sequence:UniProtKB:P28905;locus_tag=M1404_00177;product=DNA polymerase III subunit chi;protein_id=gnl|Prokka|M1404_00177

gnl|Prokka|M1404_1 prokka gene 172143 172595 . + . ID=M1404_00178_gene;locus_tag=M1404_00178

gnl|Prokka|M1404_1 Prodigal:002006 CDS 172143 172595 . + 0 ID=M1404_00178;Parent=M1404_00178_gene;inference=ab initio prediction:Prodigal:002006;locus_tag=M1404_00178;product=hypothetical protein;protein_id=gnl|Prokka|M1404_00178

gnl|Prokka|M1404_1 prokka gene 172805 173302 . + . ID=M1404_00179_gene;Name=tpx;gene=tpx;locus_tag=M1404_00179

gnl|Prokka|M1404_1 Prodigal:002006 CDS 172805 173302 . + 0 ID=M1404_00179;Parent=M1404_00179_gene;eC_number=1.11.1.15;Name=tpx;db_xref=COG:COG2077;gene=tpx;inference=ab initio prediction:Prodigal:002006,similar to AA sequence:UniProtKB:Q57549;locus_tag=M1404_00179;product=Thiol peroxidase;protein_id=gnl|Prokka|M1404_00179

gnl|Prokka|M1404_1 prokka gene 173432 173950 . + . ID=M1404_00180_gene;locus_tag=M1404_00180

gnl|Prokka|M1404_1 Prodigal:002006 CDS 173432 173950 . + 0 ID=M1404_00180;Parent=M1404_00180_gene;inference=ab initio prediction:Prodigal:002006;locus_tag=M1404_00180;product=hypothetical protein;protein_id=gnl|Prokka|M1404_00180

gnl|Prokka|M1404_1 prokka gene 173966 176875 . + . ID=M1404_00181_gene;Name=valS;gene=valS;locus_tag=M1404_00181

gnl|Prokka|M1404_1 Prodigal:002006 CDS 173966 176875 . + 0 ID=M1404_00181;Parent=M1404_00181_gene;eC_number=6.1.1.9;Name=valS;db_xref=COG:COG0525;gene=valS;inference=ab initio prediction:Prodigal:002006,similar to AA sequence:UniProtKB:P07118;locus_tag=M1404_00181;product=Valine--tRNA ligase;protein_id=gnl|Prokka|M1404_00181

gnl|Prokka|M1404_1 prokka gene 176999 177478 . - . ID=M1404_00182_gene;locus_tag=M1404_00182

gnl|Prokka|M1404_1 Prodigal:002006 CDS 176999 177478 . - 0 ID=M1404_00182;Parent=M1404_00182_gene;db_xref=COG:COG0783;inference=ab initio prediction:Prodigal:002006,similar to AA sequence:UniProtKB:P45173;locus_tag=M1404_00182;product=putative protein;protein_id=gnl|Prokka|M1404_00182

gnl|Prokka|M1404_1 prokka gene 177697 179286 . - . ID=M1404_00183_gene;Name=prfC;gene=prfC;locus_tag=M1404_00183

gnl|Prokka|M1404_1 Prodigal:002006 CDS 177697 179286 . - 0 ID=M1404_00183;Parent=M1404_00183_gene;Name=prfC;db_xref=COG:COG4108;gene=prfC;inference=ab initio prediction:Prodigal:002006,similar to AA sequence:UniProtKB:P0A7I4;locus_tag=M1404_00183;product=Peptide chain release factor RF3;protein_id=gnl|Prokka|M1404_00183

gnl|Prokka|M1404_1 prokka gene 179464 180324 . + . ID=M1404_00184_gene;Name=purC;gene=purC;locus_tag=M1404_00184

gnl|Prokka|M1404_1 Prodigal:002006 CDS 179464 180324 . + 0 ID=M1404_00184;Parent=M1404_00184_gene;eC_number=6.3.2.6;Name=purC;db_xref=COG:COG0152;gene=purC;inference=ab initio prediction:Prodigal:002006,similar to AA sequence:UniProtKB:A0R4I0;locus_tag=M1404_00184;product=Phosphoribosylaminoimidazole-succinocarboxamide synthase;protein_id=gnl|Prokka|M1404_00184

gnl|Prokka|M1404_1 prokka gene 180396 181256 . + . ID=M1404_00185_gene;Name=yeeZ;gene=yeeZ;locus_tag=M1404_00185

gnl|Prokka|M1404_1 Prodigal:002006 CDS 180396 181256 . + 0 ID=M1404_00185;Parent=M1404_00185_gene;Name=yeeZ;db_xref=COG:COG0451;gene=yeeZ;inference=ab initio prediction:Prodigal:002006,similar to AA sequence:UniProtKB:P0AD12;locus_tag=M1404_00185;product=Protein YeeZ;protein_id=gnl|Prokka|M1404_00185

gnl|Prokka|M1404_1 prokka gene 181312 182649 . + . ID=M1404_00186_gene;Name=argG;gene=argG;locus_tag=M1404_00186

gnl|Prokka|M1404_1 Prodigal:002006 CDS 181312 182649 . + 0 ID=M1404_00186;Parent=M1404_00186_gene;eC_number=6.3.4.5;Name=argG;db_xref=COG:COG0137;gene=argG;inference=ab initio prediction:Prodigal:002006,similar to AA sequence:UniProtKB:Q7VTJ9;locus_tag=M1404_00186;product=Argininosuccinate synthase;protein_id=gnl|Prokka|M1404_00186

gnl|Prokka|M1404_1 prokka gene 182842 182970 . + . ID=M1404_00187_gene;locus_tag=M1404_00187

gnl|Prokka|M1404_1 Prodigal:002006 CDS 182842 182970 . + 0 ID=M1404_00187;Parent=M1404_00187_gene;inference=ab initio prediction:Prodigal:002006;locus_tag=M1404_00187;product=hypothetical protein;protein_id=gnl|Prokka|M1404_00187

gnl|Prokka|M1404_1 prokka gene 183451 184827 . + . ID=M1404_00188_gene;Name=tpl;gene=tpl;locus_tag=M1404_00188

gnl|Prokka|M1404_1 Prodigal:002006 CDS 183451 184827 . + 0 ID=M1404_00188;Parent=M1404_00188_gene;eC_number=4.1.99.2;Name=tpl;gene=tpl;inference=ab initio prediction:Prodigal:002006,similar to AA sequence:UniProtKB:P31011;locus_tag=M1404_00188;product=Tyrosine phenol-lyase;protein_id=gnl|Prokka|M1404_00188

gnl|Prokka|M1404_1 prokka gene 184922 186136 . + . ID=M1404_00189_gene;Name=tyrP_1;gene=tyrP_1;locus_tag=M1404_00189

gnl|Prokka|M1404_1 Prodigal:002006 CDS 184922 186136 . + 0 ID=M1404_00189;Parent=M1404_00189_gene;Name=tyrP_1;db_xref=COG:COG0814;gene=tyrP_1;inference=ab initio prediction:Prodigal:002006,similar to AA sequence:UniProtKB:P0AAD4;locus_tag=M1404_00189;product=Tyrosine-specific transport protein;protein_id=gnl|Prokka|M1404_00189

gnl|Prokka|M1404_1 prokka gene 186279 187151 . + . ID=M1404_00190_gene;locus_tag=M1404_00190

gnl|Prokka|M1404_1 Prodigal:002006 CDS 186279 187151 . + 0 ID=M1404_00190;Parent=M1404_00190_gene;db_xref=COG:COG2990;inference=ab initio prediction:Prodigal:002006,similar to AA sequence:UniProtKB:Q57144;locus_tag=M1404_00190;product=putative protein;protein_id=gnl|Prokka|M1404_00190

gnl|Prokka|M1404_1 prokka gene 187221 188225 . - . ID=M1404_00191_gene;Name=argF;gene=argF;locus_tag=M1404_00191

gnl|Prokka|M1404_1 Prodigal:002006 CDS 187221 188225 . - 0 ID=M1404_00191;Parent=M1404_00191_gene;eC_number=2.1.3.3;Name=argF;db_xref=COG:COG0078;gene=argF;inference=ab initio prediction:Prodigal:002006,similar to AA sequence:UniProtKB:Q8DCF5;locus_tag=M1404_00191;product=Ornithine carbamoyltransferase;protein_id=gnl|Prokka|M1404_00191

gnl|Prokka|M1404_1 prokka gene 188408 189721 . - . ID=M1404_00192_gene;Name=potE;gene=potE;locus_tag=M1404_00192

gnl|Prokka|M1404_1 Prodigal:002006 CDS 188408 189721 . - 0 ID=M1404_00192;Parent=M1404_00192_gene;Name=potE;db_xref=COG:COG0531;gene=potE;inference=ab initio prediction:Prodigal:002006,similar to AA sequence:UniProtKB:P0AAF1;locus_tag=M1404_00192;product=Putrescine transporter PotE;protein_id=gnl|Prokka|M1404_00192

gnl|Prokka|M1404_1 prokka gene 189778 191940 . - . ID=M1404_00193_gene;Name=speC;gene=speC;locus_tag=M1404_00193

gnl|Prokka|M1404_1 Prodigal:002006 CDS 189778 191940 . - 0 ID=M1404_00193;Parent=M1404_00193_gene;eC_number=4.1.1.17;Name=speC;db_xref=COG:COG1982;gene=speC;inference=ab initio prediction:Prodigal:002006,similar to AA sequence:UniProtKB:P21169;locus_tag=M1404_00193;product=Constitutive ornithine decarboxylase;protein_id=gnl|Prokka|M1404_00193

gnl|Prokka|M1404_1 prokka gene 192664 195435 . - . ID=M1404_00194_gene;locus_tag=M1404_00194

gnl|Prokka|M1404_1 Prodigal:002006 CDS 192664 195435 . - 0 ID=M1404_00194;Parent=M1404_00194_gene;inference=ab initio prediction:Prodigal:002006;locus_tag=M1404_00194;product=hypothetical protein;protein_id=gnl|Prokka|M1404_00194

gnl|Prokka|M1404_1 prokka gene 195518 197890 . - . ID=M1404_00195_gene;locus_tag=M1404_00195

gnl|Prokka|M1404_1 Prodigal:002006 CDS 195518 197890 . - 0 ID=M1404_00195;Parent=M1404_00195_gene;inference=ab initio prediction:Prodigal:002006;locus_tag=M1404_00195;product=hypothetical protein;protein_id=gnl|Prokka|M1404_00195

gnl|Prokka|M1404_1 prokka gene 198136 198819 . + . ID=M1404_00196_gene;Name=cmk;gene=cmk;locus_tag=M1404_00196

gnl|Prokka|M1404_1 Prodigal:002006 CDS 198136 198819 . + 0 ID=M1404_00196;Parent=M1404_00196_gene;eC_number=2.7.4.25;Name=cmk;db_xref=COG:COG0283;gene=cmk;inference=ab initio prediction:Prodigal:002006,similar to AA sequence:UniProtKB:P0A6I0;locus_tag=M1404_00196;product=Cytidylate kinase;protein_id=gnl|Prokka|M1404_00196

gnl|Prokka|M1404_1 prokka gene 198925 200580 . + . ID=M1404_00197_gene;Name=rpsA;gene=rpsA;locus_tag=M1404_00197

gnl|Prokka|M1404_1 Prodigal:002006 CDS 198925 200580 . + 0 ID=M1404_00197;Parent=M1404_00197_gene;Name=rpsA;db_xref=COG:COG0539;gene=rpsA;inference=ab initio prediction:Prodigal:002006,similar to AA sequence:UniProtKB:P0AG67;locus_tag=M1404_00197;product=30S ribosomal protein S1;protein_id=gnl|Prokka|M1404_00197

gnl|Prokka|M1404_1 prokka gene 200652 200936 . + . ID=M1404_00198_gene;Name=ihfB;gene=ihfB;locus_tag=M1404_00198

gnl|Prokka|M1404_1 Prodigal:002006 CDS 200652 200936 . + 0 ID=M1404_00198;Parent=M1404_00198_gene;Name=ihfB;db_xref=COG:COG0776;gene=ihfB;inference=ab initio prediction:Prodigal:002006,similar to AA sequence:UniProtKB:P0A6Y1;locus_tag=M1404_00198;product=Integration host factor subunit beta;protein_id=gnl|Prokka|M1404_00198

gnl|Prokka|M1404_1 prokka gene 201032 201328 . + . ID=M1404_00199_gene;Name=lapA;gene=lapA;locus_tag=M1404_00199

gnl|Prokka|M1404_1 Prodigal:002006 CDS 201032 201328 . + 0 ID=M1404_00199;Parent=M1404_00199_gene;Name=lapA;db_xref=COG:COG3771;gene=lapA;inference=ab initio prediction:Prodigal:002006,similar to AA sequence:UniProtKB:P0ACV4;locus_tag=M1404_00199;product=Lipopolysaccharide assembly protein A;protein_id=gnl|Prokka|M1404_00199

gnl|Prokka|M1404_1 prokka gene 201328 202515 . + . ID=M1404_00200_gene;Name=lapB;gene=lapB;locus_tag=M1404_00200

gnl|Prokka|M1404_1 Prodigal:002006 CDS 201328 202515 . + 0 ID=M1404_00200;Parent=M1404_00200_gene;Name=lapB;db_xref=COG:COG2956;gene=lapB;inference=ab initio prediction:Prodigal:002006,similar to AA sequence:UniProtKB:P0AB58;locus_tag=M1404_00200;product=Lipopolysaccharide assembly protein B;protein_id=gnl|Prokka|M1404_00200

gnl|Prokka|M1404_1 prokka gene 202552 203253 . + . ID=M1404_00201_gene;Name=pyrF;gene=pyrF;locus_tag=M1404_00201

gnl|Prokka|M1404_1 Prodigal:002006 CDS 202552 203253 . + 0 ID=M1404_00201;Parent=M1404_00201_gene;eC_number=4.1.1.23;Name=pyrF;db_xref=COG:COG0284;gene=pyrF;inference=ab initio prediction:Prodigal:002006,similar to AA sequence:UniProtKB:Q9KQT7;locus_tag=M1404_00201;product=Orotidine 5'-phosphate decarboxylase;protein_id=gnl|Prokka|M1404_00201

gnl|Prokka|M1404_1 prokka gene 203256 203573 . + . ID=M1404_00202_gene;Name=yciH;gene=yciH;locus_tag=M1404_00202

gnl|Prokka|M1404_1 Prodigal:002006 CDS 203256 203573 . + 0 ID=M1404_00202;Parent=M1404_00202_gene;Name=yciH;db_xref=COG:COG0023;gene=yciH;inference=ab initio prediction:Prodigal:002006,similar to AA sequence:UniProtKB:P08245;locus_tag=M1404_00202;product=putative protein YciH;protein_id=gnl|Prokka|M1404_00202

gnl|Prokka|M1404_1 prokka gene 203709 204311 . - . ID=M1404_00203_gene;Name=ahpC;gene=ahpC;locus_tag=M1404_00203

gnl|Prokka|M1404_1 Prodigal:002006 CDS 203709 204311 . - 0 ID=M1404_00203;Parent=M1404_00203_gene;eC_number=1.11.1.15;Name=ahpC;db_xref=COG:COG0450;gene=ahpC;inference=ab initio prediction:Prodigal:002006,similar to AA sequence:UniProtKB:P21762;locus_tag=M1404_00203;product=Alkyl hydroperoxide reductase C;protein_id=gnl|Prokka|M1404_00203

gnl|Prokka|M1404_1 prokka gene 204494 205690 . + . ID=M1404_00204_gene;Name=metC;gene=metC;locus_tag=M1404_00204

gnl|Prokka|M1404_1 Prodigal:002006 CDS 204494 205690 . + 0 ID=M1404_00204;Parent=M1404_00204_gene;eC_number=4.4.1.13;Name=metC;db_xref=COG:COG0626;gene=metC;inference=ab initio prediction:Prodigal:002006,similar to AA sequence:UniProtKB:P06721;locus_tag=M1404_00204;product=Cystathionine beta-lyase MetC;protein_id=gnl|Prokka|M1404_00204

gnl|Prokka|M1404_1 prokka gene 205783 207132 . - . ID=M1404_00205_gene;Name=ygbN_1;gene=ygbN_1;locus_tag=M1404_00205

gnl|Prokka|M1404_1 Prodigal:002006 CDS 205783 207132 . - 0 ID=M1404_00205;Parent=M1404_00205_gene;Name=ygbN_1;db_xref=COG:COG2610;gene=ygbN_1;inference=ab initio prediction:Prodigal:002006,similar to AA sequence:UniProtKB:Q46892;locus_tag=M1404_00205;product=Inner membrane permease YgbN;protein_id=gnl|Prokka|M1404_00205

gnl|Prokka|M1404_1 prokka gene 207385 207903 . + . ID=M1404_00206_gene;Name=gntK;gene=gntK;locus_tag=M1404_00206

gnl|Prokka|M1404_1 Prodigal:002006 CDS 207385 207903 . + 0 ID=M1404_00206;Parent=M1404_00206_gene;eC_number=2.7.1.12;Name=gntK;db_xref=COG:COG3265;gene=gntK;inference=ab initio prediction:Prodigal:002006,similar to AA sequence:UniProtKB:P46859;locus_tag=M1404_00206;product=Thermoresistant gluconokinase;protein_id=gnl|Prokka|M1404_00206

gnl|Prokka|M1404_1 prokka gene 207963 208961 . - . ID=M1404_00207_gene;Name=gntR;gene=gntR;locus_tag=M1404_00207

gnl|Prokka|M1404_1 Prodigal:002006 CDS 207963 208961 . - 0 ID=M1404_00207;Parent=M1404_00207_gene;Name=gntR;gene=gntR;inference=ab initio prediction:Prodigal:002006,similar to AA sequence:UniProtKB:P0ACP5;locus_tag=M1404_00207;product=HTH-type transcriptional regulator GntR;protein_id=gnl|Prokka|M1404_00207

gnl|Prokka|M1404_1 prokka gene 209031 210131 . + . ID=M1404_00208_gene;Name=selD;gene=selD;locus_tag=M1404_00208

gnl|Prokka|M1404_1 Prodigal:002006 CDS 209031 210131 . + 0 ID=M1404_00208;Parent=M1404_00208_gene;eC_number=2.7.9.3;Name=selD;db_xref=COG:COG0709;gene=selD;inference=ab initio prediction:Prodigal:002006,similar to AA sequence:UniProtKB:P16456;locus_tag=M1404_00208;product=Selenide%2C water dikinase;protein_id=gnl|Prokka|M1404_00208

gnl|Prokka|M1404_1 prokka gene 210181 211161 . - . ID=M1404_00209_gene;Name=hemH;gene=hemH;locus_tag=M1404_00209

gnl|Prokka|M1404_1 Prodigal:002006 CDS 210181 211161 . - 0 ID=M1404_00209;Parent=M1404_00209_gene;eC_number=4.99.1.1;Name=hemH;db_xref=COG:COG0276;gene=hemH;inference=ab initio prediction:Prodigal:002006,similar to AA sequence:UniProtKB:P23871;locus_tag=M1404_00209;product=Ferrochelatase;protein_id=gnl|Prokka|M1404_00209

gnl|Prokka|M1404_1 prokka gene 211154 211573 . - . ID=M1404_00210_gene;locus_tag=M1404_00210

gnl|Prokka|M1404_1 Prodigal:002006 CDS 211154 211573 . - 0 ID=M1404_00210;Parent=M1404_00210_gene;eC_number=3.1.2.-;db_xref=COG:COG2050;inference=ab initio prediction:Prodigal:002006,similar to AA sequence:UniProtKB:P45083;locus_tag=M1404_00210;product=Putative esterase;protein_id=gnl|Prokka|M1404_00210

gnl|Prokka|M1404_1 prokka gene 211575 214661 . - . ID=M1404_00211_gene;locus_tag=M1404_00211

gnl|Prokka|M1404_1 Prodigal:002006 CDS 211575 214661 . - 0 ID=M1404_00211;Parent=M1404_00211_gene;db_xref=COG:COG0247;inference=ab initio prediction:Prodigal:002006,similar to AA sequence:UniProtKB:Q57252;locus_tag=M1404_00211;product=putative protein;protein_id=gnl|Prokka|M1404_00211

gnl|Prokka|M1404_1 prokka gene 215195 216271 . + . ID=M1404_00212_gene;Name=ompA_1;gene=ompA_1;locus_tag=M1404_00212

gnl|Prokka|M1404_1 Prodigal:002006 CDS 215195 216271 . + 0 ID=M1404_00212;Parent=M1404_00212_gene;Name=ompA_1;gene=ompA_1;inference=ab initio prediction:Prodigal:002006,similar to AA sequence:UniProtKB:P38368;locus_tag=M1404_00212;product=Outer membrane protein P5;protein_id=gnl|Prokka|M1404_00212

gnl|Prokka|M1404_1 prokka gene 216368 216949 . + . ID=M1404_00213_gene;Name=lemA;gene=lemA;locus_tag=M1404_00213

gnl|Prokka|M1404_1 Prodigal:002006 CDS 216368 216949 . + 0 ID=M1404_00213;Parent=M1404_00213_gene;Name=lemA;db_xref=COG:COG1704;gene=lemA;inference=ab initio prediction:Prodigal:002006,similar to AA sequence:UniProtKB:A8AVK0;locus_tag=M1404_00213;product=Protein LemA;protein_id=gnl|Prokka|M1404_00213

gnl|Prokka|M1404_1 prokka gene 216976 217425 . + . ID=M1404_00214_gene;locus_tag=M1404_00214

gnl|Prokka|M1404_1 Prodigal:002006 CDS 216976 217425 . + 0 ID=M1404_00214;Parent=M1404_00214_gene;inference=ab initio prediction:Prodigal:002006;locus_tag=M1404_00214;product=hypothetical protein;protein_id=gnl|Prokka|M1404_00214

gnl|Prokka|M1404_1 prokka gene 217418 218182 . + . ID=M1404_00215_gene;locus_tag=M1404_00215

gnl|Prokka|M1404_1 Prodigal:002006 CDS 217418 218182 . + 0 ID=M1404_00215;Parent=M1404_00215_gene;inference=ab initio prediction:Prodigal:002006;locus_tag=M1404_00215;product=hypothetical protein;protein_id=gnl|Prokka|M1404_00215

gnl|Prokka|M1404_1 prokka gene 218267 218596 . + . ID=M1404_00216_gene;Name=grxD;gene=grxD;locus_tag=M1404_00216

gnl|Prokka|M1404_1 Prodigal:002006 CDS 218267 218596 . + 0 ID=M1404_00216;Parent=M1404_00216_gene;Name=grxD;db_xref=COG:COG0278;gene=grxD;inference=ab initio prediction:Prodigal:002006,similar to AA sequence:UniProtKB:P0AC69;locus_tag=M1404_00216;product=Glutaredoxin 4;protein_id=gnl|Prokka|M1404_00216

gnl|Prokka|M1404_1 prokka gene 218649 219305 . - . ID=M1404_00217_gene;Name=kpsT;gene=kpsT;locus_tag=M1404_00217

gnl|Prokka|M1404_1 Prodigal:002006 CDS 218649 219305 . - 0 ID=M1404_00217;Parent=M1404_00217_gene;Name=kpsT;gene=kpsT;inference=ab initio prediction:Prodigal:002006,similar to AA sequence:UniProtKB:P23888;locus_tag=M1404_00217;product=Polysialic acid transport ATP-binding protein KpsT;protein_id=gnl|Prokka|M1404_00217

gnl|Prokka|M1404_1 prokka gene 219302 220099 . - . ID=M1404_00218_gene;Name=kpsM;gene=kpsM;locus_tag=M1404_00218

gnl|Prokka|M1404_1 Prodigal:002006 CDS 219302 220099 . - 0 ID=M1404_00218;Parent=M1404_00218_gene;Name=kpsM;db_xref=COG:COG1682;gene=kpsM;inference=ab initio prediction:Prodigal:002006,similar to AA sequence:UniProtKB:P23889;locus_tag=M1404_00218;product=Polysialic acid transport protein KpsM;protein_id=gnl|Prokka|M1404_00218

gnl|Prokka|M1404_1 prokka gene 220099 221241 . - . ID=M1404_00219_gene;locus_tag=M1404_00219

gnl|Prokka|M1404_1 Prodigal:002006 CDS 220099 221241 . - 0 ID=M1404_00219;Parent=M1404_00219_gene;inference=ab initio prediction:Prodigal:002006;locus_tag=M1404_00219;product=hypothetical protein;protein_id=gnl|Prokka|M1404_00219

gnl|Prokka|M1404_1 prokka gene 221251 222432 . - . ID=M1404_00220_gene;locus_tag=M1404_00220

gnl|Prokka|M1404_1 Prodigal:002006 CDS 221251 222432 . - 0 ID=M1404_00220;Parent=M1404_00220_gene;inference=ab initio prediction:Prodigal:002006;locus_tag=M1404_00220;product=hypothetical protein;protein_id=gnl|Prokka|M1404_00220

gnl|Prokka|M1404_1 prokka gene 222571 224619 . - . ID=M1404_00221_gene;locus_tag=M1404_00221

gnl|Prokka|M1404_1 Prodigal:002006 CDS 222571 224619 . - 0 ID=M1404_00221;Parent=M1404_00221_gene;inference=ab initio prediction:Prodigal:002006;locus_tag=M1404_00221;product=hypothetical protein;protein_id=gnl|Prokka|M1404_00221

gnl|Prokka|M1404_1 prokka gene 224619 225599 . - . ID=M1404_00222_gene;locus_tag=M1404_00222

gnl|Prokka|M1404_1 Prodigal:002006 CDS 224619 225599 . - 0 ID=M1404_00222;Parent=M1404_00222_gene;inference=ab initio prediction:Prodigal:002006;locus_tag=M1404_00222;product=hypothetical protein;protein_id=gnl|Prokka|M1404_00222

gnl|Prokka|M1404_1 prokka gene 225606 227771 . - . ID=M1404_00223_gene;locus_tag=M1404_00223

gnl|Prokka|M1404_1 Prodigal:002006 CDS 225606 227771 . - 0 ID=M1404_00223;Parent=M1404_00223_gene;inference=ab initio prediction:Prodigal:002006;locus_tag=M1404_00223;product=hypothetical protein;protein_id=gnl|Prokka|M1404_00223

gnl|Prokka|M1404_1 prokka gene 227773 229338 . - . ID=M1404_00224_gene;locus_tag=M1404_00224

gnl|Prokka|M1404_1 Prodigal:002006 CDS 227773 229338 . - 0 ID=M1404_00224;Parent=M1404_00224_gene;inference=ab initio prediction:Prodigal:002006;locus_tag=M1404_00224;product=hypothetical protein;protein_id=gnl|Prokka|M1404_00224

gnl|Prokka|M1404_1 prokka gene 229342 229716 . - . ID=M1404_00225_gene;locus_tag=M1404_00225

gnl|Prokka|M1404_1 Prodigal:002006 CDS 229342 229716 . - 0 ID=M1404_00225;Parent=M1404_00225_gene;inference=ab initio prediction:Prodigal:002006;locus_tag=M1404_00225;product=hypothetical protein;protein_id=gnl|Prokka|M1404_00225

gnl|Prokka|M1404_1 prokka gene 229731 230441 . - . ID=M1404_00226_gene;locus_tag=M1404_00226

gnl|Prokka|M1404_1 Prodigal:002006 CDS 229731 230441 . - 0 ID=M1404_00226;Parent=M1404_00226_gene;inference=ab initio prediction:Prodigal:002006;locus_tag=M1404_00226;product=hypothetical protein;protein_id=gnl|Prokka|M1404_00226

gnl|Prokka|M1404_1 prokka gene 230449 231876 . - . ID=M1404_00227_gene;locus_tag=M1404_00227

gnl|Prokka|M1404_1 Prodigal:002006 CDS 230449 231876 . - 0 ID=M1404_00227;Parent=M1404_00227_gene;inference=ab initio prediction:Prodigal:002006;locus_tag=M1404_00227;product=hypothetical protein;protein_id=gnl|Prokka|M1404_00227

gnl|Prokka|M1404_1 prokka gene 231869 234970 . - . ID=M1404_00228_gene;locus_tag=M1404_00228

gnl|Prokka|M1404_1 Prodigal:002006 CDS 231869 234970 . - 0 ID=M1404_00228;Parent=M1404_00228_gene;inference=ab initio prediction:Prodigal:002006;locus_tag=M1404_00228;product=hypothetical protein;protein_id=gnl|Prokka|M1404_00228

gnl|Prokka|M1404_1 prokka gene 235023 236291 . - . ID=M1404_00229_gene;Name=wecC;gene=wecC;locus_tag=M1404_00229

gnl|Prokka|M1404_1 Prodigal:002006 CDS 235023 236291 . - 0 ID=M1404_00229;Parent=M1404_00229_gene;eC_number=1.1.1.336;Name=wecC;db_xref=COG:COG0677;gene=wecC;inference=ab initio prediction:Prodigal:002006,similar to AA sequence:UniProtKB:P27829;locus_tag=M1404_00229;product=UDP-N-acetyl-D-mannosamine dehydrogenase;protein_id=gnl|Prokka|M1404_00229

gnl|Prokka|M1404_1 prokka gene 236310 237374 . - . ID=M1404_00230_gene;Name=wecB;gene=wecB;locus_tag=M1404_00230

gnl|Prokka|M1404_1 Prodigal:002006 CDS 236310 237374 . - 0 ID=M1404_00230;Parent=M1404_00230_gene;eC_number=5.1.3.14;Name=wecB;db_xref=COG:COG0381;gene=wecB;inference=ab initio prediction:Prodigal:002006,similar to AA sequence:UniProtKB:P27828;locus_tag=M1404_00230;product=UDP-N-acetylglucosamine 2-epimerase;protein_id=gnl|Prokka|M1404_00230

gnl|Prokka|M1404_1 prokka gene 237883 239019 . + . ID=M1404_00231_gene;locus_tag=M1404_00231

gnl|Prokka|M1404_1 Prodigal:002006 CDS 237883 239019 . + 0 ID=M1404_00231;Parent=M1404_00231_gene;inference=ab initio prediction:Prodigal:002006;locus_tag=M1404_00231;product=hypothetical protein;protein_id=gnl|Prokka|M1404_00231

gnl|Prokka|M1404_1 prokka gene 239055 239507 . + . ID=M1404_00232_gene;locus_tag=M1404_00232

gnl|Prokka|M1404_1 Prodigal:002006 CDS 239055 239507 . + 0 ID=M1404_00232;Parent=M1404_00232_gene;inference=ab initio prediction:Prodigal:002006;locus_tag=M1404_00232;product=hypothetical protein;protein_id=gnl|Prokka|M1404_00232

gnl|Prokka|M1404_1 prokka gene 239529 239912 . + . ID=M1404_00233_gene;locus_tag=M1404_00233

gnl|Prokka|M1404_1 Prodigal:002006 CDS 239529 239912 . + 0 ID=M1404_00233;Parent=M1404_00233_gene;inference=ab initio prediction:Prodigal:002006,similar to AA sequence:UniProtKB:P0ADR2;locus_tag=M1404_00233;note=UPF0382 inner membrane protein YgdD;product=hypothetical protein;protein_id=gnl|Prokka|M1404_00233

gnl|Prokka|M1404_1 prokka gene 239909 243823 . + . ID=M1404_00234_gene;locus_tag=M1404_00234

gnl|Prokka|M1404_1 Prodigal:002006 CDS 239909 243823 . + 0 ID=M1404_00234;Parent=M1404_00234_gene;inference=ab initio prediction:Prodigal:002006;locus_tag=M1404_00234;product=hypothetical protein;protein_id=gnl|Prokka|M1404_00234

gnl|Prokka|M1404_1 prokka gene 243960 244326 . + . ID=M1404_00235_gene;Name=ssrA;gene=ssrA;locus_tag=M1404_00235

gnl|Prokka|M1404_1 Aragorn:001002 tmRNA 243960 244326 . + . ID=M1404_00235;Parent=M1404_00235_gene;Name=ssrA;gene=ssrA;inference=COORDINATES:profile:Aragorn:001002;locus_tag=M1404_00235;product=transfer-messenger RNA%2C SsrA

gnl|Prokka|M1404_1 prokka gene 244869 245480 . + . ID=M1404_00236_gene;locus_tag=M1404_00236

gnl|Prokka|M1404_1 Prodigal:002006 CDS 244869 245480 . + 0 ID=M1404_00236;Parent=M1404_00236_gene;inference=ab initio prediction:Prodigal:002006;locus_tag=M1404_00236;product=hypothetical protein;protein_id=gnl|Prokka|M1404_00236

gnl|Prokka|M1404_1 prokka gene 245584 245796 . + . ID=M1404_00237_gene;locus_tag=M1404_00237

gnl|Prokka|M1404_1 Prodigal:002006 CDS 245584 245796 . + 0 ID=M1404_00237;Parent=M1404_00237_gene;inference=ab initio prediction:Prodigal:002006;locus_tag=M1404_00237;product=hypothetical protein;protein_id=gnl|Prokka|M1404_00237

gnl|Prokka|M1404_1 prokka gene 246199 246876 . - . ID=M1404_00238_gene;Name=sgbH_1;gene=sgbH_1;locus_tag=M1404_00238

gnl|Prokka|M1404_1 Prodigal:002006 CDS 246199 246876 . - 0 ID=M1404_00238;Parent=M1404_00238_gene;eC_number=4.1.1.85;Name=sgbH_1;db_xref=COG:COG0269;gene=sgbH_1;inference=ab initio prediction:Prodigal:002006,similar to AA sequence:UniProtKB:P37678;locus_tag=M1404_00238;product=3-keto-L-gulonate-6-phosphate decarboxylase SgbH;protein_id=gnl|Prokka|M1404_00238

gnl|Prokka|M1404_1 prokka gene 246894 247361 . - . ID=M1404_00239_gene;Name=ulaC;gene=ulaC;locus_tag=M1404_00239

gnl|Prokka|M1404_1 Prodigal:002006 CDS 246894 247361 . - 0 ID=M1404_00239;Parent=M1404_00239_gene;Name=ulaC;db_xref=COG:COG1762;gene=ulaC;inference=ab initio prediction:Prodigal:002006,similar to AA sequence:UniProtKB:P69820;locus_tag=M1404_00239;product=Ascorbate-specific PTS system EIIA component;protein_id=gnl|Prokka|M1404_00239

gnl|Prokka|M1404_1 prokka gene 247406 249196 . - . ID=M1404_00240_gene;locus_tag=M1404_00240

gnl|Prokka|M1404_1 Prodigal:002006 CDS 247406 249196 . - 0 ID=M1404_00240;Parent=M1404_00240_gene;inference=ab initio prediction:Prodigal:002006;locus_tag=M1404_00240;product=hypothetical protein;protein_id=gnl|Prokka|M1404_00240

gnl|Prokka|M1404_1 prokka gene 249546 250637 . + . ID=M1404_00241_gene;Name=ulaG;gene=ulaG;locus_tag=M1404_00241

gnl|Prokka|M1404_1 Prodigal:002006 CDS 249546 250637 . + 0 ID=M1404_00241;Parent=M1404_00241_gene;eC_number=3.1.1.-;Name=ulaG;db_xref=COG:COG2220;gene=ulaG;inference=ab initio prediction:Prodigal:002006,similar to AA sequence:UniProtKB:P39300;locus_tag=M1404_00241;product=putative L-ascorbate-6-phosphate lactonase UlaG;protein_id=gnl|Prokka|M1404_00241

gnl|Prokka|M1404_1 prokka gene 250732 251478 . + . ID=M1404_00242_gene;Name=ulaR;gene=ulaR;locus_tag=M1404_00242

gnl|Prokka|M1404_1 Prodigal:002006 CDS 250732 251478 . + 0 ID=M1404_00242;Parent=M1404_00242_gene;Name=ulaR;db_xref=COG:COG1349;gene=ulaR;inference=ab initio prediction:Prodigal:002006,similar to AA sequence:UniProtKB:P0A9W0;locus_tag=M1404_00242;product=HTH-type transcriptional regulator UlaR;protein_id=gnl|Prokka|M1404_00242

gnl|Prokka|M1404_1 prokka gene 251743 251819 . + . ID=M1404_00243_gene;locus_tag=M1404_00243

gnl|Prokka|M1404_1 Aragorn:001002 tRNA 251743 251819 . + . ID=M1404_00243;Parent=M1404_00243_gene;inference=COORDINATES:profile:Aragorn:001002;locus_tag=M1404_00243;product=tRNA-Met(cat)

gnl|Prokka|M1404_1 prokka gene 252028 252483 . + . ID=M1404_00244_gene;Name=rimP;gene=rimP;locus_tag=M1404_00244

gnl|Prokka|M1404_1 Prodigal:002006 CDS 252028 252483 . + 0 ID=M1404_00244;Parent=M1404_00244_gene;Name=rimP;db_xref=COG:COG0779;gene=rimP;inference=ab initio prediction:Prodigal:002006,similar to AA sequence:UniProtKB:P0A8A8;locus_tag=M1404_00244;product=Ribosome maturation factor RimP;protein_id=gnl|Prokka|M1404_00244

gnl|Prokka|M1404_1 prokka gene 252501 253985 . + . ID=M1404_00245_gene;Name=nusA;gene=nusA;locus_tag=M1404_00245

gnl|Prokka|M1404_1 Prodigal:002006 CDS 252501 253985 . + 0 ID=M1404_00245;Parent=M1404_00245_gene;Name=nusA;db_xref=COG:COG0195;gene=nusA;inference=ab initio prediction:Prodigal:002006,similar to AA sequence:UniProtKB:P0AFF6;locus_tag=M1404_00245;product=Transcription termination/antitermination protein NusA;protein_id=gnl|Prokka|M1404_00245

gnl|Prokka|M1404_1 prokka gene 254001 256502 . + . ID=M1404_00246_gene;Name=infB;gene=infB;locus_tag=M1404_00246

gnl|Prokka|M1404_1 Prodigal:002006 CDS 254001 256502 . + 0 ID=M1404_00246;Parent=M1404_00246_gene;Name=infB;db_xref=COG:COG0532;gene=infB;inference=ab initio prediction:Prodigal:002006,similar to AA sequence:UniProtKB:P0A705;locus_tag=M1404_00246;product=Translation initiation factor IF-2;protein_id=gnl|Prokka|M1404_00246

gnl|Prokka|M1404_1 prokka gene 256653 257423 . - . ID=M1404_00247_gene;locus_tag=M1404_00247

gnl|Prokka|M1404_1 Prodigal:002006 CDS 256653 257423 . - 0 ID=M1404_00247;Parent=M1404_00247_gene;inference=ab initio prediction:Prodigal:002006;locus_tag=M1404_00247;product=hypothetical protein;protein_id=gnl|Prokka|M1404_00247

gnl|Prokka|M1404_1 prokka gene 257639 258037 . + . ID=M1404_00248_gene;Name=rbfA;gene=rbfA;locus_tag=M1404_00248

gnl|Prokka|M1404_1 Prodigal:002006 CDS 257639 258037 . + 0 ID=M1404_00248;Parent=M1404_00248_gene;Name=rbfA;db_xref=COG:COG0858;gene=rbfA;inference=ab initio prediction:Prodigal:002006,similar to AA sequence:UniProtKB:P45141;locus_tag=M1404_00248;product=Ribosome-binding factor A;protein_id=gnl|Prokka|M1404_00248

gnl|Prokka|M1404_1 prokka gene 258037 258960 . + . ID=M1404_00249_gene;Name=truB;gene=truB;locus_tag=M1404_00249

gnl|Prokka|M1404_1 Prodigal:002006 CDS 258037 258960 . + 0 ID=M1404_00249;Parent=M1404_00249_gene;eC_number=5.4.99.25;Name=truB;db_xref=COG:COG0130;gene=truB;inference=ab initio prediction:Prodigal:002006,similar to AA sequence:UniProtKB:P60340;locus_tag=M1404_00249;product=tRNA pseudouridine synthase B;protein_id=gnl|Prokka|M1404_00249

gnl|Prokka|M1404_1 prokka gene 259046 260233 . - . ID=M1404_00250_gene;Name=tyrS;gene=tyrS;locus_tag=M1404_00250

gnl|Prokka|M1404_1 Prodigal:002006 CDS 259046 260233 . - 0 ID=M1404_00250;Parent=M1404_00250_gene;eC_number=6.1.1.1;Name=tyrS;db_xref=COG:COG0162;gene=tyrS;inference=ab initio prediction:Prodigal:002006,similar to AA sequence:UniProtKB:P41256;locus_tag=M1404_00250;product=Tyrosine--tRNA ligase;protein_id=gnl|Prokka|M1404_00250

gnl|Prokka|M1404_1 prokka gene 260433 260834 . + . ID=M1404_00251_gene;locus_tag=M1404_00251

gnl|Prokka|M1404_1 Prodigal:002006 CDS 260433 260834 . + 0 ID=M1404_00251;Parent=M1404_00251_gene;inference=ab initio prediction:Prodigal:002006;locus_tag=M1404_00251;product=hypothetical protein;protein_id=gnl|Prokka|M1404_00251

gnl|Prokka|M1404_1 prokka gene 261060 261782 . - . ID=M1404_00252_gene;Name=sfsA;gene=sfsA;locus_tag=M1404_00252

gnl|Prokka|M1404_1 Prodigal:002006 CDS 261060 261782 . - 0 ID=M1404_00252;Parent=M1404_00252_gene;Name=sfsA;db_xref=COG:COG1489;gene=sfsA;inference=ab initio prediction:Prodigal:002006,similar to AA sequence:UniProtKB:P0A823;locus_tag=M1404_00252;product=Sugar fermentation stimulation protein A;protein_id=gnl|Prokka|M1404_00252

gnl|Prokka|M1404_1 prokka gene 262155 263684 . + . ID=M1404_00253_gene;Name=pntA;gene=pntA;locus_tag=M1404_00253

gnl|Prokka|M1404_1 Prodigal:002006 CDS 262155 263684 . + 0 ID=M1404_00253;Parent=M1404_00253_gene;eC_number=7.1.1.1;Name=pntA;db_xref=COG:COG3288;gene=pntA;inference=ab initio prediction:Prodigal:002006,similar to AA sequence:UniProtKB:P07001;locus_tag=M1404_00253;product=NAD(P) transhydrogenase subunit alpha;protein_id=gnl|Prokka|M1404_00253

gnl|Prokka|M1404_1 prokka gene 263695 265119 . + . ID=M1404_00254_gene;Name=pntB;gene=pntB;locus_tag=M1404_00254

gnl|Prokka|M1404_1 Prodigal:002006 CDS 263695 265119 . + 0 ID=M1404_00254;Parent=M1404_00254_gene;eC_number=7.1.1.1;Name=pntB;db_xref=COG:COG1282;gene=pntB;inference=ab initio prediction:Prodigal:002006,similar to AA sequence:UniProtKB:P0AB67;locus_tag=M1404_00254;product=NAD(P) transhydrogenase subunit beta;protein_id=gnl|Prokka|M1404_00254

gnl|Prokka|M1404_1 prokka gene 265248 265841 . + . ID=M1404_00255_gene;locus_tag=M1404_00255

gnl|Prokka|M1404_1 Prodigal:002006 CDS 265248 265841 . + 0 ID=M1404_00255;Parent=M1404_00255_gene;inference=ab initio prediction:Prodigal:002006;locus_tag=M1404_00255;product=hypothetical protein;protein_id=gnl|Prokka|M1404_00255

gnl|Prokka|M1404_1 prokka gene 265932 266381 . + . ID=M1404_00256_gene;Name=nrdR;gene=nrdR;locus_tag=M1404_00256

gnl|Prokka|M1404_1 Prodigal:002006 CDS 265932 266381 . + 0 ID=M1404_00256;Parent=M1404_00256_gene;Name=nrdR;db_xref=COG:COG1327;gene=nrdR;inference=ab initio prediction:Prodigal:002006,similar to AA sequence:UniProtKB:P0A8D0;locus_tag=M1404_00256;product=Transcriptional repressor NrdR;protein_id=gnl|Prokka|M1404_00256

gnl|Prokka|M1404_1 prokka gene 266381 267511 . + . ID=M1404_00257_gene;Name=ribD;gene=ribD;locus_tag=M1404_00257

gnl|Prokka|M1404_1 Prodigal:002006 CDS 266381 267511 . + 0 ID=M1404_00257;Parent=M1404_00257_gene;Name=ribD;db_xref=COG:COG0117;gene=ribD;inference=ab initio prediction:Prodigal:002006,similar to AA sequence:UniProtKB:P25539;locus_tag=M1404_00257;product=Riboflavin biosynthesis protein RibD;protein_id=gnl|Prokka|M1404_00257

gnl|Prokka|M1404_1 prokka gene 267508 268536 . + . ID=M1404_00258_gene;Name=degS;gene=degS;locus_tag=M1404_00258

gnl|Prokka|M1404_1 Prodigal:002006 CDS 267508 268536 . + 0 ID=M1404_00258;Parent=M1404_00258_gene;eC_number=3.4.21.107;Name=degS;db_xref=COG:COG0265;gene=degS;inference=ab initio prediction:Prodigal:002006,similar to AA sequence:UniProtKB:P0AEE3;locus_tag=M1404_00258;product=Serine endoprotease DegS;protein_id=gnl|Prokka|M1404_00258

gnl|Prokka|M1404_1 prokka gene 268556 269155 . + . ID=M1404_00259_gene;Name=yfbR;gene=yfbR;locus_tag=M1404_00259

gnl|Prokka|M1404_1 Prodigal:002006 CDS 268556 269155 . + 0 ID=M1404_00259;Parent=M1404_00259_gene;eC_number=3.1.3.89;Name=yfbR;db_xref=COG:COG1896;gene=yfbR;inference=ab initio prediction:Prodigal:002006,similar to AA sequence:UniProtKB:P76491;locus_tag=M1404_00259;product=5'-deoxynucleotidase YfbR;protein_id=gnl|Prokka|M1404_00259

gnl|Prokka|M1404_1 prokka gene 269198 269989 . - . ID=M1404_00260_gene;Name=modE;gene=modE;locus_tag=M1404_00260

gnl|Prokka|M1404_1 Prodigal:002006 CDS 269198 269989 . - 0 ID=M1404_00260;Parent=M1404_00260_gene;Name=modE;db_xref=COG:COG2005;gene=modE;inference=ab initio prediction:Prodigal:002006,similar to AA sequence:UniProtKB:P0A9G8;locus_tag=M1404_00260;product=DNA-binding transcriptional dual regulator ModE;protein_id=gnl|Prokka|M1404_00260

gnl|Prokka|M1404_1 prokka gene 270280 273006 . + . ID=M1404_00261_gene;Name=btuB_1;gene=btuB_1;locus_tag=M1404_00261

gnl|Prokka|M1404_1 Prodigal:002006 CDS 270280 273006 . + 0 ID=M1404_00261;Parent=M1404_00261_gene;Name=btuB_1;gene=btuB_1;inference=ab initio prediction:Prodigal:002006,protein motif:HAMAP:MF_01531;locus_tag=M1404_00261;product=Vitamin B12 transporter BtuB;protein_id=gnl|Prokka|M1404_00261

gnl|Prokka|M1404_1 prokka gene 273174 273938 . + . ID=M1404_00262_gene;Name=modA;gene=modA;locus_tag=M1404_00262

gnl|Prokka|M1404_1 Prodigal:002006 CDS 273174 273938 . + 0 ID=M1404_00262;Parent=M1404_00262_gene;Name=modA;db_xref=COG:COG0725;gene=modA;inference=ab initio prediction:Prodigal:002006,similar to AA sequence:UniProtKB:P45323;locus_tag=M1404_00262;product=Molybdate-binding protein ModA;protein_id=gnl|Prokka|M1404_00262

gnl|Prokka|M1404_1 prokka gene 274170 274898 . + . ID=M1404_00263_gene;Name=modB;gene=modB;locus_tag=M1404_00263

gnl|Prokka|M1404_1 Prodigal:002006 CDS 274170 274898 . + 0 ID=M1404_00263;Parent=M1404_00263_gene;Name=modB;db_xref=COG:COG4149;gene=modB;inference=ab initio prediction:Prodigal:002006,similar to AA sequence:UniProtKB:P0AF01;locus_tag=M1404_00263;product=Molybdenum transport system permease protein ModB;protein_id=gnl|Prokka|M1404_00263

gnl|Prokka|M1404_1 prokka gene 274885 275940 . + . ID=M1404_00264_gene;Name=btuD_1;gene=btuD_1;locus_tag=M1404_00264

gnl|Prokka|M1404_1 Prodigal:002006 CDS 274885 275940 . + 0 ID=M1404_00264;Parent=M1404_00264_gene;eC_number=7.6.2.8;Name=btuD_1;gene=btuD_1;inference=ab initio prediction:Prodigal:002006,protein motif:HAMAP:MF_01005;locus_tag=M1404_00264;product=Vitamin B12 import ATP-binding protein BtuD;protein_id=gnl|Prokka|M1404_00264

gnl|Prokka|M1404_1 prokka gene 276029 278383 . - . ID=M1404_00265_gene;Name=btuB_2;gene=btuB_2;locus_tag=M1404_00265

gnl|Prokka|M1404_1 Prodigal:002006 CDS 276029 278383 . - 0 ID=M1404_00265;Parent=M1404_00265_gene;Name=btuB_2;gene=btuB_2;inference=ab initio prediction:Prodigal:002006,protein motif:HAMAP:MF_01531;locus_tag=M1404_00265;product=Vitamin B12 transporter BtuB;protein_id=gnl|Prokka|M1404_00265

gnl|Prokka|M1404_1 prokka gene 278690 279808 . - . ID=M1404_00266_gene;Name=dnaJ;gene=dnaJ;locus_tag=M1404_00266

gnl|Prokka|M1404_1 Prodigal:002006 CDS 278690 279808 . - 0 ID=M1404_00266;Parent=M1404_00266_gene;Name=dnaJ;db_xref=COG:COG0484;gene=dnaJ;inference=ab initio prediction:Prodigal:002006,similar to AA sequence:UniProtKB:P08622;locus_tag=M1404_00266;product=Chaperone protein DnaJ;protein_id=gnl|Prokka|M1404_00266

gnl|Prokka|M1404_1 prokka gene 279856 280332 . - . ID=M1404_00267_gene;locus_tag=M1404_00267

gnl|Prokka|M1404_1 Prodigal:002006 CDS 279856 280332 . - 0 ID=M1404_00267;Parent=M1404_00267_gene;inference=ab initio prediction:Prodigal:002006;locus_tag=M1404_00267;product=hypothetical protein;protein_id=gnl|Prokka|M1404_00267

gnl|Prokka|M1404_1 prokka gene 280387 281655 . - . ID=M1404_00268_gene;locus_tag=M1404_00268

gnl|Prokka|M1404_1 Prodigal:002006 CDS 280387 281655 . - 0 ID=M1404_00268;Parent=M1404_00268_gene;eC_number=2.5.1.-;db_xref=COG:COG2873;inference=ab initio prediction:Prodigal:002006,similar to AA sequence:UniProtKB:Q9WZY4;locus_tag=M1404_00268;product=O-acetyl-L-homoserine sulfhydrylase;protein_id=gnl|Prokka|M1404_00268

gnl|Prokka|M1404_1 prokka gene 281729 282127 . - . ID=M1404_00269_gene;locus_tag=M1404_00269

gnl|Prokka|M1404_1 Prodigal:002006 CDS 281729 282127 . - 0 ID=M1404_00269;Parent=M1404_00269_gene;inference=ab initio prediction:Prodigal:002006;locus_tag=M1404_00269;product=hypothetical protein;protein_id=gnl|Prokka|M1404_00269

gnl|Prokka|M1404_1 prokka gene 282252 284156 . - . ID=M1404_00270_gene;Name=dnaK;gene=dnaK;locus_tag=M1404_00270

gnl|Prokka|M1404_1 Prodigal:002006 CDS 282252 284156 . - 0 ID=M1404_00270;Parent=M1404_00270_gene;Name=dnaK;db_xref=COG:COG0443;gene=dnaK;inference=ab initio prediction:Prodigal:002006,similar to AA sequence:UniProtKB:P0A6Y8;locus_tag=M1404_00270;product=Chaperone protein DnaK;protein_id=gnl|Prokka|M1404_00270

gnl|Prokka|M1404_1 prokka gene 284510 285937 . + . ID=M1404_00271_gene;Name=ydgA;gene=ydgA;locus_tag=M1404_00271

gnl|Prokka|M1404_1 Prodigal:002006 CDS 284510 285937 . + 0 ID=M1404_00271;Parent=M1404_00271_gene;Name=ydgA;db_xref=COG:COG5339;gene=ydgA;inference=ab initio prediction:Prodigal:002006,similar to AA sequence:UniProtKB:P77804;locus_tag=M1404_00271;product=Protein YdgA;protein_id=gnl|Prokka|M1404_00271

gnl|Prokka|M1404_1 prokka gene 286033 287412 . - . ID=M1404_00272_gene;Name=degQ;gene=degQ;locus_tag=M1404_00272

gnl|Prokka|M1404_1 Prodigal:002006 CDS 286033 287412 . - 0 ID=M1404_00272;Parent=M1404_00272_gene;eC_number=3.4.21.107;Name=degQ;db_xref=COG:COG0265;gene=degQ;inference=ab initio prediction:Prodigal:002006,similar to AA sequence:UniProtKB:P39099;locus_tag=M1404_00272;product=Periplasmic pH-dependent serine endoprotease DegQ;protein_id=gnl|Prokka|M1404_00272

gnl|Prokka|M1404_1 prokka gene 287634 288296 . + . ID=M1404_00273_gene;locus_tag=M1404_00273

gnl|Prokka|M1404_1 Prodigal:002006 CDS 287634 288296 . + 0 ID=M1404_00273;Parent=M1404_00273_gene;eC_number=1.-.-.-;db_xref=COG:COG0778;inference=ab initio prediction:Prodigal:002006,similar to AA sequence:UniProtKB:Q57431;locus_tag=M1404_00273;product=Putative NAD(P)H nitroreductase;protein_id=gnl|Prokka|M1404_00273

gnl|Prokka|M1404_1 prokka gene 288352 289563 . - . ID=M1404_00274_gene;Name=tyrP_2;gene=tyrP_2;locus_tag=M1404_00274

gnl|Prokka|M1404_1 Prodigal:002006 CDS 288352 289563 . - 0 ID=M1404_00274;Parent=M1404_00274_gene;Name=tyrP_2;db_xref=COG:COG0814;gene=tyrP_2;inference=ab initio prediction:Prodigal:002006,similar to AA sequence:UniProtKB:P0AAD4;locus_tag=M1404_00274;product=Tyrosine-specific transport protein;protein_id=gnl|Prokka|M1404_00274

gnl|Prokka|M1404_1 prokka gene 289777 290250 . + . ID=M1404_00275_gene;Name=ribE;gene=ribE;locus_tag=M1404_00275

gnl|Prokka|M1404_1 Prodigal:002006 CDS 289777 290250 . + 0 ID=M1404_00275;Parent=M1404_00275_gene;eC_number=2.5.1.78;Name=ribE;db_xref=COG:COG0054;gene=ribE;inference=ab initio prediction:Prodigal:002006,similar to AA sequence:UniProtKB:P61714;locus_tag=M1404_00275;product=6%2C7-dimethyl-8-ribityllumazine synthase;protein_id=gnl|Prokka|M1404_00275

gnl|Prokka|M1404_1 prokka gene 290256 290690 . + . ID=M1404_00276_gene;Name=nusB;gene=nusB;locus_tag=M1404_00276

gnl|Prokka|M1404_1 Prodigal:002006 CDS 290256 290690 . + 0 ID=M1404_00276;Parent=M1404_00276_gene;Name=nusB;db_xref=COG:COG0781;gene=nusB;inference=ab initio prediction:Prodigal:002006,similar to AA sequence:UniProtKB:P0A780;locus_tag=M1404_00276;product=Transcription antitermination protein NusB;protein_id=gnl|Prokka|M1404_00276

gnl|Prokka|M1404_1 prokka gene 290707 291699 . + . ID=M1404_00277_gene;Name=thiL;gene=thiL;locus_tag=M1404_00277

gnl|Prokka|M1404_1 Prodigal:002006 CDS 290707 291699 . + 0 ID=M1404_00277;Parent=M1404_00277_gene;eC_number=2.7.4.16;Name=thiL;db_xref=COG:COG0611;gene=thiL;inference=ab initio prediction:Prodigal:002006,similar to AA sequence:UniProtKB:P0AGG0;locus_tag=M1404_00277;product=Thiamine-monophosphate kinase;protein_id=gnl|Prokka|M1404_00277

gnl|Prokka|M1404_1 prokka gene 291709 292194 . + . ID=M1404_00278_gene;Name=pgpA;gene=pgpA;locus_tag=M1404_00278

gnl|Prokka|M1404_1 Prodigal:002006 CDS 291709 292194 . + 0 ID=M1404_00278;Parent=M1404_00278_gene;eC_number=3.1.3.27;Name=pgpA;db_xref=COG:COG1267;gene=pgpA;inference=ab initio prediction:Prodigal:002006,similar to AA sequence:UniProtKB:P18200;locus_tag=M1404_00278;product=Phosphatidylglycerophosphatase A;protein_id=gnl|Prokka|M1404_00278

gnl|Prokka|M1404_1 prokka gene 292203 292844 . + . ID=M1404_00279_gene;Name=rhtC;gene=rhtC;locus_tag=M1404_00279

gnl|Prokka|M1404_1 Prodigal:002006 CDS 292203 292844 . + 0 ID=M1404_00279;Parent=M1404_00279_gene;Name=rhtC;db_xref=COG:COG1280;gene=rhtC;inference=ab initio prediction:Prodigal:002006,similar to AA sequence:UniProtKB:P0AG38;locus_tag=M1404_00279;product=Threonine efflux protein;protein_id=gnl|Prokka|M1404_00279

gnl|Prokka|M1404_1 prokka gene 292899 293711 . + . ID=M1404_00280_gene;Name=dapB;gene=dapB;locus_tag=M1404_00280

gnl|Prokka|M1404_1 Prodigal:002006 CDS 292899 293711 . + 0 ID=M1404_00280;Parent=M1404_00280_gene;eC_number=1.17.1.8;Name=dapB;db_xref=COG:COG0289;gene=dapB;inference=ab initio prediction:Prodigal:002006,similar to AA sequence:UniProtKB:P04036;locus_tag=M1404_00280;product=4-hydroxy-tetrahydrodipicolinate reductase;protein_id=gnl|Prokka|M1404_00280

gnl|Prokka|M1404_1 prokka gene 293746 294324 . - . ID=M1404_00281_gene;Name=ttrR;gene=ttrR;locus_tag=M1404_00281

gnl|Prokka|M1404_1 Prodigal:002006 CDS 293746 294324 . - 0 ID=M1404_00281;Parent=M1404_00281_gene;Name=ttrR;db_xref=COG:COG4566;gene=ttrR;inference=ab initio prediction:Prodigal:002006,similar to AA sequence:UniProtKB:Q7CQM8;locus_tag=M1404_00281;product=Tetrathionate response regulatory protein TtrR;protein_id=gnl|Prokka|M1404_00281

gnl|Prokka|M1404_1 prokka gene 294318 296033 . - . ID=M1404_00282_gene;Name=ttrS;gene=ttrS;locus_tag=M1404_00282

gnl|Prokka|M1404_1 Prodigal:002006 CDS 294318 296033 . - 0 ID=M1404_00282;Parent=M1404_00282_gene;eC_number=2.7.13.3;Name=ttrS;db_xref=COG:COG0642;gene=ttrS;inference=ab initio prediction:Prodigal:002006,similar to AA sequence:UniProtKB:Q8ZPP6;locus_tag=M1404_00282;product=Tetrathionate sensor histidine kinase TtrS;protein_id=gnl|Prokka|M1404_00282

gnl|Prokka|M1404_1 prokka gene 296181 296918 . + . ID=M1404_00283_gene;Name=ttrB;gene=ttrB;locus_tag=M1404_00283

gnl|Prokka|M1404_1 Prodigal:002006 CDS 296181 296918 . + 0 ID=M1404_00283;Parent=M1404_00283_gene;Name=ttrB;db_xref=COG:COG0437;gene=ttrB;inference=ab initio prediction:Prodigal:002006,similar to AA sequence:UniProtKB:Q7CQM9;locus_tag=M1404_00283;product=Tetrathionate reductase subunit B;protein_id=gnl|Prokka|M1404_00283

gnl|Prokka|M1404_1 prokka gene 296915 297964 . + . ID=M1404_00284_gene;Name=ttrC;gene=ttrC;locus_tag=M1404_00284

gnl|Prokka|M1404_1 Prodigal:002006 CDS 296915 297964 . + 0 ID=M1404_00284;Parent=M1404_00284_gene;Name=ttrC;gene=ttrC;inference=ab initio prediction:Prodigal:002006,similar to AA sequence:UniProtKB:Q9Z4S7;locus_tag=M1404_00284;product=Tetrathionate reductase subunit C;protein_id=gnl|Prokka|M1404_00284

gnl|Prokka|M1404_1 prokka gene 297957 301046 . + . ID=M1404_00285_gene;Name=ttrA;gene=ttrA;locus_tag=M1404_00285

gnl|Prokka|M1404_1 Prodigal:002006 CDS 297957 301046 . + 0 ID=M1404_00285;Parent=M1404_00285_gene;eC_number=1.8.-.-;Name=ttrA;db_xref=COG:COG0243;gene=ttrA;inference=ab initio prediction:Prodigal:002006,similar to AA sequence:UniProtKB:Q9Z4S6;locus_tag=M1404_00285;product=Tetrathionate reductase subunit A;protein_id=gnl|Prokka|M1404_00285

gnl|Prokka|M1404_1 prokka gene 301048 301296 . - . ID=M1404_00286_gene;locus_tag=M1404_00286

gnl|Prokka|M1404_1 Prodigal:002006 CDS 301048 301296 . - 0 ID=M1404_00286;Parent=M1404_00286_gene;inference=ab initio prediction:Prodigal:002006;locus_tag=M1404_00286;product=hypothetical protein;protein_id=gnl|Prokka|M1404_00286

gnl|Prokka|M1404_1 prokka gene 301367 302497 . - . ID=M1404_00287_gene;Name=nrdB;gene=nrdB;locus_tag=M1404_00287

gnl|Prokka|M1404_1 Prodigal:002006 CDS 301367 302497 . - 0 ID=M1404_00287;Parent=M1404_00287_gene;eC_number=1.17.4.1;Name=nrdB;db_xref=COG:COG0208;gene=nrdB;inference=ab initio prediction:Prodigal:002006,similar to AA sequence:UniProtKB:P69924;locus_tag=M1404_00287;product=Ribonucleoside-diphosphate reductase 1 subunit beta;protein_id=gnl|Prokka|M1404_00287

gnl|Prokka|M1404_1 prokka gene 302759 302851 . - . ID=M1404_00288_gene;locus_tag=M1404_00288

gnl|Prokka|M1404_1 Prodigal:002006 CDS 302759 302851 . - 0 ID=M1404_00288;Parent=M1404_00288_gene;inference=ab initio prediction:Prodigal:002006;locus_tag=M1404_00288;product=hypothetical protein;protein_id=gnl|Prokka|M1404_00288

gnl|Prokka|M1404_1 prokka gene 302851 304359 . - . ID=M1404_00289_gene;locus_tag=M1404_00289

gnl|Prokka|M1404_1 Prodigal:002006 CDS 302851 304359 . - 0 ID=M1404_00289;Parent=M1404_00289_gene;inference=ab initio prediction:Prodigal:002006;locus_tag=M1404_00289;product=hypothetical protein;protein_id=gnl|Prokka|M1404_00289

gnl|Prokka|M1404_1 prokka gene 304672 306942 . - . ID=M1404_00290_gene;Name=nrdA;gene=nrdA;locus_tag=M1404_00290

gnl|Prokka|M1404_1 Prodigal:002006 CDS 304672 306942 . - 0 ID=M1404_00290;Parent=M1404_00290_gene;eC_number=1.17.4.1;Name=nrdA;db_xref=COG:COG0209;gene=nrdA;inference=ab initio prediction:Prodigal:002006,similar to AA sequence:UniProtKB:P00452;locus_tag=M1404_00290;product=Ribonucleoside-diphosphate reductase 1 subunit alpha;protein_id=gnl|Prokka|M1404_00290

gnl|Prokka|M1404_1 prokka gene 307339 308802 . - . ID=M1404_00291_gene;Name=dacB;gene=dacB;locus_tag=M1404_00291

gnl|Prokka|M1404_1 Prodigal:002006 CDS 307339 308802 . - 0 ID=M1404_00291;Parent=M1404_00291_gene;eC_number=3.4.16.4;Name=dacB;db_xref=COG:COG2027;gene=dacB;inference=ab initio prediction:Prodigal:002006,similar to AA sequence:UniProtKB:P45161;locus_tag=M1404_00291;product=D-alanyl-D-alanine carboxypeptidase DacB;protein_id=gnl|Prokka|M1404_00291

gnl|Prokka|M1404_1 prokka gene 308970 309446 . + . ID=M1404_00292_gene;Name=greA;gene=greA;locus_tag=M1404_00292

gnl|Prokka|M1404_1 Prodigal:002006 CDS 308970 309446 . + 0 ID=M1404_00292;Parent=M1404_00292_gene;Name=greA;db_xref=COG:COG0782;gene=greA;inference=ab initio prediction:Prodigal:002006,similar to AA sequence:UniProtKB:P0A6W5;locus_tag=M1404_00292;product=Transcription elongation factor GreA;protein_id=gnl|Prokka|M1404_00292

gnl|Prokka|M1404_1 prokka gene 309853 318099 . + . ID=M1404_00293_gene;locus_tag=M1404_00293

gnl|Prokka|M1404_1 Prodigal:002006 CDS 309853 318099 . + 0 ID=M1404_00293;Parent=M1404_00293_gene;inference=ab initio prediction:Prodigal:002006;locus_tag=M1404_00293;product=hypothetical protein;protein_id=gnl|Prokka|M1404_00293

gnl|Prokka|M1404_1 prokka gene 318272 318574 . - . ID=M1404_00294_gene;locus_tag=M1404_00294

gnl|Prokka|M1404_1 Prodigal:002006 CDS 318272 318574 . - 0 ID=M1404_00294;Parent=M1404_00294_gene;db_xref=COG:COG1534;inference=ab initio prediction:Prodigal:002006,similar to AA sequence:UniProtKB:P71376;locus_tag=M1404_00294;product=RNA-binding protein;protein_id=gnl|Prokka|M1404_00294

gnl|Prokka|M1404_1 prokka gene 318705 319664 . + . ID=M1404_00295_gene;locus_tag=M1404_00295

gnl|Prokka|M1404_1 Prodigal:002006 CDS 318705 319664 . + 0 ID=M1404_00295;Parent=M1404_00295_gene;inference=ab initio prediction:Prodigal:002006;locus_tag=M1404_00295;product=hypothetical protein;protein_id=gnl|Prokka|M1404_00295

gnl|Prokka|M1404_1 prokka gene 319735 320556 . - . ID=M1404_00296_gene;Name=yeaD_1;gene=yeaD_1;locus_tag=M1404_00296

gnl|Prokka|M1404_1 Prodigal:002006 CDS 319735 320556 . - 0 ID=M1404_00296;Parent=M1404_00296_gene;eC_number=5.1.3.15;Name=yeaD_1;db_xref=COG:COG0676;gene=yeaD_1;inference=ab initio prediction:Prodigal:002006,similar to AA sequence:UniProtKB:P39173;locus_tag=M1404_00296;product=Putative glucose-6-phosphate 1-epimerase;protein_id=gnl|Prokka|M1404_00296

gnl|Prokka|M1404_1 prokka gene 320655 322583 . + . ID=M1404_00297_gene;Name=yoaA;gene=yoaA;locus_tag=M1404_00297

gnl|Prokka|M1404_1 Prodigal:002006 CDS 320655 322583 . + 0 ID=M1404_00297;Parent=M1404_00297_gene;eC_number=3.6.4.12;Name=yoaA;db_xref=COG:COG1199;gene=yoaA;inference=ab initio prediction:Prodigal:002006,similar to AA sequence:UniProtKB:P76257;locus_tag=M1404_00297;product=putative ATP-dependent DNA helicase YoaA;protein_id=gnl|Prokka|M1404_00297

gnl|Prokka|M1404_1 prokka gene 322589 323308 . + . ID=M1404_00298_gene;Name=tsaB;gene=tsaB;locus_tag=M1404_00298

gnl|Prokka|M1404_1 Prodigal:002006 CDS 322589 323308 . + 0 ID=M1404_00298;Parent=M1404_00298_gene;Name=tsaB;db_xref=COG:COG1214;gene=tsaB;inference=ab initio prediction:Prodigal:002006,similar to AA sequence:UniProtKB:P43990;locus_tag=M1404_00298;product=tRNA threonylcarbamoyladenosine biosynthesis protein TsaB;protein_id=gnl|Prokka|M1404_00298

gnl|Prokka|M1404_1 prokka gene 323344 323883 . + . ID=M1404_00299_gene;Name=slp;gene=slp;locus_tag=M1404_00299

gnl|Prokka|M1404_1 Prodigal:002006 CDS 323344 323883 . + 0 ID=M1404_00299;Parent=M1404_00299_gene;Name=slp;db_xref=COG:COG3065;gene=slp;inference=ab initio prediction:Prodigal:002006,similar to AA sequence:UniProtKB:P37194;locus_tag=M1404_00299;product=Outer membrane protein Slp;protein_id=gnl|Prokka|M1404_00299

gnl|Prokka|M1404_1 prokka gene 324006 325694 . + . ID=M1404_00300_gene;Name=fadD;gene=fadD;locus_tag=M1404_00300

gnl|Prokka|M1404_1 Prodigal:002006 CDS 324006 325694 . + 0 ID=M1404_00300;Parent=M1404_00300_gene;eC_number=6.2.1.3;Name=fadD;db_xref=COG:COG0318;gene=fadD;inference=ab initio prediction:Prodigal:002006,similar to AA sequence:UniProtKB:P69451;locus_tag=M1404_00300;product=Long-chain-fatty-acid--CoA ligase;protein_id=gnl|Prokka|M1404_00300

gnl|Prokka|M1404_1 prokka gene 325749 326900 . + . ID=M1404_00301_gene;Name=rnd;gene=rnd;locus_tag=M1404_00301

gnl|Prokka|M1404_1 Prodigal:002006 CDS 325749 326900 . + 0 ID=M1404_00301;Parent=M1404_00301_gene;eC_number=3.1.13.5;Name=rnd;db_xref=COG:COG0349;gene=rnd;inference=ab initio prediction:Prodigal:002006,similar to AA sequence:UniProtKB:P09155;locus_tag=M1404_00301;product=Ribonuclease D;protein_id=gnl|Prokka|M1404_00301

gnl|Prokka|M1404_1 prokka gene 326974 329112 . - . ID=M1404_00302_gene;Name=pta;gene=pta;locus_tag=M1404_00302

gnl|Prokka|M1404_1 Prodigal:002006 CDS 326974 329112 . - 0 ID=M1404_00302;Parent=M1404_00302_gene;eC_number=2.3.1.8;Name=pta;db_xref=COG:COG0280;gene=pta;inference=ab initio prediction:Prodigal:002006,similar to AA sequence:UniProtKB:Q8ZND6;locus_tag=M1404_00302;product=Phosphate acetyltransferase;protein_id=gnl|Prokka|M1404_00302

gnl|Prokka|M1404_1 prokka gene 329181 330386 . - . ID=M1404_00303_gene;Name=ackA;gene=ackA;locus_tag=M1404_00303

gnl|Prokka|M1404_1 Prodigal:002006 CDS 329181 330386 . - 0 ID=M1404_00303;Parent=M1404_00303_gene;eC_number=2.7.2.1;Name=ackA;db_xref=COG:COG0282;gene=ackA;inference=ab initio prediction:Prodigal:002006,similar to AA sequence:UniProtKB:P0A6A3;locus_tag=M1404_00303;product=Acetate kinase;protein_id=gnl|Prokka|M1404_00303

gnl|Prokka|M1404_1 prokka gene 330673 331089 . + . ID=M1404_00304_gene;locus_tag=M1404_00304

gnl|Prokka|M1404_1 Prodigal:002006 CDS 330673 331089 . + 0 ID=M1404_00304;Parent=M1404_00304_gene;inference=ab initio prediction:Prodigal:002006,similar to AA sequence:UniProtKB:P0A8D9;locus_tag=M1404_00304;note=UPF0208 membrane protein YfbV;product=hypothetical protein;protein_id=gnl|Prokka|M1404_00304

gnl|Prokka|M1404_1 prokka gene 331271 331762 . + . ID=M1404_00305_gene;Name=cvpA;gene=cvpA;locus_tag=M1404_00305

gnl|Prokka|M1404_1 Prodigal:002006 CDS 331271 331762 . + 0 ID=M1404_00305;Parent=M1404_00305_gene;Name=cvpA;db_xref=COG:COG1286;gene=cvpA;inference=ab initio prediction:Prodigal:002006,similar to AA sequence:UniProtKB:P08550;locus_tag=M1404_00305;product=Colicin V production protein;protein_id=gnl|Prokka|M1404_00305

gnl|Prokka|M1404_1 prokka gene 331776 333290 . + . ID=M1404_00306_gene;Name=purF;gene=purF;locus_tag=M1404_00306

gnl|Prokka|M1404_1 Prodigal:002006 CDS 331776 333290 . + 0 ID=M1404_00306;Parent=M1404_00306_gene;eC_number=2.4.2.14;Name=purF;db_xref=COG:COG0034;gene=purF;inference=ab initio prediction:Prodigal:002006,similar to AA sequence:UniProtKB:P0AG16;locus_tag=M1404_00306;product=Amidophosphoribosyltransferase;protein_id=gnl|Prokka|M1404_00306

gnl|Prokka|M1404_1 prokka gene 333460 334047 . + . ID=M1404_00307_gene;Name=ubiX;gene=ubiX;locus_tag=M1404_00307

gnl|Prokka|M1404_1 Prodigal:002006 CDS 333460 334047 . + 0 ID=M1404_00307;Parent=M1404_00307_gene;eC_number=2.5.1.129;Name=ubiX;db_xref=COG:COG0163;gene=ubiX;inference=ab initio prediction:Prodigal:002006,similar to AA sequence:UniProtKB:P0AG03;locus_tag=M1404_00307;product=Flavin prenyltransferase UbiX;protein_id=gnl|Prokka|M1404_00307

gnl|Prokka|M1404_1 prokka gene 334064 334786 . - . ID=M1404_00308_gene;Name=moeB;gene=moeB;locus_tag=M1404_00308

gnl|Prokka|M1404_1 Prodigal:002006 CDS 334064 334786 . - 0 ID=M1404_00308;Parent=M1404_00308_gene;eC_number=2.7.7.80;Name=moeB;db_xref=COG:COG0476;gene=moeB;inference=ab initio prediction:Prodigal:002006,similar to AA sequence:UniProtKB:P12282;locus_tag=M1404_00308;product=Molybdopterin-synthase adenylyltransferase;protein_id=gnl|Prokka|M1404_00308

gnl|Prokka|M1404_1 prokka gene 334788 336008 . - . ID=M1404_00309_gene;Name=moeA;gene=moeA;locus_tag=M1404_00309

gnl|Prokka|M1404_1 Prodigal:002006 CDS 334788 336008 . - 0 ID=M1404_00309;Parent=M1404_00309_gene;eC_number=2.10.1.1;Name=moeA;db_xref=COG:COG0303;gene=moeA;inference=ab initio prediction:Prodigal:002006,similar to AA sequence:UniProtKB:P12281;locus_tag=M1404_00309;product=Molybdopterin molybdenumtransferase;protein_id=gnl|Prokka|M1404_00309

gnl|Prokka|M1404_1 prokka gene 336668 337324 . + . ID=M1404_00310_gene;Name=folE;gene=folE;locus_tag=M1404_00310

gnl|Prokka|M1404_1 Prodigal:002006 CDS 336668 337324 . + 0 ID=M1404_00310;Parent=M1404_00310_gene;eC_number=3.5.4.16;Name=folE;db_xref=COG:COG0302;gene=folE;inference=ab initio prediction:Prodigal:002006,similar to AA sequence:UniProtKB:Q8ZG15;locus_tag=M1404_00310;product=GTP cyclohydrolase 1;protein_id=gnl|Prokka|M1404_00310

gnl|Prokka|M1404_1 prokka gene 337419 339380 . - . ID=M1404_00311_gene;Name=acs;gene=acs;locus_tag=M1404_00311

gnl|Prokka|M1404_1 Prodigal:002006 CDS 337419 339380 . - 0 ID=M1404_00311;Parent=M1404_00311_gene;eC_number=6.2.1.1;Name=acs;db_xref=COG:COG0365;gene=acs;inference=ab initio prediction:Prodigal:002006,similar to AA sequence:UniProtKB:Q8ZKF6;locus_tag=M1404_00311;product=Acetyl-coenzyme A synthetase;protein_id=gnl|Prokka|M1404_00311

gnl|Prokka|M1404_1 prokka gene 339589 340431 . + . ID=M1404_00312_gene;Name=xthA;gene=xthA;locus_tag=M1404_00312

gnl|Prokka|M1404_1 Prodigal:002006 CDS 339589 340431 . + 0 ID=M1404_00312;Parent=M1404_00312_gene;eC_number=3.1.11.2;Name=xthA;db_xref=COG:COG0708;gene=xthA;inference=ab initio prediction:Prodigal:002006,similar to AA sequence:UniProtKB:P09030;locus_tag=M1404_00312;product=Exodeoxyribonuclease III;protein_id=gnl|Prokka|M1404_00312

gnl|Prokka|M1404_1 prokka gene 340441 341214 . + . ID=M1404_00313_gene;locus_tag=M1404_00313

gnl|Prokka|M1404_1 Prodigal:002006 CDS 340441 341214 . + 0 ID=M1404_00313;Parent=M1404_00313_gene;inference=ab initio prediction:Prodigal:002006;locus_tag=M1404_00313;product=hypothetical protein;protein_id=gnl|Prokka|M1404_00313

gnl|Prokka|M1404_1 prokka gene 341285 341986 . - . ID=M1404_00314_gene;locus_tag=M1404_00314

gnl|Prokka|M1404_1 Prodigal:002006 CDS 341285 341986 . - 0 ID=M1404_00314;Parent=M1404_00314_gene;inference=ab initio prediction:Prodigal:002006;locus_tag=M1404_00314;product=hypothetical protein;protein_id=gnl|Prokka|M1404_00314

gnl|Prokka|M1404_1 prokka gene 342217 342630 . - . ID=M1404_00315_gene;Name=yhcB;gene=yhcB;locus_tag=M1404_00315

gnl|Prokka|M1404_1 Prodigal:002006 CDS 342217 342630 . - 0 ID=M1404_00315;Parent=M1404_00315_gene;Name=yhcB;db_xref=COG:COG3105;gene=yhcB;inference=ab initio prediction:Prodigal:002006,similar to AA sequence:UniProtKB:P0ADW3;locus_tag=M1404_00315;product=Inner membrane protein YhcB;protein_id=gnl|Prokka|M1404_00315

gnl|Prokka|M1404_1 prokka gene 342751 344511 . - . ID=M1404_00316_gene;Name=ycaO;gene=ycaO;locus_tag=M1404_00316

gnl|Prokka|M1404_1 Prodigal:002006 CDS 342751 344511 . - 0 ID=M1404_00316;Parent=M1404_00316_gene;Name=ycaO;db_xref=COG:COG1944;gene=ycaO;inference=ab initio prediction:Prodigal:002006,similar to AA sequence:UniProtKB:P75838;locus_tag=M1404_00316;product=Ribosomal protein S12 methylthiotransferase accessory factor YcaO;protein_id=gnl|Prokka|M1404_00316

gnl|Prokka|M1404_1 prokka gene 344589 345305 . - . ID=M1404_00317_gene;locus_tag=M1404_00317

gnl|Prokka|M1404_1 Prodigal:002006 CDS 344589 345305 . - 0 ID=M1404_00317;Parent=M1404_00317_gene;inference=ab initio prediction:Prodigal:002006;locus_tag=M1404_00317;product=hypothetical protein;protein_id=gnl|Prokka|M1404_00317

gnl|Prokka|M1404_1 prokka gene 345328 346029 . + . ID=M1404_00318_gene;Name=gloB;gene=gloB;locus_tag=M1404_00318

gnl|Prokka|M1404_1 Prodigal:002006 CDS 345328 346029 . + 0 ID=M1404_00318;Parent=M1404_00318_gene;eC_number=3.1.2.6;Name=gloB;db_xref=COG:COG0491;gene=gloB;inference=ab initio prediction:Prodigal:002006,similar to AA sequence:UniProtKB:Q8ZRM2;locus_tag=M1404_00318;product=Hydroxyacylglutathione hydrolase;protein_id=gnl|Prokka|M1404_00318

gnl|Prokka|M1404_1 prokka gene 346168 347472 . + . ID=M1404_00319_gene;Name=hemA;gene=hemA;locus_tag=M1404_00319

gnl|Prokka|M1404_1 Prodigal:002006 CDS 346168 347472 . + 0 ID=M1404_00319;Parent=M1404_00319_gene;eC_number=1.2.1.70;Name=hemA;db_xref=COG:COG0373;gene=hemA;inference=ab initio prediction:Prodigal:002006,similar to AA sequence:UniProtKB:P0A6X1;locus_tag=M1404_00319;product=Glutamyl-tRNA reductase;protein_id=gnl|Prokka|M1404_00319

gnl|Prokka|M1404_1 prokka gene 347550 348773 . + . ID=M1404_00320_gene;Name=nagC;gene=nagC;locus_tag=M1404_00320

gnl|Prokka|M1404_1 Prodigal:002006 CDS 347550 348773 . + 0 ID=M1404_00320;Parent=M1404_00320_gene;Name=nagC;db_xref=COG:COG1940;gene=nagC;inference=ab initio prediction:Prodigal:002006,similar to AA sequence:UniProtKB:P0AF20;locus_tag=M1404_00320;product=N-acetylglucosamine repressor;protein_id=gnl|Prokka|M1404_00320

gnl|Prokka|M1404_1 prokka gene 348921 348997 . + . ID=M1404_00321_gene;locus_tag=M1404_00321

gnl|Prokka|M1404_1 Aragorn:001002 tRNA 348921 348997 . + . ID=M1404_00321;Parent=M1404_00321_gene;inference=COORDINATES:profile:Aragorn:001002;locus_tag=M1404_00321;product=tRNA-Met(cat)

gnl|Prokka|M1404_1 prokka gene 349003 349087 . + . ID=M1404_00322_gene;locus_tag=M1404_00322

gnl|Prokka|M1404_1 Aragorn:001002 tRNA 349003 349087 . + . ID=M1404_00322;Parent=M1404_00322_gene;inference=COORDINATES:profile:Aragorn:001002;locus_tag=M1404_00322;product=tRNA-Leu(tag)

gnl|Prokka|M1404_1 prokka gene 349117 349191 . + . ID=M1404_00323_gene;locus_tag=M1404_00323

gnl|Prokka|M1404_1 Aragorn:001002 tRNA 349117 349191 . + . ID=M1404_00323;Parent=M1404_00323_gene;inference=COORDINATES:profile:Aragorn:001002;locus_tag=M1404_00323;product=tRNA-Gln(ttg)

gnl|Prokka|M1404_1 prokka gene 349237 349311 . + . ID=M1404_00324_gene;locus_tag=M1404_00324

gnl|Prokka|M1404_1 Aragorn:001002 tRNA 349237 349311 . + . ID=M1404_00324;Parent=M1404_00324_gene;inference=COORDINATES:profile:Aragorn:001002;locus_tag=M1404_00324;product=tRNA-Gln(ttg)

gnl|Prokka|M1404_1 prokka gene 349611 350879 . + . ID=M1404_00325_gene;locus_tag=M1404_00325

gnl|Prokka|M1404_1 Prodigal:002006 CDS 349611 350879 . + 0 ID=M1404_00325;Parent=M1404_00325_gene;inference=ab initio prediction:Prodigal:002006;locus_tag=M1404_00325;product=hypothetical protein;protein_id=gnl|Prokka|M1404_00325

gnl|Prokka|M1404_1 prokka gene 350940 352979 . - . ID=M1404_00326_gene;Name=prlC;gene=prlC;locus_tag=M1404_00326

gnl|Prokka|M1404_1 Prodigal:002006 CDS 350940 352979 . - 0 ID=M1404_00326;Parent=M1404_00326_gene;eC_number=3.4.24.70;Name=prlC;db_xref=COG:COG0339;gene=prlC;inference=ab initio prediction:Prodigal:002006,similar to AA sequence:UniProtKB:P27237;locus_tag=M1404_00326;product=Oligopeptidase A;protein_id=gnl|Prokka|M1404_00326

gnl|Prokka|M1404_1 prokka gene 353101 353463 . + . ID=M1404_00327_gene;Name=ybaN;gene=ybaN;locus_tag=M1404_00327

gnl|Prokka|M1404_1 Prodigal:002006 CDS 353101 353463 . + 0 ID=M1404_00327;Parent=M1404_00327_gene;Name=ybaN;db_xref=COG:COG2832;gene=ybaN;inference=ab initio prediction:Prodigal:002006,similar to AA sequence:UniProtKB:P0AAR5;locus_tag=M1404_00327;product=Inner membrane protein YbaN;protein_id=gnl|Prokka|M1404_00327

gnl|Prokka|M1404_1 prokka gene 353520 355091 . + . ID=M1404_00328_gene;Name=oppA_1;gene=oppA_1;locus_tag=M1404_00328

gnl|Prokka|M1404_1 Prodigal:002006 CDS 353520 355091 . + 0 ID=M1404_00328;Parent=M1404_00328_gene;Name=oppA_1;db_xref=COG:COG4166;gene=oppA_1;inference=ab initio prediction:Prodigal:002006,similar to AA sequence:UniProtKB:P06202;locus_tag=M1404_00328;product=Periplasmic oligopeptide-binding protein;protein_id=gnl|Prokka|M1404_00328

gnl|Prokka|M1404_1 prokka gene 355143 355802 . - . ID=M1404_00329_gene;Name=ribA;gene=ribA;locus_tag=M1404_00329

gnl|Prokka|M1404_1 Prodigal:002006 CDS 355143 355802 . - 0 ID=M1404_00329;Parent=M1404_00329_gene;eC_number=3.5.4.25;Name=ribA;db_xref=COG:COG0807;gene=ribA;inference=ab initio prediction:Prodigal:002006,similar to AA sequence:UniProtKB:P0A7I7;locus_tag=M1404_00329;product=GTP cyclohydrolase-2;protein_id=gnl|Prokka|M1404_00329

gnl|Prokka|M1404_1 prokka gene 355854 356603 . + . ID=M1404_00330_gene;Name=pgpB;gene=pgpB;locus_tag=M1404_00330

gnl|Prokka|M1404_1 Prodigal:002006 CDS 355854 356603 . + 0 ID=M1404_00330;Parent=M1404_00330_gene;eC_number=3.1.3.27;Name=pgpB;db_xref=COG:COG0671;gene=pgpB;inference=ab initio prediction:Prodigal:002006,similar to AA sequence:UniProtKB:P0A924;locus_tag=M1404_00330;product=Phosphatidylglycerophosphatase B;protein_id=gnl|Prokka|M1404_00330

gnl|Prokka|M1404_1 prokka gene 356653 357567 . + . ID=M1404_00331_gene;Name=nagK;gene=nagK;locus_tag=M1404_00331

gnl|Prokka|M1404_1 Prodigal:002006 CDS 356653 357567 . + 0 ID=M1404_00331;Parent=M1404_00331_gene;eC_number=2.7.1.59;Name=nagK;db_xref=COG:COG1940;gene=nagK;inference=ab initio prediction:Prodigal:002006,similar to AA sequence:UniProtKB:Q8D9M7;locus_tag=M1404_00331;product=N-acetyl-D-glucosamine kinase;protein_id=gnl|Prokka|M1404_00331

gnl|Prokka|M1404_1 prokka gene 357721 358413 . + . ID=M1404_00332_gene;locus_tag=M1404_00332

gnl|Prokka|M1404_1 Prodigal:002006 CDS 357721 358413 . + 0 ID=M1404_00332;Parent=M1404_00332_gene;inference=ab initio prediction:Prodigal:002006;locus_tag=M1404_00332;product=hypothetical protein;protein_id=gnl|Prokka|M1404_00332

gnl|Prokka|M1404_1 prokka gene 358489 359229 . + . ID=M1404_00333_gene;locus_tag=M1404_00333

gnl|Prokka|M1404_1 Prodigal:002006 CDS 358489 359229 . + 0 ID=M1404_00333;Parent=M1404_00333_gene;inference=ab initio prediction:Prodigal:002006;locus_tag=M1404_00333;product=hypothetical protein;protein_id=gnl|Prokka|M1404_00333

gnl|Prokka|M1404_1 prokka gene 359231 359365 . + . ID=M1404_00334_gene;locus_tag=M1404_00334

gnl|Prokka|M1404_1 Prodigal:002006 CDS 359231 359365 . + 0 ID=M1404_00334;Parent=M1404_00334_gene;inference=ab initio prediction:Prodigal:002006;locus_tag=M1404_00334;product=hypothetical protein;protein_id=gnl|Prokka|M1404_00334

gnl|Prokka|M1404_1 prokka gene 359458 360012 . - . ID=M1404_00335_gene;Name=ydjA;gene=ydjA;locus_tag=M1404_00335

gnl|Prokka|M1404_1 Prodigal:002006 CDS 359458 360012 . - 0 ID=M1404_00335;Parent=M1404_00335_gene;eC_number=1.-.-.-;Name=ydjA;db_xref=COG:COG0778;gene=ydjA;inference=ab initio prediction:Prodigal:002006,similar to AA sequence:UniProtKB:P0ACY1;locus_tag=M1404_00335;product=Putative NAD(P)H nitroreductase YdjA;protein_id=gnl|Prokka|M1404_00335

gnl|Prokka|M1404_1 prokka gene 360136 362019 . + . ID=M1404_00336_gene;Name=sppA;gene=sppA;locus_tag=M1404_00336

gnl|Prokka|M1404_1 Prodigal:002006 CDS 360136 362019 . + 0 ID=M1404_00336;Parent=M1404_00336_gene;eC_number=3.4.21.-;Name=sppA;db_xref=COG:COG0616;gene=sppA;inference=ab initio prediction:Prodigal:002006,similar to AA sequence:UniProtKB:P45243;locus_tag=M1404_00336;product=Protease 4;protein_id=gnl|Prokka|M1404_00336

gnl|Prokka|M1404_1 prokka gene 362098 362472 . + . ID=M1404_00337_gene;locus_tag=M1404_00337

gnl|Prokka|M1404_1 Prodigal:002006 CDS 362098 362472 . + 0 ID=M1404_00337;Parent=M1404_00337_gene;inference=ab initio prediction:Prodigal:002006;locus_tag=M1404_00337;product=hypothetical protein;protein_id=gnl|Prokka|M1404_00337

gnl|Prokka|M1404_1 prokka gene 362535 363473 . - . ID=M1404_00338_gene;Name=uspE;gene=uspE;locus_tag=M1404_00338

gnl|Prokka|M1404_1 Prodigal:002006 CDS 362535 363473 . - 0 ID=M1404_00338;Parent=M1404_00338_gene;Name=uspE;db_xref=COG:COG0589;gene=uspE;inference=ab initio prediction:Prodigal:002006,similar to AA sequence:UniProtKB:P44195;locus_tag=M1404_00338;product=Universal stress protein E ;protein_id=gnl|Prokka|M1404_00338

gnl|Prokka|M1404_1 prokka gene 363585 364343 . - . ID=M1404_00339_gene;Name=fnr;gene=fnr;locus_tag=M1404_00339

gnl|Prokka|M1404_1 Prodigal:002006 CDS 363585 364343 . - 0 ID=M1404_00339;Parent=M1404_00339_gene;Name=fnr;db_xref=COG:COG0664;gene=fnr;inference=ab initio prediction:Prodigal:002006,similar to AA sequence:UniProtKB:Q9EXQ1;locus_tag=M1404_00339;product=Anaerobic regulatory protein;protein_id=gnl|Prokka|M1404_00339

gnl|Prokka|M1404_1 prokka gene 364454 364555 . + . ID=M1404_00340_gene;locus_tag=M1404_00340

gnl|Prokka|M1404_1 Prodigal:002006 CDS 364454 364555 . + 0 ID=M1404_00340;Parent=M1404_00340_gene;inference=ab initio prediction:Prodigal:002006;locus_tag=M1404_00340;product=hypothetical protein;protein_id=gnl|Prokka|M1404_00340

gnl|Prokka|M1404_1 prokka gene 364629 365126 . - . ID=M1404_00341_gene;Name=ftnA_1;gene=ftnA_1;locus_tag=M1404_00341

gnl|Prokka|M1404_1 Prodigal:002006 CDS 364629 365126 . - 0 ID=M1404_00341;Parent=M1404_00341_gene;eC_number=1.16.3.2;Name=ftnA_1;db_xref=COG:COG1528;gene=ftnA_1;inference=ab initio prediction:Prodigal:002006,similar to AA sequence:UniProtKB:P43707;locus_tag=M1404_00341;product=putative bacterial non-heme ferritin;protein_id=gnl|Prokka|M1404_00341

gnl|Prokka|M1404_1 prokka gene 365142 365627 . - . ID=M1404_00342_gene;Name=ftnA_2;gene=ftnA_2;locus_tag=M1404_00342

gnl|Prokka|M1404_1 Prodigal:002006 CDS 365142 365627 . - 0 ID=M1404_00342;Parent=M1404_00342_gene;eC_number=1.16.3.2;Name=ftnA_2;db_xref=COG:COG1528;gene=ftnA_2;inference=ab initio prediction:Prodigal:002006,similar to AA sequence:UniProtKB:P43707;locus_tag=M1404_00342;product=putative bacterial non-heme ferritin;protein_id=gnl|Prokka|M1404_00342

gnl|Prokka|M1404_1 prokka gene 366012 367064 . + . ID=M1404_00343_gene;Name=aroF;gene=aroF;locus_tag=M1404_00343

gnl|Prokka|M1404_1 Prodigal:002006 CDS 366012 367064 . + 0 ID=M1404_00343;Parent=M1404_00343_gene;eC_number=2.5.1.54;Name=aroF;db_xref=COG:COG0722;gene=aroF;inference=ab initio prediction:Prodigal:002006,similar to AA sequence:UniProtKB:P00888;locus_tag=M1404_00343;product=Phospho-2-dehydro-3-deoxyheptonate aldolase%2C Tyr-sensitive;protein_id=gnl|Prokka|M1404_00343

gnl|Prokka|M1404_1 prokka gene 367074 368198 . + . ID=M1404_00344_gene;Name=tyrA;gene=tyrA;locus_tag=M1404_00344

gnl|Prokka|M1404_1 Prodigal:002006 CDS 367074 368198 . + 0 ID=M1404_00344;Parent=M1404_00344_gene;Name=tyrA;db_xref=COG:COG0287;gene=tyrA;inference=ab initio prediction:Prodigal:002006,similar to AA sequence:UniProtKB:P43902;locus_tag=M1404_00344;product=T-protein;protein_id=gnl|Prokka|M1404_00344

gnl|Prokka|M1404_1 prokka gene 368540 370945 . + . ID=M1404_00345_gene;locus_tag=M1404_00345

gnl|Prokka|M1404_1 Prodigal:002006 CDS 368540 370945 . + 0 ID=M1404_00345;Parent=M1404_00345_gene;inference=ab initio prediction:Prodigal:002006;locus_tag=M1404_00345;product=hypothetical protein;protein_id=gnl|Prokka|M1404_00345

gnl|Prokka|M1404_1 prokka gene 371261 371884 . + . ID=M1404_00346_gene;Name=yciO;gene=yciO;locus_tag=M1404_00346

gnl|Prokka|M1404_1 Prodigal:002006 CDS 371261 371884 . + 0 ID=M1404_00346;Parent=M1404_00346_gene;Name=yciO;db_xref=COG:COG0009;gene=yciO;inference=ab initio prediction:Prodigal:002006,similar to AA sequence:UniProtKB:P0AFR4;locus_tag=M1404_00346;product=putative protein YciO;protein_id=gnl|Prokka|M1404_00346

gnl|Prokka|M1404_1 prokka gene 371933 372970 . + . ID=M1404_00347_gene;Name=rluB;gene=rluB;locus_tag=M1404_00347

gnl|Prokka|M1404_1 Prodigal:002006 CDS 371933 372970 . + 0 ID=M1404_00347;Parent=M1404_00347_gene;eC_number=5.4.99.22;Name=rluB;db_xref=COG:COG1187;gene=rluB;inference=ab initio prediction:Prodigal:002006,similar to AA sequence:UniProtKB:P45104;locus_tag=M1404_00347;product=Ribosomal large subunit pseudouridine synthase B;protein_id=gnl|Prokka|M1404_00347

gnl|Prokka|M1404_1 prokka gene 373011 373982 . + . ID=M1404_00348_gene;Name=cysB;gene=cysB;locus_tag=M1404_00348

gnl|Prokka|M1404_1 Prodigal:002006 CDS 373011 373982 . + 0 ID=M1404_00348;Parent=M1404_00348_gene;Name=cysB;gene=cysB;inference=ab initio prediction:Prodigal:002006,similar to AA sequence:UniProtKB:P06614;locus_tag=M1404_00348;product=HTH-type transcriptional regulator CysB;protein_id=gnl|Prokka|M1404_00348

gnl|Prokka|M1404_1 prokka gene 374063 379780 . - . ID=M1404_00349_gene;Name=yfhM;gene=yfhM;locus_tag=M1404_00349

gnl|Prokka|M1404_1 Prodigal:002006 CDS 374063 379780 . - 0 ID=M1404_00349;Parent=M1404_00349_gene;Name=yfhM;db_xref=COG:COG2373;gene=yfhM;inference=ab initio prediction:Prodigal:002006,similar to AA sequence:UniProtKB:P76578;locus_tag=M1404_00349;product=Alpha-2-macroglobulin;protein_id=gnl|Prokka|M1404_00349

gnl|Prokka|M1404_1 prokka gene 379963 380787 . + . ID=M1404_00350_gene;Name=dapD;gene=dapD;locus_tag=M1404_00350

gnl|Prokka|M1404_1 Prodigal:002006 CDS 379963 380787 . + 0 ID=M1404_00350;Parent=M1404_00350_gene;eC_number=2.3.1.117;Name=dapD;db_xref=COG:COG2171;gene=dapD;inference=ab initio prediction:Prodigal:002006,similar to AA sequence:UniProtKB:P0A9D8;locus_tag=M1404_00350;product=2%2C3%2C4%2C5-tetrahydropyridine-2%2C6-dicarboxylate N-succinyltransferase;protein_id=gnl|Prokka|M1404_00350

gnl|Prokka|M1404_1 prokka gene 380947 382029 . + . ID=M1404_00351_gene;locus_tag=M1404_00351

gnl|Prokka|M1404_1 Prodigal:002006 CDS 380947 382029 . + 0 ID=M1404_00351;Parent=M1404_00351_gene;inference=ab initio prediction:Prodigal:002006;locus_tag=M1404_00351;product=hypothetical protein;protein_id=gnl|Prokka|M1404_00351

gnl|Prokka|M1404_1 prokka gene 382067 382927 . - . ID=M1404_00352_gene;Name=tehB;gene=tehB;locus_tag=M1404_00352

gnl|Prokka|M1404_1 Prodigal:002006 CDS 382067 382927 . - 0 ID=M1404_00352;Parent=M1404_00352_gene;eC_number=2.1.1.-;Name=tehB;gene=tehB;inference=ab initio prediction:Prodigal:002006,similar to AA sequence:UniProtKB:E1X791;locus_tag=M1404_00352;product=putative S-adenosyl-L-methionine-dependent methyltransferase TehB;protein_id=gnl|Prokka|M1404_00352

gnl|Prokka|M1404_1 prokka gene 383275 383484 . + . ID=M1404_00353_gene;Name=cspC;gene=cspC;locus_tag=M1404_00353

gnl|Prokka|M1404_1 Prodigal:002006 CDS 383275 383484 . + 0 ID=M1404_00353;Parent=M1404_00353_gene;Name=cspC;gene=cspC;inference=ab initio prediction:Prodigal:002006,similar to AA sequence:UniProtKB:E0J500;locus_tag=M1404_00353;product=Cold shock-like protein CspC;protein_id=gnl|Prokka|M1404_00353

gnl|Prokka|M1404_1 prokka gene 384203 384697 . + . ID=M1404_00354_gene;locus_tag=M1404_00354

gnl|Prokka|M1404_1 Prodigal:002006 CDS 384203 384697 . + 0 ID=M1404_00354;Parent=M1404_00354_gene;inference=ab initio prediction:Prodigal:002006;locus_tag=M1404_00354;product=hypothetical protein;protein_id=gnl|Prokka|M1404_00354

gnl|Prokka|M1404_1 prokka gene 384850 386061 . - . ID=M1404_00355_gene;Name=intA_1;gene=intA_1;locus_tag=M1404_00355

gnl|Prokka|M1404_1 Prodigal:002006 CDS 384850 386061 . - 0 ID=M1404_00355;Parent=M1404_00355_gene;Name=intA_1;db_xref=COG:COG0582;gene=intA_1;inference=ab initio prediction:Prodigal:002006,similar to AA sequence:UniProtKB:P32053;locus_tag=M1404_00355;product=Prophage integrase IntA;protein_id=gnl|Prokka|M1404_00355

gnl|Prokka|M1404_1 prokka gene 386253 386329 . - . ID=M1404_00356_gene;locus_tag=M1404_00356

gnl|Prokka|M1404_1 Aragorn:001002 tRNA 386253 386329 . - . ID=M1404_00356;Parent=M1404_00356_gene;inference=COORDINATES:profile:Aragorn:001002;locus_tag=M1404_00356;product=tRNA-Val(gac)

gnl|Prokka|M1404_1 prokka gene 386576 388015 . + . ID=M1404_00357_gene;Name=pykA;gene=pykA;locus_tag=M1404_00357

gnl|Prokka|M1404_1 Prodigal:002006 CDS 386576 388015 . + 0 ID=M1404_00357;Parent=M1404_00357_gene;eC_number=2.7.1.40;Name=pykA;db_xref=COG:COG0469;gene=pykA;inference=ab initio prediction:Prodigal:002006,similar to AA sequence:UniProtKB:P21599;locus_tag=M1404_00357;product=Pyruvate kinase II;protein_id=gnl|Prokka|M1404_00357

gnl|Prokka|M1404_1 prokka gene 388145 389197 . - . ID=M1404_00358_gene;Name=ydgJ;gene=ydgJ;locus_tag=M1404_00358

gnl|Prokka|M1404_1 Prodigal:002006 CDS 388145 389197 . - 0 ID=M1404_00358;Parent=M1404_00358_gene;eC_number=1.-.-.-;Name=ydgJ;db_xref=COG:COG0673;gene=ydgJ;inference=ab initio prediction:Prodigal:002006,similar to AA sequence:UniProtKB:P77376;locus_tag=M1404_00358;product=putative oxidoreductase YdgJ;protein_id=gnl|Prokka|M1404_00358

gnl|Prokka|M1404_1 prokka gene 389197 390591 . - . ID=M1404_00359_gene;Name=mdtK;gene=mdtK;locus_tag=M1404_00359

gnl|Prokka|M1404_1 Prodigal:002006 CDS 389197 390591 . - 0 ID=M1404_00359;Parent=M1404_00359_gene;Name=mdtK;db_xref=COG:COG0534;gene=mdtK;inference=ab initio prediction:Prodigal:002006,similar to AA sequence:UniProtKB:P37340;locus_tag=M1404_00359;product=Multidrug resistance protein MdtK;protein_id=gnl|Prokka|M1404_00359

gnl|Prokka|M1404_1 prokka gene 390647 391261 . + . ID=M1404_00360_gene;Name=ribC;gene=ribC;locus_tag=M1404_00360

gnl|Prokka|M1404_1 Prodigal:002006 CDS 390647 391261 . + 0 ID=M1404_00360;Parent=M1404_00360_gene;eC_number=2.5.1.9;Name=ribC;db_xref=COG:COG0307;gene=ribC;inference=ab initio prediction:Prodigal:002006,similar to AA sequence:UniProtKB:P0AFU8;locus_tag=M1404_00360;product=Riboflavin synthase;protein_id=gnl|Prokka|M1404_00360

gnl|Prokka|M1404_1 prokka gene 391322 391906 . - . ID=M1404_00361_gene;locus_tag=M1404_00361

gnl|Prokka|M1404_1 Prodigal:002006 CDS 391322 391906 . - 0 ID=M1404_00361;Parent=M1404_00361_gene;inference=ab initio prediction:Prodigal:002006;locus_tag=M1404_00361;product=hypothetical protein;protein_id=gnl|Prokka|M1404_00361

gnl|Prokka|M1404_1 prokka gene 391970 392557 . - . ID=M1404_00362_gene;Name=gmhA_1;gene=gmhA_1;locus_tag=M1404_00362

gnl|Prokka|M1404_1 Prodigal:002006 CDS 391970 392557 . - 0 ID=M1404_00362;Parent=M1404_00362_gene;eC_number=5.3.1.28;Name=gmhA_1;db_xref=COG:COG0279;gene=gmhA_1;inference=ab initio prediction:Prodigal:002006,similar to AA sequence:UniProtKB:Q47VU0;locus_tag=M1404_00362;product=Phosphoheptose isomerase;protein_id=gnl|Prokka|M1404_00362

gnl|Prokka|M1404_1 prokka gene 392560 392919 . - . ID=M1404_00363_gene;locus_tag=M1404_00363

gnl|Prokka|M1404_1 Prodigal:002006 CDS 392560 392919 . - 0 ID=M1404_00363;Parent=M1404_00363_gene;inference=ab initio prediction:Prodigal:002006;locus_tag=M1404_00363;product=hypothetical protein;protein_id=gnl|Prokka|M1404_00363

gnl|Prokka|M1404_1 prokka gene 392921 394633 . - . ID=M1404_00364_gene;Name=lpoA;gene=lpoA;locus_tag=M1404_00364

gnl|Prokka|M1404_1 Prodigal:002006 CDS 392921 394633 . - 0 ID=M1404_00364;Parent=M1404_00364_gene;Name=lpoA;db_xref=COG:COG3107;gene=lpoA;inference=ab initio prediction:Prodigal:002006,similar to AA sequence:UniProtKB:P45299;locus_tag=M1404_00364;product=Penicillin-binding protein activator LpoA;protein_id=gnl|Prokka|M1404_00364

gnl|Prokka|M1404_1 prokka gene 394711 395556 . + . ID=M1404_00365_gene;Name=rsmI;gene=rsmI;locus_tag=M1404_00365

gnl|Prokka|M1404_1 Prodigal:002006 CDS 394711 395556 . + 0 ID=M1404_00365;Parent=M1404_00365_gene;eC_number=2.1.1.198;Name=rsmI;db_xref=COG:COG0313;gene=rsmI;inference=ab initio prediction:Prodigal:002006,similar to AA sequence:UniProtKB:P67087;locus_tag=M1404_00365;product=Ribosomal RNA small subunit methyltransferase I;protein_id=gnl|Prokka|M1404_00365

gnl|Prokka|M1404_1 prokka gene 395602 397959 . - . ID=M1404_00366_gene;Name=pbpC;gene=pbpC;locus_tag=M1404_00366

gnl|Prokka|M1404_1 Prodigal:002006 CDS 395602 397959 . - 0 ID=M1404_00366;Parent=M1404_00366_gene;Name=pbpC;db_xref=COG:COG4953;gene=pbpC;inference=ab initio prediction:Prodigal:002006,similar to AA sequence:UniProtKB:P76577;locus_tag=M1404_00366;product=Penicillin-binding protein 1C;protein_id=gnl|Prokka|M1404_00366

gnl|Prokka|M1404_1 prokka gene 398020 399423 . - . ID=M1404_00367_gene;Name=asnS;gene=asnS;locus_tag=M1404_00367

gnl|Prokka|M1404_1 Prodigal:002006 CDS 398020 399423 . - 0 ID=M1404_00367;Parent=M1404_00367_gene;eC_number=6.1.1.22;Name=asnS;db_xref=COG:COG0017;gene=asnS;inference=ab initio prediction:Prodigal:002006,similar to AA sequence:UniProtKB:P0A8M0;locus_tag=M1404_00367;product=Asparagine--tRNA ligase;protein_id=gnl|Prokka|M1404_00367

gnl|Prokka|M1404_1 prokka gene 399718 400929 . + . ID=M1404_00368_gene;Name=mlc;gene=mlc;locus_tag=M1404_00368

gnl|Prokka|M1404_1 Prodigal:002006 CDS 399718 400929 . + 0 ID=M1404_00368;Parent=M1404_00368_gene;Name=mlc;db_xref=COG:COG1940;gene=mlc;inference=ab initio prediction:Prodigal:002006,similar to AA sequence:UniProtKB:P50456;locus_tag=M1404_00368;product=Protein mlc;protein_id=gnl|Prokka|M1404_00368

gnl|Prokka|M1404_1 prokka gene 401096 401830 . + . ID=M1404_00369_gene;Name=bioD1_1;gene=bioD1_1;locus_tag=M1404_00369

gnl|Prokka|M1404_1 Prodigal:002006 CDS 401096 401830 . + 0 ID=M1404_00369;Parent=M1404_00369_gene;eC_number=6.3.3.3;Name=bioD1_1;db_xref=COG:COG0132;gene=bioD1_1;inference=ab initio prediction:Prodigal:002006,similar to AA sequence:UniProtKB:P13000;locus_tag=M1404_00369;product=ATP-dependent dethiobiotin synthetase BioD 1;protein_id=gnl|Prokka|M1404_00369

gnl|Prokka|M1404_1 prokka gene 401899 402513 . - . ID=M1404_00370_gene;locus_tag=M1404_00370

gnl|Prokka|M1404_1 Prodigal:002006 CDS 401899 402513 . - 0 ID=M1404_00370;Parent=M1404_00370_gene;inference=ab initio prediction:Prodigal:002006;locus_tag=M1404_00370;product=hypothetical protein;protein_id=gnl|Prokka|M1404_00370

gnl|Prokka|M1404_1 prokka gene 402731 403015 . + . ID=M1404_00371_gene;Name=rplY;gene=rplY;locus_tag=M1404_00371

gnl|Prokka|M1404_1 Prodigal:002006 CDS 402731 403015 . + 0 ID=M1404_00371;Parent=M1404_00371_gene;Name=rplY;db_xref=COG:COG1825;gene=rplY;inference=ab initio prediction:Prodigal:002006,similar to AA sequence:UniProtKB:P68919;locus_tag=M1404_00371;product=50S ribosomal protein L25;protein_id=gnl|Prokka|M1404_00371

gnl|Prokka|M1404_1 prokka gene 403084 403926 . - . ID=M1404_00372_gene;locus_tag=M1404_00372

gnl|Prokka|M1404_1 Prodigal:002006 CDS 403084 403926 . - 0 ID=M1404_00372;Parent=M1404_00372_gene;inference=ab initio prediction:Prodigal:002006;locus_tag=M1404_00372;product=hypothetical protein;protein_id=gnl|Prokka|M1404_00372

gnl|Prokka|M1404_1 prokka gene 404084 404878 . + . ID=M1404_00373_gene;Name=truA;gene=truA;locus_tag=M1404_00373

gnl|Prokka|M1404_1 Prodigal:002006 CDS 404084 404878 . + 0 ID=M1404_00373;Parent=M1404_00373_gene;eC_number=5.4.99.12;Name=truA;db_xref=COG:COG0101;gene=truA;inference=ab initio prediction:Prodigal:002006,similar to AA sequence:UniProtKB:P07649;locus_tag=M1404_00373;product=tRNA pseudouridine synthase A;protein_id=gnl|Prokka|M1404_00373

gnl|Prokka|M1404_1 prokka gene 404962 405876 . + . ID=M1404_00374_gene;Name=accD;gene=accD;locus_tag=M1404_00374

gnl|Prokka|M1404_1 Prodigal:002006 CDS 404962 405876 . + 0 ID=M1404_00374;Parent=M1404_00374_gene;eC_number=2.1.3.15;Name=accD;db_xref=COG:COG0777;gene=accD;inference=ab initio prediction:Prodigal:002006,similar to AA sequence:UniProtKB:P0A9Q5;locus_tag=M1404_00374;product=Acetyl-coenzyme A carboxylase carboxyl transferase subunit beta;protein_id=gnl|Prokka|M1404_00374

gnl|Prokka|M1404_1 prokka gene 405869 407197 . + . ID=M1404_00375_gene;Name=folC;gene=folC;locus_tag=M1404_00375

gnl|Prokka|M1404_1 Prodigal:002006 CDS 405869 407197 . + 0 ID=M1404_00375;Parent=M1404_00375_gene;eC_number=6.3.2.12;Name=folC;db_xref=COG:COG0285;gene=folC;inference=ab initio prediction:Prodigal:002006,similar to AA sequence:UniProtKB:P08192;locus_tag=M1404_00375;product=Dihydrofolate synthase/folylpolyglutamate synthase;protein_id=gnl|Prokka|M1404_00375

gnl|Prokka|M1404_1 prokka gene 407235 407885 . - . ID=M1404_00376_gene;Name=pspA;gene=pspA;locus_tag=M1404_00376

gnl|Prokka|M1404_1 Prodigal:002006 CDS 407235 407885 . - 0 ID=M1404_00376;Parent=M1404_00376_gene;eC_number=3.1.3.3;Name=pspA;db_xref=COG:COG0406;gene=pspA;inference=ab initio prediction:Prodigal:002006,similar to AA sequence:UniProtKB:D3DFG8;locus_tag=M1404_00376;product=Phosphoserine phosphatase 1;protein_id=gnl|Prokka|M1404_00376

gnl|Prokka|M1404_1 prokka gene 407993 408655 . + . ID=M1404_00377_gene;Name=rluA_1;gene=rluA_1;locus_tag=M1404_00377

gnl|Prokka|M1404_1 Prodigal:002006 CDS 407993 408655 . + 0 ID=M1404_00377;Parent=M1404_00377_gene;eC_number=5.4.99.28;Name=rluA_1;db_xref=COG:COG0564;gene=rluA_1;inference=ab initio prediction:Prodigal:002006,similar to AA sequence:UniProtKB:P0AA37;locus_tag=M1404_00377;product=Dual-specificity RNA pseudouridine synthase RluA;protein_id=gnl|Prokka|M1404_00377

gnl|Prokka|M1404_1 prokka gene 408684 409202 . + . ID=M1404_00378_gene;locus_tag=M1404_00378

gnl|Prokka|M1404_1 Prodigal:002006 CDS 408684 409202 . + 0 ID=M1404_00378;Parent=M1404_00378_gene;inference=ab initio prediction:Prodigal:002006;locus_tag=M1404_00378;product=hypothetical protein;protein_id=gnl|Prokka|M1404_00378

gnl|Prokka|M1404_1 prokka gene 409381 410364 . + . ID=M1404_00379_gene;Name=pheS;gene=pheS;locus_tag=M1404_00379

gnl|Prokka|M1404_1 Prodigal:002006 CDS 409381 410364 . + 0 ID=M1404_00379;Parent=M1404_00379_gene;eC_number=6.1.1.20;Name=pheS;db_xref=COG:COG0016;gene=pheS;inference=ab initio prediction:Prodigal:002006,similar to AA sequence:UniProtKB:P08312;locus_tag=M1404_00379;product=Phenylalanine--tRNA ligase alpha subunit;protein_id=gnl|Prokka|M1404_00379

gnl|Prokka|M1404_1 prokka gene 410375 410854 . + . ID=M1404_00380_gene;locus_tag=M1404_00380

gnl|Prokka|M1404_1 Prodigal:002006 CDS 410375 410854 . + 0 ID=M1404_00380;Parent=M1404_00380_gene;inference=ab initio prediction:Prodigal:002006;locus_tag=M1404_00380;product=hypothetical protein;protein_id=gnl|Prokka|M1404_00380

gnl|Prokka|M1404_1 prokka gene 410877 413264 . + . ID=M1404_00381_gene;Name=pheT;gene=pheT;locus_tag=M1404_00381

gnl|Prokka|M1404_1 Prodigal:002006 CDS 410877 413264 . + 0 ID=M1404_00381;Parent=M1404_00381_gene;eC_number=6.1.1.20;Name=pheT;db_xref=COG:COG0072;gene=pheT;inference=ab initio prediction:Prodigal:002006,similar to AA sequence:UniProtKB:P07395;locus_tag=M1404_00381;product=Phenylalanine--tRNA ligase beta subunit;protein_id=gnl|Prokka|M1404_00381

gnl|Prokka|M1404_1 prokka gene 413268 413564 . + . ID=M1404_00382_gene;Name=ihfA;gene=ihfA;locus_tag=M1404_00382

gnl|Prokka|M1404_1 Prodigal:002006 CDS 413268 413564 . + 0 ID=M1404_00382;Parent=M1404_00382_gene;Name=ihfA;db_xref=COG:COG0776;gene=ihfA;inference=ab initio prediction:Prodigal:002006,similar to AA sequence:UniProtKB:P0A6X7;locus_tag=M1404_00382;product=Integration host factor subunit alpha;protein_id=gnl|Prokka|M1404_00382

gnl|Prokka|M1404_1 prokka gene 413617 414132 . + . ID=M1404_00383_gene;Name=mepS;gene=mepS;locus_tag=M1404_00383

gnl|Prokka|M1404_1 Prodigal:002006 CDS 413617 414132 . + 0 ID=M1404_00383;Parent=M1404_00383_gene;eC_number=3.4.-.-;Name=mepS;db_xref=COG:COG0791;gene=mepS;inference=ab initio prediction:Prodigal:002006,similar to AA sequence:UniProtKB:P0AFV4;locus_tag=M1404_00383;product=Murein DD-endopeptidase MepS/Murein LD-carboxypeptidase;protein_id=gnl|Prokka|M1404_00383

gnl|Prokka|M1404_1 prokka gene 414152 415078 . - . ID=M1404_00384_gene;Name=ybhK;gene=ybhK;locus_tag=M1404_00384

gnl|Prokka|M1404_1 Prodigal:002006 CDS 414152 415078 . - 0 ID=M1404_00384;Parent=M1404_00384_gene;Name=ybhK;db_xref=COG:COG0391;gene=ybhK;inference=ab initio prediction:Prodigal:002006,similar to AA sequence:UniProtKB:P75767;locus_tag=M1404_00384;product=Putative gluconeogenesis factor;protein_id=gnl|Prokka|M1404_00384

gnl|Prokka|M1404_1 prokka gene 415451 416464 . + . ID=M1404_00385_gene;Name=moaA;gene=moaA;locus_tag=M1404_00385

gnl|Prokka|M1404_1 Prodigal:002006 CDS 415451 416464 . + 0 ID=M1404_00385;Parent=M1404_00385_gene;eC_number=4.1.99.22;Name=moaA;db_xref=COG:COG2896;gene=moaA;inference=ab initio prediction:Prodigal:002006,similar to AA sequence:UniProtKB:P30745;locus_tag=M1404_00385;product=GTP 3'%2C8-cyclase;protein_id=gnl|Prokka|M1404_00385

gnl|Prokka|M1404_1 prokka gene 416524 417000 . + . ID=M1404_00386_gene;Name=moaC;gene=moaC;locus_tag=M1404_00386

gnl|Prokka|M1404_1 Prodigal:002006 CDS 416524 417000 . + 0 ID=M1404_00386;Parent=M1404_00386_gene;eC_number=4.6.1.17;Name=moaC;db_xref=COG:COG0315;gene=moaC;inference=ab initio prediction:Prodigal:002006,similar to AA sequence:UniProtKB:P0A738;locus_tag=M1404_00386;product=Cyclic pyranopterin monophosphate synthase;protein_id=gnl|Prokka|M1404_00386

gnl|Prokka|M1404_1 prokka gene 417013 417258 . + . ID=M1404_00387_gene;Name=moaD;gene=moaD;locus_tag=M1404_00387

gnl|Prokka|M1404_1 Prodigal:002006 CDS 417013 417258 . + 0 ID=M1404_00387;Parent=M1404_00387_gene;Name=moaD;db_xref=COG:COG1977;gene=moaD;inference=ab initio prediction:Prodigal:002006,similar to AA sequence:UniProtKB:P30748;locus_tag=M1404_00387;product=Molybdopterin synthase sulfur carrier subunit;protein_id=gnl|Prokka|M1404_00387

gnl|Prokka|M1404_1 prokka gene 417259 417711 . + . ID=M1404_00388_gene;Name=moaE;gene=moaE;locus_tag=M1404_00388

gnl|Prokka|M1404_1 Prodigal:002006 CDS 417259 417711 . + 0 ID=M1404_00388;Parent=M1404_00388_gene;eC_number=2.8.1.12;Name=moaE;db_xref=COG:COG0314;gene=moaE;inference=ab initio prediction:Prodigal:002006,similar to AA sequence:UniProtKB:P30749;locus_tag=M1404_00388;product=Molybdopterin synthase catalytic subunit;protein_id=gnl|Prokka|M1404_00388

gnl|Prokka|M1404_1 prokka gene 417773 418963 . - . ID=M1404_00389_gene;Name=aspC;gene=aspC;locus_tag=M1404_00389

gnl|Prokka|M1404_1 Prodigal:002006 CDS 417773 418963 . - 0 ID=M1404_00389;Parent=M1404_00389_gene;eC_number=2.6.1.1;Name=aspC;db_xref=COG:COG1448;gene=aspC;inference=ab initio prediction:Prodigal:002006,similar to AA sequence:UniProtKB:P00509;locus_tag=M1404_00389;product=Aspartate aminotransferase;protein_id=gnl|Prokka|M1404_00389

gnl|Prokka|M1404_1 prokka gene 419106 420191 . - . ID=M1404_00390_gene;Name=purK;gene=purK;locus_tag=M1404_00390

gnl|Prokka|M1404_1 Prodigal:002006 CDS 419106 420191 . - 0 ID=M1404_00390;Parent=M1404_00390_gene;eC_number=6.3.4.18;Name=purK;db_xref=COG:COG0026;gene=purK;inference=ab initio prediction:Prodigal:002006,similar to AA sequence:UniProtKB:P09029;locus_tag=M1404_00390;product=N5-carboxyaminoimidazole ribonucleotide synthase;protein_id=gnl|Prokka|M1404_00390

gnl|Prokka|M1404_1 prokka gene 420255 420755 . - . ID=M1404_00391_gene;Name=purE;gene=purE;locus_tag=M1404_00391

gnl|Prokka|M1404_1 Prodigal:002006 CDS 420255 420755 . - 0 ID=M1404_00391;Parent=M1404_00391_gene;eC_number=5.4.99.18;Name=purE;db_xref=COG:COG0041;gene=purE;inference=ab initio prediction:Prodigal:002006,similar to AA sequence:UniProtKB:P0AG18;locus_tag=M1404_00391;product=N5-carboxyaminoimidazole ribonucleotide mutase;protein_id=gnl|Prokka|M1404_00391

gnl|Prokka|M1404_1 prokka gene 420927 423536 . + . ID=M1404_00392_gene;Name=pepN;gene=pepN;locus_tag=M1404_00392

gnl|Prokka|M1404_1 Prodigal:002006 CDS 420927 423536 . + 0 ID=M1404_00392;Parent=M1404_00392_gene;eC_number=3.4.11.2;Name=pepN;db_xref=COG:COG0308;gene=pepN;inference=ab initio prediction:Prodigal:002006,similar to AA sequence:UniProtKB:P04825;locus_tag=M1404_00392;product=Aminopeptidase N;protein_id=gnl|Prokka|M1404_00392

gnl|Prokka|M1404_1 prokka gene 423689 424708 . + . ID=M1404_00393_gene;Name=pyrD;gene=pyrD;locus_tag=M1404_00393

gnl|Prokka|M1404_1 Prodigal:002006 CDS 423689 424708 . + 0 ID=M1404_00393;Parent=M1404_00393_gene;eC_number=1.3.5.2;Name=pyrD;db_xref=COG:COG0167;gene=pyrD;inference=ab initio prediction:Prodigal:002006,similar to AA sequence:UniProtKB:P0A7E1;locus_tag=M1404_00393;product=Dihydroorotate dehydrogenase (quinone);protein_id=gnl|Prokka|M1404_00393

gnl|Prokka|M1404_1 prokka gene 424795 425628 . + . ID=M1404_00394_gene;Name=yciV;gene=yciV;locus_tag=M1404_00394

gnl|Prokka|M1404_1 Prodigal:002006 CDS 424795 425628 . + 0 ID=M1404_00394;Parent=M1404_00394_gene;eC_number=3.1.13.-;Name=yciV;db_xref=COG:COG0613;gene=yciV;inference=ab initio prediction:Prodigal:002006,similar to AA sequence:UniProtKB:P77766;locus_tag=M1404_00394;product=5'-3' exoribonuclease;protein_id=gnl|Prokka|M1404_00394

gnl|Prokka|M1404_1 prokka gene 425789 428395 . + . ID=M1404_00395_gene;Name=topA_1;gene=topA_1;locus_tag=M1404_00395

gnl|Prokka|M1404_1 Prodigal:002006 CDS 425789 428395 . + 0 ID=M1404_00395;Parent=M1404_00395_gene;eC_number=5.6.2.1;Name=topA_1;db_xref=COG:COG0550;gene=topA_1;inference=ab initio prediction:Prodigal:002006,similar to AA sequence:UniProtKB:P06612;locus_tag=M1404_00395;product=DNA topoisomerase 1;protein_id=gnl|Prokka|M1404_00395

gnl|Prokka|M1404_1 prokka gene 428461 428739 . - . ID=M1404_00396_gene;locus_tag=M1404_00396

gnl|Prokka|M1404_1 Prodigal:002006 CDS 428461 428739 . - 0 ID=M1404_00396;Parent=M1404_00396_gene;inference=ab initio prediction:Prodigal:002006;locus_tag=M1404_00396;product=hypothetical protein;protein_id=gnl|Prokka|M1404_00396

gnl|Prokka|M1404_1 prokka gene 428752 429144 . - . ID=M1404_00397_gene;locus_tag=M1404_00397

gnl|Prokka|M1404_1 Prodigal:002006 CDS 428752 429144 . - 0 ID=M1404_00397;Parent=M1404_00397_gene;inference=ab initio prediction:Prodigal:002006;locus_tag=M1404_00397;product=hypothetical protein;protein_id=gnl|Prokka|M1404_00397

gnl|Prokka|M1404_1 prokka gene 429203 429514 . - . ID=M1404_00398_gene;Name=yqjD;gene=yqjD;locus_tag=M1404_00398

gnl|Prokka|M1404_1 Prodigal:002006 CDS 429203 429514 . - 0 ID=M1404_00398;Parent=M1404_00398_gene;Name=yqjD;db_xref=COG:COG4575;gene=yqjD;inference=ab initio prediction:Prodigal:002006,similar to AA sequence:UniProtKB:P64581;locus_tag=M1404_00398;product=putative protein YqjD;protein_id=gnl|Prokka|M1404_00398

gnl|Prokka|M1404_1 prokka gene 430382 431809 . - . ID=M1404_00399_gene;Name=sbcB;gene=sbcB;locus_tag=M1404_00399

gnl|Prokka|M1404_1 Prodigal:002006 CDS 430382 431809 . - 0 ID=M1404_00399;Parent=M1404_00399_gene;eC_number=3.1.11.1;Name=sbcB;db_xref=COG:COG2925;gene=sbcB;inference=ab initio prediction:Prodigal:002006,similar to AA sequence:UniProtKB:P04995;locus_tag=M1404_00399;product=Exodeoxyribonuclease I;protein_id=gnl|Prokka|M1404_00399

gnl|Prokka|M1404_1 prokka gene 431911 432777 . - . ID=M1404_00400_gene;locus_tag=M1404_00400

gnl|Prokka|M1404_1 Prodigal:002006 CDS 431911 432777 . - 0 ID=M1404_00400;Parent=M1404_00400_gene;inference=ab initio prediction:Prodigal:002006;locus_tag=M1404_00400;product=hypothetical protein;protein_id=gnl|Prokka|M1404_00400

gnl|Prokka|M1404_1 prokka gene 432946 437433 . - . ID=M1404_00401_gene;Name=mukB;gene=mukB;locus_tag=M1404_00401

gnl|Prokka|M1404_1 Prodigal:002006 CDS 432946 437433 . - 0 ID=M1404_00401;Parent=M1404_00401_gene;Name=mukB;db_xref=COG:COG3096;gene=mukB;inference=ab initio prediction:Prodigal:002006,similar to AA sequence:UniProtKB:Q7VL96;locus_tag=M1404_00401;product=Chromosome partition protein MukB;protein_id=gnl|Prokka|M1404_00401

gnl|Prokka|M1404_1 prokka gene 437433 438164 . - . ID=M1404_00402_gene;Name=mukE;gene=mukE;locus_tag=M1404_00402

gnl|Prokka|M1404_1 Prodigal:002006 CDS 437433 438164 . - 0 ID=M1404_00402;Parent=M1404_00402_gene;Name=mukE;db_xref=COG:COG3095;gene=mukE;inference=ab initio prediction:Prodigal:002006,similar to AA sequence:UniProtKB:Q7VL95;locus_tag=M1404_00402;product=Chromosome partition protein MukE;protein_id=gnl|Prokka|M1404_00402

gnl|Prokka|M1404_1 prokka gene 438215 439540 . - . ID=M1404_00403_gene;Name=mukF;gene=mukF;locus_tag=M1404_00403

gnl|Prokka|M1404_1 Prodigal:002006 CDS 438215 439540 . - 0 ID=M1404_00403;Parent=M1404_00403_gene;Name=mukF;db_xref=COG:COG3006;gene=mukF;inference=ab initio prediction:Prodigal:002006,similar to AA sequence:UniProtKB:Q7VL94;locus_tag=M1404_00403;product=Chromosome partition protein MukF;protein_id=gnl|Prokka|M1404_00403

gnl|Prokka|M1404_1 prokka gene 439724 440662 . + . ID=M1404_00404_gene;Name=ttcA;gene=ttcA;locus_tag=M1404_00404

gnl|Prokka|M1404_1 Prodigal:002006 CDS 439724 440662 . + 0 ID=M1404_00404;Parent=M1404_00404_gene;eC_number=2.8.1.-;Name=ttcA;db_xref=COG:COG0037;gene=ttcA;inference=ab initio prediction:Prodigal:002006,similar to AA sequence:UniProtKB:P76055;locus_tag=M1404_00404;product=tRNA-cytidine(32) 2-sulfurtransferase;protein_id=gnl|Prokka|M1404_00404

gnl|Prokka|M1404_1 prokka gene 440697 441221 . + . ID=M1404_00405_gene;Name=msrA;gene=msrA;locus_tag=M1404_00405

gnl|Prokka|M1404_1 Prodigal:002006 CDS 440697 441221 . + 0 ID=M1404_00405;Parent=M1404_00405_gene;eC_number=1.8.4.11;Name=msrA;db_xref=COG:COG0225;gene=msrA;inference=ab initio prediction:Prodigal:002006,similar to AA sequence:UniProtKB:P47648;locus_tag=M1404_00405;product=Peptide methionine sulfoxide reductase MsrA;protein_id=gnl|Prokka|M1404_00405

gnl|Prokka|M1404_1 prokka gene 442489 442842 . - . ID=M1404_00406_gene;Name=rplT;gene=rplT;locus_tag=M1404_00406

gnl|Prokka|M1404_1 Prodigal:002006 CDS 442489 442842 . - 0 ID=M1404_00406;Parent=M1404_00406_gene;Name=rplT;db_xref=COG:COG0292;gene=rplT;inference=ab initio prediction:Prodigal:002006,similar to AA sequence:UniProtKB:P0A7L3;locus_tag=M1404_00406;product=50S ribosomal protein L20;protein_id=gnl|Prokka|M1404_00406

gnl|Prokka|M1404_1 prokka gene 442913 443110 . - . ID=M1404_00407_gene;Name=rpmI;gene=rpmI;locus_tag=M1404_00407

gnl|Prokka|M1404_1 Prodigal:002006 CDS 442913 443110 . - 0 ID=M1404_00407;Parent=M1404_00407_gene;Name=rpmI;db_xref=COG:COG0291;gene=rpmI;inference=ab initio prediction:Prodigal:002006,similar to AA sequence:UniProtKB:P0A7Q1;locus_tag=M1404_00407;product=50S ribosomal protein L35;protein_id=gnl|Prokka|M1404_00407

gnl|Prokka|M1404_1 prokka gene 443508 443972 . - . ID=M1404_00408_gene;Name=infC;gene=infC;locus_tag=M1404_00408

gnl|Prokka|M1404_1 Prodigal:002006 CDS 443508 443972 . - 0 ID=M1404_00408;Parent=M1404_00408_gene;Name=infC;db_xref=COG:COG0290;gene=infC;inference=ab initio prediction:Prodigal:002006,similar to AA sequence:UniProtKB:P0A707;locus_tag=M1404_00408;product=Translation initiation factor IF-3;protein_id=gnl|Prokka|M1404_00408

gnl|Prokka|M1404_1 prokka gene 444173 444997 . - . ID=M1404_00409_gene;Name=yeaD_2;gene=yeaD_2;locus_tag=M1404_00409

gnl|Prokka|M1404_1 Prodigal:002006 CDS 444173 444997 . - 0 ID=M1404_00409;Parent=M1404_00409_gene;eC_number=5.1.3.15;Name=yeaD_2;db_xref=COG:COG0676;gene=yeaD_2;inference=ab initio prediction:Prodigal:002006,similar to AA sequence:UniProtKB:P39173;locus_tag=M1404_00409;product=Putative glucose-6-phosphate 1-epimerase;protein_id=gnl|Prokka|M1404_00409

gnl|Prokka|M1404_1 prokka gene 445019 446152 . - . ID=M1404_00410_gene;Name=chuR_1;gene=chuR_1;locus_tag=M1404_00410

gnl|Prokka|M1404_1 Prodigal:002006 CDS 445019 446152 . - 0 ID=M1404_00410;Parent=M1404_00410_gene;eC_number=1.1.99.-;Name=chuR_1;db_xref=COG:COG0641;gene=chuR_1;inference=ab initio prediction:Prodigal:002006,similar to AA sequence:UniProtKB:Q02550;locus_tag=M1404_00410;product=Anaerobic sulfatase-maturating enzyme;protein_id=gnl|Prokka|M1404_00410

gnl|Prokka|M1404_1 prokka gene 446165 446758 . - . ID=M1404_00411_gene;locus_tag=M1404_00411

gnl|Prokka|M1404_1 Prodigal:002006 CDS 446165 446758 . - 0 ID=M1404_00411;Parent=M1404_00411_gene;inference=ab initio prediction:Prodigal:002006,similar to AA sequence:UniProtKB:Q53W63;locus_tag=M1404_00411;product=Transcriptional regulator LdrP;protein_id=gnl|Prokka|M1404_00411

gnl|Prokka|M1404_1 prokka gene 446909 448354 . - . ID=M1404_00412_gene;locus_tag=M1404_00412

gnl|Prokka|M1404_1 Prodigal:002006 CDS 446909 448354 . - 0 ID=M1404_00412;Parent=M1404_00412_gene;eC_number=3.1.4.-;inference=ab initio prediction:Prodigal:002006,similar to AA sequence:UniProtKB:Q1M964;locus_tag=M1404_00412;product=Multifunctional alkaline phosphatase superfamily protein;protein_id=gnl|Prokka|M1404_00412

gnl|Prokka|M1404_1 prokka gene 448358 450037 . - . ID=M1404_00413_gene;Name=yidK;gene=yidK;locus_tag=M1404_00413

gnl|Prokka|M1404_1 Prodigal:002006 CDS 448358 450037 . - 0 ID=M1404_00413;Parent=M1404_00413_gene;Name=yidK;db_xref=COG:COG4146;gene=yidK;inference=ab initio prediction:Prodigal:002006,similar to AA sequence:UniProtKB:P31448;locus_tag=M1404_00413;product=putative symporter YidK;protein_id=gnl|Prokka|M1404_00413

gnl|Prokka|M1404_1 prokka gene 450268 451068 . + . ID=M1404_00414_gene;locus_tag=M1404_00414

gnl|Prokka|M1404_1 Prodigal:002006 CDS 450268 451068 . + 0 ID=M1404_00414;Parent=M1404_00414_gene;inference=ab initio prediction:Prodigal:002006;locus_tag=M1404_00414;product=hypothetical protein;protein_id=gnl|Prokka|M1404_00414

gnl|Prokka|M1404_1 prokka gene 451377 453308 . - . ID=M1404_00415_gene;Name=thrS;gene=thrS;locus_tag=M1404_00415

gnl|Prokka|M1404_1 Prodigal:002006 CDS 451377 453308 . - 0 ID=M1404_00415;Parent=M1404_00415_gene;eC_number=6.1.1.3;Name=thrS;db_xref=COG:COG0441;gene=thrS;inference=ab initio prediction:Prodigal:002006,similar to AA sequence:UniProtKB:P0A8M3;locus_tag=M1404_00415;product=Threonine--tRNA ligase;protein_id=gnl|Prokka|M1404_00415

gnl|Prokka|M1404_1 prokka gene 453643 455238 . + . ID=M1404_00416_gene;Name=hbpA;gene=hbpA;locus_tag=M1404_00416

gnl|Prokka|M1404_1 Prodigal:002006 CDS 453643 455238 . + 0 ID=M1404_00416;Parent=M1404_00416_gene;Name=hbpA;db_xref=COG:COG0747;gene=hbpA;inference=ab initio prediction:Prodigal:002006,similar to AA sequence:UniProtKB:P33950;locus_tag=M1404_00416;product=Heme-binding protein A;protein_id=gnl|Prokka|M1404_00416

gnl|Prokka|M1404_1 prokka gene 455307 456677 . - . ID=M1404_00417_gene;locus_tag=M1404_00417

gnl|Prokka|M1404_1 Prodigal:002006 CDS 455307 456677 . - 0 ID=M1404_00417;Parent=M1404_00417_gene;inference=ab initio prediction:Prodigal:002006;locus_tag=M1404_00417;product=hypothetical protein;protein_id=gnl|Prokka|M1404_00417

gnl|Prokka|M1404_1 prokka gene 456750 457169 . - . ID=M1404_00418_gene;locus_tag=M1404_00418

gnl|Prokka|M1404_1 Prodigal:002006 CDS 456750 457169 . - 0 ID=M1404_00418;Parent=M1404_00418_gene;inference=ab initio prediction:Prodigal:002006;locus_tag=M1404_00418;product=hypothetical protein;protein_id=gnl|Prokka|M1404_00418

gnl|Prokka|M1404_1 prokka gene 457287 457883 . - . ID=M1404_00419_gene;locus_tag=M1404_00419

gnl|Prokka|M1404_1 Prodigal:002006 CDS 457287 457883 . - 0 ID=M1404_00419;Parent=M1404_00419_gene;inference=ab initio prediction:Prodigal:002006;locus_tag=M1404_00419;product=hypothetical protein;protein_id=gnl|Prokka|M1404_00419

gnl|Prokka|M1404_1 prokka gene 457978 461574 . - . ID=M1404_00420_gene;Name=putA;gene=putA;locus_tag=M1404_00420

gnl|Prokka|M1404_1 Prodigal:002006 CDS 457978 461574 . - 0 ID=M1404_00420;Parent=M1404_00420_gene;Name=putA;gene=putA;inference=ab initio prediction:Prodigal:002006,similar to AA sequence:UniProtKB:P10503;locus_tag=M1404_00420;product=Bifunctional protein PutA;protein_id=gnl|Prokka|M1404_00420

gnl|Prokka|M1404_1 prokka gene 461647 463149 . - . ID=M1404_00421_gene;Name=putP_1;gene=putP_1;locus_tag=M1404_00421

gnl|Prokka|M1404_1 Prodigal:002006 CDS 461647 463149 . - 0 ID=M1404_00421;Parent=M1404_00421_gene;Name=putP_1;db_xref=COG:COG0591;gene=putP_1;inference=ab initio prediction:Prodigal:002006,similar to AA sequence:UniProtKB:P07117;locus_tag=M1404_00421;product=Sodium/proline symporter;protein_id=gnl|Prokka|M1404_00421

gnl|Prokka|M1404_1 prokka gene 463405 464271 . + . ID=M1404_00422_gene;locus_tag=M1404_00422

gnl|Prokka|M1404_1 Prodigal:002006 CDS 463405 464271 . + 0 ID=M1404_00422;Parent=M1404_00422_gene;eC_number=2.7.1.-;inference=ab initio prediction:Prodigal:002006,similar to AA sequence:UniProtKB:Q3XZZ9;locus_tag=M1404_00422;product=putative ketoamine kinase;protein_id=gnl|Prokka|M1404_00422

gnl|Prokka|M1404_1 prokka gene 464325 465143 . - . ID=M1404_00423_gene;Name=ompA_2;gene=ompA_2;locus_tag=M1404_00423

gnl|Prokka|M1404_1 Prodigal:002006 CDS 464325 465143 . - 0 ID=M1404_00423;Parent=M1404_00423_gene;Name=ompA_2;gene=ompA_2;inference=ab initio prediction:Prodigal:002006,protein motif:HAMAP:MF_00842;locus_tag=M1404_00423;product=Outer membrane protein A;protein_id=gnl|Prokka|M1404_00423

gnl|Prokka|M1404_1 prokka gene 465384 466445 . - . ID=M1404_00424_gene;Name=sohB;gene=sohB;locus_tag=M1404_00424

gnl|Prokka|M1404_1 Prodigal:002006 CDS 465384 466445 . - 0 ID=M1404_00424;Parent=M1404_00424_gene;eC_number=3.4.21.-;Name=sohB;db_xref=COG:COG0616;gene=sohB;inference=ab initio prediction:Prodigal:002006,similar to AA sequence:UniProtKB:P0AG14;locus_tag=M1404_00424;product=putative protease SohB;protein_id=gnl|Prokka|M1404_00424

gnl|Prokka|M1404_1 prokka gene 466691 468241 . + . ID=M1404_00425_gene;Name=trpE;gene=trpE;locus_tag=M1404_00425

gnl|Prokka|M1404_1 Prodigal:002006 CDS 466691 468241 . + 0 ID=M1404_00425;Parent=M1404_00425_gene;eC_number=4.1.3.27;Name=trpE;db_xref=COG:COG0147;gene=trpE;inference=ab initio prediction:Prodigal:002006,similar to AA sequence:UniProtKB:P00895;locus_tag=M1404_00425;product=Anthranilate synthase component 1;protein_id=gnl|Prokka|M1404_00425

gnl|Prokka|M1404_1 prokka gene 468252 468839 . + . ID=M1404_00426_gene;Name=trpG;gene=trpG;locus_tag=M1404_00426

gnl|Prokka|M1404_1 Prodigal:002006 CDS 468252 468839 . + 0 ID=M1404_00426;Parent=M1404_00426_gene;eC_number=4.1.3.27;Name=trpG;gene=trpG;inference=ab initio prediction:Prodigal:002006,similar to AA sequence:UniProtKB:P00900;locus_tag=M1404_00426;product=Anthranilate synthase component 2;protein_id=gnl|Prokka|M1404_00426

gnl|Prokka|M1404_1 prokka gene 468856 469740 . - . ID=M1404_00427_gene;locus_tag=M1404_00427

gnl|Prokka|M1404_1 Prodigal:002006 CDS 468856 469740 . - 0 ID=M1404_00427;Parent=M1404_00427_gene;inference=ab initio prediction:Prodigal:002006;locus_tag=M1404_00427;product=hypothetical protein;protein_id=gnl|Prokka|M1404_00427

gnl|Prokka|M1404_1 prokka gene 469908 470909 . + . ID=M1404_00428_gene;Name=trpGD;gene=trpGD;locus_tag=M1404_00428

gnl|Prokka|M1404_1 Prodigal:002006 CDS 469908 470909 . + 0 ID=M1404_00428;Parent=M1404_00428_gene;Name=trpGD;db_xref=COG:COG0512;gene=trpGD;inference=ab initio prediction:Prodigal:002006,similar to AA sequence:UniProtKB:P00904;locus_tag=M1404_00428;product=Bifunctional protein TrpGD;protein_id=gnl|Prokka|M1404_00428

gnl|Prokka|M1404_1 prokka gene 470946 472355 . + . ID=M1404_00429_gene;Name=trpC;gene=trpC;locus_tag=M1404_00429

gnl|Prokka|M1404_1 Prodigal:002006 CDS 470946 472355 . + 0 ID=M1404_00429;Parent=M1404_00429_gene;Name=trpC;db_xref=COG:COG0134;gene=trpC;inference=ab initio prediction:Prodigal:002006,similar to AA sequence:UniProtKB:P00909;locus_tag=M1404_00429;product=Tryptophan biosynthesis protein TrpCF;protein_id=gnl|Prokka|M1404_00429

gnl|Prokka|M1404_1 prokka gene 472391 473149 . + . ID=M1404_00430_gene;Name=ydfG;gene=ydfG;locus_tag=M1404_00430

gnl|Prokka|M1404_1 Prodigal:002006 CDS 472391 473149 . + 0 ID=M1404_00430;Parent=M1404_00430_gene;eC_number=1.1.1.381;Name=ydfG;db_xref=COG:COG4221;gene=ydfG;inference=ab initio prediction:Prodigal:002006,similar to AA sequence:UniProtKB:P39831;locus_tag=M1404_00430;product=NADP-dependent 3-hydroxy acid dehydrogenase YdfG;protein_id=gnl|Prokka|M1404_00430

gnl|Prokka|M1404_1 prokka gene 473352 474560 . + . ID=M1404_00431_gene;Name=trpB;gene=trpB;locus_tag=M1404_00431

gnl|Prokka|M1404_1 Prodigal:002006 CDS 473352 474560 . + 0 ID=M1404_00431;Parent=M1404_00431_gene;eC_number=4.2.1.20;Name=trpB;db_xref=COG:COG0133;gene=trpB;inference=ab initio prediction:Prodigal:002006,similar to AA sequence:UniProtKB:P0A2K1;locus_tag=M1404_00431;product=Tryptophan synthase beta chain;protein_id=gnl|Prokka|M1404_00431

gnl|Prokka|M1404_1 prokka gene 474547 475353 . + . ID=M1404_00432_gene;Name=trpA;gene=trpA;locus_tag=M1404_00432

gnl|Prokka|M1404_1 Prodigal:002006 CDS 474547 475353 . + 0 ID=M1404_00432;Parent=M1404_00432_gene;eC_number=4.2.1.20;Name=trpA;db_xref=COG:COG0159;gene=trpA;inference=ab initio prediction:Prodigal:002006,similar to AA sequence:UniProtKB:Q9KST7;locus_tag=M1404_00432;product=Tryptophan synthase alpha chain;protein_id=gnl|Prokka|M1404_00432

gnl|Prokka|M1404_1 prokka gene 475392 477026 . - . ID=M1404_00433_gene;Name=tdhA_1;gene=tdhA_1;locus_tag=M1404_00433

gnl|Prokka|M1404_1 Prodigal:002006 CDS 475392 477026 . - 0 ID=M1404_00433;Parent=M1404_00433_gene;Name=tdhA_1;db_xref=COG:COG1629;gene=tdhA_1;inference=ab initio prediction:Prodigal:002006,similar to AA sequence:UniProtKB:Q7VNU1;locus_tag=M1404_00433;product=TonB-dependent heme receptor A;protein_id=gnl|Prokka|M1404_00433

gnl|Prokka|M1404_1 prokka gene 477106 477582 . - . ID=M1404_00434_gene;Name=tdhA_2;gene=tdhA_2;locus_tag=M1404_00434

gnl|Prokka|M1404_1 Prodigal:002006 CDS 477106 477582 . - 0 ID=M1404_00434;Parent=M1404_00434_gene;Name=tdhA_2;db_xref=COG:COG1629;gene=tdhA_2;inference=ab initio prediction:Prodigal:002006,similar to AA sequence:UniProtKB:Q7VNU1;locus_tag=M1404_00434;product=TonB-dependent heme receptor A;protein_id=gnl|Prokka|M1404_00434

gnl|Prokka|M1404_1 prokka gene 477748 478437 . + . ID=M1404_00435_gene;Name=can;gene=can;locus_tag=M1404_00435

gnl|Prokka|M1404_1 Prodigal:002006 CDS 477748 478437 . + 0 ID=M1404_00435;Parent=M1404_00435_gene;eC_number=4.2.1.1;Name=can;db_xref=COG:COG0288;gene=can;inference=ab initio prediction:Prodigal:002006,similar to AA sequence:UniProtKB:P45148;locus_tag=M1404_00435;product=Carbonic anhydrase 2;protein_id=gnl|Prokka|M1404_00435

gnl|Prokka|M1404_1 prokka gene 478569 479426 . + . ID=M1404_00436_gene;locus_tag=M1404_00436

gnl|Prokka|M1404_1 Prodigal:002006 CDS 478569 479426 . + 0 ID=M1404_00436;Parent=M1404_00436_gene;inference=ab initio prediction:Prodigal:002006;locus_tag=M1404_00436;product=hypothetical protein;protein_id=gnl|Prokka|M1404_00436

gnl|Prokka|M1404_1 prokka gene 479508 480461 . + . ID=M1404_00437_gene;Name=trxB;gene=trxB;locus_tag=M1404_00437

gnl|Prokka|M1404_1 Prodigal:002006 CDS 479508 480461 . + 0 ID=M1404_00437;Parent=M1404_00437_gene;eC_number=1.8.1.9;Name=trxB;db_xref=COG:COG0492;gene=trxB;inference=ab initio prediction:Prodigal:002006,similar to AA sequence:UniProtKB:P43788;locus_tag=M1404_00437;product=Thioredoxin reductase;protein_id=gnl|Prokka|M1404_00437

gnl|Prokka|M1404_1 prokka gene 480547 482310 . + . ID=M1404_00438_gene;Name=cydD_1;gene=cydD_1;locus_tag=M1404_00438

gnl|Prokka|M1404_1 Prodigal:002006 CDS 480547 482310 . + 0 ID=M1404_00438;Parent=M1404_00438_gene;Name=cydD_1;db_xref=COG:COG4988;gene=cydD_1;inference=ab initio prediction:Prodigal:002006,similar to AA sequence:UniProtKB:P29018;locus_tag=M1404_00438;product=ATP-binding/permease protein CydD;protein_id=gnl|Prokka|M1404_00438

gnl|Prokka|M1404_1 prokka gene 482310 484040 . + . ID=M1404_00439_gene;Name=msbA_2;gene=msbA_2;locus_tag=M1404_00439

gnl|Prokka|M1404_1 Prodigal:002006 CDS 482310 484040 . + 0 ID=M1404_00439;Parent=M1404_00439_gene;eC_number=7.5.2.6;Name=msbA_2;db_xref=COG:COG1132;gene=msbA_2;inference=ab initio prediction:Prodigal:002006,similar to AA sequence:UniProtKB:P63359;locus_tag=M1404_00439;product=Lipid A export ATP-binding/permease protein MsbA;protein_id=gnl|Prokka|M1404_00439

gnl|Prokka|M1404_1 prokka gene 484355 485233 . + . ID=M1404_00440_gene;Name=tesB;gene=tesB;locus_tag=M1404_00440

gnl|Prokka|M1404_1 Prodigal:002006 CDS 484355 485233 . + 0 ID=M1404_00440;Parent=M1404_00440_gene;eC_number=3.1.2.-;Name=tesB;db_xref=COG:COG1946;gene=tesB;inference=ab initio prediction:Prodigal:002006,similar to AA sequence:UniProtKB:P0AGG2;locus_tag=M1404_00440;product=Acyl-CoA thioesterase 2;protein_id=gnl|Prokka|M1404_00440

gnl|Prokka|M1404_1 prokka gene 485230 486093 . + . ID=M1404_00441_gene;locus_tag=M1404_00441

gnl|Prokka|M1404_1 Prodigal:002006 CDS 485230 486093 . + 0 ID=M1404_00441;Parent=M1404_00441_gene;inference=ab initio prediction:Prodigal:002006;locus_tag=M1404_00441;product=hypothetical protein;protein_id=gnl|Prokka|M1404_00441

gnl|Prokka|M1404_1 prokka gene 486176 487273 . - . ID=M1404_00442_gene;Name=rlmM;gene=rlmM;locus_tag=M1404_00442

gnl|Prokka|M1404_1 Prodigal:002006 CDS 486176 487273 . - 0 ID=M1404_00442;Parent=M1404_00442_gene;eC_number=2.1.1.186;Name=rlmM;db_xref=COG:COG2933;gene=rlmM;inference=ab initio prediction:Prodigal:002006,similar to AA sequence:UniProtKB:P0ADR6;locus_tag=M1404_00442;product=Ribosomal RNA large subunit methyltransferase M;protein_id=gnl|Prokka|M1404_00442

gnl|Prokka|M1404_1 prokka gene 487273 488154 . - . ID=M1404_00443_gene;Name=gcvA;gene=gcvA;locus_tag=M1404_00443

gnl|Prokka|M1404_1 Prodigal:002006 CDS 487273 488154 . - 0 ID=M1404_00443;Parent=M1404_00443_gene;Name=gcvA;gene=gcvA;inference=ab initio prediction:Prodigal:002006,similar to AA sequence:UniProtKB:P0A9F6;locus_tag=M1404_00443;product=Glycine cleavage system transcriptional activator;protein_id=gnl|Prokka|M1404_00443

gnl|Prokka|M1404_1 prokka gene 488626 489648 . + . ID=M1404_00444_gene;Name=ilvE;gene=ilvE;locus_tag=M1404_00444

gnl|Prokka|M1404_1 Prodigal:002006 CDS 488626 489648 . + 0 ID=M1404_00444;Parent=M1404_00444_gene;eC_number=2.6.1.42;Name=ilvE;db_xref=COG:COG0115;gene=ilvE;inference=ab initio prediction:Prodigal:002006,similar to AA sequence:UniProtKB:P9WQ75;locus_tag=M1404_00444;product=Branched-chain-amino-acid aminotransferase;protein_id=gnl|Prokka|M1404_00444

gnl|Prokka|M1404_1 prokka gene 489740 491002 . - . ID=M1404_00445_gene;Name=codA;gene=codA;locus_tag=M1404_00445

gnl|Prokka|M1404_1 Prodigal:002006 CDS 489740 491002 . - 0 ID=M1404_00445;Parent=M1404_00445_gene;eC_number=3.5.4.1;Name=codA;db_xref=COG:COG0402;gene=codA;inference=ab initio prediction:Prodigal:002006,similar to AA sequence:UniProtKB:P25524;locus_tag=M1404_00445;product=Cytosine deaminase;protein_id=gnl|Prokka|M1404_00445

gnl|Prokka|M1404_1 prokka gene 491012 492238 . - . ID=M1404_00446_gene;Name=codB;gene=codB;locus_tag=M1404_00446

gnl|Prokka|M1404_1 Prodigal:002006 CDS 491012 492238 . - 0 ID=M1404_00446;Parent=M1404_00446_gene;Name=codB;db_xref=COG:COG1457;gene=codB;inference=ab initio prediction:Prodigal:002006,similar to AA sequence:UniProtKB:P0AA82;locus_tag=M1404_00446;product=Cytosine permease;protein_id=gnl|Prokka|M1404_00446

gnl|Prokka|M1404_1 prokka gene 492460 493545 . - . ID=M1404_00447_gene;Name=aroG;gene=aroG;locus_tag=M1404_00447

gnl|Prokka|M1404_1 Prodigal:002006 CDS 492460 493545 . - 0 ID=M1404_00447;Parent=M1404_00447_gene;eC_number=2.5.1.54;Name=aroG;db_xref=COG:COG0722;gene=aroG;inference=ab initio prediction:Prodigal:002006,similar to AA sequence:UniProtKB:P0AB91;locus_tag=M1404_00447;product=Phospho-2-dehydro-3-deoxyheptonate aldolase%2C Phe-sensitive;protein_id=gnl|Prokka|M1404_00447

gnl|Prokka|M1404_1 prokka gene 493691 494944 . - . ID=M1404_00448_gene;Name=lolE;gene=lolE;locus_tag=M1404_00448

gnl|Prokka|M1404_1 Prodigal:002006 CDS 493691 494944 . - 0 ID=M1404_00448;Parent=M1404_00448_gene;Name=lolE;db_xref=COG:COG4591;gene=lolE;inference=ab initio prediction:Prodigal:002006,similar to AA sequence:UniProtKB:P75958;locus_tag=M1404_00448;product=Lipoprotein-releasing system transmembrane protein LolE;protein_id=gnl|Prokka|M1404_00448

gnl|Prokka|M1404_1 prokka gene 494945 495628 . - . ID=M1404_00449_gene;Name=lolD_1;gene=lolD_1;locus_tag=M1404_00449

gnl|Prokka|M1404_1 Prodigal:002006 CDS 494945 495628 . - 0 ID=M1404_00449;Parent=M1404_00449_gene;eC_number=3.6.3.-;Name=lolD_1;db_xref=COG:COG1136;gene=lolD_1;inference=ab initio prediction:Prodigal:002006,similar to AA sequence:UniProtKB:P75957;locus_tag=M1404_00449;product=Lipoprotein-releasing system ATP-binding protein LolD;protein_id=gnl|Prokka|M1404_00449

gnl|Prokka|M1404_1 prokka gene 495645 496835 . - . ID=M1404_00450_gene;Name=lolC;gene=lolC;locus_tag=M1404_00450

gnl|Prokka|M1404_1 Prodigal:002006 CDS 495645 496835 . - 0 ID=M1404_00450;Parent=M1404_00450_gene;Name=lolC;db_xref=COG:COG4591;gene=lolC;inference=ab initio prediction:Prodigal:002006,similar to AA sequence:UniProtKB:P0ADC3;locus_tag=M1404_00450;product=Lipoprotein-releasing system transmembrane protein LolC;protein_id=gnl|Prokka|M1404_00450

gnl|Prokka|M1404_1 prokka gene 496917 497867 . - . ID=M1404_00451_gene;locus_tag=M1404_00451

gnl|Prokka|M1404_1 Prodigal:002006 CDS 496917 497867 . - 0 ID=M1404_00451;Parent=M1404_00451_gene;eC_number=1.-.-.-;db_xref=COG:COG1052;inference=ab initio prediction:Prodigal:002006,similar to AA sequence:UniProtKB:P45250;locus_tag=M1404_00451;product=Putative 2-hydroxyacid dehydrogenase;protein_id=gnl|Prokka|M1404_00451

gnl|Prokka|M1404_1 prokka gene 498050 498904 . - . ID=M1404_00452_gene;Name=kdsA;gene=kdsA;locus_tag=M1404_00452

gnl|Prokka|M1404_1 Prodigal:002006 CDS 498050 498904 . - 0 ID=M1404_00452;Parent=M1404_00452_gene;eC_number=2.5.1.55;Name=kdsA;db_xref=COG:COG2877;gene=kdsA;inference=ab initio prediction:Prodigal:002006,similar to AA sequence:UniProtKB:P45251;locus_tag=M1404_00452;product=2-dehydro-3-deoxyphosphooctonate aldolase;protein_id=gnl|Prokka|M1404_00452

gnl|Prokka|M1404_1 prokka gene 498918 499712 . - . ID=M1404_00453_gene;locus_tag=M1404_00453

gnl|Prokka|M1404_1 Prodigal:002006 CDS 498918 499712 . - 0 ID=M1404_00453;Parent=M1404_00453_gene;inference=ab initio prediction:Prodigal:002006;locus_tag=M1404_00453;product=hypothetical protein;protein_id=gnl|Prokka|M1404_00453

gnl|Prokka|M1404_1 prokka gene 499718 500614 . - . ID=M1404_00454_gene;Name=prmC;gene=prmC;locus_tag=M1404_00454

gnl|Prokka|M1404_1 Prodigal:002006 CDS 499718 500614 . - 0 ID=M1404_00454;Parent=M1404_00454_gene;eC_number=2.1.1.297;Name=prmC;db_xref=COG:COG2890;gene=prmC;inference=ab initio prediction:Prodigal:002006,similar to AA sequence:UniProtKB:P0ACC1;locus_tag=M1404_00454;product=Release factor glutamine methyltransferase;protein_id=gnl|Prokka|M1404_00454

gnl|Prokka|M1404_1 prokka gene 500673 501755 . - . ID=M1404_00455_gene;Name=prfA;gene=prfA;locus_tag=M1404_00455

gnl|Prokka|M1404_1 Prodigal:002006 CDS 500673 501755 . - 0 ID=M1404_00455;Parent=M1404_00455_gene;Name=prfA;db_xref=COG:COG0216;gene=prfA;inference=ab initio prediction:Prodigal:002006,similar to AA sequence:UniProtKB:P0A7I0;locus_tag=M1404_00455;product=Peptide chain release factor RF1;protein_id=gnl|Prokka|M1404_00455

gnl|Prokka|M1404_1 prokka gene 501920 502384 . - . ID=M1404_00456_gene;Name=pcp;gene=pcp;locus_tag=M1404_00456

gnl|Prokka|M1404_1 Prodigal:002006 CDS 501920 502384 . - 0 ID=M1404_00456;Parent=M1404_00456_gene;Name=pcp;db_xref=COG:COG3133;gene=pcp;inference=ab initio prediction:Prodigal:002006,similar to AA sequence:UniProtKB:P10325;locus_tag=M1404_00456;product=Outer membrane lipoprotein pcp;protein_id=gnl|Prokka|M1404_00456

gnl|Prokka|M1404_1 prokka gene 502412 502717 . - . ID=M1404_00457_gene;locus_tag=M1404_00457

gnl|Prokka|M1404_1 Prodigal:002006 CDS 502412 502717 . - 0 ID=M1404_00457;Parent=M1404_00457_gene;inference=ab initio prediction:Prodigal:002006;locus_tag=M1404_00457;product=hypothetical protein;protein_id=gnl|Prokka|M1404_00457

gnl|Prokka|M1404_1 prokka gene 502719 503309 . - . ID=M1404_00458_gene;Name=yecM;gene=yecM;locus_tag=M1404_00458

gnl|Prokka|M1404_1 Prodigal:002006 CDS 502719 503309 . - 0 ID=M1404_00458;Parent=M1404_00458_gene;Name=yecM;db_xref=COG:COG3102;gene=yecM;inference=ab initio prediction:Prodigal:002006,similar to AA sequence:UniProtKB:P52007;locus_tag=M1404_00458;product=Protein YecM;protein_id=gnl|Prokka|M1404_00458

gnl|Prokka|M1404_1 prokka gene 503396 505129 . + . ID=M1404_00459_gene;Name=argS;gene=argS;locus_tag=M1404_00459

gnl|Prokka|M1404_1 Prodigal:002006 CDS 503396 505129 . + 0 ID=M1404_00459;Parent=M1404_00459_gene;eC_number=6.1.1.19;Name=argS;db_xref=COG:COG0018;gene=argS;inference=ab initio prediction:Prodigal:002006,similar to AA sequence:UniProtKB:P11875;locus_tag=M1404_00459;product=Arginine--tRNA ligase;protein_id=gnl|Prokka|M1404_00459

gnl|Prokka|M1404_1 prokka gene 505430 506365 . - . ID=M1404_00460_gene;Name=mdh;gene=mdh;locus_tag=M1404_00460

gnl|Prokka|M1404_1 Prodigal:002006 CDS 505430 506365 . - 0 ID=M1404_00460;Parent=M1404_00460_gene;eC_number=1.1.1.37;Name=mdh;db_xref=COG:COG0039;gene=mdh;inference=ab initio prediction:Prodigal:002006,similar to AA sequence:UniProtKB:P44427;locus_tag=M1404_00460;product=Malate dehydrogenase;protein_id=gnl|Prokka|M1404_00460

gnl|Prokka|M1404_1 prokka gene 506584 507051 . + . ID=M1404_00461_gene;Name=argR;gene=argR;locus_tag=M1404_00461

gnl|Prokka|M1404_1 Prodigal:002006 CDS 506584 507051 . + 0 ID=M1404_00461;Parent=M1404_00461_gene;Name=argR;gene=argR;inference=ab initio prediction:Prodigal:002006,similar to AA sequence:UniProtKB:Q7MP98;locus_tag=M1404_00461;product=Arginine repressor;protein_id=gnl|Prokka|M1404_00461

gnl|Prokka|M1404_1 prokka gene 507075 507962 . + . ID=M1404_00462_gene;locus_tag=M1404_00462

gnl|Prokka|M1404_1 Prodigal:002006 CDS 507075 507962 . + 0 ID=M1404_00462;Parent=M1404_00462_gene;inference=ab initio prediction:Prodigal:002006,similar to AA sequence:UniProtKB:Q7A6Q5;locus_tag=M1404_00462;product=Epimerase family protein;protein_id=gnl|Prokka|M1404_00462

gnl|Prokka|M1404_1 prokka gene 508280 509284 . + . ID=M1404_00463_gene;Name=purR;gene=purR;locus_tag=M1404_00463

gnl|Prokka|M1404_1 Prodigal:002006 CDS 508280 509284 . + 0 ID=M1404_00463;Parent=M1404_00463_gene;Name=purR;db_xref=COG:COG1609;gene=purR;inference=ab initio prediction:Prodigal:002006,similar to AA sequence:UniProtKB:P0ACP7;locus_tag=M1404_00463;product=HTH-type transcriptional repressor PurR;protein_id=gnl|Prokka|M1404_00463

gnl|Prokka|M1404_1 prokka gene 509530 512169 . + . ID=M1404_00464_gene;Name=ppc;gene=ppc;locus_tag=M1404_00464

gnl|Prokka|M1404_1 Prodigal:002006 CDS 509530 512169 . + 0 ID=M1404_00464;Parent=M1404_00464_gene;eC_number=4.1.1.31;Name=ppc;db_xref=COG:COG2352;gene=ppc;inference=ab initio prediction:Prodigal:002006,similar to AA sequence:UniProtKB:P00864;locus_tag=M1404_00464;product=Phosphoenolpyruvate carboxylase;protein_id=gnl|Prokka|M1404_00464

gnl|Prokka|M1404_1 prokka gene 512275 514731 . - . ID=M1404_00465_gene;Name=malP;gene=malP;locus_tag=M1404_00465

gnl|Prokka|M1404_1 Prodigal:002006 CDS 512275 514731 . - 0 ID=M1404_00465;Parent=M1404_00465_gene;eC_number=2.4.1.1;Name=malP;db_xref=COG:COG0058;gene=malP;inference=ab initio prediction:Prodigal:002006,similar to AA sequence:UniProtKB:P00490;locus_tag=M1404_00465;product=Maltodextrin phosphorylase;protein_id=gnl|Prokka|M1404_00465

gnl|Prokka|M1404_1 prokka gene 514856 516298 . - . ID=M1404_00466_gene;Name=glgA;gene=glgA;locus_tag=M1404_00466

gnl|Prokka|M1404_1 Prodigal:002006 CDS 514856 516298 . - 0 ID=M1404_00466;Parent=M1404_00466_gene;eC_number=2.4.1.21;Name=glgA;db_xref=COG:COG0297;gene=glgA;inference=ab initio prediction:Prodigal:002006,similar to AA sequence:UniProtKB:P0A6U8;locus_tag=M1404_00466;product=Glycogen synthase;protein_id=gnl|Prokka|M1404_00466

gnl|Prokka|M1404_1 prokka gene 516378 517685 . - . ID=M1404_00467_gene;Name=glgC;gene=glgC;locus_tag=M1404_00467

gnl|Prokka|M1404_1 Prodigal:002006 CDS 516378 517685 . - 0 ID=M1404_00467;Parent=M1404_00467_gene;eC_number=2.7.7.27;Name=glgC;db_xref=COG:COG0448;gene=glgC;inference=ab initio prediction:Prodigal:002006,similar to AA sequence:UniProtKB:P0A6V1;locus_tag=M1404_00467;product=Glucose-1-phosphate adenylyltransferase;protein_id=gnl|Prokka|M1404_00467

gnl|Prokka|M1404_1 prokka gene 517758 519746 . - . ID=M1404_00468_gene;Name=glgX;gene=glgX;locus_tag=M1404_00468

gnl|Prokka|M1404_1 Prodigal:002006 CDS 517758 519746 . - 0 ID=M1404_00468;Parent=M1404_00468_gene;eC_number=3.2.1.196;Name=glgX;db_xref=COG:COG1523;gene=glgX;inference=ab initio prediction:Prodigal:002006,similar to AA sequence:UniProtKB:P15067;locus_tag=M1404_00468;product=Glycogen debranching enzyme;protein_id=gnl|Prokka|M1404_00468

gnl|Prokka|M1404_1 prokka gene 519820 522012 . - . ID=M1404_00469_gene;Name=glgB;gene=glgB;locus_tag=M1404_00469

gnl|Prokka|M1404_1 Prodigal:002006 CDS 519820 522012 . - 0 ID=M1404_00469;Parent=M1404_00469_gene;eC_number=2.4.1.18;Name=glgB;db_xref=COG:COG0296;gene=glgB;inference=ab initio prediction:Prodigal:002006,similar to AA sequence:UniProtKB:P07762;locus_tag=M1404_00469;product=1%2C4-alpha-glucan branching enzyme GlgB;protein_id=gnl|Prokka|M1404_00469

gnl|Prokka|M1404_1 prokka gene 522044 524137 . - . ID=M1404_00470_gene;Name=malQ;gene=malQ;locus_tag=M1404_00470

gnl|Prokka|M1404_1 Prodigal:002006 CDS 522044 524137 . - 0 ID=M1404_00470;Parent=M1404_00470_gene;eC_number=2.4.1.25;Name=malQ;db_xref=COG:COG1640;gene=malQ;inference=ab initio prediction:Prodigal:002006,similar to AA sequence:UniProtKB:P15977;locus_tag=M1404_00470;product=4-alpha-glucanotransferase;protein_id=gnl|Prokka|M1404_00470

gnl|Prokka|M1404_1 prokka gene 524592 525038 . - . ID=M1404_00471_gene;locus_tag=M1404_00471

gnl|Prokka|M1404_1 Prodigal:002006 CDS 524592 525038 . - 0 ID=M1404_00471;Parent=M1404_00471_gene;inference=ab initio prediction:Prodigal:002006;locus_tag=M1404_00471;product=hypothetical protein;protein_id=gnl|Prokka|M1404_00471

gnl|Prokka|M1404_1 prokka gene 525109 525729 . - . ID=M1404_00472_gene;Name=msrQ;gene=msrQ;locus_tag=M1404_00472

gnl|Prokka|M1404_1 Prodigal:002006 CDS 525109 525729 . - 0 ID=M1404_00472;Parent=M1404_00472_gene;Name=msrQ;db_xref=COG:COG2717;gene=msrQ;inference=ab initio prediction:Prodigal:002006,similar to AA sequence:UniProtKB:P76343;locus_tag=M1404_00472;product=Protein-methionine-sulfoxide reductase heme-binding subunit MsrQ;protein_id=gnl|Prokka|M1404_00472

gnl|Prokka|M1404_1 prokka gene 525729 526688 . - . ID=M1404_00473_gene;Name=msrP;gene=msrP;locus_tag=M1404_00473

gnl|Prokka|M1404_1 Prodigal:002006 CDS 525729 526688 . - 0 ID=M1404_00473;Parent=M1404_00473_gene;eC_number=1.8.5.-;Name=msrP;db_xref=COG:COG2041;gene=msrP;inference=ab initio prediction:Prodigal:002006,similar to AA sequence:UniProtKB:P76342;locus_tag=M1404_00473;product=Protein-methionine-sulfoxide reductase catalytic subunit MsrP;protein_id=gnl|Prokka|M1404_00473

gnl|Prokka|M1404_1 prokka gene 526895 527215 . + . ID=M1404_00474_gene;locus_tag=M1404_00474

gnl|Prokka|M1404_1 Prodigal:002006 CDS 526895 527215 . + 0 ID=M1404_00474;Parent=M1404_00474_gene;inference=ab initio prediction:Prodigal:002006,similar to AA sequence:UniProtKB:P44199;locus_tag=M1404_00474;note=UPF0263 protein HI_1450;product=hypothetical protein;protein_id=gnl|Prokka|M1404_00474

gnl|Prokka|M1404_1 prokka gene 527255 528697 . - . ID=M1404_00475_gene;Name=thiI;gene=thiI;locus_tag=M1404_00475

gnl|Prokka|M1404_1 Prodigal:002006 CDS 527255 528697 . - 0 ID=M1404_00475;Parent=M1404_00475_gene;eC_number=2.8.1.4;Name=thiI;db_xref=COG:COG0301;gene=thiI;inference=ab initio prediction:Prodigal:002006,similar to AA sequence:UniProtKB:Q8XE74;locus_tag=M1404_00475;product=tRNA sulfurtransferase;protein_id=gnl|Prokka|M1404_00475

gnl|Prokka|M1404_1 prokka gene 528898 529143 . + . ID=M1404_00476_gene;Name=xseB;gene=xseB;locus_tag=M1404_00476

gnl|Prokka|M1404_1 Prodigal:002006 CDS 528898 529143 . + 0 ID=M1404_00476;Parent=M1404_00476_gene;eC_number=3.1.11.6;Name=xseB;db_xref=COG:COG1722;gene=xseB;inference=ab initio prediction:Prodigal:002006,similar to AA sequence:UniProtKB:P0A8G9;locus_tag=M1404_00476;product=Exodeoxyribonuclease 7 small subunit;protein_id=gnl|Prokka|M1404_00476

gnl|Prokka|M1404_1 prokka gene 529162 530049 . + . ID=M1404_00477_gene;Name=ispA;gene=ispA;locus_tag=M1404_00477

gnl|Prokka|M1404_1 Prodigal:002006 CDS 529162 530049 . + 0 ID=M1404_00477;Parent=M1404_00477_gene;eC_number=2.5.1.10;Name=ispA;db_xref=COG:COG0142;gene=ispA;inference=ab initio prediction:Prodigal:002006,similar to AA sequence:UniProtKB:P22939;locus_tag=M1404_00477;product=Farnesyl diphosphate synthase;protein_id=gnl|Prokka|M1404_00477

gnl|Prokka|M1404_1 prokka gene 530116 531960 . + . ID=M1404_00478_gene;Name=dxs;gene=dxs;locus_tag=M1404_00478

gnl|Prokka|M1404_1 Prodigal:002006 CDS 530116 531960 . + 0 ID=M1404_00478;Parent=M1404_00478_gene;eC_number=2.2.1.7;Name=dxs;db_xref=COG:COG1154;gene=dxs;inference=ab initio prediction:Prodigal:002006,similar to AA sequence:UniProtKB:P77488;locus_tag=M1404_00478;product=1-deoxy-D-xylulose-5-phosphate synthase;protein_id=gnl|Prokka|M1404_00478

gnl|Prokka|M1404_1 prokka gene 532067 532606 . - . ID=M1404_00479_gene;locus_tag=M1404_00479

gnl|Prokka|M1404_1 Prodigal:002006 CDS 532067 532606 . - 0 ID=M1404_00479;Parent=M1404_00479_gene;inference=ab initio prediction:Prodigal:002006;locus_tag=M1404_00479;product=hypothetical protein;protein_id=gnl|Prokka|M1404_00479

gnl|Prokka|M1404_1 prokka gene 532591 533082 . - . ID=M1404_00480_gene;locus_tag=M1404_00480

gnl|Prokka|M1404_1 Prodigal:002006 CDS 532591 533082 . - 0 ID=M1404_00480;Parent=M1404_00480_gene;inference=ab initio prediction:Prodigal:002006;locus_tag=M1404_00480;product=hypothetical protein;protein_id=gnl|Prokka|M1404_00480

gnl|Prokka|M1404_1 prokka gene 533182 534627 . - . ID=M1404_00481_gene;Name=alsT_1;gene=alsT_1;locus_tag=M1404_00481

gnl|Prokka|M1404_1 Prodigal:002006 CDS 533182 534627 . - 0 ID=M1404_00481;Parent=M1404_00481_gene;Name=alsT_1;db_xref=COG:COG1115;gene=alsT_1;inference=ab initio prediction:Prodigal:002006,similar to AA sequence:UniProtKB:Q45068;locus_tag=M1404_00481;product=Amino-acid carrier protein AlsT;protein_id=gnl|Prokka|M1404_00481

gnl|Prokka|M1404_1 prokka gene 534832 536511 . - . ID=M1404_00482_gene;Name=glnS;gene=glnS;locus_tag=M1404_00482

gnl|Prokka|M1404_1 Prodigal:002006 CDS 534832 536511 . - 0 ID=M1404_00482;Parent=M1404_00482_gene;eC_number=6.1.1.18;Name=glnS;db_xref=COG:COG0008;gene=glnS;inference=ab initio prediction:Prodigal:002006,similar to AA sequence:UniProtKB:P00962;locus_tag=M1404_00482;product=Glutamine--tRNA ligase;protein_id=gnl|Prokka|M1404_00482

gnl|Prokka|M1404_1 prokka gene 536989 538356 . + . ID=M1404_00483_gene;Name=tdeA;gene=tdeA;locus_tag=M1404_00483

gnl|Prokka|M1404_1 Prodigal:002006 CDS 536989 538356 . + 0 ID=M1404_00483;Parent=M1404_00483_gene;Name=tdeA;db_xref=COG:COG1538;gene=tdeA;inference=ab initio prediction:Prodigal:002006,similar to AA sequence:UniProtKB:Q2EHL7;locus_tag=M1404_00483;product=Toxin and drug export protein A;protein_id=gnl|Prokka|M1404_00483

gnl|Prokka|M1404_1 prokka gene 538412 539146 . + . ID=M1404_00484_gene;Name=cutC;gene=cutC;locus_tag=M1404_00484

gnl|Prokka|M1404_1 Prodigal:002006 CDS 538412 539146 . + 0 ID=M1404_00484;Parent=M1404_00484_gene;Name=cutC;db_xref=COG:COG3142;gene=cutC;inference=ab initio prediction:Prodigal:002006,similar to AA sequence:UniProtKB:P67825;locus_tag=M1404_00484;product=Copper homeostasis protein CutC;protein_id=gnl|Prokka|M1404_00484

gnl|Prokka|M1404_1 prokka gene 539709 540644 . + . ID=M1404_00485_gene;Name=kpsF;gene=kpsF;locus_tag=M1404_00485

gnl|Prokka|M1404_1 Prodigal:002006 CDS 539709 540644 . + 0 ID=M1404_00485;Parent=M1404_00485_gene;eC_number=5.3.1.13;Name=kpsF;db_xref=COG:COG0517;gene=kpsF;inference=ab initio prediction:Prodigal:002006,similar to AA sequence:UniProtKB:Q8FDQ2;locus_tag=M1404_00485;product=Arabinose 5-phosphate isomerase KpsF;protein_id=gnl|Prokka|M1404_00485

gnl|Prokka|M1404_1 prokka gene 540644 541189 . + . ID=M1404_00486_gene;locus_tag=M1404_00486

gnl|Prokka|M1404_1 Prodigal:002006 CDS 540644 541189 . + 0 ID=M1404_00486;Parent=M1404_00486_gene;eC_number=3.1.3.45;db_xref=COG:COG1778;inference=ab initio prediction:Prodigal:002006,similar to AA sequence:UniProtKB:P45314;locus_tag=M1404_00486;product=3-deoxy-D-manno-octulosonate 8-phosphate phosphatase KdsC;protein_id=gnl|Prokka|M1404_00486

gnl|Prokka|M1404_1 prokka gene 541252 541734 . - . ID=M1404_00487_gene;Name=sspB;gene=sspB;locus_tag=M1404_00487

gnl|Prokka|M1404_1 Prodigal:002006 CDS 541252 541734 . - 0 ID=M1404_00487;Parent=M1404_00487_gene;Name=sspB;db_xref=COG:COG2969;gene=sspB;inference=ab initio prediction:Prodigal:002006,similar to AA sequence:UniProtKB:P45206;locus_tag=M1404_00487;product=Stringent starvation protein B ;protein_id=gnl|Prokka|M1404_00487

gnl|Prokka|M1404_1 prokka gene 541741 542379 . - . ID=M1404_00488_gene;Name=sspA;gene=sspA;locus_tag=M1404_00488

gnl|Prokka|M1404_1 Prodigal:002006 CDS 541741 542379 . - 0 ID=M1404_00488;Parent=M1404_00488_gene;Name=sspA;db_xref=COG:COG0625;gene=sspA;inference=ab initio prediction:Prodigal:002006,similar to AA sequence:UniProtKB:P45207;locus_tag=M1404_00488;product=Stringent starvation protein A ;protein_id=gnl|Prokka|M1404_00488

gnl|Prokka|M1404_1 prokka gene 542578 542970 . - . ID=M1404_00489_gene;Name=rpsI;gene=rpsI;locus_tag=M1404_00489

gnl|Prokka|M1404_1 Prodigal:002006 CDS 542578 542970 . - 0 ID=M1404_00489;Parent=M1404_00489_gene;Name=rpsI;db_xref=COG:COG0103;gene=rpsI;inference=ab initio prediction:Prodigal:002006,similar to AA sequence:UniProtKB:P0A7X3;locus_tag=M1404_00489;product=30S ribosomal protein S9;protein_id=gnl|Prokka|M1404_00489

gnl|Prokka|M1404_1 prokka gene 542987 543415 . - . ID=M1404_00490_gene;Name=rplM;gene=rplM;locus_tag=M1404_00490

gnl|Prokka|M1404_1 Prodigal:002006 CDS 542987 543415 . - 0 ID=M1404_00490;Parent=M1404_00490_gene;Name=rplM;db_xref=COG:COG0102;gene=rplM;inference=ab initio prediction:Prodigal:002006,similar to AA sequence:UniProtKB:P0AA10;locus_tag=M1404_00490;product=50S ribosomal protein L13;protein_id=gnl|Prokka|M1404_00490

gnl|Prokka|M1404_1 prokka gene 543696 544040 . - . ID=M1404_00491_gene;locus_tag=M1404_00491

gnl|Prokka|M1404_1 Prodigal:002006 CDS 543696 544040 . - 0 ID=M1404_00491;Parent=M1404_00491_gene;inference=ab initio prediction:Prodigal:002006,similar to AA sequence:UniProtKB:Q8EAL4;locus_tag=M1404_00491;note=UPF0339 protein SO_3888;product=hypothetical protein;protein_id=gnl|Prokka|M1404_00491

gnl|Prokka|M1404_1 prokka gene 544131 544778 . - . ID=M1404_00492_gene;Name=grxB;gene=grxB;locus_tag=M1404_00492

gnl|Prokka|M1404_1 Prodigal:002006 CDS 544131 544778 . - 0 ID=M1404_00492;Parent=M1404_00492_gene;Name=grxB;db_xref=COG:COG2999;gene=grxB;inference=ab initio prediction:Prodigal:002006,similar to AA sequence:UniProtKB:P0AC59;locus_tag=M1404_00492;product=Glutaredoxin 2;protein_id=gnl|Prokka|M1404_00492

gnl|Prokka|M1404_1 prokka gene 544816 546789 . - . ID=M1404_00493_gene;Name=recD;gene=recD;locus_tag=M1404_00493

gnl|Prokka|M1404_1 Prodigal:002006 CDS 544816 546789 . - 0 ID=M1404_00493;Parent=M1404_00493_gene;eC_number=3.1.11.5;Name=recD;db_xref=COG:COG0507;gene=recD;inference=ab initio prediction:Prodigal:002006,similar to AA sequence:UniProtKB:P04993;locus_tag=M1404_00493;product=RecBCD enzyme subunit RecD;protein_id=gnl|Prokka|M1404_00493

gnl|Prokka|M1404_1 prokka gene 546789 550481 . - . ID=M1404_00494_gene;Name=recB;gene=recB;locus_tag=M1404_00494

gnl|Prokka|M1404_1 Prodigal:002006 CDS 546789 550481 . - 0 ID=M1404_00494;Parent=M1404_00494_gene;eC_number=3.1.11.5;Name=recB;db_xref=COG:COG1074;gene=recB;inference=ab initio prediction:Prodigal:002006,similar to AA sequence:UniProtKB:P08394;locus_tag=M1404_00494;product=RecBCD enzyme subunit RecB;protein_id=gnl|Prokka|M1404_00494

gnl|Prokka|M1404_1 prokka gene 550542 550898 . - . ID=M1404_00495_gene;Name=ychN;gene=ychN;locus_tag=M1404_00495

gnl|Prokka|M1404_1 Prodigal:002006 CDS 550542 550898 . - 0 ID=M1404_00495;Parent=M1404_00495_gene;Name=ychN;db_xref=COG:COG1553;gene=ychN;inference=ab initio prediction:Prodigal:002006,similar to AA sequence:UniProtKB:P0AB52;locus_tag=M1404_00495;product=Protein YchN;protein_id=gnl|Prokka|M1404_00495

gnl|Prokka|M1404_1 prokka gene 550901 552211 . - . ID=M1404_00496_gene;Name=adeP;gene=adeP;locus_tag=M1404_00496

gnl|Prokka|M1404_1 Prodigal:002006 CDS 550901 552211 . - 0 ID=M1404_00496;Parent=M1404_00496_gene;Name=adeP;db_xref=COG:COG2252;gene=adeP;inference=ab initio prediction:Prodigal:002006,similar to AA sequence:UniProtKB:P31466;locus_tag=M1404_00496;product=Adenine permease AdeP;protein_id=gnl|Prokka|M1404_00496

gnl|Prokka|M1404_1 prokka gene 552384 553478 . - . ID=M1404_00497_gene;locus_tag=M1404_00497

gnl|Prokka|M1404_1 Prodigal:002006 CDS 552384 553478 . - 0 ID=M1404_00497;Parent=M1404_00497_gene;inference=ab initio prediction:Prodigal:002006,similar to AA sequence:ISfinder:TnXax1;locus_tag=M1404_00497;product=Tn3 family transposase TnXax1;protein_id=gnl|Prokka|M1404_00497

gnl|Prokka|M1404_1 prokka gene 553489 554289 . - . ID=M1404_00498_gene;Name=wbbD;gene=wbbD;locus_tag=M1404_00498

gnl|Prokka|M1404_1 Prodigal:002006 CDS 553489 554289 . - 0 ID=M1404_00498;Parent=M1404_00498_gene;eC_number=2.4.1.303;Name=wbbD;db_xref=COG:COG0463;gene=wbbD;inference=ab initio prediction:Prodigal:002006,similar to AA sequence:UniProtKB:Q03084;locus_tag=M1404_00498;product=UDP-Gal:alpha-D-GlcNAc-diphosphoundecaprenol beta-1%2C3-galactosyltransferase;protein_id=gnl|Prokka|M1404_00498

gnl|Prokka|M1404_1 prokka gene 554289 555173 . - . ID=M1404_00499_gene;locus_tag=M1404_00499

gnl|Prokka|M1404_1 Prodigal:002006 CDS 554289 555173 . - 0 ID=M1404_00499;Parent=M1404_00499_gene;inference=ab initio prediction:Prodigal:002006;locus_tag=M1404_00499;product=hypothetical protein;protein_id=gnl|Prokka|M1404_00499

gnl|Prokka|M1404_1 prokka gene 555173 556360 . - . ID=M1404_00500_gene;locus_tag=M1404_00500

gnl|Prokka|M1404_1 Prodigal:002006 CDS 555173 556360 . - 0 ID=M1404_00500;Parent=M1404_00500_gene;inference=ab initio prediction:Prodigal:002006;locus_tag=M1404_00500;product=hypothetical protein;protein_id=gnl|Prokka|M1404_00500

gnl|Prokka|M1404_1 prokka gene 556370 557155 . - . ID=M1404_00501_gene;locus_tag=M1404_00501

gnl|Prokka|M1404_1 Prodigal:002006 CDS 556370 557155 . - 0 ID=M1404_00501;Parent=M1404_00501_gene;inference=ab initio prediction:Prodigal:002006;locus_tag=M1404_00501;product=hypothetical protein;protein_id=gnl|Prokka|M1404_00501

gnl|Prokka|M1404_1 prokka gene 557157 558083 . - . ID=M1404_00502_gene;Name=lst;gene=lst;locus_tag=M1404_00502

gnl|Prokka|M1404_1 Prodigal:002006 CDS 557157 558083 . - 0 ID=M1404_00502;Parent=M1404_00502_gene;eC_number=2.4.99.-;Name=lst;gene=lst;inference=ab initio prediction:Prodigal:002006,similar to AA sequence:UniProtKB:Q9CNC4;locus_tag=M1404_00502;product=CMP-N-acetylneuraminate:beta-galactoside alpha-2%2C3-sialyltransferase;protein_id=gnl|Prokka|M1404_00502

gnl|Prokka|M1404_1 prokka gene 558085 559284 . - . ID=M1404_00503_gene;locus_tag=M1404_00503

gnl|Prokka|M1404_1 Prodigal:002006 CDS 558085 559284 . - 0 ID=M1404_00503;Parent=M1404_00503_gene;inference=ab initio prediction:Prodigal:002006;locus_tag=M1404_00503;product=hypothetical protein;protein_id=gnl|Prokka|M1404_00503

gnl|Prokka|M1404_1 prokka gene 559402 560145 . + . ID=M1404_00504_gene;locus_tag=M1404_00504

gnl|Prokka|M1404_1 Prodigal:002006 CDS 559402 560145 . + 0 ID=M1404_00504;Parent=M1404_00504_gene;inference=ab initio prediction:Prodigal:002006;locus_tag=M1404_00504;product=hypothetical protein;protein_id=gnl|Prokka|M1404_00504

gnl|Prokka|M1404_1 prokka gene 560225 560647 . - . ID=M1404_00505_gene;locus_tag=M1404_00505

gnl|Prokka|M1404_1 Prodigal:002006 CDS 560225 560647 . - 0 ID=M1404_00505;Parent=M1404_00505_gene;inference=ab initio prediction:Prodigal:002006;locus_tag=M1404_00505;product=hypothetical protein;protein_id=gnl|Prokka|M1404_00505

gnl|Prokka|M1404_1 prokka gene 561126 561530 . - . ID=M1404_00506_gene;locus_tag=M1404_00506

gnl|Prokka|M1404_1 Prodigal:002006 CDS 561126 561530 . - 0 ID=M1404_00506;Parent=M1404_00506_gene;inference=ab initio prediction:Prodigal:002006;locus_tag=M1404_00506;product=hypothetical protein;protein_id=gnl|Prokka|M1404_00506

gnl|Prokka|M1404_1 prokka gene 561709 562164 . - . ID=M1404_00507_gene;locus_tag=M1404_00507

gnl|Prokka|M1404_1 Prodigal:002006 CDS 561709 562164 . - 0 ID=M1404_00507;Parent=M1404_00507_gene;inference=ab initio prediction:Prodigal:002006;locus_tag=M1404_00507;product=hypothetical protein;protein_id=gnl|Prokka|M1404_00507

gnl|Prokka|M1404_1 prokka gene 562643 563029 . - . ID=M1404_00508_gene;locus_tag=M1404_00508

gnl|Prokka|M1404_1 Prodigal:002006 CDS 562643 563029 . - 0 ID=M1404_00508;Parent=M1404_00508_gene;inference=ab initio prediction:Prodigal:002006;locus_tag=M1404_00508;product=hypothetical protein;protein_id=gnl|Prokka|M1404_00508

gnl|Prokka|M1404_1 prokka gene 563272 563778 . - . ID=M1404_00509_gene;locus_tag=M1404_00509

gnl|Prokka|M1404_1 Prodigal:002006 CDS 563272 563778 . - 0 ID=M1404_00509;Parent=M1404_00509_gene;inference=ab initio prediction:Prodigal:002006;locus_tag=M1404_00509;product=hypothetical protein;protein_id=gnl|Prokka|M1404_00509

gnl|Prokka|M1404_1 prokka gene 563780 564010 . - . ID=M1404_00510_gene;locus_tag=M1404_00510

gnl|Prokka|M1404_1 Prodigal:002006 CDS 563780 564010 . - 0 ID=M1404_00510;Parent=M1404_00510_gene;inference=ab initio prediction:Prodigal:002006;locus_tag=M1404_00510;product=hypothetical protein;protein_id=gnl|Prokka|M1404_00510

gnl|Prokka|M1404_1 prokka gene 564231 564710 . - . ID=M1404_00511_gene;locus_tag=M1404_00511

gnl|Prokka|M1404_1 Prodigal:002006 CDS 564231 564710 . - 0 ID=M1404_00511;Parent=M1404_00511_gene;inference=ab initio prediction:Prodigal:002006;locus_tag=M1404_00511;product=hypothetical protein;protein_id=gnl|Prokka|M1404_00511

gnl|Prokka|M1404_1 prokka gene 564886 565401 . - . ID=M1404_00512_gene;locus_tag=M1404_00512

gnl|Prokka|M1404_1 Prodigal:002006 CDS 564886 565401 . - 0 ID=M1404_00512;Parent=M1404_00512_gene;inference=ab initio prediction:Prodigal:002006;locus_tag=M1404_00512;product=hypothetical protein;protein_id=gnl|Prokka|M1404_00512

gnl|Prokka|M1404_1 prokka gene 565733 566104 . - . ID=M1404_00513_gene;locus_tag=M1404_00513

gnl|Prokka|M1404_1 Prodigal:002006 CDS 565733 566104 . - 0 ID=M1404_00513;Parent=M1404_00513_gene;inference=ab initio prediction:Prodigal:002006;locus_tag=M1404_00513;product=hypothetical protein;protein_id=gnl|Prokka|M1404_00513

gnl|Prokka|M1404_1 prokka gene 566694 566942 . - . ID=M1404_00514_gene;locus_tag=M1404_00514

gnl|Prokka|M1404_1 Prodigal:002006 CDS 566694 566942 . - 0 ID=M1404_00514;Parent=M1404_00514_gene;inference=ab initio prediction:Prodigal:002006;locus_tag=M1404_00514;product=hypothetical protein;protein_id=gnl|Prokka|M1404_00514

gnl|Prokka|M1404_1 prokka gene 567165 567383 . - . ID=M1404_00515_gene;locus_tag=M1404_00515

gnl|Prokka|M1404_1 Prodigal:002006 CDS 567165 567383 . - 0 ID=M1404_00515;Parent=M1404_00515_gene;inference=ab initio prediction:Prodigal:002006;locus_tag=M1404_00515;product=hypothetical protein;protein_id=gnl|Prokka|M1404_00515

gnl|Prokka|M1404_1 prokka gene 567420 567926 . - . ID=M1404_00516_gene;locus_tag=M1404_00516

gnl|Prokka|M1404_1 Prodigal:002006 CDS 567420 567926 . - 0 ID=M1404_00516;Parent=M1404_00516_gene;inference=ab initio prediction:Prodigal:002006;locus_tag=M1404_00516;product=hypothetical protein;protein_id=gnl|Prokka|M1404_00516

gnl|Prokka|M1404_1 prokka gene 567929 568648 . - . ID=M1404_00517_gene;locus_tag=M1404_00517

gnl|Prokka|M1404_1 Prodigal:002006 CDS 567929 568648 . - 0 ID=M1404_00517;Parent=M1404_00517_gene;inference=ab initio prediction:Prodigal:002006;locus_tag=M1404_00517;product=hypothetical protein;protein_id=gnl|Prokka|M1404_00517

gnl|Prokka|M1404_1 prokka gene 568664 568891 . - . ID=M1404_00518_gene;locus_tag=M1404_00518

gnl|Prokka|M1404_1 Prodigal:002006 CDS 568664 568891 . - 0 ID=M1404_00518;Parent=M1404_00518_gene;inference=ab initio prediction:Prodigal:002006;locus_tag=M1404_00518;product=hypothetical protein;protein_id=gnl|Prokka|M1404_00518

gnl|Prokka|M1404_1 prokka gene 569708 570139 . - . ID=M1404_00519_gene;locus_tag=M1404_00519

gnl|Prokka|M1404_1 Prodigal:002006 CDS 569708 570139 . - 0 ID=M1404_00519;Parent=M1404_00519_gene;inference=ab initio prediction:Prodigal:002006;locus_tag=M1404_00519;product=hypothetical protein;protein_id=gnl|Prokka|M1404_00519

gnl|Prokka|M1404_1 prokka gene 570618 571121 . - . ID=M1404_00520_gene;locus_tag=M1404_00520

gnl|Prokka|M1404_1 Prodigal:002006 CDS 570618 571121 . - 0 ID=M1404_00520;Parent=M1404_00520_gene;inference=ab initio prediction:Prodigal:002006;locus_tag=M1404_00520;product=hypothetical protein;protein_id=gnl|Prokka|M1404_00520

gnl|Prokka|M1404_1 prokka gene 571121 572506 . - . ID=M1404_00521_gene;locus_tag=M1404_00521

gnl|Prokka|M1404_1 Prodigal:002006 CDS 571121 572506 . - 0 ID=M1404_00521;Parent=M1404_00521_gene;inference=ab initio prediction:Prodigal:002006;locus_tag=M1404_00521;product=hypothetical protein;protein_id=gnl|Prokka|M1404_00521

gnl|Prokka|M1404_1 prokka gene 572796 573059 . - . ID=M1404_00522_gene;locus_tag=M1404_00522

gnl|Prokka|M1404_1 Prodigal:002006 CDS 572796 573059 . - 0 ID=M1404_00522;Parent=M1404_00522_gene;inference=ab initio prediction:Prodigal:002006;locus_tag=M1404_00522;product=hypothetical protein;protein_id=gnl|Prokka|M1404_00522

gnl|Prokka|M1404_1 prokka gene 573056 573421 . - . ID=M1404_00523_gene;locus_tag=M1404_00523

gnl|Prokka|M1404_1 Prodigal:002006 CDS 573056 573421 . - 0 ID=M1404_00523;Parent=M1404_00523_gene;inference=ab initio prediction:Prodigal:002006;locus_tag=M1404_00523;product=hypothetical protein;protein_id=gnl|Prokka|M1404_00523

gnl|Prokka|M1404_1 prokka gene 573423 573854 . - . ID=M1404_00524_gene;locus_tag=M1404_00524

gnl|Prokka|M1404_1 Prodigal:002006 CDS 573423 573854 . - 0 ID=M1404_00524;Parent=M1404_00524_gene;inference=ab initio prediction:Prodigal:002006;locus_tag=M1404_00524;product=hypothetical protein;protein_id=gnl|Prokka|M1404_00524

gnl|Prokka|M1404_1 prokka gene 573880 575532 . - . ID=M1404_00525_gene;locus_tag=M1404_00525

gnl|Prokka|M1404_1 Prodigal:002006 CDS 573880 575532 . - 0 ID=M1404_00525;Parent=M1404_00525_gene;inference=ab initio prediction:Prodigal:002006;locus_tag=M1404_00525;product=hypothetical protein;protein_id=gnl|Prokka|M1404_00525

gnl|Prokka|M1404_1 prokka gene 575622 579122 . - . ID=M1404_00526_gene;locus_tag=M1404_00526

gnl|Prokka|M1404_1 Prodigal:002006 CDS 575622 579122 . - 0 ID=M1404_00526;Parent=M1404_00526_gene;inference=ab initio prediction:Prodigal:002006;locus_tag=M1404_00526;product=hypothetical protein;protein_id=gnl|Prokka|M1404_00526

gnl|Prokka|M1404_1 prokka gene 579200 580282 . - . ID=M1404_00527_gene;locus_tag=M1404_00527

gnl|Prokka|M1404_1 Prodigal:002006 CDS 579200 580282 . - 0 ID=M1404_00527;Parent=M1404_00527_gene;inference=ab initio prediction:Prodigal:002006;locus_tag=M1404_00527;product=hypothetical protein;protein_id=gnl|Prokka|M1404_00527

gnl|Prokka|M1404_1 prokka gene 580391 581056 . - . ID=M1404_00528_gene;locus_tag=M1404_00528

gnl|Prokka|M1404_1 Prodigal:002006 CDS 580391 581056 . - 0 ID=M1404_00528;Parent=M1404_00528_gene;inference=ab initio prediction:Prodigal:002006;locus_tag=M1404_00528;product=hypothetical protein;protein_id=gnl|Prokka|M1404_00528

gnl|Prokka|M1404_1 prokka gene 581909 582373 . - . ID=M1404_00529_gene;locus_tag=M1404_00529

gnl|Prokka|M1404_1 Prodigal:002006 CDS 581909 582373 . - 0 ID=M1404_00529;Parent=M1404_00529_gene;inference=ab initio prediction:Prodigal:002006;locus_tag=M1404_00529;product=hypothetical protein;protein_id=gnl|Prokka|M1404_00529

gnl|Prokka|M1404_1 prokka gene 582650 583021 . - . ID=M1404_00530_gene;locus_tag=M1404_00530

gnl|Prokka|M1404_1 Prodigal:002006 CDS 582650 583021 . - 0 ID=M1404_00530;Parent=M1404_00530_gene;inference=ab initio prediction:Prodigal:002006;locus_tag=M1404_00530;product=hypothetical protein;protein_id=gnl|Prokka|M1404_00530

gnl|Prokka|M1404_1 prokka gene 583260 583631 . - . ID=M1404_00531_gene;locus_tag=M1404_00531

gnl|Prokka|M1404_1 Prodigal:002006 CDS 583260 583631 . - 0 ID=M1404_00531;Parent=M1404_00531_gene;inference=ab initio prediction:Prodigal:002006;locus_tag=M1404_00531;product=hypothetical protein;protein_id=gnl|Prokka|M1404_00531

gnl|Prokka|M1404_1 prokka gene 583710 584228 . - . ID=M1404_00532_gene;locus_tag=M1404_00532

gnl|Prokka|M1404_1 Prodigal:002006 CDS 583710 584228 . - 0 ID=M1404_00532;Parent=M1404_00532_gene;inference=ab initio prediction:Prodigal:002006;locus_tag=M1404_00532;product=hypothetical protein;protein_id=gnl|Prokka|M1404_00532

gnl|Prokka|M1404_1 prokka gene 584514 585488 . - . ID=M1404_00533_gene;Name=usg;gene=usg;locus_tag=M1404_00533

gnl|Prokka|M1404_1 Prodigal:002006 CDS 584514 585488 . - 0 ID=M1404_00533;Parent=M1404_00533_gene;Name=usg;db_xref=COG:COG0136;gene=usg;inference=ab initio prediction:Prodigal:002006,similar to AA sequence:UniProtKB:P08390;locus_tag=M1404_00533;product=USG-1 protein;protein_id=gnl|Prokka|M1404_00533

gnl|Prokka|M1404_1 prokka gene 585545 586021 . + . ID=M1404_00534_gene;Name=ybaK;gene=ybaK;locus_tag=M1404_00534

gnl|Prokka|M1404_1 Prodigal:002006 CDS 585545 586021 . + 0 ID=M1404_00534;Parent=M1404_00534_gene;eC_number=4.2.-.-;Name=ybaK;db_xref=COG:COG2606;gene=ybaK;inference=ab initio prediction:Prodigal:002006,similar to AA sequence:UniProtKB:P45202;locus_tag=M1404_00534;product=Cys-tRNA(Pro)/Cys-tRNA(Cys) deacylase YbaK;protein_id=gnl|Prokka|M1404_00534

gnl|Prokka|M1404_1 prokka gene 586090 586620 . - . ID=M1404_00535_gene;Name=fabA;gene=fabA;locus_tag=M1404_00535

gnl|Prokka|M1404_1 Prodigal:002006 CDS 586090 586620 . - 0 ID=M1404_00535;Parent=M1404_00535_gene;eC_number=4.2.1.59;Name=fabA;db_xref=COG:COG0764;gene=fabA;inference=ab initio prediction:Prodigal:002006,similar to AA sequence:UniProtKB:P0A6Q3;locus_tag=M1404_00535;product=3-hydroxydecanoyl-[acyl-carrier-protein] dehydratase;protein_id=gnl|Prokka|M1404_00535

gnl|Prokka|M1404_1 prokka gene 586738 588528 . - . ID=M1404_00536_gene;locus_tag=M1404_00536

gnl|Prokka|M1404_1 Prodigal:002006 CDS 586738 588528 . - 0 ID=M1404_00536;Parent=M1404_00536_gene;inference=ab initio prediction:Prodigal:002006;locus_tag=M1404_00536;product=hypothetical protein;protein_id=gnl|Prokka|M1404_00536

gnl|Prokka|M1404_1 prokka gene 588693 589139 . + . ID=M1404_00537_gene;Name=matP;gene=matP;locus_tag=M1404_00537

gnl|Prokka|M1404_1 Prodigal:002006 CDS 588693 589139 . + 0 ID=M1404_00537;Parent=M1404_00537_gene;Name=matP;db_xref=COG:COG3120;gene=matP;inference=ab initio prediction:Prodigal:002006,similar to AA sequence:UniProtKB:Q8ZG78;locus_tag=M1404_00537;product=Macrodomain Ter protein;protein_id=gnl|Prokka|M1404_00537

gnl|Prokka|M1404_1 prokka gene 589503 589784 . + . ID=M1404_00538_gene;locus_tag=M1404_00538

gnl|Prokka|M1404_1 Prodigal:002006 CDS 589503 589784 . + 0 ID=M1404_00538;Parent=M1404_00538_gene;inference=ab initio prediction:Prodigal:002006,similar to AA sequence:ISfinder:ISAac3;locus_tag=M1404_00538;product=IS200/IS605 family transposase ISAac3;protein_id=gnl|Prokka|M1404_00538

gnl|Prokka|M1404_1 prokka gene 589927 590136 . - . ID=M1404_00539_gene;Name=cspD;gene=cspD;locus_tag=M1404_00539

gnl|Prokka|M1404_1 Prodigal:002006 CDS 589927 590136 . - 0 ID=M1404_00539;Parent=M1404_00539_gene;Name=cspD;db_xref=COG:COG1278;gene=cspD;inference=ab initio prediction:Prodigal:002006,similar to AA sequence:UniProtKB:P0A968;locus_tag=M1404_00539;product=Cold shock-like protein CspD;protein_id=gnl|Prokka|M1404_00539

gnl|Prokka|M1404_1 prokka gene 590342 590506 . - . ID=M1404_00540_gene;locus_tag=M1404_00540

gnl|Prokka|M1404_1 Prodigal:002006 CDS 590342 590506 . - 0 ID=M1404_00540;Parent=M1404_00540_gene;inference=ab initio prediction:Prodigal:002006;locus_tag=M1404_00540;product=hypothetical protein;protein_id=gnl|Prokka|M1404_00540

gnl|Prokka|M1404_1 prokka gene 590546 591277 . - . ID=M1404_00541_gene;Name=truC;gene=truC;locus_tag=M1404_00541

gnl|Prokka|M1404_1 Prodigal:002006 CDS 590546 591277 . - 0 ID=M1404_00541;Parent=M1404_00541_gene;eC_number=5.4.99.26;Name=truC;db_xref=COG:COG0564;gene=truC;inference=ab initio prediction:Prodigal:002006,similar to AA sequence:UniProtKB:P0AA41;locus_tag=M1404_00541;product=tRNA pseudouridine synthase C;protein_id=gnl|Prokka|M1404_00541

gnl|Prokka|M1404_1 prokka gene 591281 591592 . - . ID=M1404_00542_gene;locus_tag=M1404_00542

gnl|Prokka|M1404_1 Prodigal:002006 CDS 591281 591592 . - 0 ID=M1404_00542;Parent=M1404_00542_gene;inference=ab initio prediction:Prodigal:002006;locus_tag=M1404_00542;product=hypothetical protein;protein_id=gnl|Prokka|M1404_00542

gnl|Prokka|M1404_1 prokka gene 591729 592514 . + . ID=M1404_00543_gene;locus_tag=M1404_00543

gnl|Prokka|M1404_1 Prodigal:002006 CDS 591729 592514 . + 0 ID=M1404_00543;Parent=M1404_00543_gene;inference=ab initio prediction:Prodigal:002006;locus_tag=M1404_00543;product=hypothetical protein;protein_id=gnl|Prokka|M1404_00543

gnl|Prokka|M1404_1 prokka gene 592471 593397 . + . ID=M1404_00544_gene;Name=queF;gene=queF;locus_tag=M1404_00544

gnl|Prokka|M1404_1 Prodigal:002006 CDS 592471 593397 . + 0 ID=M1404_00544;Parent=M1404_00544_gene;eC_number=1.7.1.13;Name=queF;db_xref=COG:COG0780;gene=queF;inference=ab initio prediction:Prodigal:002006,similar to AA sequence:UniProtKB:Q46920;locus_tag=M1404_00544;product=NADPH-dependent 7-cyano-7-deazaguanine reductase;protein_id=gnl|Prokka|M1404_00544

gnl|Prokka|M1404_1 prokka gene 593406 594773 . + . ID=M1404_00545_gene;Name=ppnN;gene=ppnN;locus_tag=M1404_00545

gnl|Prokka|M1404_1 Prodigal:002006 CDS 593406 594773 . + 0 ID=M1404_00545;Parent=M1404_00545_gene;eC_number=3.2.2.-;Name=ppnN;db_xref=COG:COG1611;gene=ppnN;inference=ab initio prediction:Prodigal:002006,similar to AA sequence:UniProtKB:P0ADR8;locus_tag=M1404_00545;product=Pyrimidine/purine nucleotide 5'-monophosphate nucleosidase;protein_id=gnl|Prokka|M1404_00545

gnl|Prokka|M1404_1 prokka gene 594845 595267 . - . ID=M1404_00546_gene;locus_tag=M1404_00546

gnl|Prokka|M1404_1 Prodigal:002006 CDS 594845 595267 . - 0 ID=M1404_00546;Parent=M1404_00546_gene;inference=ab initio prediction:Prodigal:002006;locus_tag=M1404_00546;product=hypothetical protein;protein_id=gnl|Prokka|M1404_00546

gnl|Prokka|M1404_1 prokka gene 595277 597889 . - . ID=M1404_00547_gene;locus_tag=M1404_00547

gnl|Prokka|M1404_1 Prodigal:002006 CDS 595277 597889 . - 0 ID=M1404_00547;Parent=M1404_00547_gene;inference=ab initio prediction:Prodigal:002006;locus_tag=M1404_00547;product=hypothetical protein;protein_id=gnl|Prokka|M1404_00547

gnl|Prokka|M1404_1 prokka gene 597972 598931 . - . ID=M1404_00548_gene;locus_tag=M1404_00548

gnl|Prokka|M1404_1 Prodigal:002006 CDS 597972 598931 . - 0 ID=M1404_00548;Parent=M1404_00548_gene;inference=ab initio prediction:Prodigal:002006;locus_tag=M1404_00548;product=hypothetical protein;protein_id=gnl|Prokka|M1404_00548

gnl|Prokka|M1404_1 prokka gene 599091 599858 . - . ID=M1404_00549_gene;Name=yadH;gene=yadH;locus_tag=M1404_00549

gnl|Prokka|M1404_1 Prodigal:002006 CDS 599091 599858 . - 0 ID=M1404_00549;Parent=M1404_00549_gene;Name=yadH;db_xref=COG:COG0842;gene=yadH;inference=ab initio prediction:Prodigal:002006,similar to AA sequence:UniProtKB:P0AFN6;locus_tag=M1404_00549;product=Inner membrane transport permease YadH;protein_id=gnl|Prokka|M1404_00549

gnl|Prokka|M1404_1 prokka gene 599855 600793 . - . ID=M1404_00550_gene;Name=yadG;gene=yadG;locus_tag=M1404_00550

gnl|Prokka|M1404_1 Prodigal:002006 CDS 599855 600793 . - 0 ID=M1404_00550;Parent=M1404_00550_gene;Name=yadG;db_xref=COG:COG1131;gene=yadG;inference=ab initio prediction:Prodigal:002006,similar to AA sequence:UniProtKB:P36879;locus_tag=M1404_00550;product=putative ABC transporter ATP-binding protein YadG;protein_id=gnl|Prokka|M1404_00550

gnl|Prokka|M1404_1 prokka gene 600807 601343 . - . ID=M1404_00551_gene;Name=epmC;gene=epmC;locus_tag=M1404_00551

gnl|Prokka|M1404_1 Prodigal:002006 CDS 600807 601343 . - 0 ID=M1404_00551;Parent=M1404_00551_gene;eC_number=1.14.-.-;Name=epmC;db_xref=COG:COG3101;gene=epmC;inference=ab initio prediction:Prodigal:002006,similar to AA sequence:UniProtKB:P76938;locus_tag=M1404_00551;product=Elongation factor P hydroxylase;protein_id=gnl|Prokka|M1404_00551

gnl|Prokka|M1404_1 prokka gene 601438 602298 . - . ID=M1404_00552_gene;Name=htpX;gene=htpX;locus_tag=M1404_00552

gnl|Prokka|M1404_1 Prodigal:002006 CDS 601438 602298 . - 0 ID=M1404_00552;Parent=M1404_00552_gene;eC_number=3.4.24.-;Name=htpX;db_xref=COG:COG0501;gene=htpX;inference=ab initio prediction:Prodigal:002006,similar to AA sequence:UniProtKB:P65813;locus_tag=M1404_00552;product=Protease HtpX;protein_id=gnl|Prokka|M1404_00552

gnl|Prokka|M1404_1 prokka gene 602385 603461 . - . ID=M1404_00553_gene;Name=dinB;gene=dinB;locus_tag=M1404_00553

gnl|Prokka|M1404_1 Prodigal:002006 CDS 602385 603461 . - 0 ID=M1404_00553;Parent=M1404_00553_gene;eC_number=2.7.7.7;Name=dinB;db_xref=COG:COG0389;gene=dinB;inference=ab initio prediction:Prodigal:002006,similar to AA sequence:UniProtKB:Q47155;locus_tag=M1404_00553;product=DNA polymerase IV;protein_id=gnl|Prokka|M1404_00553

gnl|Prokka|M1404_1 prokka gene 603540 605102 . - . ID=M1404_00554_gene;locus_tag=M1404_00554

gnl|Prokka|M1404_1 Prodigal:002006 CDS 603540 605102 . - 0 ID=M1404_00554;Parent=M1404_00554_gene;inference=ab initio prediction:Prodigal:002006;locus_tag=M1404_00554;product=hypothetical protein;protein_id=gnl|Prokka|M1404_00554

gnl|Prokka|M1404_1 prokka gene 605141 606855 . - . ID=M1404_00555_gene;locus_tag=M1404_00555

gnl|Prokka|M1404_1 barrnap:0.9 rRNA 605141 606855 0 - . ID=M1404_00555;Parent=M1404_00555_gene;locus_tag=M1404_00555;note=aligned only 53 percent of the 23S ribosomal RNA;product=23S ribosomal RNA (partial)

gnl|Prokka|M1404_1 prokka gene 607083 607158 . - . ID=M1404_00556_gene;locus_tag=M1404_00556

gnl|Prokka|M1404_1 Aragorn:001002 tRNA 607083 607158 . - . ID=M1404_00556;Parent=M1404_00556_gene;inference=COORDINATES:profile:Aragorn:001002;locus_tag=M1404_00556;product=tRNA-Glu(ttc)

gnl|Prokka|M1404_1 prokka gene 607253 608790 . - . ID=M1404_00557_gene;locus_tag=M1404_00557

gnl|Prokka|M1404_1 barrnap:0.9 rRNA 607253 608790 0 - . ID=M1404_00557;Parent=M1404_00557_gene;locus_tag=M1404_00557;product=16S ribosomal RNA

gnl|Prokka|M1404_1 prokka gene 608968 609044 . - . ID=M1404_00558_gene;locus_tag=M1404_00558

gnl|Prokka|M1404_1 Aragorn:001002 tRNA 608968 609044 . - . ID=M1404_00558;Parent=M1404_00558_gene;inference=COORDINATES:profile:Aragorn:001002;locus_tag=M1404_00558;product=tRNA-Pro(tgg)

gnl|Prokka|M1404_1 prokka gene 609059 609134 . - . ID=M1404_00559_gene;locus_tag=M1404_00559

gnl|Prokka|M1404_1 Aragorn:001002 tRNA 609059 609134 . - . ID=M1404_00559;Parent=M1404_00559_gene;inference=COORDINATES:profile:Aragorn:001002;locus_tag=M1404_00559;product=tRNA-His(gtg)

gnl|Prokka|M1404_1 prokka gene 609170 609246 . - . ID=M1404_00560_gene;locus_tag=M1404_00560

gnl|Prokka|M1404_1 Aragorn:001002 tRNA 609170 609246 . - . ID=M1404_00560;Parent=M1404_00560_gene;inference=COORDINATES:profile:Aragorn:001002;locus_tag=M1404_00560;product=tRNA-Arg(ccg)

gnl|Prokka|M1404_1 prokka gene 609384 610217 . - . ID=M1404_00561_gene;Name=wzzE;gene=wzzE;locus_tag=M1404_00561

gnl|Prokka|M1404_1 Prodigal:002006 CDS 609384 610217 . - 0 ID=M1404_00561;Parent=M1404_00561_gene;Name=wzzE;db_xref=COG:COG3765;gene=wzzE;inference=ab initio prediction:Prodigal:002006,similar to AA sequence:UniProtKB:P0AG00;locus_tag=M1404_00561;product=ECA polysaccharide chain length modulation protein;protein_id=gnl|Prokka|M1404_00561

gnl|Prokka|M1404_1 prokka gene 610223 611296 . - . ID=M1404_00562_gene;Name=wecA;gene=wecA;locus_tag=M1404_00562

gnl|Prokka|M1404_1 Prodigal:002006 CDS 610223 611296 . - 0 ID=M1404_00562;Parent=M1404_00562_gene;eC_number=2.7.8.33;Name=wecA;db_xref=COG:COG0472;gene=wecA;inference=ab initio prediction:Prodigal:002006,similar to AA sequence:UniProtKB:P0AC78;locus_tag=M1404_00562;product=Undecaprenyl-phosphate alpha-N-acetylglucosaminyl 1-phosphate transferase;protein_id=gnl|Prokka|M1404_00562

gnl|Prokka|M1404_1 prokka gene 611429 612712 . + . ID=M1404_00563_gene;Name=hemL;gene=hemL;locus_tag=M1404_00563

gnl|Prokka|M1404_1 Prodigal:002006 CDS 611429 612712 . + 0 ID=M1404_00563;Parent=M1404_00563_gene;eC_number=5.4.3.8;Name=hemL;db_xref=COG:COG0001;gene=hemL;inference=ab initio prediction:Prodigal:002006,similar to AA sequence:UniProtKB:Q8ZBL9;locus_tag=M1404_00563;product=Glutamate-1-semialdehyde 2%2C1-aminomutase;protein_id=gnl|Prokka|M1404_00563

gnl|Prokka|M1404_1 prokka gene 612722 613582 . + . ID=M1404_00564_gene;locus_tag=M1404_00564

gnl|Prokka|M1404_1 Prodigal:002006 CDS 612722 613582 . + 0 ID=M1404_00564;Parent=M1404_00564_gene;inference=ab initio prediction:Prodigal:002006;locus_tag=M1404_00564;product=hypothetical protein;protein_id=gnl|Prokka|M1404_00564

gnl|Prokka|M1404_1 prokka gene 613678 616272 . - . ID=M1404_00565_gene;Name=glnD;gene=glnD;locus_tag=M1404_00565

gnl|Prokka|M1404_1 Prodigal:002006 CDS 613678 616272 . - 0 ID=M1404_00565;Parent=M1404_00565_gene;Name=glnD;db_xref=COG:COG2844;gene=glnD;inference=ab initio prediction:Prodigal:002006,similar to AA sequence:UniProtKB:P27249;locus_tag=M1404_00565;product=Bifunctional uridylyltransferase/uridylyl-removing enzyme;protein_id=gnl|Prokka|M1404_00565

gnl|Prokka|M1404_1 prokka gene 616333 617136 . - . ID=M1404_00566_gene;Name=map;gene=map;locus_tag=M1404_00566

gnl|Prokka|M1404_1 Prodigal:002006 CDS 616333 617136 . - 0 ID=M1404_00566;Parent=M1404_00566_gene;eC_number=3.4.11.18;Name=map;db_xref=COG:COG0024;gene=map;inference=ab initio prediction:Prodigal:002006,similar to AA sequence:UniProtKB:P0A1X6;locus_tag=M1404_00566;product=Methionine aminopeptidase;protein_id=gnl|Prokka|M1404_00566

gnl|Prokka|M1404_1 prokka gene 617275 617616 . + . ID=M1404_00567_gene;Name=erpA;gene=erpA;locus_tag=M1404_00567

gnl|Prokka|M1404_1 Prodigal:002006 CDS 617275 617616 . + 0 ID=M1404_00567;Parent=M1404_00567_gene;Name=erpA;db_xref=COG:COG0316;gene=erpA;inference=ab initio prediction:Prodigal:002006,similar to AA sequence:UniProtKB:P45344;locus_tag=M1404_00567;product=Iron-sulfur cluster insertion protein ErpA;protein_id=gnl|Prokka|M1404_00567

gnl|Prokka|M1404_1 prokka gene 617654 617974 . + . ID=M1404_00568_gene;locus_tag=M1404_00568

gnl|Prokka|M1404_1 Prodigal:002006 CDS 617654 617974 . + 0 ID=M1404_00568;Parent=M1404_00568_gene;inference=ab initio prediction:Prodigal:002006,similar to AA sequence:UniProtKB:P0A8E5;locus_tag=M1404_00568;note=UPF0231 protein YacL;product=hypothetical protein;protein_id=gnl|Prokka|M1404_00568

gnl|Prokka|M1404_1 prokka gene 618003 620330 . + . ID=M1404_00569_gene;Name=mrcB;gene=mrcB;locus_tag=M1404_00569

gnl|Prokka|M1404_1 Prodigal:002006 CDS 618003 620330 . + 0 ID=M1404_00569;Parent=M1404_00569_gene;Name=mrcB;db_xref=COG:COG0744;gene=mrcB;inference=ab initio prediction:Prodigal:002006,similar to AA sequence:UniProtKB:P02919;locus_tag=M1404_00569;product=Penicillin-binding protein 1B;protein_id=gnl|Prokka|M1404_00569

gnl|Prokka|M1404_1 prokka gene 620405 621295 . - . ID=M1404_00570_gene;Name=yfeX;gene=yfeX;locus_tag=M1404_00570

gnl|Prokka|M1404_1 Prodigal:002006 CDS 620405 621295 . - 0 ID=M1404_00570;Parent=M1404_00570_gene;eC_number=1.11.1.-;Name=yfeX;db_xref=COG:COG2837;gene=yfeX;inference=ab initio prediction:Prodigal:002006,similar to AA sequence:UniProtKB:P76536;locus_tag=M1404_00570;product=Dye-decolorizing peroxidase YfeX;protein_id=gnl|Prokka|M1404_00570

gnl|Prokka|M1404_1 prokka gene 621392 622096 . - . ID=M1404_00571_gene;Name=pepE;gene=pepE;locus_tag=M1404_00571

gnl|Prokka|M1404_1 Prodigal:002006 CDS 621392 622096 . - 0 ID=M1404_00571;Parent=M1404_00571_gene;eC_number=3.4.13.21;Name=pepE;db_xref=COG:COG3340;gene=pepE;inference=ab initio prediction:Prodigal:002006,similar to AA sequence:UniProtKB:P36936;locus_tag=M1404_00571;product=Peptidase E;protein_id=gnl|Prokka|M1404_00571

gnl|Prokka|M1404_1 prokka gene 622363 624267 . + . ID=M1404_00572_gene;locus_tag=M1404_00572

gnl|Prokka|M1404_1 Prodigal:002006 CDS 622363 624267 . + 0 ID=M1404_00572;Parent=M1404_00572_gene;inference=ab initio prediction:Prodigal:002006;locus_tag=M1404_00572;product=hypothetical protein;protein_id=gnl|Prokka|M1404_00572

gnl|Prokka|M1404_1 prokka gene 624307 624828 . + . ID=M1404_00573_gene;Name=tpd;gene=tpd;locus_tag=M1404_00573

gnl|Prokka|M1404_1 Prodigal:002006 CDS 624307 624828 . + 0 ID=M1404_00573;Parent=M1404_00573_gene;Name=tpd;db_xref=COG:COG3470;gene=tpd;inference=ab initio prediction:Prodigal:002006,similar to AA sequence:UniProtKB:P19478;locus_tag=M1404_00573;product=34 kDa membrane antigen;protein_id=gnl|Prokka|M1404_00573

gnl|Prokka|M1404_1 prokka gene 624994 626391 . + . ID=M1404_00574_gene;locus_tag=M1404_00574

gnl|Prokka|M1404_1 Prodigal:002006 CDS 624994 626391 . + 0 ID=M1404_00574;Parent=M1404_00574_gene;inference=ab initio prediction:Prodigal:002006;locus_tag=M1404_00574;product=hypothetical protein;protein_id=gnl|Prokka|M1404_00574

gnl|Prokka|M1404_1 prokka gene 626391 627713 . + . ID=M1404_00575_gene;locus_tag=M1404_00575

gnl|Prokka|M1404_1 Prodigal:002006 CDS 626391 627713 . + 0 ID=M1404_00575;Parent=M1404_00575_gene;inference=ab initio prediction:Prodigal:002006;locus_tag=M1404_00575;product=hypothetical protein;protein_id=gnl|Prokka|M1404_00575

gnl|Prokka|M1404_1 prokka gene 627700 628839 . + . ID=M1404_00576_gene;locus_tag=M1404_00576

gnl|Prokka|M1404_1 Prodigal:002006 CDS 627700 628839 . + 0 ID=M1404_00576;Parent=M1404_00576_gene;inference=ab initio prediction:Prodigal:002006;locus_tag=M1404_00576;product=hypothetical protein;protein_id=gnl|Prokka|M1404_00576

gnl|Prokka|M1404_1 prokka gene 628888 629583 . + . ID=M1404_00577_gene;Name=lolD_2;gene=lolD_2;locus_tag=M1404_00577

gnl|Prokka|M1404_1 Prodigal:002006 CDS 628888 629583 . + 0 ID=M1404_00577;Parent=M1404_00577_gene;eC_number=3.6.3.-;Name=lolD_2;db_xref=COG:COG1136;gene=lolD_2;inference=ab initio prediction:Prodigal:002006,similar to AA sequence:UniProtKB:O66646;locus_tag=M1404_00577;product=Lipoprotein-releasing system ATP-binding protein LolD;protein_id=gnl|Prokka|M1404_00577

gnl|Prokka|M1404_1 prokka gene 629549 630049 . + . ID=M1404_00578_gene;Name=resA;gene=resA;locus_tag=M1404_00578

gnl|Prokka|M1404_1 Prodigal:002006 CDS 629549 630049 . + 0 ID=M1404_00578;Parent=M1404_00578_gene;Name=resA;gene=resA;inference=ab initio prediction:Prodigal:002006,similar to AA sequence:UniProtKB:P35160;locus_tag=M1404_00578;product=Thiol-disulfide oxidoreductase ResA;protein_id=gnl|Prokka|M1404_00578

gnl|Prokka|M1404_1 prokka gene 630053 630355 . + . ID=M1404_00579_gene;locus_tag=M1404_00579

gnl|Prokka|M1404_1 Prodigal:002006 CDS 630053 630355 . + 0 ID=M1404_00579;Parent=M1404_00579_gene;inference=ab initio prediction:Prodigal:002006;locus_tag=M1404_00579;product=hypothetical protein;protein_id=gnl|Prokka|M1404_00579

gnl|Prokka|M1404_1 prokka gene 630415 630804 . + . ID=M1404_00580_gene;locus_tag=M1404_00580

gnl|Prokka|M1404_1 Prodigal:002006 CDS 630415 630804 . + 0 ID=M1404_00580;Parent=M1404_00580_gene;inference=ab initio prediction:Prodigal:002006;locus_tag=M1404_00580;product=hypothetical protein;protein_id=gnl|Prokka|M1404_00580

gnl|Prokka|M1404_1 prokka gene 630794 631045 . - . ID=M1404_00581_gene;Name=ycgL;gene=ycgL;locus_tag=M1404_00581

gnl|Prokka|M1404_1 Prodigal:002006 CDS 630794 631045 . - 0 ID=M1404_00581;Parent=M1404_00581_gene;Name=ycgL;db_xref=COG:COG3100;gene=ycgL;inference=ab initio prediction:Prodigal:002006,similar to AA sequence:UniProtKB:P0AB43;locus_tag=M1404_00581;product=Protein YcgL;protein_id=gnl|Prokka|M1404_00581

gnl|Prokka|M1404_1 prokka gene 631139 631816 . + . ID=M1404_00582_gene;Name=minC;gene=minC;locus_tag=M1404_00582

gnl|Prokka|M1404_1 Prodigal:002006 CDS 631139 631816 . + 0 ID=M1404_00582;Parent=M1404_00582_gene;Name=minC;db_xref=COG:COG0850;gene=minC;inference=ab initio prediction:Prodigal:002006,similar to AA sequence:UniProtKB:P18196;locus_tag=M1404_00582;product=Septum site-determining protein MinC;protein_id=gnl|Prokka|M1404_00582

gnl|Prokka|M1404_1 prokka gene 631865 632554 . - . ID=M1404_00583_gene;locus_tag=M1404_00583

gnl|Prokka|M1404_1 Prodigal:002006 CDS 631865 632554 . - 0 ID=M1404_00583;Parent=M1404_00583_gene;inference=ab initio prediction:Prodigal:002006;locus_tag=M1404_00583;product=hypothetical protein;protein_id=gnl|Prokka|M1404_00583

gnl|Prokka|M1404_1 prokka gene 632834 633292 . - . ID=M1404_00584_gene;Name=sixA;gene=sixA;locus_tag=M1404_00584

gnl|Prokka|M1404_1 Prodigal:002006 CDS 632834 633292 . - 0 ID=M1404_00584;Parent=M1404_00584_gene;eC_number=3.1.3.-;Name=sixA;db_xref=COG:COG2062;gene=sixA;inference=ab initio prediction:Prodigal:002006,similar to AA sequence:UniProtKB:P76502;locus_tag=M1404_00584;product=Phosphohistidine phosphatase SixA;protein_id=gnl|Prokka|M1404_00584

gnl|Prokka|M1404_1 prokka gene 633356 634690 . - . ID=M1404_00585_gene;Name=glmM;gene=glmM;locus_tag=M1404_00585

gnl|Prokka|M1404_1 Prodigal:002006 CDS 633356 634690 . - 0 ID=M1404_00585;Parent=M1404_00585_gene;eC_number=5.4.2.10;Name=glmM;db_xref=COG:COG1109;gene=glmM;inference=ab initio prediction:Prodigal:002006,similar to AA sequence:UniProtKB:P31120;locus_tag=M1404_00585;product=Phosphoglucosamine mutase;protein_id=gnl|Prokka|M1404_00585

gnl|Prokka|M1404_1 prokka gene 634710 635537 . - . ID=M1404_00586_gene;Name=folP;gene=folP;locus_tag=M1404_00586

gnl|Prokka|M1404_1 Prodigal:002006 CDS 634710 635537 . - 0 ID=M1404_00586;Parent=M1404_00586_gene;eC_number=2.5.1.15;Name=folP;db_xref=COG:COG0294;gene=folP;inference=ab initio prediction:Prodigal:002006,similar to AA sequence:UniProtKB:P0AC13;locus_tag=M1404_00586;product=Dihydropteroate synthase;protein_id=gnl|Prokka|M1404_00586

gnl|Prokka|M1404_1 prokka gene 635670 637598 . - . ID=M1404_00587_gene;Name=ftsH;gene=ftsH;locus_tag=M1404_00587

gnl|Prokka|M1404_1 Prodigal:002006 CDS 635670 637598 . - 0 ID=M1404_00587;Parent=M1404_00587_gene;eC_number=3.4.24.-;Name=ftsH;db_xref=COG:COG0465;gene=ftsH;inference=ab initio prediction:Prodigal:002006,similar to AA sequence:UniProtKB:P0AAI3;locus_tag=M1404_00587;product=ATP-dependent zinc metalloprotease FtsH;protein_id=gnl|Prokka|M1404_00587

gnl|Prokka|M1404_1 prokka gene 637721 638350 . - . ID=M1404_00588_gene;Name=rlmE;gene=rlmE;locus_tag=M1404_00588

gnl|Prokka|M1404_1 Prodigal:002006 CDS 637721 638350 . - 0 ID=M1404_00588;Parent=M1404_00588_gene;eC_number=2.1.1.166;Name=rlmE;db_xref=COG:COG0293;gene=rlmE;inference=ab initio prediction:Prodigal:002006,similar to AA sequence:UniProtKB:P0C0R7;locus_tag=M1404_00588;product=Ribosomal RNA large subunit methyltransferase E;protein_id=gnl|Prokka|M1404_00588

gnl|Prokka|M1404_1 prokka gene 638621 639655 . + . ID=M1404_00589_gene;Name=pstS;gene=pstS;locus_tag=M1404_00589

gnl|Prokka|M1404_1 Prodigal:002006 CDS 638621 639655 . + 0 ID=M1404_00589;Parent=M1404_00589_gene;Name=pstS;db_xref=COG:COG0226;gene=pstS;inference=ab initio prediction:Prodigal:002006,similar to AA sequence:UniProtKB:P0AG82;locus_tag=M1404_00589;product=Phosphate-binding protein PstS;protein_id=gnl|Prokka|M1404_00589

gnl|Prokka|M1404_1 prokka gene 639717 640679 . + . ID=M1404_00590_gene;Name=pstC;gene=pstC;locus_tag=M1404_00590

gnl|Prokka|M1404_1 Prodigal:002006 CDS 639717 640679 . + 0 ID=M1404_00590;Parent=M1404_00590_gene;Name=pstC;db_xref=COG:COG0573;gene=pstC;inference=ab initio prediction:Prodigal:002006,similar to AA sequence:UniProtKB:P0AGH8;locus_tag=M1404_00590;product=Phosphate transport system permease protein PstC;protein_id=gnl|Prokka|M1404_00590

gnl|Prokka|M1404_1 prokka gene 640680 641522 . + . ID=M1404_00591_gene;Name=pstA;gene=pstA;locus_tag=M1404_00591

gnl|Prokka|M1404_1 Prodigal:002006 CDS 640680 641522 . + 0 ID=M1404_00591;Parent=M1404_00591_gene;Name=pstA;db_xref=COG:COG0581;gene=pstA;inference=ab initio prediction:Prodigal:002006,similar to AA sequence:UniProtKB:P07654;locus_tag=M1404_00591;product=Phosphate transport system permease protein PstA;protein_id=gnl|Prokka|M1404_00591

gnl|Prokka|M1404_1 prokka gene 641540 642319 . + . ID=M1404_00592_gene;Name=pstB;gene=pstB;locus_tag=M1404_00592

gnl|Prokka|M1404_1 Prodigal:002006 CDS 641540 642319 . + 0 ID=M1404_00592;Parent=M1404_00592_gene;eC_number=7.3.2.1;Name=pstB;db_xref=COG:COG1117;gene=pstB;inference=ab initio prediction:Prodigal:002006,similar to AA sequence:UniProtKB:P0AAH0;locus_tag=M1404_00592;product=Phosphate import ATP-binding protein PstB;protein_id=gnl|Prokka|M1404_00592

gnl|Prokka|M1404_1 prokka gene 642446 643141 . + . ID=M1404_00593_gene;Name=phoB;gene=phoB;locus_tag=M1404_00593

gnl|Prokka|M1404_1 Prodigal:002006 CDS 642446 643141 . + 0 ID=M1404_00593;Parent=M1404_00593_gene;Name=phoB;db_xref=COG:COG0745;gene=phoB;inference=ab initio prediction:Prodigal:002006,similar to AA sequence:UniProtKB:P0AFJ5;locus_tag=M1404_00593;product=Phosphate regulon transcriptional regulatory protein PhoB;protein_id=gnl|Prokka|M1404_00593

gnl|Prokka|M1404_1 prokka gene 643138 644433 . + . ID=M1404_00594_gene;Name=phoR;gene=phoR;locus_tag=M1404_00594

gnl|Prokka|M1404_1 Prodigal:002006 CDS 643138 644433 . + 0 ID=M1404_00594;Parent=M1404_00594_gene;eC_number=2.7.13.3;Name=phoR;gene=phoR;inference=ab initio prediction:Prodigal:002006,similar to AA sequence:UniProtKB:P08400;locus_tag=M1404_00594;product=Phosphate regulon sensor protein PhoR;protein_id=gnl|Prokka|M1404_00594

gnl|Prokka|M1404_1 prokka gene 644480 645259 . - . ID=M1404_00595_gene;locus_tag=M1404_00595

gnl|Prokka|M1404_1 Prodigal:002006 CDS 644480 645259 . - 0 ID=M1404_00595;Parent=M1404_00595_gene;inference=ab initio prediction:Prodigal:002006;locus_tag=M1404_00595;product=hypothetical protein;protein_id=gnl|Prokka|M1404_00595

gnl|Prokka|M1404_1 prokka gene 645272 647308 . - . ID=M1404_00596_gene;Name=uvrB;gene=uvrB;locus_tag=M1404_00596

gnl|Prokka|M1404_1 Prodigal:002006 CDS 645272 647308 . - 0 ID=M1404_00596;Parent=M1404_00596_gene;Name=uvrB;db_xref=COG:COG0556;gene=uvrB;inference=ab initio prediction:Prodigal:002006,similar to AA sequence:UniProtKB:P0A8F8;locus_tag=M1404_00596;product=UvrABC system protein B;protein_id=gnl|Prokka|M1404_00596

gnl|Prokka|M1404_1 prokka gene 647707 647782 . + . ID=M1404_00597_gene;locus_tag=M1404_00597

gnl|Prokka|M1404_1 Aragorn:001002 tRNA 647707 647782 . + . ID=M1404_00597;Parent=M1404_00597_gene;inference=COORDINATES:profile:Aragorn:001002;locus_tag=M1404_00597;product=tRNA-Asn(gtt)

gnl|Prokka|M1404_1 prokka gene 647970 648938 . + . ID=M1404_00598_gene;Name=yrbG;gene=yrbG;locus_tag=M1404_00598

gnl|Prokka|M1404_1 Prodigal:002006 CDS 647970 648938 . + 0 ID=M1404_00598;Parent=M1404_00598_gene;Name=yrbG;db_xref=COG:COG0530;gene=yrbG;inference=ab initio prediction:Prodigal:002006,similar to AA sequence:UniProtKB:P45394;locus_tag=M1404_00598;product=Inner membrane protein YrbG;protein_id=gnl|Prokka|M1404_00598

gnl|Prokka|M1404_1 prokka gene 648939 649946 . - . ID=M1404_00599_gene;locus_tag=M1404_00599

gnl|Prokka|M1404_1 Prodigal:002006 CDS 648939 649946 . - 0 ID=M1404_00599;Parent=M1404_00599_gene;inference=ab initio prediction:Prodigal:002006;locus_tag=M1404_00599;product=hypothetical protein;protein_id=gnl|Prokka|M1404_00599

gnl|Prokka|M1404_1 prokka gene 649937 650578 . - . ID=M1404_00600_gene;locus_tag=M1404_00600

gnl|Prokka|M1404_1 Prodigal:002006 CDS 649937 650578 . - 0 ID=M1404_00600;Parent=M1404_00600_gene;inference=ab initio prediction:Prodigal:002006;locus_tag=M1404_00600;product=hypothetical protein;protein_id=gnl|Prokka|M1404_00600

gnl|Prokka|M1404_1 prokka gene 651127 652797 . + . ID=M1404_00601_gene;Name=ettA;gene=ettA;locus_tag=M1404_00601

gnl|Prokka|M1404_1 Prodigal:002006 CDS 651127 652797 . + 0 ID=M1404_00601;Parent=M1404_00601_gene;Name=ettA;db_xref=COG:COG0488;gene=ettA;inference=ab initio prediction:Prodigal:002006,similar to AA sequence:UniProtKB:P45127;locus_tag=M1404_00601;product=Energy-dependent translational throttle protein EttA;protein_id=gnl|Prokka|M1404_00601

gnl|Prokka|M1404_1 prokka gene 652837 653079 . - . ID=M1404_00602_gene;locus_tag=M1404_00602

gnl|Prokka|M1404_1 Prodigal:002006 CDS 652837 653079 . - 0 ID=M1404_00602;Parent=M1404_00602_gene;inference=ab initio prediction:Prodigal:002006;locus_tag=M1404_00602;product=hypothetical protein;protein_id=gnl|Prokka|M1404_00602

gnl|Prokka|M1404_1 prokka gene 653342 654268 . - . ID=M1404_00603_gene;Name=metR;gene=metR;locus_tag=M1404_00603

gnl|Prokka|M1404_1 Prodigal:002006 CDS 653342 654268 . - 0 ID=M1404_00603;Parent=M1404_00603_gene;Name=metR;gene=metR;inference=ab initio prediction:Prodigal:002006,similar to AA sequence:UniProtKB:P0A9F9;locus_tag=M1404_00603;product=HTH-type transcriptional regulator MetR;protein_id=gnl|Prokka|M1404_00603

gnl|Prokka|M1404_1 prokka gene 654567 656840 . + . ID=M1404_00604_gene;Name=metE;gene=metE;locus_tag=M1404_00604

gnl|Prokka|M1404_1 Prodigal:002006 CDS 654567 656840 . + 0 ID=M1404_00604;Parent=M1404_00604_gene;eC_number=2.1.1.14;Name=metE;db_xref=COG:COG0620;gene=metE;inference=ab initio prediction:Prodigal:002006,similar to AA sequence:UniProtKB:Q9JZQ2;locus_tag=M1404_00604;product=5-methyltetrahydropteroyltriglutamate--homocysteine methyltransferase;protein_id=gnl|Prokka|M1404_00604

gnl|Prokka|M1404_1 prokka gene 656998 658035 . + . ID=M1404_00605_gene;locus_tag=M1404_00605

gnl|Prokka|M1404_1 Prodigal:002006 CDS 656998 658035 . + 0 ID=M1404_00605;Parent=M1404_00605_gene;inference=ab initio prediction:Prodigal:002006;locus_tag=M1404_00605;product=hypothetical protein;protein_id=gnl|Prokka|M1404_00605

gnl|Prokka|M1404_1 prokka gene 658513 658722 . - . ID=M1404_00606_gene;locus_tag=M1404_00606

gnl|Prokka|M1404_1 Prodigal:002006 CDS 658513 658722 . - 0 ID=M1404_00606;Parent=M1404_00606_gene;inference=ab initio prediction:Prodigal:002006;locus_tag=M1404_00606;product=hypothetical protein;protein_id=gnl|Prokka|M1404_00606

gnl|Prokka|M1404_1 prokka gene 658920 659609 . + . ID=M1404_00607_gene;locus_tag=M1404_00607

gnl|Prokka|M1404_1 Prodigal:002006 CDS 658920 659609 . + 0 ID=M1404_00607;Parent=M1404_00607_gene;inference=ab initio prediction:Prodigal:002006;locus_tag=M1404_00607;product=hypothetical protein;protein_id=gnl|Prokka|M1404_00607

gnl|Prokka|M1404_1 prokka gene 659693 661342 . - . ID=M1404_00608_gene;Name=pgi;gene=pgi;locus_tag=M1404_00608

gnl|Prokka|M1404_1 Prodigal:002006 CDS 659693 661342 . - 0 ID=M1404_00608;Parent=M1404_00608_gene;eC_number=5.3.1.9;Name=pgi;db_xref=COG:COG0166;gene=pgi;inference=ab initio prediction:Prodigal:002006,similar to AA sequence:UniProtKB:P0A6T1;locus_tag=M1404_00608;product=Glucose-6-phosphate isomerase;protein_id=gnl|Prokka|M1404_00608

gnl|Prokka|M1404_1 prokka gene 662523 664331 . + . ID=M1404_00609_gene;locus_tag=M1404_00609

gnl|Prokka|M1404_1 Prodigal:002006 CDS 662523 664331 . + 0 ID=M1404_00609;Parent=M1404_00609_gene;inference=ab initio prediction:Prodigal:002006;locus_tag=M1404_00609;product=hypothetical protein;protein_id=gnl|Prokka|M1404_00609

gnl|Prokka|M1404_1 prokka gene 664422 665504 . - . ID=M1404_00610_gene;Name=alr;gene=alr;locus_tag=M1404_00610

gnl|Prokka|M1404_1 Prodigal:002006 CDS 664422 665504 . - 0 ID=M1404_00610;Parent=M1404_00610_gene;eC_number=5.1.1.1;Name=alr;db_xref=COG:COG0787;gene=alr;inference=ab initio prediction:Prodigal:002006,similar to AA sequence:UniProtKB:P0A6B5;locus_tag=M1404_00610;product=Alanine racemase%2C biosynthetic;protein_id=gnl|Prokka|M1404_00610

gnl|Prokka|M1404_1 prokka gene 665504 666907 . - . ID=M1404_00611_gene;Name=dnaB;gene=dnaB;locus_tag=M1404_00611

gnl|Prokka|M1404_1 Prodigal:002006 CDS 665504 666907 . - 0 ID=M1404_00611;Parent=M1404_00611_gene;eC_number=3.6.4.12;Name=dnaB;db_xref=COG:COG0305;gene=dnaB;inference=ab initio prediction:Prodigal:002006,similar to AA sequence:UniProtKB:P0ACB0;locus_tag=M1404_00611;product=Replicative DNA helicase;protein_id=gnl|Prokka|M1404_00611

gnl|Prokka|M1404_1 prokka gene 667068 669248 . - . ID=M1404_00612_gene;Name=uvrD;gene=uvrD;locus_tag=M1404_00612

gnl|Prokka|M1404_1 Prodigal:002006 CDS 667068 669248 . - 0 ID=M1404_00612;Parent=M1404_00612_gene;eC_number=3.6.4.12;Name=uvrD;db_xref=COG:COG0210;gene=uvrD;inference=ab initio prediction:Prodigal:002006,similar to AA sequence:UniProtKB:Q02322;locus_tag=M1404_00612;product=DNA helicase II;protein_id=gnl|Prokka|M1404_00612

gnl|Prokka|M1404_1 prokka gene 669409 670230 . - . ID=M1404_00613_gene;Name=fdhD;gene=fdhD;locus_tag=M1404_00613

gnl|Prokka|M1404_1 Prodigal:002006 CDS 669409 670230 . - 0 ID=M1404_00613;Parent=M1404_00613_gene;Name=fdhD;db_xref=COG:COG1526;gene=fdhD;inference=ab initio prediction:Prodigal:002006,similar to AA sequence:UniProtKB:P32177;locus_tag=M1404_00613;product=Sulfur carrier protein FdhD;protein_id=gnl|Prokka|M1404_00613

gnl|Prokka|M1404_1 prokka gene 670473 671060 . + . ID=M1404_00614_gene;Name=fdnG_1;gene=fdnG_1;locus_tag=M1404_00614

gnl|Prokka|M1404_1 Prodigal:002006 CDS 670473 671060 . + 0 ID=M1404_00614;Parent=M1404_00614_gene;eC_number=1.17.5.3;Name=fdnG_1;db_xref=COG:COG0243;gene=fdnG_1;inference=ab initio prediction:Prodigal:002006,similar to AA sequence:UniProtKB:P24183;locus_tag=M1404_00614;product=Formate dehydrogenase%2C nitrate-inducible%2C major subunit;protein_id=gnl|Prokka|M1404_00614

gnl|Prokka|M1404_1 prokka gene 671109 673538 . + . ID=M1404_00615_gene;Name=fdnG_2;gene=fdnG_2;locus_tag=M1404_00615

gnl|Prokka|M1404_1 Prodigal:002006 CDS 671109 673538 . + 0 ID=M1404_00615;Parent=M1404_00615_gene;eC_number=1.17.5.3;Name=fdnG_2;db_xref=COG:COG0243;gene=fdnG_2;inference=ab initio prediction:Prodigal:002006,similar to AA sequence:UniProtKB:P24183;locus_tag=M1404_00615;product=Formate dehydrogenase%2C nitrate-inducible%2C major subunit;protein_id=gnl|Prokka|M1404_00615

gnl|Prokka|M1404_1 prokka gene 673540 674448 . + . ID=M1404_00616_gene;Name=fdoH;gene=fdoH;locus_tag=M1404_00616

gnl|Prokka|M1404_1 Prodigal:002006 CDS 673540 674448 . + 0 ID=M1404_00616;Parent=M1404_00616_gene;Name=fdoH;db_xref=COG:COG0437;gene=fdoH;inference=ab initio prediction:Prodigal:002006,similar to AA sequence:UniProtKB:P0AAJ5;locus_tag=M1404_00616;product=Formate dehydrogenase-O iron-sulfur subunit;protein_id=gnl|Prokka|M1404_00616

gnl|Prokka|M1404_1 prokka gene 674441 675127 . + . ID=M1404_00617_gene;Name=fdnI;gene=fdnI;locus_tag=M1404_00617

gnl|Prokka|M1404_1 Prodigal:002006 CDS 674441 675127 . + 0 ID=M1404_00617;Parent=M1404_00617_gene;Name=fdnI;db_xref=COG:COG2864;gene=fdnI;inference=ab initio prediction:Prodigal:002006,similar to AA sequence:UniProtKB:P0AEK7;locus_tag=M1404_00617;product=Formate dehydrogenase%2C nitrate-inducible%2C cytochrome b556(Fdn) subunit;protein_id=gnl|Prokka|M1404_00617

gnl|Prokka|M1404_1 prokka gene 675218 676147 . + . ID=M1404_00618_gene;Name=fdhE;gene=fdhE;locus_tag=M1404_00618

gnl|Prokka|M1404_1 Prodigal:002006 CDS 675218 676147 . + 0 ID=M1404_00618;Parent=M1404_00618_gene;Name=fdhE;db_xref=COG:COG3058;gene=fdhE;inference=ab initio prediction:Prodigal:002006,similar to AA sequence:UniProtKB:P13024;locus_tag=M1404_00618;product=Protein FdhE;protein_id=gnl|Prokka|M1404_00618

gnl|Prokka|M1404_1 prokka gene 676305 676394 . - . ID=M1404_00619_gene;locus_tag=M1404_00619

gnl|Prokka|M1404_1 Aragorn:001002 tRNA 676305 676394 . - . ID=M1404_00619;Parent=M1404_00619_gene;inference=COORDINATES:profile:Aragorn:001002;locus_tag=M1404_00619;product=tRNA-Ser(tga)

gnl|Prokka|M1404_1 prokka gene 676610 677272 . + . ID=M1404_00620_gene;Name=yccA;gene=yccA;locus_tag=M1404_00620

gnl|Prokka|M1404_1 Prodigal:002006 CDS 676610 677272 . + 0 ID=M1404_00620;Parent=M1404_00620_gene;Name=yccA;db_xref=COG:COG0670;gene=yccA;inference=ab initio prediction:Prodigal:002006,similar to AA sequence:UniProtKB:P0AAC6;locus_tag=M1404_00620;product=Modulator of FtsH protease YccA;protein_id=gnl|Prokka|M1404_00620

gnl|Prokka|M1404_1 prokka gene 677371 677700 . + . ID=M1404_00621_gene;Name=tusE;gene=tusE;locus_tag=M1404_00621

gnl|Prokka|M1404_1 Prodigal:002006 CDS 677371 677700 . + 0 ID=M1404_00621;Parent=M1404_00621_gene;eC_number=2.8.1.-;Name=tusE;db_xref=COG:COG2920;gene=tusE;inference=ab initio prediction:Prodigal:002006,similar to AA sequence:UniProtKB:P0AB18;locus_tag=M1404_00621;product=Sulfurtransferase TusE;protein_id=gnl|Prokka|M1404_00621

gnl|Prokka|M1404_1 prokka gene 677813 678694 . + . ID=M1404_00622_gene;locus_tag=M1404_00622

gnl|Prokka|M1404_1 Prodigal:002006 CDS 677813 678694 . + 0 ID=M1404_00622;Parent=M1404_00622_gene;db_xref=COG:COG0803;inference=ab initio prediction:Prodigal:002006,similar to AA sequence:UniProtKB:Q57449;locus_tag=M1404_00622;product=putative periplasmic iron-binding protein;protein_id=gnl|Prokka|M1404_00622

gnl|Prokka|M1404_1 prokka gene 678694 679587 . + . ID=M1404_00623_gene;Name=mntB_1;gene=mntB_1;locus_tag=M1404_00623

gnl|Prokka|M1404_1 Prodigal:002006 CDS 678694 679587 . + 0 ID=M1404_00623;Parent=M1404_00623_gene;Name=mntB_1;db_xref=COG:COG1121;gene=mntB_1;inference=ab initio prediction:Prodigal:002006,similar to AA sequence:UniProtKB:O34338;locus_tag=M1404_00623;product=Manganese transport system ATP-binding protein MntB;protein_id=gnl|Prokka|M1404_00623

gnl|Prokka|M1404_1 prokka gene 679587 680456 . + . ID=M1404_00624_gene;Name=mntB_2;gene=mntB_2;locus_tag=M1404_00624

gnl|Prokka|M1404_1 Prodigal:002006 CDS 679587 680456 . + 0 ID=M1404_00624;Parent=M1404_00624_gene;Name=mntB_2;gene=mntB_2;inference=ab initio prediction:Prodigal:002006,similar to AA sequence:UniProtKB:Q55282;locus_tag=M1404_00624;product=Manganese transport system membrane protein MntB;protein_id=gnl|Prokka|M1404_00624

gnl|Prokka|M1404_1 prokka gene 680449 681288 . + . ID=M1404_00625_gene;Name=mntB_3;gene=mntB_3;locus_tag=M1404_00625

gnl|Prokka|M1404_1 Prodigal:002006 CDS 680449 681288 . + 0 ID=M1404_00625;Parent=M1404_00625_gene;Name=mntB_3;gene=mntB_3;inference=ab initio prediction:Prodigal:002006,similar to AA sequence:UniProtKB:Q55282;locus_tag=M1404_00625;product=Manganese transport system membrane protein MntB;protein_id=gnl|Prokka|M1404_00625

gnl|Prokka|M1404_1 prokka gene 681290 681562 . - . ID=M1404_00626_gene;Name=yccX;gene=yccX;locus_tag=M1404_00626

gnl|Prokka|M1404_1 Prodigal:002006 CDS 681290 681562 . - 0 ID=M1404_00626;Parent=M1404_00626_gene;eC_number=3.6.1.7;Name=yccX;db_xref=COG:COG1254;gene=yccX;inference=ab initio prediction:Prodigal:002006,similar to AA sequence:UniProtKB:P0AB65;locus_tag=M1404_00626;product=Acylphosphatase;protein_id=gnl|Prokka|M1404_00626

gnl|Prokka|M1404_1 prokka gene 681738 682394 . + . ID=M1404_00627_gene;locus_tag=M1404_00627

gnl|Prokka|M1404_1 Prodigal:002006 CDS 681738 682394 . + 0 ID=M1404_00627;Parent=M1404_00627_gene;inference=ab initio prediction:Prodigal:002006,similar to AA sequence:UniProtKB:P44290;locus_tag=M1404_00627;note=UPF0319 protein HI_1681;product=hypothetical protein;protein_id=gnl|Prokka|M1404_00627

gnl|Prokka|M1404_1 prokka gene 682453 682911 . + . ID=M1404_00628_gene;Name=mgsA;gene=mgsA;locus_tag=M1404_00628

gnl|Prokka|M1404_1 Prodigal:002006 CDS 682453 682911 . + 0 ID=M1404_00628;Parent=M1404_00628_gene;eC_number=4.2.3.3;Name=mgsA;db_xref=COG:COG1803;gene=mgsA;inference=ab initio prediction:Prodigal:002006,similar to AA sequence:UniProtKB:P0A731;locus_tag=M1404_00628;product=Methylglyoxal synthase;protein_id=gnl|Prokka|M1404_00628

gnl|Prokka|M1404_1 prokka gene 683039 683491 . + . ID=M1404_00629_gene;Name=yccF;gene=yccF;locus_tag=M1404_00629

gnl|Prokka|M1404_1 Prodigal:002006 CDS 683039 683491 . + 0 ID=M1404_00629;Parent=M1404_00629_gene;Name=yccF;db_xref=COG:COG3304;gene=yccF;inference=ab initio prediction:Prodigal:002006,similar to AA sequence:UniProtKB:P0AB12;locus_tag=M1404_00629;product=Inner membrane protein YccF;protein_id=gnl|Prokka|M1404_00629

gnl|Prokka|M1404_1 prokka gene 683525 685678 . + . ID=M1404_00630_gene;Name=yccS;gene=yccS;locus_tag=M1404_00630

gnl|Prokka|M1404_1 Prodigal:002006 CDS 683525 685678 . + 0 ID=M1404_00630;Parent=M1404_00630_gene;Name=yccS;db_xref=COG:COG1289;gene=yccS;inference=ab initio prediction:Prodigal:002006,similar to AA sequence:UniProtKB:P75870;locus_tag=M1404_00630;product=Inner membrane protein YccS;protein_id=gnl|Prokka|M1404_00630

gnl|Prokka|M1404_1 prokka gene 685658 686164 . - . ID=M1404_00631_gene;locus_tag=M1404_00631

gnl|Prokka|M1404_1 Prodigal:002006 CDS 685658 686164 . - 0 ID=M1404_00631;Parent=M1404_00631_gene;inference=ab initio prediction:Prodigal:002006;locus_tag=M1404_00631;product=hypothetical protein;protein_id=gnl|Prokka|M1404_00631

gnl|Prokka|M1404_1 prokka gene 686287 687228 . + . ID=M1404_00632_gene;Name=prmB;gene=prmB;locus_tag=M1404_00632

gnl|Prokka|M1404_1 Prodigal:002006 CDS 686287 687228 . + 0 ID=M1404_00632;Parent=M1404_00632_gene;eC_number=2.1.1.298;Name=prmB;db_xref=COG:COG2890;gene=prmB;inference=ab initio prediction:Prodigal:002006,similar to AA sequence:UniProtKB:P39199;locus_tag=M1404_00632;product=50S ribosomal protein L3 glutamine methyltransferase;protein_id=gnl|Prokka|M1404_00632

gnl|Prokka|M1404_1 prokka gene 687331 688383 . - . ID=M1404_00633_gene;locus_tag=M1404_00633

gnl|Prokka|M1404_1 Prodigal:002006 CDS 687331 688383 . - 0 ID=M1404_00633;Parent=M1404_00633_gene;inference=ab initio prediction:Prodigal:002006;locus_tag=M1404_00633;product=hypothetical protein;protein_id=gnl|Prokka|M1404_00633

gnl|Prokka|M1404_1 prokka gene 688537 689544 . - . ID=M1404_00634_gene;locus_tag=M1404_00634

gnl|Prokka|M1404_1 Prodigal:002006 CDS 688537 689544 . - 0 ID=M1404_00634;Parent=M1404_00634_gene;inference=ab initio prediction:Prodigal:002006;locus_tag=M1404_00634;product=hypothetical protein;protein_id=gnl|Prokka|M1404_00634

gnl|Prokka|M1404_1 prokka gene 690235 690813 . + . ID=M1404_00635_gene;Name=rnfA;gene=rnfA;locus_tag=M1404_00635

gnl|Prokka|M1404_1 Prodigal:002006 CDS 690235 690813 . + 0 ID=M1404_00635;Parent=M1404_00635_gene;eC_number=7.-.-.-;Name=rnfA;gene=rnfA;inference=ab initio prediction:Prodigal:002006,similar to AA sequence:UniProtKB:A0A0H3AKU6;locus_tag=M1404_00635;product=Ion-translocating oxidoreductase complex subunit A;protein_id=gnl|Prokka|M1404_00635

gnl|Prokka|M1404_1 prokka gene 690810 691406 . + . ID=M1404_00636_gene;Name=rnfB;gene=rnfB;locus_tag=M1404_00636

gnl|Prokka|M1404_1 Prodigal:002006 CDS 690810 691406 . + 0 ID=M1404_00636;Parent=M1404_00636_gene;eC_number=7.-.-.-;Name=rnfB;db_xref=COG:COG2878;gene=rnfB;inference=ab initio prediction:Prodigal:002006,similar to AA sequence:UniProtKB:A5F2R3;locus_tag=M1404_00636;product=Ion-translocating oxidoreductase complex subunit B;protein_id=gnl|Prokka|M1404_00636

gnl|Prokka|M1404_1 prokka gene 691411 693918 . + . ID=M1404_00637_gene;Name=rsxC;gene=rsxC;locus_tag=M1404_00637

gnl|Prokka|M1404_1 Prodigal:002006 CDS 691411 693918 . + 0 ID=M1404_00637;Parent=M1404_00637_gene;eC_number=7.-.-.-;Name=rsxC;gene=rsxC;inference=ab initio prediction:Prodigal:002006,protein motif:HAMAP:MF_00461;locus_tag=M1404_00637;product=Ion-translocating oxidoreductase complex subunit C;protein_id=gnl|Prokka|M1404_00637

gnl|Prokka|M1404_1 prokka gene 693933 694982 . + . ID=M1404_00638_gene;Name=rnfD;gene=rnfD;locus_tag=M1404_00638

gnl|Prokka|M1404_1 Prodigal:002006 CDS 693933 694982 . + 0 ID=M1404_00638;Parent=M1404_00638_gene;eC_number=7.-.-.-;Name=rnfD;db_xref=COG:COG4658;gene=rnfD;inference=ab initio prediction:Prodigal:002006,similar to AA sequence:UniProtKB:A5F2R1;locus_tag=M1404_00638;product=Ion-translocating oxidoreductase complex subunit D;protein_id=gnl|Prokka|M1404_00638

gnl|Prokka|M1404_1 prokka gene 694992 695600 . + . ID=M1404_00639_gene;Name=rnfG;gene=rnfG;locus_tag=M1404_00639

gnl|Prokka|M1404_1 Prodigal:002006 CDS 694992 695600 . + 0 ID=M1404_00639;Parent=M1404_00639_gene;eC_number=7.-.-.-;Name=rnfG;db_xref=COG:COG4659;gene=rnfG;inference=ab initio prediction:Prodigal:002006,similar to AA sequence:UniProtKB:A5F2S8;locus_tag=M1404_00639;product=Ion-translocating oxidoreductase complex subunit G;protein_id=gnl|Prokka|M1404_00639

gnl|Prokka|M1404_1 prokka gene 695603 696331 . + . ID=M1404_00640_gene;Name=rnfE;gene=rnfE;locus_tag=M1404_00640

gnl|Prokka|M1404_1 Prodigal:002006 CDS 695603 696331 . + 0 ID=M1404_00640;Parent=M1404_00640_gene;eC_number=7.-.-.-;Name=rnfE;db_xref=COG:COG4660;gene=rnfE;inference=ab initio prediction:Prodigal:002006,similar to AA sequence:UniProtKB:A5F2S7;locus_tag=M1404_00640;product=Ion-translocating oxidoreductase complex subunit E;protein_id=gnl|Prokka|M1404_00640

gnl|Prokka|M1404_1 prokka gene 696328 696960 . + . ID=M1404_00641_gene;Name=nth;gene=nth;locus_tag=M1404_00641

gnl|Prokka|M1404_1 Prodigal:002006 CDS 696328 696960 . + 0 ID=M1404_00641;Parent=M1404_00641_gene;eC_number=4.2.99.18;Name=nth;db_xref=COG:COG0177;gene=nth;inference=ab initio prediction:Prodigal:002006,similar to AA sequence:UniProtKB:P0AB83;locus_tag=M1404_00641;product=Endonuclease III;protein_id=gnl|Prokka|M1404_00641

gnl|Prokka|M1404_1 prokka gene 696985 698352 . + . ID=M1404_00642_gene;locus_tag=M1404_00642

gnl|Prokka|M1404_1 Prodigal:002006 CDS 696985 698352 . + 0 ID=M1404_00642;Parent=M1404_00642_gene;inference=ab initio prediction:Prodigal:002006;locus_tag=M1404_00642;product=hypothetical protein;protein_id=gnl|Prokka|M1404_00642

gnl|Prokka|M1404_1 prokka gene 698379 699389 . - . ID=M1404_00643_gene;Name=bioB;gene=bioB;locus_tag=M1404_00643

gnl|Prokka|M1404_1 Prodigal:002006 CDS 698379 699389 . - 0 ID=M1404_00643;Parent=M1404_00643_gene;eC_number=2.8.1.6;Name=bioB;db_xref=COG:COG0502;gene=bioB;inference=ab initio prediction:Prodigal:002006,similar to AA sequence:UniProtKB:P12996;locus_tag=M1404_00643;product=Biotin synthase;protein_id=gnl|Prokka|M1404_00643

gnl|Prokka|M1404_1 prokka gene 699424 700101 . - . ID=M1404_00644_gene;Name=thiQ;gene=thiQ;locus_tag=M1404_00644

gnl|Prokka|M1404_1 Prodigal:002006 CDS 699424 700101 . - 0 ID=M1404_00644;Parent=M1404_00644_gene;eC_number=3.6.3.-;Name=thiQ;db_xref=COG:COG3840;gene=thiQ;inference=ab initio prediction:Prodigal:002006,similar to AA sequence:UniProtKB:P44986;locus_tag=M1404_00644;product=Thiamine import ATP-binding protein ThiQ;protein_id=gnl|Prokka|M1404_00644

gnl|Prokka|M1404_1 prokka gene 700094 701704 . - . ID=M1404_00645_gene;locus_tag=M1404_00645

gnl|Prokka|M1404_1 Prodigal:002006 CDS 700094 701704 . - 0 ID=M1404_00645;Parent=M1404_00645_gene;inference=ab initio prediction:Prodigal:002006;locus_tag=M1404_00645;product=hypothetical protein;protein_id=gnl|Prokka|M1404_00645

gnl|Prokka|M1404_1 prokka gene 701717 702721 . - . ID=M1404_00646_gene;Name=thiB;gene=thiB;locus_tag=M1404_00646

gnl|Prokka|M1404_1 Prodigal:002006 CDS 701717 702721 . - 0 ID=M1404_00646;Parent=M1404_00646_gene;Name=thiB;db_xref=COG:COG4143;gene=thiB;inference=ab initio prediction:Prodigal:002006,similar to AA sequence:UniProtKB:P31550;locus_tag=M1404_00646;product=Thiamine-binding periplasmic protein;protein_id=gnl|Prokka|M1404_00646

gnl|Prokka|M1404_1 prokka gene 703016 705976 . + . ID=M1404_00647_gene;Name=glnE;gene=glnE;locus_tag=M1404_00647

gnl|Prokka|M1404_1 Prodigal:002006 CDS 703016 705976 . + 0 ID=M1404_00647;Parent=M1404_00647_gene;Name=glnE;db_xref=COG:COG1391;gene=glnE;inference=ab initio prediction:Prodigal:002006,similar to AA sequence:UniProtKB:P30870;locus_tag=M1404_00647;product=Bifunctional glutamine synthetase adenylyltransferase/adenylyl-removing enzyme;protein_id=gnl|Prokka|M1404_00647

gnl|Prokka|M1404_1 prokka gene 706036 707517 . - . ID=M1404_00648_gene;locus_tag=M1404_00648

gnl|Prokka|M1404_1 Prodigal:002006 CDS 706036 707517 . - 0 ID=M1404_00648;Parent=M1404_00648_gene;inference=ab initio prediction:Prodigal:002006;locus_tag=M1404_00648;product=hypothetical protein;protein_id=gnl|Prokka|M1404_00648

gnl|Prokka|M1404_1 prokka gene 707549 708463 . - . ID=M1404_00649_gene;locus_tag=M1404_00649

gnl|Prokka|M1404_1 Prodigal:002006 CDS 707549 708463 . - 0 ID=M1404_00649;Parent=M1404_00649_gene;inference=ab initio prediction:Prodigal:002006;locus_tag=M1404_00649;product=hypothetical protein;protein_id=gnl|Prokka|M1404_00649

gnl|Prokka|M1404_1 prokka gene 708619 709857 . - . ID=M1404_00650_gene;locus_tag=M1404_00650

gnl|Prokka|M1404_1 Prodigal:002006 CDS 708619 709857 . - 0 ID=M1404_00650;Parent=M1404_00650_gene;eC_number=1.5.1.43;inference=ab initio prediction:Prodigal:002006,similar to AA sequence:UniProtKB:P0DPE4;locus_tag=M1404_00650;product=Carboxynorspermidine synthase;protein_id=gnl|Prokka|M1404_00650

gnl|Prokka|M1404_1 prokka gene 710057 711172 . - . ID=M1404_00651_gene;Name=nspC;gene=nspC;locus_tag=M1404_00651

gnl|Prokka|M1404_1 Prodigal:002006 CDS 710057 711172 . - 0 ID=M1404_00651;Parent=M1404_00651_gene;eC_number=4.1.1.96;Name=nspC;db_xref=COG:COG0019;gene=nspC;inference=ab initio prediction:Prodigal:002006,similar to AA sequence:UniProtKB:Q5QCP2;locus_tag=M1404_00651;product=Carboxynorspermidine/carboxyspermidine decarboxylase;protein_id=gnl|Prokka|M1404_00651

gnl|Prokka|M1404_1 prokka gene 711375 713273 . + . ID=M1404_00652_gene;Name=parE;gene=parE;locus_tag=M1404_00652

gnl|Prokka|M1404_1 Prodigal:002006 CDS 711375 713273 . + 0 ID=M1404_00652;Parent=M1404_00652_gene;eC_number=5.6.2.2;Name=parE;db_xref=COG:COG0187;gene=parE;inference=ab initio prediction:Prodigal:002006,similar to AA sequence:UniProtKB:P20083;locus_tag=M1404_00652;product=DNA topoisomerase 4 subunit B;protein_id=gnl|Prokka|M1404_00652

gnl|Prokka|M1404_1 prokka gene 713541 715793 . + . ID=M1404_00653_gene;Name=parC;gene=parC;locus_tag=M1404_00653

gnl|Prokka|M1404_1 Prodigal:002006 CDS 713541 715793 . + 0 ID=M1404_00653;Parent=M1404_00653_gene;eC_number=5.6.2.2;Name=parC;db_xref=COG:COG0188;gene=parC;inference=ab initio prediction:Prodigal:002006,similar to AA sequence:UniProtKB:P0AFI2;locus_tag=M1404_00653;product=DNA topoisomerase 4 subunit A;protein_id=gnl|Prokka|M1404_00653

gnl|Prokka|M1404_1 prokka gene 715967 716431 . + . ID=M1404_00654_gene;locus_tag=M1404_00654

gnl|Prokka|M1404_1 Prodigal:002006 CDS 715967 716431 . + 0 ID=M1404_00654;Parent=M1404_00654_gene;inference=ab initio prediction:Prodigal:002006,similar to AA sequence:ISfinder:ISAac3;locus_tag=M1404_00654;product=IS200/IS605 family transposase ISAac3;protein_id=gnl|Prokka|M1404_00654

gnl|Prokka|M1404_1 prokka gene 716542 717270 . - . ID=M1404_00655_gene;locus_tag=M1404_00655

gnl|Prokka|M1404_1 Prodigal:002006 CDS 716542 717270 . - 0 ID=M1404_00655;Parent=M1404_00655_gene;inference=ab initio prediction:Prodigal:002006;locus_tag=M1404_00655;product=hypothetical protein;protein_id=gnl|Prokka|M1404_00655

gnl|Prokka|M1404_1 prokka gene 717280 717996 . - . ID=M1404_00656_gene;locus_tag=M1404_00656

gnl|Prokka|M1404_1 Prodigal:002006 CDS 717280 717996 . - 0 ID=M1404_00656;Parent=M1404_00656_gene;inference=ab initio prediction:Prodigal:002006;locus_tag=M1404_00656;product=hypothetical protein;protein_id=gnl|Prokka|M1404_00656

gnl|Prokka|M1404_1 prokka gene 718098 718451 . + . ID=M1404_00657_gene;Name=yabJ;gene=yabJ;locus_tag=M1404_00657

gnl|Prokka|M1404_1 Prodigal:002006 CDS 718098 718451 . + 0 ID=M1404_00657;Parent=M1404_00657_gene;eC_number=3.5.99.10;Name=yabJ;db_xref=COG:COG0251;gene=yabJ;inference=ab initio prediction:Prodigal:002006,similar to AA sequence:UniProtKB:P37552;locus_tag=M1404_00657;product=2-iminobutanoate/2-iminopropanoate deaminase;protein_id=gnl|Prokka|M1404_00657

gnl|Prokka|M1404_1 prokka gene 718577 719902 . + . ID=M1404_00658_gene;Name=nhaP;gene=nhaP;locus_tag=M1404_00658

gnl|Prokka|M1404_1 Prodigal:002006 CDS 718577 719902 . + 0 ID=M1404_00658;Parent=M1404_00658_gene;Name=nhaP;db_xref=COG:COG0025;gene=nhaP;inference=ab initio prediction:Prodigal:002006,similar to AA sequence:UniProtKB:G3XD29;locus_tag=M1404_00658;product=Na(+)/H(+) antiporter NhaP;protein_id=gnl|Prokka|M1404_00658

gnl|Prokka|M1404_1 prokka gene 719956 722100 . - . ID=M1404_00659_gene;locus_tag=M1404_00659

gnl|Prokka|M1404_1 Prodigal:002006 CDS 719956 722100 . - 0 ID=M1404_00659;Parent=M1404_00659_gene;inference=ab initio prediction:Prodigal:002006;locus_tag=M1404_00659;product=hypothetical protein;protein_id=gnl|Prokka|M1404_00659

gnl|Prokka|M1404_1 prokka gene 722135 722677 . - . ID=M1404_00660_gene;Name=apt;gene=apt;locus_tag=M1404_00660

gnl|Prokka|M1404_1 Prodigal:002006 CDS 722135 722677 . - 0 ID=M1404_00660;Parent=M1404_00660_gene;eC_number=2.4.2.7;Name=apt;gene=apt;inference=ab initio prediction:Prodigal:002006,similar to AA sequence:UniProtKB:Q66DQ2;locus_tag=M1404_00660;product=Adenine phosphoribosyltransferase;protein_id=gnl|Prokka|M1404_00660

gnl|Prokka|M1404_1 prokka gene 722912 723862 . - . ID=M1404_00661_gene;Name=lpxM;gene=lpxM;locus_tag=M1404_00661

gnl|Prokka|M1404_1 Prodigal:002006 CDS 722912 723862 . - 0 ID=M1404_00661;Parent=M1404_00661_gene;eC_number=2.3.1.243;Name=lpxM;db_xref=COG:COG1560;gene=lpxM;inference=ab initio prediction:Prodigal:002006,similar to AA sequence:UniProtKB:P24205;locus_tag=M1404_00661;product=Lipid A biosynthesis myristoyltransferase;protein_id=gnl|Prokka|M1404_00661

gnl|Prokka|M1404_1 prokka gene 723874 724638 . - . ID=M1404_00662_gene;Name=yfcA;gene=yfcA;locus_tag=M1404_00662

gnl|Prokka|M1404_1 Prodigal:002006 CDS 723874 724638 . - 0 ID=M1404_00662;Parent=M1404_00662_gene;Name=yfcA;db_xref=COG:COG0730;gene=yfcA;inference=ab initio prediction:Prodigal:002006,similar to AA sequence:UniProtKB:P0AD30;locus_tag=M1404_00662;product=putative membrane transporter protein YfcA;protein_id=gnl|Prokka|M1404_00662

gnl|Prokka|M1404_1 prokka gene 724648 725523 . - . ID=M1404_00663_gene;Name=mepA;gene=mepA;locus_tag=M1404_00663

gnl|Prokka|M1404_1 Prodigal:002006 CDS 724648 725523 . - 0 ID=M1404_00663;Parent=M1404_00663_gene;eC_number=3.4.24.-;Name=mepA;db_xref=COG:COG3770;gene=mepA;inference=ab initio prediction:Prodigal:002006,similar to AA sequence:UniProtKB:P0C0T5;locus_tag=M1404_00663;product=Penicillin-insensitive murein endopeptidase;protein_id=gnl|Prokka|M1404_00663

gnl|Prokka|M1404_1 prokka gene 725573 726646 . - . ID=M1404_00664_gene;Name=aroC;gene=aroC;locus_tag=M1404_00664

gnl|Prokka|M1404_1 Prodigal:002006 CDS 725573 726646 . - 0 ID=M1404_00664;Parent=M1404_00664_gene;eC_number=4.2.3.5;Name=aroC;db_xref=COG:COG0082;gene=aroC;inference=ab initio prediction:Prodigal:002006,similar to AA sequence:UniProtKB:P12008;locus_tag=M1404_00664;product=Chorismate synthase;protein_id=gnl|Prokka|M1404_00664

gnl|Prokka|M1404_1 prokka gene 726650 729991 . - . ID=M1404_00665_gene;locus_tag=M1404_00665

gnl|Prokka|M1404_1 Prodigal:002006 CDS 726650 729991 . - 0 ID=M1404_00665;Parent=M1404_00665_gene;db_xref=COG:COG3264;inference=ab initio prediction:Prodigal:002006,similar to AA sequence:UniProtKB:Q57362;locus_tag=M1404_00665;product=putative MscS family protein.1;protein_id=gnl|Prokka|M1404_00665

gnl|Prokka|M1404_1 prokka gene 729988 731406 . - . ID=M1404_00666_gene;Name=menE;gene=menE;locus_tag=M1404_00666

gnl|Prokka|M1404_1 Prodigal:002006 CDS 729988 731406 . - 0 ID=M1404_00666;Parent=M1404_00666_gene;eC_number=6.2.1.26;Name=menE;db_xref=COG:COG0318;gene=menE;inference=ab initio prediction:Prodigal:002006,similar to AA sequence:UniProtKB:P37353;locus_tag=M1404_00666;product=2-succinylbenzoate--CoA ligase;protein_id=gnl|Prokka|M1404_00666

gnl|Prokka|M1404_1 prokka gene 731409 732071 . - . ID=M1404_00667_gene;Name=seqA;gene=seqA;locus_tag=M1404_00667

gnl|Prokka|M1404_1 Prodigal:002006 CDS 731409 732071 . - 0 ID=M1404_00667;Parent=M1404_00667_gene;Name=seqA;db_xref=COG:COG3057;gene=seqA;inference=ab initio prediction:Prodigal:002006,similar to AA sequence:UniProtKB:P0AFY8;locus_tag=M1404_00667;product=Negative modulator of initiation of replication;protein_id=gnl|Prokka|M1404_00667

gnl|Prokka|M1404_1 prokka gene 732109 732897 . + . ID=M1404_00668_gene;Name=ybfF;gene=ybfF;locus_tag=M1404_00668

gnl|Prokka|M1404_1 Prodigal:002006 CDS 732109 732897 . + 0 ID=M1404_00668;Parent=M1404_00668_gene;eC_number=3.1.-.-;Name=ybfF;db_xref=COG:COG0596;gene=ybfF;inference=ab initio prediction:Prodigal:002006,similar to AA sequence:UniProtKB:P75736;locus_tag=M1404_00668;product=Esterase YbfF;protein_id=gnl|Prokka|M1404_00668

gnl|Prokka|M1404_1 prokka gene 733013 733297 . + . ID=M1404_00669_gene;Name=ybfE;gene=ybfE;locus_tag=M1404_00669

gnl|Prokka|M1404_1 Prodigal:002006 CDS 733013 733297 . + 0 ID=M1404_00669;Parent=M1404_00669_gene;Name=ybfE;gene=ybfE;inference=ab initio prediction:Prodigal:002006,similar to AA sequence:UniProtKB:P0AAU7;locus_tag=M1404_00669;product=putative protein YbfE;protein_id=gnl|Prokka|M1404_00669

gnl|Prokka|M1404_1 prokka gene 733303 733827 . + . ID=M1404_00670_gene;Name=fldA;gene=fldA;locus_tag=M1404_00670

gnl|Prokka|M1404_1 Prodigal:002006 CDS 733303 733827 . + 0 ID=M1404_00670;Parent=M1404_00670_gene;Name=fldA;db_xref=COG:COG0716;gene=fldA;inference=ab initio prediction:Prodigal:002006,similar to AA sequence:UniProtKB:P61949;locus_tag=M1404_00670;product=Flavodoxin 1;protein_id=gnl|Prokka|M1404_00670

gnl|Prokka|M1404_1 prokka gene 733852 734292 . + . ID=M1404_00671_gene;Name=fur;gene=fur;locus_tag=M1404_00671

gnl|Prokka|M1404_1 Prodigal:002006 CDS 733852 734292 . + 0 ID=M1404_00671;Parent=M1404_00671_gene;Name=fur;db_xref=COG:COG0735;gene=fur;inference=ab initio prediction:Prodigal:002006,similar to AA sequence:UniProtKB:P0A9A9;locus_tag=M1404_00671;product=Ferric uptake regulation protein;protein_id=gnl|Prokka|M1404_00671

gnl|Prokka|M1404_1 prokka gene 734423 734704 . - . ID=M1404_00672_gene;locus_tag=M1404_00672

gnl|Prokka|M1404_1 Prodigal:002006 CDS 734423 734704 . - 0 ID=M1404_00672;Parent=M1404_00672_gene;inference=ab initio prediction:Prodigal:002006,similar to AA sequence:ISfinder:ISAac3;locus_tag=M1404_00672;product=IS200/IS605 family transposase ISAac3;protein_id=gnl|Prokka|M1404_00672

gnl|Prokka|M1404_1 prokka gene 735095 736267 . - . ID=M1404_00673_gene;Name=obgE;gene=obgE;locus_tag=M1404_00673

gnl|Prokka|M1404_1 Prodigal:002006 CDS 735095 736267 . - 0 ID=M1404_00673;Parent=M1404_00673_gene;eC_number=3.6.5.-;Name=obgE;db_xref=COG:COG0536;gene=obgE;inference=ab initio prediction:Prodigal:002006,similar to AA sequence:UniProtKB:P42641;locus_tag=M1404_00673;product=GTPase ObgE/CgtA;protein_id=gnl|Prokka|M1404_00673

gnl|Prokka|M1404_1 prokka gene 736276 737193 . - . ID=M1404_00674_gene;Name=yhbE_1;gene=yhbE_1;locus_tag=M1404_00674

gnl|Prokka|M1404_1 Prodigal:002006 CDS 736276 737193 . - 0 ID=M1404_00674;Parent=M1404_00674_gene;Name=yhbE_1;db_xref=COG:COG0697;gene=yhbE_1;inference=ab initio prediction:Prodigal:002006,similar to AA sequence:UniProtKB:P0AA73;locus_tag=M1404_00674;product=putative inner membrane transporter YhbE;protein_id=gnl|Prokka|M1404_00674

gnl|Prokka|M1404_1 prokka gene 737195 738127 . - . ID=M1404_00675_gene;Name=yhbE_2;gene=yhbE_2;locus_tag=M1404_00675

gnl|Prokka|M1404_1 Prodigal:002006 CDS 737195 738127 . - 0 ID=M1404_00675;Parent=M1404_00675_gene;Name=yhbE_2;db_xref=COG:COG0697;gene=yhbE_2;inference=ab initio prediction:Prodigal:002006,similar to AA sequence:UniProtKB:P0AA73;locus_tag=M1404_00675;product=putative inner membrane transporter YhbE;protein_id=gnl|Prokka|M1404_00675

gnl|Prokka|M1404_1 prokka gene 738192 738449 . - . ID=M1404_00676_gene;Name=rpmA;gene=rpmA;locus_tag=M1404_00676

gnl|Prokka|M1404_1 Prodigal:002006 CDS 738192 738449 . - 0 ID=M1404_00676;Parent=M1404_00676_gene;Name=rpmA;db_xref=COG:COG0211;gene=rpmA;inference=ab initio prediction:Prodigal:002006,similar to AA sequence:UniProtKB:P0A7L8;locus_tag=M1404_00676;product=50S ribosomal protein L27;protein_id=gnl|Prokka|M1404_00676

gnl|Prokka|M1404_1 prokka gene 738470 738781 . - . ID=M1404_00677_gene;Name=rplU;gene=rplU;locus_tag=M1404_00677

gnl|Prokka|M1404_1 Prodigal:002006 CDS 738470 738781 . - 0 ID=M1404_00677;Parent=M1404_00677_gene;Name=rplU;db_xref=COG:COG0261;gene=rplU;inference=ab initio prediction:Prodigal:002006,similar to AA sequence:UniProtKB:P0AG48;locus_tag=M1404_00677;product=50S ribosomal protein L21;protein_id=gnl|Prokka|M1404_00677

gnl|Prokka|M1404_1 prokka gene 739000 739992 . + . ID=M1404_00678_gene;Name=ispB;gene=ispB;locus_tag=M1404_00678

gnl|Prokka|M1404_1 Prodigal:002006 CDS 739000 739992 . + 0 ID=M1404_00678;Parent=M1404_00678_gene;eC_number=2.5.1.90;Name=ispB;db_xref=COG:COG0142;gene=ispB;inference=ab initio prediction:Prodigal:002006,similar to AA sequence:UniProtKB:P44916;locus_tag=M1404_00678;product=Octaprenyl diphosphate synthase;protein_id=gnl|Prokka|M1404_00678

gnl|Prokka|M1404_1 prokka gene 740071 740772 . + . ID=M1404_00679_gene;Name=queH;gene=queH;locus_tag=M1404_00679

gnl|Prokka|M1404_1 Prodigal:002006 CDS 740071 740772 . + 0 ID=M1404_00679;Parent=M1404_00679_gene;eC_number=1.17.99.6;Name=queH;db_xref=COG:COG1636;gene=queH;inference=ab initio prediction:Prodigal:002006,similar to AA sequence:UniProtKB:Q0I1Q0;locus_tag=M1404_00679;product=Epoxyqueuosine reductase QueH;protein_id=gnl|Prokka|M1404_00679

gnl|Prokka|M1404_1 prokka gene 740828 742024 . - . ID=M1404_00680_gene;Name=argD;gene=argD;locus_tag=M1404_00680

gnl|Prokka|M1404_1 Prodigal:002006 CDS 740828 742024 . - 0 ID=M1404_00680;Parent=M1404_00680_gene;eC_number=2.6.1.11;Name=argD;db_xref=COG:COG4992;gene=argD;inference=ab initio prediction:Prodigal:002006,similar to AA sequence:UniProtKB:P18335;locus_tag=M1404_00680;product=Acetylornithine/succinyldiaminopimelate aminotransferase;protein_id=gnl|Prokka|M1404_00680

gnl|Prokka|M1404_1 prokka gene 742153 743601 . - . ID=M1404_00681_gene;Name=tldD;gene=tldD;locus_tag=M1404_00681

gnl|Prokka|M1404_1 Prodigal:002006 CDS 742153 743601 . - 0 ID=M1404_00681;Parent=M1404_00681_gene;eC_number=3.4.-.-;Name=tldD;db_xref=COG:COG0312;gene=tldD;inference=ab initio prediction:Prodigal:002006,similar to AA sequence:UniProtKB:P0AGG8;locus_tag=M1404_00681;product=Metalloprotease TldD;protein_id=gnl|Prokka|M1404_00681

gnl|Prokka|M1404_1 prokka gene 743782 744123 . - . ID=M1404_00682_gene;locus_tag=M1404_00682

gnl|Prokka|M1404_1 Prodigal:002006 CDS 743782 744123 . - 0 ID=M1404_00682;Parent=M1404_00682_gene;inference=ab initio prediction:Prodigal:002006;locus_tag=M1404_00682;product=hypothetical protein;protein_id=gnl|Prokka|M1404_00682

gnl|Prokka|M1404_1 prokka gene 744107 744676 . - . ID=M1404_00683_gene;Name=wbnI;gene=wbnI;locus_tag=M1404_00683

gnl|Prokka|M1404_1 Prodigal:002006 CDS 744107 744676 . - 0 ID=M1404_00683;Parent=M1404_00683_gene;eC_number=2.4.1.309;Name=wbnI;gene=wbnI;inference=ab initio prediction:Prodigal:002006,similar to AA sequence:UniProtKB:Q5JBG6;locus_tag=M1404_00683;product=O-antigen biosynthesis glycosyltransferase WbnI;protein_id=gnl|Prokka|M1404_00683

gnl|Prokka|M1404_1 prokka gene 744710 746185 . - . ID=M1404_00684_gene;Name=rng;gene=rng;locus_tag=M1404_00684

gnl|Prokka|M1404_1 Prodigal:002006 CDS 744710 746185 . - 0 ID=M1404_00684;Parent=M1404_00684_gene;eC_number=3.1.26.-;Name=rng;db_xref=COG:COG1530;gene=rng;inference=ab initio prediction:Prodigal:002006,similar to AA sequence:UniProtKB:P0A9J0;locus_tag=M1404_00684;product=Ribonuclease G;protein_id=gnl|Prokka|M1404_00684

gnl|Prokka|M1404_1 prokka gene 746340 747854 . + . ID=M1404_00685_gene;Name=putP_2;gene=putP_2;locus_tag=M1404_00685

gnl|Prokka|M1404_1 Prodigal:002006 CDS 746340 747854 . + 0 ID=M1404_00685;Parent=M1404_00685_gene;Name=putP_2;db_xref=COG:COG0591;gene=putP_2;inference=ab initio prediction:Prodigal:002006,similar to AA sequence:UniProtKB:P07117;locus_tag=M1404_00685;product=Sodium/proline symporter;protein_id=gnl|Prokka|M1404_00685

gnl|Prokka|M1404_1 prokka gene 747851 748816 . + . ID=M1404_00686_gene;Name=cmoB;gene=cmoB;locus_tag=M1404_00686

gnl|Prokka|M1404_1 Prodigal:002006 CDS 747851 748816 . + 0 ID=M1404_00686;Parent=M1404_00686_gene;eC_number=2.5.1.-;Name=cmoB;db_xref=COG:COG0500;gene=cmoB;inference=ab initio prediction:Prodigal:002006,similar to AA sequence:UniProtKB:P44167;locus_tag=M1404_00686;product=tRNA U34 carboxymethyltransferase;protein_id=gnl|Prokka|M1404_00686

gnl|Prokka|M1404_1 prokka gene 748950 750170 . - . ID=M1404_00687_gene;Name=fabB;gene=fabB;locus_tag=M1404_00687

gnl|Prokka|M1404_1 Prodigal:002006 CDS 748950 750170 . - 0 ID=M1404_00687;Parent=M1404_00687_gene;eC_number=2.3.1.41;Name=fabB;db_xref=COG:COG0304;gene=fabB;inference=ab initio prediction:Prodigal:002006,similar to AA sequence:UniProtKB:P0A953;locus_tag=M1404_00687;product=3-oxoacyl-[acyl-carrier-protein] synthase 1;protein_id=gnl|Prokka|M1404_00687

gnl|Prokka|M1404_1 prokka gene 750330 752348 . + . ID=M1404_00688_gene;Name=mnmC;gene=mnmC;locus_tag=M1404_00688

gnl|Prokka|M1404_1 Prodigal:002006 CDS 750330 752348 . + 0 ID=M1404_00688;Parent=M1404_00688_gene;Name=mnmC;db_xref=COG:COG0665;gene=mnmC;inference=ab initio prediction:Prodigal:002006,similar to AA sequence:UniProtKB:Q8ZD36;locus_tag=M1404_00688;product=tRNA 5-methylaminomethyl-2-thiouridine biosynthesis bifunctional protein MnmC;protein_id=gnl|Prokka|M1404_00688

gnl|Prokka|M1404_1 prokka gene 752667 755645 . + . ID=M1404_00689_gene;locus_tag=M1404_00689

gnl|Prokka|M1404_1 Prodigal:002006 CDS 752667 755645 . + 0 ID=M1404_00689;Parent=M1404_00689_gene;db_xref=COG:COG1629;inference=ab initio prediction:Prodigal:002006,similar to AA sequence:UniProtKB:P44795;locus_tag=M1404_00689;product=putative hemoglobin and hemoglobin-haptoglobin-binding protein 1;protein_id=gnl|Prokka|M1404_00689

gnl|Prokka|M1404_1 prokka gene 755933 759064 . + . ID=M1404_00690_gene;Name=hgpA;gene=hgpA;locus_tag=M1404_00690

gnl|Prokka|M1404_1 Prodigal:002006 CDS 755933 759064 . + 0 ID=M1404_00690;Parent=M1404_00690_gene;Name=hgpA;gene=hgpA;inference=ab initio prediction:Prodigal:002006,similar to AA sequence:UniProtKB:Q9ZA21;locus_tag=M1404_00690;product=Hemoglobin and hemoglobin-haptoglobin-binding protein A;protein_id=gnl|Prokka|M1404_00690

gnl|Prokka|M1404_1 prokka gene 759121 760458 . - . ID=M1404_00691_gene;Name=tcyP;gene=tcyP;locus_tag=M1404_00691

gnl|Prokka|M1404_1 Prodigal:002006 CDS 759121 760458 . - 0 ID=M1404_00691;Parent=M1404_00691_gene;Name=tcyP;db_xref=COG:COG1823;gene=tcyP;inference=ab initio prediction:Prodigal:002006,similar to AA sequence:UniProtKB:P54596;locus_tag=M1404_00691;product=L-cystine uptake protein TcyP;protein_id=gnl|Prokka|M1404_00691

gnl|Prokka|M1404_1 prokka gene 760639 761232 . - . ID=M1404_00692_gene;Name=grpE;gene=grpE;locus_tag=M1404_00692

gnl|Prokka|M1404_1 Prodigal:002006 CDS 760639 761232 . - 0 ID=M1404_00692;Parent=M1404_00692_gene;Name=grpE;db_xref=COG:COG0576;gene=grpE;inference=ab initio prediction:Prodigal:002006,similar to AA sequence:UniProtKB:P09372;locus_tag=M1404_00692;product=Protein GrpE;protein_id=gnl|Prokka|M1404_00692

gnl|Prokka|M1404_1 prokka gene 761395 762312 . + . ID=M1404_00693_gene;Name=nadK;gene=nadK;locus_tag=M1404_00693

gnl|Prokka|M1404_1 Prodigal:002006 CDS 761395 762312 . + 0 ID=M1404_00693;Parent=M1404_00693_gene;eC_number=2.7.1.23;Name=nadK;db_xref=COG:COG0061;gene=nadK;inference=ab initio prediction:Prodigal:002006,similar to AA sequence:UniProtKB:P44497;locus_tag=M1404_00693;product=NAD kinase;protein_id=gnl|Prokka|M1404_00693

gnl|Prokka|M1404_1 prokka gene 762397 764073 . + . ID=M1404_00694_gene;Name=recN;gene=recN;locus_tag=M1404_00694

gnl|Prokka|M1404_1 Prodigal:002006 CDS 762397 764073 . + 0 ID=M1404_00694;Parent=M1404_00694_gene;Name=recN;db_xref=COG:COG0497;gene=recN;inference=ab initio prediction:Prodigal:002006,similar to AA sequence:UniProtKB:P05824;locus_tag=M1404_00694;product=DNA repair protein RecN;protein_id=gnl|Prokka|M1404_00694

gnl|Prokka|M1404_1 prokka gene 764138 764752 . - . ID=M1404_00695_gene;Name=ompW;gene=ompW;locus_tag=M1404_00695

gnl|Prokka|M1404_1 Prodigal:002006 CDS 764138 764752 . - 0 ID=M1404_00695;Parent=M1404_00695_gene;Name=ompW;db_xref=COG:COG3047;gene=ompW;inference=ab initio prediction:Prodigal:002006,similar to AA sequence:UniProtKB:P0A915;locus_tag=M1404_00695;product=Outer membrane protein W;protein_id=gnl|Prokka|M1404_00695

gnl|Prokka|M1404_1 prokka gene 765019 765783 . + . ID=M1404_00696_gene;locus_tag=M1404_00696

gnl|Prokka|M1404_1 Prodigal:002006 CDS 765019 765783 . + 0 ID=M1404_00696;Parent=M1404_00696_gene;inference=ab initio prediction:Prodigal:002006;locus_tag=M1404_00696;product=hypothetical protein;protein_id=gnl|Prokka|M1404_00696

gnl|Prokka|M1404_1 prokka gene 765790 766344 . + . ID=M1404_00697_gene;Name=yciB;gene=yciB;locus_tag=M1404_00697

gnl|Prokka|M1404_1 Prodigal:002006 CDS 765790 766344 . + 0 ID=M1404_00697;Parent=M1404_00697_gene;Name=yciB;db_xref=COG:COG2917;gene=yciB;inference=ab initio prediction:Prodigal:002006,similar to AA sequence:UniProtKB:P0A710;locus_tag=M1404_00697;product=putative intracellular septation protein A;protein_id=gnl|Prokka|M1404_00697

gnl|Prokka|M1404_1 prokka gene 766347 766790 . + . ID=M1404_00698_gene;locus_tag=M1404_00698

gnl|Prokka|M1404_1 Prodigal:002006 CDS 766347 766790 . + 0 ID=M1404_00698;Parent=M1404_00698_gene;eC_number=3.1.2.-;db_xref=COG:COG1607;inference=ab initio prediction:Prodigal:002006,similar to AA sequence:UniProtKB:P44886;locus_tag=M1404_00698;product=putative acyl-CoA thioester hydrolase;protein_id=gnl|Prokka|M1404_00698

gnl|Prokka|M1404_1 prokka gene 766813 767112 . + . ID=M1404_00699_gene;locus_tag=M1404_00699

gnl|Prokka|M1404_1 Prodigal:002006 CDS 766813 767112 . + 0 ID=M1404_00699;Parent=M1404_00699_gene;db_xref=COG:COG2350;inference=ab initio prediction:Prodigal:002006,similar to AA sequence:UniProtKB:P44887;locus_tag=M1404_00699;product=putative protein;protein_id=gnl|Prokka|M1404_00699

gnl|Prokka|M1404_1 prokka gene 767256 767465 . + . ID=M1404_00700_gene;locus_tag=M1404_00700

gnl|Prokka|M1404_1 Prodigal:002006 CDS 767256 767465 . + 0 ID=M1404_00700;Parent=M1404_00700_gene;inference=ab initio prediction:Prodigal:002006;locus_tag=M1404_00700;product=hypothetical protein;protein_id=gnl|Prokka|M1404_00700

gnl|Prokka|M1404_1 prokka gene 767607 769298 . + . ID=M1404_00701_gene;Name=slt;gene=slt;locus_tag=M1404_00701

gnl|Prokka|M1404_1 Prodigal:002006 CDS 767607 769298 . + 0 ID=M1404_00701;Parent=M1404_00701_gene;eC_number=4.2.2.-;Name=slt;db_xref=COG:COG0741;gene=slt;inference=ab initio prediction:Prodigal:002006,similar to AA sequence:UniProtKB:P0AGC3;locus_tag=M1404_00701;product=Soluble lytic murein transglycosylase;protein_id=gnl|Prokka|M1404_00701

gnl|Prokka|M1404_1 prokka gene 769325 769633 . + . ID=M1404_00702_gene;Name=trpR;gene=trpR;locus_tag=M1404_00702

gnl|Prokka|M1404_1 Prodigal:002006 CDS 769325 769633 . + 0 ID=M1404_00702;Parent=M1404_00702_gene;Name=trpR;db_xref=COG:COG2973;gene=trpR;inference=ab initio prediction:Prodigal:002006,similar to AA sequence:UniProtKB:P0A881;locus_tag=M1404_00702;product=Trp operon repressor;protein_id=gnl|Prokka|M1404_00702

gnl|Prokka|M1404_1 prokka gene 769767 770351 . + . ID=M1404_00703_gene;Name=mtgA;gene=mtgA;locus_tag=M1404_00703

gnl|Prokka|M1404_1 Prodigal:002006 CDS 769767 770351 . + 0 ID=M1404_00703;Parent=M1404_00703_gene;eC_number=2.4.1.129;Name=mtgA;db_xref=COG:COG0744;gene=mtgA;inference=ab initio prediction:Prodigal:002006,similar to AA sequence:UniProtKB:P46022;locus_tag=M1404_00703;product=Biosynthetic peptidoglycan transglycosylase;protein_id=gnl|Prokka|M1404_00703

gnl|Prokka|M1404_1 prokka gene 770427 770762 . - . ID=M1404_00704_gene;Name=fdx;gene=fdx;locus_tag=M1404_00704

gnl|Prokka|M1404_1 Prodigal:002006 CDS 770427 770762 . - 0 ID=M1404_00704;Parent=M1404_00704_gene;Name=fdx;db_xref=COG:COG0633;gene=fdx;inference=ab initio prediction:Prodigal:002006,similar to AA sequence:UniProtKB:P0A9R4;locus_tag=M1404_00704;product=2Fe-2S ferredoxin;protein_id=gnl|Prokka|M1404_00704

gnl|Prokka|M1404_1 prokka gene 770783 772645 . - . ID=M1404_00705_gene;Name=hscA;gene=hscA;locus_tag=M1404_00705

gnl|Prokka|M1404_1 Prodigal:002006 CDS 770783 772645 . - 0 ID=M1404_00705;Parent=M1404_00705_gene;Name=hscA;db_xref=COG:COG0443;gene=hscA;inference=ab initio prediction:Prodigal:002006,similar to AA sequence:UniProtKB:P0A6Z1;locus_tag=M1404_00705;product=Chaperone protein HscA;protein_id=gnl|Prokka|M1404_00705

gnl|Prokka|M1404_1 prokka gene 772723 773241 . - . ID=M1404_00706_gene;Name=hscB;gene=hscB;locus_tag=M1404_00706

gnl|Prokka|M1404_1 Prodigal:002006 CDS 772723 773241 . - 0 ID=M1404_00706;Parent=M1404_00706_gene;Name=hscB;db_xref=COG:COG1076;gene=hscB;inference=ab initio prediction:Prodigal:002006,similar to AA sequence:UniProtKB:Q9KTX9;locus_tag=M1404_00706;product=Co-chaperone protein HscB ;protein_id=gnl|Prokka|M1404_00706

gnl|Prokka|M1404_1 prokka gene 773272 773595 . - . ID=M1404_00707_gene;Name=iscA;gene=iscA;locus_tag=M1404_00707

gnl|Prokka|M1404_1 Prodigal:002006 CDS 773272 773595 . - 0 ID=M1404_00707;Parent=M1404_00707_gene;Name=iscA;db_xref=COG:COG0316;gene=iscA;inference=ab initio prediction:Prodigal:002006,similar to AA sequence:UniProtKB:P0AAC8;locus_tag=M1404_00707;product=Iron-binding protein IscA;protein_id=gnl|Prokka|M1404_00707

gnl|Prokka|M1404_1 prokka gene 773665 774051 . - . ID=M1404_00708_gene;Name=iscU;gene=iscU;locus_tag=M1404_00708

gnl|Prokka|M1404_1 Prodigal:002006 CDS 773665 774051 . - 0 ID=M1404_00708;Parent=M1404_00708_gene;Name=iscU;db_xref=COG:COG0822;gene=iscU;inference=ab initio prediction:Prodigal:002006,similar to AA sequence:UniProtKB:Q57074;locus_tag=M1404_00708;product=Iron-sulfur cluster assembly scaffold protein IscU;protein_id=gnl|Prokka|M1404_00708

gnl|Prokka|M1404_1 prokka gene 774283 775497 . - . ID=M1404_00709_gene;Name=iscS;gene=iscS;locus_tag=M1404_00709

gnl|Prokka|M1404_1 Prodigal:002006 CDS 774283 775497 . - 0 ID=M1404_00709;Parent=M1404_00709_gene;eC_number=2.8.1.7;Name=iscS;db_xref=COG:COG1104;gene=iscS;inference=ab initio prediction:Prodigal:002006,similar to AA sequence:UniProtKB:P0A6B7;locus_tag=M1404_00709;product=Cysteine desulfurase IscS;protein_id=gnl|Prokka|M1404_00709

gnl|Prokka|M1404_1 prokka gene 775545 776009 . - . ID=M1404_00710_gene;Name=iscR;gene=iscR;locus_tag=M1404_00710

gnl|Prokka|M1404_1 Prodigal:002006 CDS 775545 776009 . - 0 ID=M1404_00710;Parent=M1404_00710_gene;Name=iscR;db_xref=COG:COG1959;gene=iscR;inference=ab initio prediction:Prodigal:002006,similar to AA sequence:UniProtKB:P0AGK8;locus_tag=M1404_00710;product=HTH-type transcriptional regulator IscR;protein_id=gnl|Prokka|M1404_00710

gnl|Prokka|M1404_1 prokka gene 776088 776804 . - . ID=M1404_00711_gene;locus_tag=M1404_00711

gnl|Prokka|M1404_1 Prodigal:002006 CDS 776088 776804 . - 0 ID=M1404_00711;Parent=M1404_00711_gene;eC_number=2.1.1.-;db_xref=COG:COG0565;inference=ab initio prediction:Prodigal:002006,similar to AA sequence:UniProtKB:P44676;locus_tag=M1404_00711;product=putative tRNA/rRNA methyltransferase;protein_id=gnl|Prokka|M1404_00711

gnl|Prokka|M1404_1 prokka gene 777018 777821 . + . ID=M1404_00712_gene;Name=suhB;gene=suhB;locus_tag=M1404_00712

gnl|Prokka|M1404_1 Prodigal:002006 CDS 777018 777821 . + 0 ID=M1404_00712;Parent=M1404_00712_gene;eC_number=3.1.3.25;Name=suhB;db_xref=COG:COG0483;gene=suhB;inference=ab initio prediction:Prodigal:002006,similar to AA sequence:UniProtKB:P0ADG4;locus_tag=M1404_00712;product=Inositol-1-monophosphatase;protein_id=gnl|Prokka|M1404_00712

gnl|Prokka|M1404_1 prokka gene 777885 778475 . - . ID=M1404_00713_gene;locus_tag=M1404_00713

gnl|Prokka|M1404_1 Prodigal:002006 CDS 777885 778475 . - 0 ID=M1404_00713;Parent=M1404_00713_gene;inference=ab initio prediction:Prodigal:002006;locus_tag=M1404_00713;product=hypothetical protein;protein_id=gnl|Prokka|M1404_00713

gnl|Prokka|M1404_1 prokka gene 778616 780472 . - . ID=M1404_00714_gene;Name=arcB;gene=arcB;locus_tag=M1404_00714

gnl|Prokka|M1404_1 Prodigal:002006 CDS 778616 780472 . - 0 ID=M1404_00714;Parent=M1404_00714_gene;eC_number=2.7.13.3;Name=arcB;db_xref=COG:COG0642;gene=arcB;inference=ab initio prediction:Prodigal:002006,similar to AA sequence:UniProtKB:P0AEC3;locus_tag=M1404_00714;product=Aerobic respiration control sensor protein ArcB;protein_id=gnl|Prokka|M1404_00714

gnl|Prokka|M1404_1 prokka gene 780530 780865 . - . ID=M1404_00715_gene;locus_tag=M1404_00715

gnl|Prokka|M1404_1 Prodigal:002006 CDS 780530 780865 . - 0 ID=M1404_00715;Parent=M1404_00715_gene;inference=ab initio prediction:Prodigal:002006;locus_tag=M1404_00715;product=hypothetical protein;protein_id=gnl|Prokka|M1404_00715

gnl|Prokka|M1404_1 prokka gene 780908 783850 . - . ID=M1404_00716_gene;Name=cas3;gene=cas3;locus_tag=M1404_00716

gnl|Prokka|M1404_1 Prodigal:002006 CDS 780908 783850 . - 0 ID=M1404_00716;Parent=M1404_00716_gene;eC_number=3.1.-.-;Name=cas3;gene=cas3;inference=ab initio prediction:Prodigal:002006,similar to AA sequence:UniProtKB:Q02ML8;locus_tag=M1404_00716;product=CRISPR-associated nuclease/helicase Cas3 subtype I-F/YPEST;protein_id=gnl|Prokka|M1404_00716

gnl|Prokka|M1404_1 prokka gene 783831 784811 . - . ID=M1404_00717_gene;Name=cas1_2;gene=cas1_2;locus_tag=M1404_00717

gnl|Prokka|M1404_1 Prodigal:002006 CDS 783831 784811 . - 0 ID=M1404_00717;Parent=M1404_00717_gene;eC_number=3.1.-.-;Name=cas1_2;db_xref=COG:COG1518;gene=cas1_2;inference=ab initio prediction:Prodigal:002006,similar to AA sequence:UniProtKB:Q6D0X0;locus_tag=M1404_00717;product=CRISPR-associated endonuclease Cas1;protein_id=gnl|Prokka|M1404_00717

gnl|Prokka|M1404_1 minced:0.4.2 repeat_region 784981 785309 . . . note=CRISPR with 6 repeat units;rpt_family=CRISPR;rpt_type=direct;rpt_unit_seq=TTTCTAAGCCATCTACACGATGGTGAAC

gnl|Prokka|M1404_1 prokka gene 785451 786011 . - . ID=M1404_00718_gene;Name=cas6f;gene=cas6f;locus_tag=M1404_00718

gnl|Prokka|M1404_1 Prodigal:002006 CDS 785451 786011 . - 0 ID=M1404_00718;Parent=M1404_00718_gene;eC_number=3.1.-.-;Name=cas6f;gene=cas6f;inference=ab initio prediction:Prodigal:002006,similar to AA sequence:UniProtKB:Q6D0W5;locus_tag=M1404_00718;product=CRISPR-associated endonuclease Cas6f/Csy4;protein_id=gnl|Prokka|M1404_00718

gnl|Prokka|M1404_1 prokka gene 786014 787021 . - . ID=M1404_00719_gene;Name=csy3;gene=csy3;locus_tag=M1404_00719

gnl|Prokka|M1404_1 Prodigal:002006 CDS 786014 787021 . - 0 ID=M1404_00719;Parent=M1404_00719_gene;Name=csy3;gene=csy3;inference=ab initio prediction:Prodigal:002006,similar to AA sequence:UniProtKB:Q6D0W6;locus_tag=M1404_00719;product=CRISPR-associated protein Csy3;protein_id=gnl|Prokka|M1404_00719

gnl|Prokka|M1404_1 prokka gene 787024 788007 . - . ID=M1404_00720_gene;Name=csy2;gene=csy2;locus_tag=M1404_00720

gnl|Prokka|M1404_1 Prodigal:002006 CDS 787024 788007 . - 0 ID=M1404_00720;Parent=M1404_00720_gene;Name=csy2;gene=csy2;inference=ab initio prediction:Prodigal:002006,similar to AA sequence:UniProtKB:Q02MM0;locus_tag=M1404_00720;product=CRISPR-associated protein Csy2;protein_id=gnl|Prokka|M1404_00720

gnl|Prokka|M1404_1 prokka gene 788017 789363 . - . ID=M1404_00721_gene;Name=csy1;gene=csy1;locus_tag=M1404_00721

gnl|Prokka|M1404_1 Prodigal:002006 CDS 788017 789363 . - 0 ID=M1404_00721;Parent=M1404_00721_gene;Name=csy1;gene=csy1;inference=ab initio prediction:Prodigal:002006,similar to AA sequence:UniProtKB:Q6D0W8;locus_tag=M1404_00721;product=CRISPR-associated protein Csy1;protein_id=gnl|Prokka|M1404_00721

gnl|Prokka|M1404_1 prokka gene 789653 791812 . - . ID=M1404_00722_gene;Name=rlmL;gene=rlmL;locus_tag=M1404_00722

gnl|Prokka|M1404_1 Prodigal:002006 CDS 789653 791812 . - 0 ID=M1404_00722;Parent=M1404_00722_gene;Name=rlmL;db_xref=COG:COG0116;gene=rlmL;inference=ab initio prediction:Prodigal:002006,similar to AA sequence:UniProtKB:P75864;locus_tag=M1404_00722;product=Ribosomal RNA large subunit methyltransferase K/L;protein_id=gnl|Prokka|M1404_00722

gnl|Prokka|M1404_1 prokka gene 791946 793994 . - . ID=M1404_00723_gene;Name=metG;gene=metG;locus_tag=M1404_00723

gnl|Prokka|M1404_1 Prodigal:002006 CDS 791946 793994 . - 0 ID=M1404_00723;Parent=M1404_00723_gene;eC_number=6.1.1.10;Name=metG;db_xref=COG:COG0073;gene=metG;inference=ab initio prediction:Prodigal:002006,similar to AA sequence:UniProtKB:P00959;locus_tag=M1404_00723;product=Methionine--tRNA ligase;protein_id=gnl|Prokka|M1404_00723

gnl|Prokka|M1404_1 prokka gene 794176 795288 . + . ID=M1404_00724_gene;Name=apbC;gene=apbC;locus_tag=M1404_00724

gnl|Prokka|M1404_1 Prodigal:002006 CDS 794176 795288 . + 0 ID=M1404_00724;Parent=M1404_00724_gene;Name=apbC;db_xref=COG:COG0489;gene=apbC;inference=ab initio prediction:Prodigal:002006,similar to AA sequence:UniProtKB:Q8ZNN5;locus_tag=M1404_00724;product=Iron-sulfur cluster carrier protein;protein_id=gnl|Prokka|M1404_00724

gnl|Prokka|M1404_1 prokka gene 795425 795694 . - . ID=M1404_00725_gene;Name=rpsO;gene=rpsO;locus_tag=M1404_00725

gnl|Prokka|M1404_1 Prodigal:002006 CDS 795425 795694 . - 0 ID=M1404_00725;Parent=M1404_00725_gene;Name=rpsO;db_xref=COG:COG0184;gene=rpsO;inference=ab initio prediction:Prodigal:002006,similar to AA sequence:UniProtKB:P0ADZ4;locus_tag=M1404_00725;product=30S ribosomal protein S15;protein_id=gnl|Prokka|M1404_00725

gnl|Prokka|M1404_1 prokka gene 795861 798776 . - . ID=M1404_00726_gene;locus_tag=M1404_00726

gnl|Prokka|M1404_1 Prodigal:002006 CDS 795861 798776 . - 0 ID=M1404_00726;Parent=M1404_00726_gene;db_xref=COG:COG1629;inference=ab initio prediction:Prodigal:002006,similar to AA sequence:UniProtKB:P44809;locus_tag=M1404_00726;product=putative hemoglobin and hemoglobin-haptoglobin-binding protein 2;protein_id=gnl|Prokka|M1404_00726

gnl|Prokka|M1404_1 prokka gene 798818 799333 . - . ID=M1404_00727_gene;Name=hutZ_1;gene=hutZ_1;locus_tag=M1404_00727

gnl|Prokka|M1404_1 Prodigal:002006 CDS 798818 799333 . - 0 ID=M1404_00727;Parent=M1404_00727_gene;eC_number=1.14.99.58;Name=hutZ_1;db_xref=COG:COG0748;gene=hutZ_1;inference=ab initio prediction:Prodigal:002006,similar to AA sequence:UniProtKB:Q9KL41;locus_tag=M1404_00727;product=Heme oxygenase HutZ;protein_id=gnl|Prokka|M1404_00727

gnl|Prokka|M1404_1 prokka gene 799349 799840 . - . ID=M1404_00728_gene;Name=hutX;gene=hutX;locus_tag=M1404_00728

gnl|Prokka|M1404_1 Prodigal:002006 CDS 799349 799840 . - 0 ID=M1404_00728;Parent=M1404_00728_gene;Name=hutX;db_xref=COG:COG3721;gene=hutX;inference=ab initio prediction:Prodigal:002006,similar to AA sequence:UniProtKB:Q9KL40;locus_tag=M1404_00728;product=Intracellular heme transport protein HutX;protein_id=gnl|Prokka|M1404_00728

gnl|Prokka|M1404_1 prokka gene 800152 800236 . + . ID=M1404_00729_gene;locus_tag=M1404_00729

gnl|Prokka|M1404_1 Aragorn:001002 tRNA 800152 800236 . + . ID=M1404_00729;Parent=M1404_00729_gene;inference=COORDINATES:profile:Aragorn:001002;locus_tag=M1404_00729;product=tRNA-Leu(caa)

gnl|Prokka|M1404_1 prokka gene 800497 801168 . + . ID=M1404_00730_gene;locus_tag=M1404_00730

gnl|Prokka|M1404_1 Prodigal:002006 CDS 800497 801168 . + 0 ID=M1404_00730;Parent=M1404_00730_gene;inference=ab initio prediction:Prodigal:002006;locus_tag=M1404_00730;product=hypothetical protein;protein_id=gnl|Prokka|M1404_00730

gnl|Prokka|M1404_1 prokka gene 801338 801448 . - . ID=M1404_00731_gene;locus_tag=M1404_00731

gnl|Prokka|M1404_1 barrnap:0.9 rRNA 801338 801448 3.6e-09 - . ID=M1404_00731;Parent=M1404_00731_gene;locus_tag=M1404_00731;product=5S ribosomal RNA

gnl|Prokka|M1404_1 prokka gene 801616 804515 . - . ID=M1404_00732_gene;locus_tag=M1404_00732

gnl|Prokka|M1404_1 barrnap:0.9 rRNA 801616 804515 0 - . ID=M1404_00732;Parent=M1404_00732_gene;locus_tag=M1404_00732;product=23S ribosomal RNA

gnl|Prokka|M1404_1 prokka gene 804838 804913 . - . ID=M1404_00733_gene;locus_tag=M1404_00733

gnl|Prokka|M1404_1 Aragorn:001002 tRNA 804838 804913 . - . ID=M1404_00733;Parent=M1404_00733_gene;inference=COORDINATES:profile:Aragorn:001002;locus_tag=M1404_00733;product=tRNA-Ala(tgc)

gnl|Prokka|M1404_1 prokka gene 804975 805051 . - . ID=M1404_00734_gene;locus_tag=M1404_00734

gnl|Prokka|M1404_1 Aragorn:001002 tRNA 804975 805051 . - . ID=M1404_00734;Parent=M1404_00734_gene;inference=COORDINATES:profile:Aragorn:001002;locus_tag=M1404_00734;product=tRNA-Ile(gat)

gnl|Prokka|M1404_1 prokka gene 805136 806673 . - . ID=M1404_00735_gene;locus_tag=M1404_00735

gnl|Prokka|M1404_1 barrnap:0.9 rRNA 805136 806673 0 - . ID=M1404_00735;Parent=M1404_00735_gene;locus_tag=M1404_00735;product=16S ribosomal RNA

gnl|Prokka|M1404_1 prokka gene 807066 807911 . + . ID=M1404_00736_gene;locus_tag=M1404_00736

gnl|Prokka|M1404_1 Prodigal:002006 CDS 807066 807911 . + 0 ID=M1404_00736;Parent=M1404_00736_gene;inference=ab initio prediction:Prodigal:002006;locus_tag=M1404_00736;product=hypothetical protein;protein_id=gnl|Prokka|M1404_00736

gnl|Prokka|M1404_1 prokka gene 807974 808912 . - . ID=M1404_00737_gene;Name=birA;gene=birA;locus_tag=M1404_00737

gnl|Prokka|M1404_1 Prodigal:002006 CDS 807974 808912 . - 0 ID=M1404_00737;Parent=M1404_00737_gene;eC_number=6.3.4.15;Name=birA;db_xref=COG:COG0340;gene=birA;inference=ab initio prediction:Prodigal:002006,similar to AA sequence:UniProtKB:P06709;locus_tag=M1404_00737;product=Bifunctional ligase/repressor BirA;protein_id=gnl|Prokka|M1404_00737

gnl|Prokka|M1404_1 prokka gene 809080 810543 . + . ID=M1404_00738_gene;Name=guaB;gene=guaB;locus_tag=M1404_00738

gnl|Prokka|M1404_1 Prodigal:002006 CDS 809080 810543 . + 0 ID=M1404_00738;Parent=M1404_00738_gene;eC_number=1.1.1.205;Name=guaB;db_xref=COG:COG0516;gene=guaB;inference=ab initio prediction:Prodigal:002006,similar to AA sequence:UniProtKB:P0ADG7;locus_tag=M1404_00738;product=Inosine-5'-monophosphate dehydrogenase;protein_id=gnl|Prokka|M1404_00738

gnl|Prokka|M1404_1 prokka gene 810617 811888 . + . ID=M1404_00739_gene;locus_tag=M1404_00739

gnl|Prokka|M1404_1 Prodigal:002006 CDS 810617 811888 . + 0 ID=M1404_00739;Parent=M1404_00739_gene;inference=ab initio prediction:Prodigal:002006;locus_tag=M1404_00739;product=hypothetical protein;protein_id=gnl|Prokka|M1404_00739

gnl|Prokka|M1404_1 prokka gene 811997 813568 . + . ID=M1404_00740_gene;Name=guaA;gene=guaA;locus_tag=M1404_00740

gnl|Prokka|M1404_1 Prodigal:002006 CDS 811997 813568 . + 0 ID=M1404_00740;Parent=M1404_00740_gene;eC_number=6.3.5.2;Name=guaA;db_xref=COG:COG0518;gene=guaA;inference=ab initio prediction:Prodigal:002006,similar to AA sequence:UniProtKB:P04079;locus_tag=M1404_00740;product=GMP synthase [glutamine-hydrolyzing];protein_id=gnl|Prokka|M1404_00740

gnl|Prokka|M1404_1 prokka gene 813637 813990 . - . ID=M1404_00741_gene;locus_tag=M1404_00741

gnl|Prokka|M1404_1 Prodigal:002006 CDS 813637 813990 . - 0 ID=M1404_00741;Parent=M1404_00741_gene;inference=ab initio prediction:Prodigal:002006;locus_tag=M1404_00741;product=hypothetical protein;protein_id=gnl|Prokka|M1404_00741

gnl|Prokka|M1404_1 prokka gene 814273 815226 . + . ID=M1404_00742_gene;Name=accA;gene=accA;locus_tag=M1404_00742

gnl|Prokka|M1404_1 Prodigal:002006 CDS 814273 815226 . + 0 ID=M1404_00742;Parent=M1404_00742_gene;eC_number=2.1.3.15;Name=accA;db_xref=COG:COG0825;gene=accA;inference=ab initio prediction:Prodigal:002006,similar to AA sequence:UniProtKB:P0ABD5;locus_tag=M1404_00742;product=Acetyl-coenzyme A carboxylase carboxyl transferase subunit alpha;protein_id=gnl|Prokka|M1404_00742

gnl|Prokka|M1404_1 prokka gene 815278 815994 . + . ID=M1404_00743_gene;locus_tag=M1404_00743

gnl|Prokka|M1404_1 Prodigal:002006 CDS 815278 815994 . + 0 ID=M1404_00743;Parent=M1404_00743_gene;inference=ab initio prediction:Prodigal:002006;locus_tag=M1404_00743;product=hypothetical protein;protein_id=gnl|Prokka|M1404_00743

gnl|Prokka|M1404_1 prokka gene 815996 816856 . + . ID=M1404_00744_gene;Name=pdxY;gene=pdxY;locus_tag=M1404_00744

gnl|Prokka|M1404_1 Prodigal:002006 CDS 815996 816856 . + 0 ID=M1404_00744;Parent=M1404_00744_gene;eC_number=2.7.1.35;Name=pdxY;db_xref=COG:COG2240;gene=pdxY;inference=ab initio prediction:Prodigal:002006,similar to AA sequence:UniProtKB:Q7CIR8;locus_tag=M1404_00744;product=Pyridoxal kinase PdxY;protein_id=gnl|Prokka|M1404_00744

gnl|Prokka|M1404_1 prokka gene 816912 818207 . + . ID=M1404_00745_gene;Name=tilS;gene=tilS;locus_tag=M1404_00745

gnl|Prokka|M1404_1 Prodigal:002006 CDS 816912 818207 . + 0 ID=M1404_00745;Parent=M1404_00745_gene;eC_number=6.3.4.19;Name=tilS;db_xref=COG:COG0037;gene=tilS;inference=ab initio prediction:Prodigal:002006,similar to AA sequence:UniProtKB:P52097;locus_tag=M1404_00745;product=tRNA(Ile)-lysidine synthase;protein_id=gnl|Prokka|M1404_00745

gnl|Prokka|M1404_1 prokka gene 818444 819610 . + . ID=M1404_00746_gene;Name=lldD;gene=lldD;locus_tag=M1404_00746

gnl|Prokka|M1404_1 Prodigal:002006 CDS 818444 819610 . + 0 ID=M1404_00746;Parent=M1404_00746_gene;eC_number=1.1.-.-;Name=lldD;db_xref=COG:COG1304;gene=lldD;inference=ab initio prediction:Prodigal:002006,similar to AA sequence:UniProtKB:P33232;locus_tag=M1404_00746;product=L-lactate dehydrogenase;protein_id=gnl|Prokka|M1404_00746

gnl|Prokka|M1404_1 prokka gene 819721 821034 . - . ID=M1404_00747_gene;Name=ghxP;gene=ghxP;locus_tag=M1404_00747

gnl|Prokka|M1404_1 Prodigal:002006 CDS 819721 821034 . - 0 ID=M1404_00747;Parent=M1404_00747_gene;Name=ghxP;db_xref=COG:COG2252;gene=ghxP;inference=ab initio prediction:Prodigal:002006,similar to AA sequence:UniProtKB:P0AF52;locus_tag=M1404_00747;product=Guanine/hypoxanthine permease GhxP;protein_id=gnl|Prokka|M1404_00747

gnl|Prokka|M1404_1 prokka gene 821301 822317 . + . ID=M1404_00748_gene;Name=lnpD;gene=lnpD;locus_tag=M1404_00748

gnl|Prokka|M1404_1 Prodigal:002006 CDS 821301 822317 . + 0 ID=M1404_00748;Parent=M1404_00748_gene;eC_number=5.1.3.2;Name=lnpD;gene=lnpD;inference=ab initio prediction:Prodigal:002006,similar to AA sequence:UniProtKB:E8MF10;locus_tag=M1404_00748;product=UDP-glucose 4-epimerase;protein_id=gnl|Prokka|M1404_00748

gnl|Prokka|M1404_1 prokka gene 822320 823597 . + . ID=M1404_00749_gene;Name=ampG;gene=ampG;locus_tag=M1404_00749

gnl|Prokka|M1404_1 Prodigal:002006 CDS 822320 823597 . + 0 ID=M1404_00749;Parent=M1404_00749_gene;Name=ampG;db_xref=COG:COG0477;gene=ampG;inference=ab initio prediction:Prodigal:002006,similar to AA sequence:UniProtKB:P0AE16;locus_tag=M1404_00749;product=Anhydromuropeptide permease;protein_id=gnl|Prokka|M1404_00749

gnl|Prokka|M1404_1 prokka gene 823746 824390 . + . ID=M1404_00750_gene;Name=adk;gene=adk;locus_tag=M1404_00750

gnl|Prokka|M1404_1 Prodigal:002006 CDS 823746 824390 . + 0 ID=M1404_00750;Parent=M1404_00750_gene;eC_number=2.7.4.3;Name=adk;db_xref=COG:COG0563;gene=adk;inference=ab initio prediction:Prodigal:002006,similar to AA sequence:UniProtKB:Q6LTE1;locus_tag=M1404_00750;product=Adenylate kinase;protein_id=gnl|Prokka|M1404_00750

gnl|Prokka|M1404_1 prokka gene 824458 825327 . - . ID=M1404_00751_gene;Name=sucD;gene=sucD;locus_tag=M1404_00751

gnl|Prokka|M1404_1 Prodigal:002006 CDS 824458 825327 . - 0 ID=M1404_00751;Parent=M1404_00751_gene;eC_number=6.2.1.5;Name=sucD;db_xref=COG:COG0074;gene=sucD;inference=ab initio prediction:Prodigal:002006,similar to AA sequence:UniProtKB:P0AGE9;locus_tag=M1404_00751;product=Succinate--CoA ligase [ADP-forming] subunit alpha;protein_id=gnl|Prokka|M1404_00751

gnl|Prokka|M1404_1 prokka gene 825329 826495 . - . ID=M1404_00752_gene;Name=sucC;gene=sucC;locus_tag=M1404_00752

gnl|Prokka|M1404_1 Prodigal:002006 CDS 825329 826495 . - 0 ID=M1404_00752;Parent=M1404_00752_gene;eC_number=6.2.1.5;Name=sucC;db_xref=COG:COG0045;gene=sucC;inference=ab initio prediction:Prodigal:002006,similar to AA sequence:UniProtKB:P53593;locus_tag=M1404_00752;product=Succinate--CoA ligase [ADP-forming] subunit beta;protein_id=gnl|Prokka|M1404_00752

gnl|Prokka|M1404_1 prokka gene 826600 826980 . - . ID=M1404_00753_gene;locus_tag=M1404_00753

gnl|Prokka|M1404_1 Prodigal:002006 CDS 826600 826980 . - 0 ID=M1404_00753;Parent=M1404_00753_gene;inference=ab initio prediction:Prodigal:002006;locus_tag=M1404_00753;product=hypothetical protein;protein_id=gnl|Prokka|M1404_00753

gnl|Prokka|M1404_1 prokka gene 827074 828288 . - . ID=M1404_00754_gene;Name=sucB;gene=sucB;locus_tag=M1404_00754

gnl|Prokka|M1404_1 Prodigal:002006 CDS 827074 828288 . - 0 ID=M1404_00754;Parent=M1404_00754_gene;eC_number=2.3.1.61;Name=sucB;db_xref=COG:COG0508;gene=sucB;inference=ab initio prediction:Prodigal:002006,similar to AA sequence:UniProtKB:P0AFG6;locus_tag=M1404_00754;product=Dihydrolipoyllysine-residue succinyltransferase component of 2-oxoglutarate dehydrogenase complex;protein_id=gnl|Prokka|M1404_00754

gnl|Prokka|M1404_1 prokka gene 828363 831158 . - . ID=M1404_00755_gene;Name=sucA;gene=sucA;locus_tag=M1404_00755

gnl|Prokka|M1404_1 Prodigal:002006 CDS 828363 831158 . - 0 ID=M1404_00755;Parent=M1404_00755_gene;eC_number=1.2.4.2;Name=sucA;db_xref=COG:COG0567;gene=sucA;inference=ab initio prediction:Prodigal:002006,similar to AA sequence:UniProtKB:P0AFG3;locus_tag=M1404_00755;product=2-oxoglutarate dehydrogenase E1 component;protein_id=gnl|Prokka|M1404_00755

gnl|Prokka|M1404_1 prokka gene 831476 832762 . + . ID=M1404_00756_gene;Name=gltA;gene=gltA;locus_tag=M1404_00756

gnl|Prokka|M1404_1 Prodigal:002006 CDS 831476 832762 . + 0 ID=M1404_00756;Parent=M1404_00756_gene;eC_number=2.3.3.16;Name=gltA;db_xref=COG:COG0372;gene=gltA;inference=ab initio prediction:Prodigal:002006,similar to AA sequence:UniProtKB:P0ABH7;locus_tag=M1404_00756;product=Citrate synthase;protein_id=gnl|Prokka|M1404_00756

gnl|Prokka|M1404_1 prokka gene 833019 834017 . + . ID=M1404_00757_gene;Name=dctP;gene=dctP;locus_tag=M1404_00757

gnl|Prokka|M1404_1 Prodigal:002006 CDS 833019 834017 . + 0 ID=M1404_00757;Parent=M1404_00757_gene;Name=dctP;db_xref=COG:COG1638;gene=dctP;inference=ab initio prediction:Prodigal:002006,similar to AA sequence:UniProtKB:Q9HU18;locus_tag=M1404_00757;product=C4-dicarboxylate-binding periplasmic protein DctP;protein_id=gnl|Prokka|M1404_00757

gnl|Prokka|M1404_1 prokka gene 834088 834714 . + . ID=M1404_00758_gene;Name=dctQ;gene=dctQ;locus_tag=M1404_00758

gnl|Prokka|M1404_1 Prodigal:002006 CDS 834088 834714 . + 0 ID=M1404_00758;Parent=M1404_00758_gene;Name=dctQ;db_xref=COG:COG3090;gene=dctQ;inference=ab initio prediction:Prodigal:002006,similar to AA sequence:UniProtKB:Q9HU17;locus_tag=M1404_00758;product=C4-dicarboxylate TRAP transporter small permease protein DctQ;protein_id=gnl|Prokka|M1404_00758

gnl|Prokka|M1404_1 prokka gene 834711 835994 . + . ID=M1404_00759_gene;Name=dctM_1;gene=dctM_1;locus_tag=M1404_00759

gnl|Prokka|M1404_1 Prodigal:002006 CDS 834711 835994 . + 0 ID=M1404_00759;Parent=M1404_00759_gene;Name=dctM_1;db_xref=COG:COG1593;gene=dctM_1;inference=ab initio prediction:Prodigal:002006,similar to AA sequence:UniProtKB:Q9HU16;locus_tag=M1404_00759;product=C4-dicarboxylate TRAP transporter large permease protein DctM;protein_id=gnl|Prokka|M1404_00759

gnl|Prokka|M1404_1 prokka gene 836052 836681 . - . ID=M1404_00760_gene;Name=gloC;gene=gloC;locus_tag=M1404_00760

gnl|Prokka|M1404_1 Prodigal:002006 CDS 836052 836681 . - 0 ID=M1404_00760;Parent=M1404_00760_gene;eC_number=3.1.2.6;Name=gloC;db_xref=COG:COG0491;gene=gloC;inference=ab initio prediction:Prodigal:002006,similar to AA sequence:UniProtKB:P75849;locus_tag=M1404_00760;product=Hydroxyacylglutathione hydrolase GloC;protein_id=gnl|Prokka|M1404_00760

gnl|Prokka|M1404_1 prokka gene 836754 837314 . - . ID=M1404_00761_gene;locus_tag=M1404_00761

gnl|Prokka|M1404_1 Prodigal:002006 CDS 836754 837314 . - 0 ID=M1404_00761;Parent=M1404_00761_gene;inference=ab initio prediction:Prodigal:002006;locus_tag=M1404_00761;product=hypothetical protein;protein_id=gnl|Prokka|M1404_00761

gnl|Prokka|M1404_1 prokka gene 837458 838960 . - . ID=M1404_00762_gene;locus_tag=M1404_00762

gnl|Prokka|M1404_1 Prodigal:002006 CDS 837458 838960 . - 0 ID=M1404_00762;Parent=M1404_00762_gene;inference=ab initio prediction:Prodigal:002006;locus_tag=M1404_00762;product=hypothetical protein;protein_id=gnl|Prokka|M1404_00762

gnl|Prokka|M1404_1 prokka gene 839030 841081 . - . ID=M1404_00763_gene;Name=prc;gene=prc;locus_tag=M1404_00763

gnl|Prokka|M1404_1 Prodigal:002006 CDS 839030 841081 . - 0 ID=M1404_00763;Parent=M1404_00763_gene;eC_number=3.4.21.102;Name=prc;db_xref=COG:COG0793;gene=prc;inference=ab initio prediction:Prodigal:002006,similar to AA sequence:UniProtKB:P23865;locus_tag=M1404_00763;product=Tail-specific protease;protein_id=gnl|Prokka|M1404_00763

gnl|Prokka|M1404_1 prokka gene 841174 841806 . - . ID=M1404_00764_gene;Name=proQ;gene=proQ;locus_tag=M1404_00764

gnl|Prokka|M1404_1 Prodigal:002006 CDS 841174 841806 . - 0 ID=M1404_00764;Parent=M1404_00764_gene;Name=proQ;db_xref=COG:COG3109;gene=proQ;inference=ab initio prediction:Prodigal:002006,similar to AA sequence:UniProtKB:P45577;locus_tag=M1404_00764;product=RNA chaperone ProQ;protein_id=gnl|Prokka|M1404_00764

gnl|Prokka|M1404_1 prokka gene 842032 843288 . + . ID=M1404_00765_gene;Name=yebS;gene=yebS;locus_tag=M1404_00765

gnl|Prokka|M1404_1 Prodigal:002006 CDS 842032 843288 . + 0 ID=M1404_00765;Parent=M1404_00765_gene;Name=yebS;db_xref=COG:COG2995;gene=yebS;inference=ab initio prediction:Prodigal:002006,similar to AA sequence:UniProtKB:P0AD03;locus_tag=M1404_00765;product=Intermembrane transport protein YebS;protein_id=gnl|Prokka|M1404_00765

gnl|Prokka|M1404_1 prokka gene 843266 845920 . + . ID=M1404_00766_gene;locus_tag=M1404_00766

gnl|Prokka|M1404_1 Prodigal:002006 CDS 843266 845920 . + 0 ID=M1404_00766;Parent=M1404_00766_gene;db_xref=COG:COG3008;inference=ab initio prediction:Prodigal:002006,similar to AA sequence:UniProtKB:P44288;locus_tag=M1404_00766;product=putative protein;protein_id=gnl|Prokka|M1404_00766

gnl|Prokka|M1404_1 prokka gene 845986 847224 . - . ID=M1404_00767_gene;Name=pepT;gene=pepT;locus_tag=M1404_00767

gnl|Prokka|M1404_1 Prodigal:002006 CDS 845986 847224 . - 0 ID=M1404_00767;Parent=M1404_00767_gene;eC_number=3.4.11.4;Name=pepT;db_xref=COG:COG2195;gene=pepT;inference=ab initio prediction:Prodigal:002006,similar to AA sequence:UniProtKB:P29745;locus_tag=M1404_00767;product=Peptidase T;protein_id=gnl|Prokka|M1404_00767

gnl|Prokka|M1404_1 prokka gene 847548 848660 . + . ID=M1404_00768_gene;Name=potA_1;gene=potA_1;locus_tag=M1404_00768

gnl|Prokka|M1404_1 Prodigal:002006 CDS 847548 848660 . + 0 ID=M1404_00768;Parent=M1404_00768_gene;eC_number=7.6.2.11;Name=potA_1;db_xref=COG:COG3842;gene=potA_1;inference=ab initio prediction:Prodigal:002006,similar to AA sequence:UniProtKB:P69874;locus_tag=M1404_00768;product=Spermidine/putrescine import ATP-binding protein PotA;protein_id=gnl|Prokka|M1404_00768

gnl|Prokka|M1404_1 prokka gene 848650 849504 . + . ID=M1404_00769_gene;Name=potB;gene=potB;locus_tag=M1404_00769

gnl|Prokka|M1404_1 Prodigal:002006 CDS 848650 849504 . + 0 ID=M1404_00769;Parent=M1404_00769_gene;Name=potB;db_xref=COG:COG1176;gene=potB;inference=ab initio prediction:Prodigal:002006,similar to AA sequence:UniProtKB:P0AFK4;locus_tag=M1404_00769;product=Spermidine/putrescine transport system permease protein PotB;protein_id=gnl|Prokka|M1404_00769

gnl|Prokka|M1404_1 prokka gene 849504 850271 . + . ID=M1404_00770_gene;Name=ydcV;gene=ydcV;locus_tag=M1404_00770

gnl|Prokka|M1404_1 Prodigal:002006 CDS 849504 850271 . + 0 ID=M1404_00770;Parent=M1404_00770_gene;Name=ydcV;gene=ydcV;inference=ab initio prediction:Prodigal:002006,similar to AA sequence:UniProtKB:P0AFR9;locus_tag=M1404_00770;product=Inner membrane ABC transporter permease protein YdcV;protein_id=gnl|Prokka|M1404_00770

gnl|Prokka|M1404_1 prokka gene 850401 851447 . + . ID=M1404_00771_gene;Name=potD_1;gene=potD_1;locus_tag=M1404_00771

gnl|Prokka|M1404_1 Prodigal:002006 CDS 850401 851447 . + 0 ID=M1404_00771;Parent=M1404_00771_gene;Name=potD_1;db_xref=COG:COG0687;gene=potD_1;inference=ab initio prediction:Prodigal:002006,similar to AA sequence:UniProtKB:P0AFK9;locus_tag=M1404_00771;product=Spermidine/putrescine-binding periplasmic protein;protein_id=gnl|Prokka|M1404_00771

gnl|Prokka|M1404_1 prokka gene 851707 852753 . + . ID=M1404_00772_gene;Name=potD_2;gene=potD_2;locus_tag=M1404_00772

gnl|Prokka|M1404_1 Prodigal:002006 CDS 851707 852753 . + 0 ID=M1404_00772;Parent=M1404_00772_gene;Name=potD_2;db_xref=COG:COG0687;gene=potD_2;inference=ab initio prediction:Prodigal:002006,similar to AA sequence:UniProtKB:P0AFK9;locus_tag=M1404_00772;product=Spermidine/putrescine-binding periplasmic protein;protein_id=gnl|Prokka|M1404_00772

gnl|Prokka|M1404_1 prokka gene 852858 853751 . + . ID=M1404_00773_gene;Name=cdd;gene=cdd;locus_tag=M1404_00773

gnl|Prokka|M1404_1 Prodigal:002006 CDS 852858 853751 . + 0 ID=M1404_00773;Parent=M1404_00773_gene;eC_number=3.5.4.5;Name=cdd;db_xref=COG:COG0295;gene=cdd;inference=ab initio prediction:Prodigal:002006,similar to AA sequence:UniProtKB:Q9KSM5;locus_tag=M1404_00773;product=Cytidine deaminase;protein_id=gnl|Prokka|M1404_00773

gnl|Prokka|M1404_1 prokka gene 853823 855109 . - . ID=M1404_00774_gene;Name=serS;gene=serS;locus_tag=M1404_00774

gnl|Prokka|M1404_1 Prodigal:002006 CDS 853823 855109 . - 0 ID=M1404_00774;Parent=M1404_00774_gene;eC_number=6.1.1.11;Name=serS;db_xref=COG:COG0172;gene=serS;inference=ab initio prediction:Prodigal:002006,similar to AA sequence:UniProtKB:P0A8L1;locus_tag=M1404_00774;product=Serine--tRNA ligase;protein_id=gnl|Prokka|M1404_00774

gnl|Prokka|M1404_1 prokka gene 855368 856705 . - . ID=M1404_00775_gene;Name=rarA;gene=rarA;locus_tag=M1404_00775

gnl|Prokka|M1404_1 Prodigal:002006 CDS 855368 856705 . - 0 ID=M1404_00775;Parent=M1404_00775_gene;Name=rarA;db_xref=COG:COG2256;gene=rarA;inference=ab initio prediction:Prodigal:002006,similar to AA sequence:UniProtKB:P0AAZ4;locus_tag=M1404_00775;product=Replication-associated recombination protein A;protein_id=gnl|Prokka|M1404_00775

gnl|Prokka|M1404_1 prokka gene 856717 857334 . - . ID=M1404_00776_gene;Name=lolA;gene=lolA;locus_tag=M1404_00776

gnl|Prokka|M1404_1 Prodigal:002006 CDS 856717 857334 . - 0 ID=M1404_00776;Parent=M1404_00776_gene;Name=lolA;db_xref=COG:COG2834;gene=lolA;inference=ab initio prediction:Prodigal:002006,similar to AA sequence:UniProtKB:Q8ZGC6;locus_tag=M1404_00776;product=Outer-membrane lipoprotein carrier protein;protein_id=gnl|Prokka|M1404_00776

gnl|Prokka|M1404_1 prokka gene 857345 860137 . - . ID=M1404_00777_gene;locus_tag=M1404_00777

gnl|Prokka|M1404_1 Prodigal:002006 CDS 857345 860137 . - 0 ID=M1404_00777;Parent=M1404_00777_gene;inference=ab initio prediction:Prodigal:002006;locus_tag=M1404_00777;product=hypothetical protein;protein_id=gnl|Prokka|M1404_00777

gnl|Prokka|M1404_1 prokka gene 860142 860621 . - . ID=M1404_00778_gene;Name=lrp;gene=lrp;locus_tag=M1404_00778

gnl|Prokka|M1404_1 Prodigal:002006 CDS 860142 860621 . - 0 ID=M1404_00778;Parent=M1404_00778_gene;Name=lrp;db_xref=COG:COG1522;gene=lrp;inference=ab initio prediction:Prodigal:002006,similar to AA sequence:UniProtKB:P0ACJ0;locus_tag=M1404_00778;product=Leucine-responsive regulatory protein;protein_id=gnl|Prokka|M1404_00778

gnl|Prokka|M1404_1 prokka gene 860793 861128 . + . ID=M1404_00779_gene;locus_tag=M1404_00779

gnl|Prokka|M1404_1 Prodigal:002006 CDS 860793 861128 . + 0 ID=M1404_00779;Parent=M1404_00779_gene;inference=ab initio prediction:Prodigal:002006;locus_tag=M1404_00779;product=hypothetical protein;protein_id=gnl|Prokka|M1404_00779

gnl|Prokka|M1404_1 prokka gene 861150 862529 . - . ID=M1404_00780_gene;Name=radA;gene=radA;locus_tag=M1404_00780

gnl|Prokka|M1404_1 Prodigal:002006 CDS 861150 862529 . - 0 ID=M1404_00780;Parent=M1404_00780_gene;eC_number=3.6.4.-;Name=radA;db_xref=COG:COG1066;gene=radA;inference=ab initio prediction:Prodigal:002006,similar to AA sequence:UniProtKB:P24554;locus_tag=M1404_00780;product=DNA repair protein RadA;protein_id=gnl|Prokka|M1404_00780

gnl|Prokka|M1404_1 prokka gene 862533 863600 . - . ID=M1404_00781_gene;locus_tag=M1404_00781

gnl|Prokka|M1404_1 Prodigal:002006 CDS 862533 863600 . - 0 ID=M1404_00781;Parent=M1404_00781_gene;inference=ab initio prediction:Prodigal:002006;locus_tag=M1404_00781;product=hypothetical protein;protein_id=gnl|Prokka|M1404_00781

gnl|Prokka|M1404_1 prokka gene 863776 864456 . + . ID=M1404_00782_gene;locus_tag=M1404_00782

gnl|Prokka|M1404_1 Prodigal:002006 CDS 863776 864456 . + 0 ID=M1404_00782;Parent=M1404_00782_gene;inference=ab initio prediction:Prodigal:002006,similar to AA sequence:UniProtKB:P44271;locus_tag=M1404_00782;note=UPF0111 protein HI_1603;product=hypothetical protein;protein_id=gnl|Prokka|M1404_00782

gnl|Prokka|M1404_1 prokka gene 864483 865745 . + . ID=M1404_00783_gene;locus_tag=M1404_00783

gnl|Prokka|M1404_1 Prodigal:002006 CDS 864483 865745 . + 0 ID=M1404_00783;Parent=M1404_00783_gene;inference=ab initio prediction:Prodigal:002006;locus_tag=M1404_00783;product=hypothetical protein;protein_id=gnl|Prokka|M1404_00783

gnl|Prokka|M1404_1 prokka gene 865810 866421 . + . ID=M1404_00784_gene;locus_tag=M1404_00784

gnl|Prokka|M1404_1 Prodigal:002006 CDS 865810 866421 . + 0 ID=M1404_00784;Parent=M1404_00784_gene;inference=ab initio prediction:Prodigal:002006;locus_tag=M1404_00784;product=hypothetical protein;protein_id=gnl|Prokka|M1404_00784

gnl|Prokka|M1404_1 prokka gene 866431 867705 . + . ID=M1404_00785_gene;Name=cca;gene=cca;locus_tag=M1404_00785

gnl|Prokka|M1404_1 Prodigal:002006 CDS 866431 867705 . + 0 ID=M1404_00785;Parent=M1404_00785_gene;Name=cca;db_xref=COG:COG0617;gene=cca;inference=ab initio prediction:Prodigal:002006,similar to AA sequence:UniProtKB:P06961;locus_tag=M1404_00785;product=Multifunctional CCA protein;protein_id=gnl|Prokka|M1404_00785

gnl|Prokka|M1404_1 prokka gene 867746 868363 . + . ID=M1404_00786_gene;Name=lolB;gene=lolB;locus_tag=M1404_00786

gnl|Prokka|M1404_1 Prodigal:002006 CDS 867746 868363 . + 0 ID=M1404_00786;Parent=M1404_00786_gene;Name=lolB;db_xref=COG:COG3017;gene=lolB;inference=ab initio prediction:Prodigal:002006,similar to AA sequence:UniProtKB:P61320;locus_tag=M1404_00786;product=Outer-membrane lipoprotein LolB;protein_id=gnl|Prokka|M1404_00786

gnl|Prokka|M1404_1 prokka gene 868363 869250 . + . ID=M1404_00787_gene;Name=ispE;gene=ispE;locus_tag=M1404_00787

gnl|Prokka|M1404_1 Prodigal:002006 CDS 868363 869250 . + 0 ID=M1404_00787;Parent=M1404_00787_gene;eC_number=2.7.1.148;Name=ispE;db_xref=COG:COG1947;gene=ispE;inference=ab initio prediction:Prodigal:002006,similar to AA sequence:UniProtKB:Q8FI04;locus_tag=M1404_00787;product=4-diphosphocytidyl-2-C-methyl-D-erythritol kinase;protein_id=gnl|Prokka|M1404_00787

gnl|Prokka|M1404_1 prokka gene 869320 870267 . + . ID=M1404_00788_gene;Name=prs;gene=prs;locus_tag=M1404_00788

gnl|Prokka|M1404_1 Prodigal:002006 CDS 869320 870267 . + 0 ID=M1404_00788;Parent=M1404_00788_gene;eC_number=2.7.6.1;Name=prs;db_xref=COG:COG0462;gene=prs;inference=ab initio prediction:Prodigal:002006,similar to AA sequence:UniProtKB:P0A1V6;locus_tag=M1404_00788;product=Ribose-phosphate pyrophosphokinase;protein_id=gnl|Prokka|M1404_00788

gnl|Prokka|M1404_1 prokka gene 870343 871938 . - . ID=M1404_00789_gene;locus_tag=M1404_00789

gnl|Prokka|M1404_1 Prodigal:002006 CDS 870343 871938 . - 0 ID=M1404_00789;Parent=M1404_00789_gene;inference=ab initio prediction:Prodigal:002006;locus_tag=M1404_00789;product=hypothetical protein;protein_id=gnl|Prokka|M1404_00789

gnl|Prokka|M1404_1 prokka gene 872131 872922 . + . ID=M1404_00790_gene;Name=znuC;gene=znuC;locus_tag=M1404_00790

gnl|Prokka|M1404_1 Prodigal:002006 CDS 872131 872922 . + 0 ID=M1404_00790;Parent=M1404_00790_gene;eC_number=7.2.2.-;Name=znuC;db_xref=COG:COG1121;gene=znuC;inference=ab initio prediction:Prodigal:002006,similar to AA sequence:UniProtKB:Q8ZNV7;locus_tag=M1404_00790;product=Zinc import ATP-binding protein ZnuC;protein_id=gnl|Prokka|M1404_00790

gnl|Prokka|M1404_1 prokka gene 872931 873716 . + . ID=M1404_00791_gene;Name=znuB;gene=znuB;locus_tag=M1404_00791

gnl|Prokka|M1404_1 Prodigal:002006 CDS 872931 873716 . + 0 ID=M1404_00791;Parent=M1404_00791_gene;Name=znuB;db_xref=COG:COG1108;gene=znuB;inference=ab initio prediction:Prodigal:002006,similar to AA sequence:UniProtKB:P39832;locus_tag=M1404_00791;product=High-affinity zinc uptake system membrane protein ZnuB;protein_id=gnl|Prokka|M1404_00791

gnl|Prokka|M1404_1 prokka gene 873795 874775 . - . ID=M1404_00792_gene;Name=ddpF;gene=ddpF;locus_tag=M1404_00792

gnl|Prokka|M1404_1 Prodigal:002006 CDS 873795 874775 . - 0 ID=M1404_00792;Parent=M1404_00792_gene;Name=ddpF;db_xref=COG:COG1124;gene=ddpF;inference=ab initio prediction:Prodigal:002006,similar to AA sequence:UniProtKB:P77622;locus_tag=M1404_00792;product=putative D%2CD-dipeptide transport ATP-binding protein DdpF;protein_id=gnl|Prokka|M1404_00792

gnl|Prokka|M1404_1 prokka gene 874792 875790 . - . ID=M1404_00793_gene;Name=dppD;gene=dppD;locus_tag=M1404_00793

gnl|Prokka|M1404_1 Prodigal:002006 CDS 874792 875790 . - 0 ID=M1404_00793;Parent=M1404_00793_gene;Name=dppD;db_xref=COG:COG0444;gene=dppD;inference=ab initio prediction:Prodigal:002006,similar to AA sequence:UniProtKB:P0AAG0;locus_tag=M1404_00793;product=Dipeptide transport ATP-binding protein DppD;protein_id=gnl|Prokka|M1404_00793

gnl|Prokka|M1404_1 prokka gene 875801 876694 . - . ID=M1404_00794_gene;Name=dppC;gene=dppC;locus_tag=M1404_00794

gnl|Prokka|M1404_1 Prodigal:002006 CDS 875801 876694 . - 0 ID=M1404_00794;Parent=M1404_00794_gene;Name=dppC;db_xref=COG:COG1173;gene=dppC;inference=ab initio prediction:Prodigal:002006,similar to AA sequence:UniProtKB:P0AEG1;locus_tag=M1404_00794;product=Dipeptide transport system permease protein DppC;protein_id=gnl|Prokka|M1404_00794

gnl|Prokka|M1404_1 prokka gene 876717 877721 . - . ID=M1404_00795_gene;Name=dppB;gene=dppB;locus_tag=M1404_00795

gnl|Prokka|M1404_1 Prodigal:002006 CDS 876717 877721 . - 0 ID=M1404_00795;Parent=M1404_00795_gene;Name=dppB;db_xref=COG:COG0601;gene=dppB;inference=ab initio prediction:Prodigal:002006,similar to AA sequence:UniProtKB:P0AEF8;locus_tag=M1404_00795;product=Dipeptide transport system permease protein DppB;protein_id=gnl|Prokka|M1404_00795

gnl|Prokka|M1404_1 prokka gene 877909 879507 . - . ID=M1404_00796_gene;Name=dppA;gene=dppA;locus_tag=M1404_00796

gnl|Prokka|M1404_1 Prodigal:002006 CDS 877909 879507 . - 0 ID=M1404_00796;Parent=M1404_00796_gene;Name=dppA;db_xref=COG:COG0747;gene=dppA;inference=ab initio prediction:Prodigal:002006,similar to AA sequence:UniProtKB:P23847;locus_tag=M1404_00796;product=Periplasmic dipeptide transport protein;protein_id=gnl|Prokka|M1404_00796

gnl|Prokka|M1404_1 prokka gene 879850 880737 . - . ID=M1404_00797_gene;Name=metF;gene=metF;locus_tag=M1404_00797

gnl|Prokka|M1404_1 Prodigal:002006 CDS 879850 880737 . - 0 ID=M1404_00797;Parent=M1404_00797_gene;eC_number=1.5.1.20;Name=metF;db_xref=COG:COG0685;gene=metF;inference=ab initio prediction:Prodigal:002006,similar to AA sequence:UniProtKB:P45208;locus_tag=M1404_00797;product=5%2C10-methylenetetrahydrofolate reductase;protein_id=gnl|Prokka|M1404_00797

gnl|Prokka|M1404_1 prokka gene 880940 883066 . + . ID=M1404_00798_gene;locus_tag=M1404_00798

gnl|Prokka|M1404_1 Prodigal:002006 CDS 880940 883066 . + 0 ID=M1404_00798;Parent=M1404_00798_gene;inference=ab initio prediction:Prodigal:002006;locus_tag=M1404_00798;product=hypothetical protein;protein_id=gnl|Prokka|M1404_00798

gnl|Prokka|M1404_1 prokka gene 883227 884597 . + . ID=M1404_00799_gene;locus_tag=M1404_00799

gnl|Prokka|M1404_1 Prodigal:002006 CDS 883227 884597 . + 0 ID=M1404_00799;Parent=M1404_00799_gene;inference=ab initio prediction:Prodigal:002006;locus_tag=M1404_00799;product=hypothetical protein;protein_id=gnl|Prokka|M1404_00799

gnl|Prokka|M1404_1 prokka gene 884851 885942 . + . ID=M1404_00800_gene;Name=queA;gene=queA;locus_tag=M1404_00800

gnl|Prokka|M1404_1 Prodigal:002006 CDS 884851 885942 . + 0 ID=M1404_00800;Parent=M1404_00800_gene;eC_number=2.4.99.17;Name=queA;db_xref=COG:COG0809;gene=queA;inference=ab initio prediction:Prodigal:002006,similar to AA sequence:UniProtKB:P0A7F9;locus_tag=M1404_00800;product=S-adenosylmethionine:tRNA ribosyltransferase-isomerase;protein_id=gnl|Prokka|M1404_00800

gnl|Prokka|M1404_1 prokka gene 885942 886697 . + . ID=M1404_00801_gene;Name=cof;gene=cof;locus_tag=M1404_00801

gnl|Prokka|M1404_1 Prodigal:002006 CDS 885942 886697 . + 0 ID=M1404_00801;Parent=M1404_00801_gene;eC_number=3.6.1.-;Name=cof;gene=cof;inference=ab initio prediction:Prodigal:002006,protein motif:HAMAP:MF_01847;locus_tag=M1404_00801;product=HMP-PP phosphatase;protein_id=gnl|Prokka|M1404_00801

gnl|Prokka|M1404_1 prokka gene 886736 888085 . + . ID=M1404_00802_gene;Name=dcuD_1;gene=dcuD_1;locus_tag=M1404_00802

gnl|Prokka|M1404_1 Prodigal:002006 CDS 886736 888085 . + 0 ID=M1404_00802;Parent=M1404_00802_gene;Name=dcuD_1;db_xref=COG:COG3069;gene=dcuD_1;inference=ab initio prediction:Prodigal:002006,similar to AA sequence:UniProtKB:P45428;locus_tag=M1404_00802;product=Putative cryptic C4-dicarboxylate transporter DcuD;protein_id=gnl|Prokka|M1404_00802

gnl|Prokka|M1404_1 prokka gene 888300 889457 . + . ID=M1404_00803_gene;Name=tgt;gene=tgt;locus_tag=M1404_00803

gnl|Prokka|M1404_1 Prodigal:002006 CDS 888300 889457 . + 0 ID=M1404_00803;Parent=M1404_00803_gene;eC_number=2.4.2.29;Name=tgt;db_xref=COG:COG0343;gene=tgt;inference=ab initio prediction:Prodigal:002006,similar to AA sequence:UniProtKB:P0A847;locus_tag=M1404_00803;product=Queuine tRNA-ribosyltransferase;protein_id=gnl|Prokka|M1404_00803

gnl|Prokka|M1404_1 prokka gene 889755 890051 . + . ID=M1404_00804_gene;Name=yajC;gene=yajC;locus_tag=M1404_00804

gnl|Prokka|M1404_1 Prodigal:002006 CDS 889755 890051 . + 0 ID=M1404_00804;Parent=M1404_00804_gene;Name=yajC;db_xref=COG:COG1862;gene=yajC;inference=ab initio prediction:Prodigal:002006,similar to AA sequence:UniProtKB:P0ADZ7;locus_tag=M1404_00804;product=Sec translocon accessory complex subunit YajC;protein_id=gnl|Prokka|M1404_00804

gnl|Prokka|M1404_1 prokka gene 890077 891927 . + . ID=M1404_00805_gene;Name=secD;gene=secD;locus_tag=M1404_00805

gnl|Prokka|M1404_1 Prodigal:002006 CDS 890077 891927 . + 0 ID=M1404_00805;Parent=M1404_00805_gene;Name=secD;db_xref=COG:COG0342;gene=secD;inference=ab initio prediction:Prodigal:002006,similar to AA sequence:UniProtKB:P0AG90;locus_tag=M1404_00805;product=Protein translocase subunit SecD;protein_id=gnl|Prokka|M1404_00805

gnl|Prokka|M1404_1 prokka gene 891938 892909 . + . ID=M1404_00806_gene;Name=secF;gene=secF;locus_tag=M1404_00806

gnl|Prokka|M1404_1 Prodigal:002006 CDS 891938 892909 . + 0 ID=M1404_00806;Parent=M1404_00806_gene;Name=secF;db_xref=COG:COG0341;gene=secF;inference=ab initio prediction:Prodigal:002006,similar to AA sequence:UniProtKB:E9RGS4;locus_tag=M1404_00806;product=Protein translocase subunit SecF;protein_id=gnl|Prokka|M1404_00806

gnl|Prokka|M1404_1 prokka gene 893048 894310 . - . ID=M1404_00807_gene;Name=glyA;gene=glyA;locus_tag=M1404_00807

gnl|Prokka|M1404_1 Prodigal:002006 CDS 893048 894310 . - 0 ID=M1404_00807;Parent=M1404_00807_gene;eC_number=2.1.2.1;Name=glyA;db_xref=COG:COG0112;gene=glyA;inference=ab initio prediction:Prodigal:002006,similar to AA sequence:UniProtKB:P0A825;locus_tag=M1404_00807;product=Serine hydroxymethyltransferase;protein_id=gnl|Prokka|M1404_00807

gnl|Prokka|M1404_1 prokka gene 894438 895727 . - . ID=M1404_00808_gene;Name=purD;gene=purD;locus_tag=M1404_00808

gnl|Prokka|M1404_1 Prodigal:002006 CDS 894438 895727 . - 0 ID=M1404_00808;Parent=M1404_00808_gene;eC_number=6.3.4.13;Name=purD;db_xref=COG:COG0151;gene=purD;inference=ab initio prediction:Prodigal:002006,similar to AA sequence:UniProtKB:Q8ZAR2;locus_tag=M1404_00808;product=Phosphoribosylamine--glycine ligase;protein_id=gnl|Prokka|M1404_00808

gnl|Prokka|M1404_1 prokka gene 895730 897007 . - . ID=M1404_00809_gene;locus_tag=M1404_00809

gnl|Prokka|M1404_1 Prodigal:002006 CDS 895730 897007 . - 0 ID=M1404_00809;Parent=M1404_00809_gene;inference=ab initio prediction:Prodigal:002006;locus_tag=M1404_00809;product=hypothetical protein;protein_id=gnl|Prokka|M1404_00809

gnl|Prokka|M1404_1 prokka gene 897032 897316 . - . ID=M1404_00810_gene;locus_tag=M1404_00810

gnl|Prokka|M1404_1 Prodigal:002006 CDS 897032 897316 . - 0 ID=M1404_00810;Parent=M1404_00810_gene;inference=ab initio prediction:Prodigal:002006;locus_tag=M1404_00810;product=hypothetical protein;protein_id=gnl|Prokka|M1404_00810

gnl|Prokka|M1404_1 prokka gene 897355 898956 . - . ID=M1404_00811_gene;Name=purH;gene=purH;locus_tag=M1404_00811

gnl|Prokka|M1404_1 Prodigal:002006 CDS 897355 898956 . - 0 ID=M1404_00811;Parent=M1404_00811_gene;Name=purH;db_xref=COG:COG0138;gene=purH;inference=ab initio prediction:Prodigal:002006,similar to AA sequence:UniProtKB:P15639;locus_tag=M1404_00811;product=Bifunctional purine biosynthesis protein PurH;protein_id=gnl|Prokka|M1404_00811

gnl|Prokka|M1404_1 prokka gene 899129 900871 . - . ID=M1404_00812_gene;Name=dsbD_1;gene=dsbD_1;locus_tag=M1404_00812

gnl|Prokka|M1404_1 Prodigal:002006 CDS 899129 900871 . - 0 ID=M1404_00812;Parent=M1404_00812_gene;eC_number=1.8.1.8;Name=dsbD_1;db_xref=COG:COG4232;gene=dsbD_1;inference=ab initio prediction:Prodigal:002006,similar to AA sequence:UniProtKB:P36655;locus_tag=M1404_00812;product=Thiol:disulfide interchange protein DsbD;protein_id=gnl|Prokka|M1404_00812

gnl|Prokka|M1404_1 prokka gene 901068 901334 . + . ID=M1404_00813_gene;Name=rpmE2;gene=rpmE2;locus_tag=M1404_00813

gnl|Prokka|M1404_1 Prodigal:002006 CDS 901068 901334 . + 0 ID=M1404_00813;Parent=M1404_00813_gene;Name=rpmE2;db_xref=COG:COG0254;gene=rpmE2;inference=ab initio prediction:Prodigal:002006,similar to AA sequence:UniProtKB:Q9KTM4;locus_tag=M1404_00813;product=50S ribosomal protein L31 type B;protein_id=gnl|Prokka|M1404_00813

gnl|Prokka|M1404_1 prokka gene 901345 901485 . + . ID=M1404_00814_gene;Name=ykgO;gene=ykgO;locus_tag=M1404_00814

gnl|Prokka|M1404_1 Prodigal:002006 CDS 901345 901485 . + 0 ID=M1404_00814;Parent=M1404_00814_gene;Name=ykgO;db_xref=COG:COG0257;gene=ykgO;inference=ab initio prediction:Prodigal:002006,similar to AA sequence:UniProtKB:Q2EEQ2;locus_tag=M1404_00814;product=50S ribosomal protein L36 2;protein_id=gnl|Prokka|M1404_00814

gnl|Prokka|M1404_1 prokka gene 901723 902433 . + . ID=M1404_00815_gene;Name=arcA;gene=arcA;locus_tag=M1404_00815

gnl|Prokka|M1404_1 Prodigal:002006 CDS 901723 902433 . + 0 ID=M1404_00815;Parent=M1404_00815_gene;Name=arcA;db_xref=COG:COG0745;gene=arcA;inference=ab initio prediction:Prodigal:002006,similar to AA sequence:UniProtKB:P0A9Q1;locus_tag=M1404_00815;product=Aerobic respiration control protein ArcA;protein_id=gnl|Prokka|M1404_00815

gnl|Prokka|M1404_1 prokka gene 902482 904008 . - . ID=M1404_00816_gene;Name=ubiD;gene=ubiD;locus_tag=M1404_00816

gnl|Prokka|M1404_1 Prodigal:002006 CDS 902482 904008 . - 0 ID=M1404_00816;Parent=M1404_00816_gene;eC_number=4.1.1.98;Name=ubiD;db_xref=COG:COG0043;gene=ubiD;inference=ab initio prediction:Prodigal:002006,similar to AA sequence:UniProtKB:P0AAB4;locus_tag=M1404_00816;product=3-octaprenyl-4-hydroxybenzoate carboxy-lyase;protein_id=gnl|Prokka|M1404_00816

gnl|Prokka|M1404_1 prokka gene 904120 904602 . + . ID=M1404_00817_gene;Name=smpB;gene=smpB;locus_tag=M1404_00817

gnl|Prokka|M1404_1 Prodigal:002006 CDS 904120 904602 . + 0 ID=M1404_00817;Parent=M1404_00817_gene;Name=smpB;db_xref=COG:COG0691;gene=smpB;inference=ab initio prediction:Prodigal:002006,similar to AA sequence:UniProtKB:P0A832;locus_tag=M1404_00817;product=SsrA-binding protein;protein_id=gnl|Prokka|M1404_00817

gnl|Prokka|M1404_1 prokka gene 904652 904861 . - . ID=M1404_00818_gene;Name=mopII;gene=mopII;locus_tag=M1404_00818

gnl|Prokka|M1404_1 Prodigal:002006 CDS 904652 904861 . - 0 ID=M1404_00818;Parent=M1404_00818_gene;Name=mopII;gene=mopII;inference=ab initio prediction:Prodigal:002006,similar to AA sequence:UniProtKB:P08854;locus_tag=M1404_00818;product=Molybdenum-pterin-binding protein 2;protein_id=gnl|Prokka|M1404_00818

gnl|Prokka|M1404_1 prokka gene 904949 906322 . - . ID=M1404_00819_gene;Name=qseC;gene=qseC;locus_tag=M1404_00819

gnl|Prokka|M1404_1 Prodigal:002006 CDS 904949 906322 . - 0 ID=M1404_00819;Parent=M1404_00819_gene;eC_number=2.7.13.3;Name=qseC;gene=qseC;inference=ab initio prediction:Prodigal:002006,similar to AA sequence:UniProtKB:P45336;locus_tag=M1404_00819;product=Sensor protein QseC;protein_id=gnl|Prokka|M1404_00819

gnl|Prokka|M1404_1 prokka gene 906309 906980 . - . ID=M1404_00820_gene;Name=qseB;gene=qseB;locus_tag=M1404_00820

gnl|Prokka|M1404_1 Prodigal:002006 CDS 906309 906980 . - 0 ID=M1404_00820;Parent=M1404_00820_gene;Name=qseB;gene=qseB;inference=ab initio prediction:Prodigal:002006,similar to AA sequence:UniProtKB:P52076;locus_tag=M1404_00820;product=Transcriptional regulatory protein QseB;protein_id=gnl|Prokka|M1404_00820

gnl|Prokka|M1404_1 prokka gene 907457 907906 . + . ID=M1404_00821_gene;locus_tag=M1404_00821

gnl|Prokka|M1404_1 Prodigal:002006 CDS 907457 907906 . + 0 ID=M1404_00821;Parent=M1404_00821_gene;inference=ab initio prediction:Prodigal:002006;locus_tag=M1404_00821;product=hypothetical protein;protein_id=gnl|Prokka|M1404_00821

gnl|Prokka|M1404_1 prokka gene 907949 908239 . + . ID=M1404_00822_gene;locus_tag=M1404_00822

gnl|Prokka|M1404_1 Prodigal:002006 CDS 907949 908239 . + 0 ID=M1404_00822;Parent=M1404_00822_gene;inference=ab initio prediction:Prodigal:002006;locus_tag=M1404_00822;product=hypothetical protein;protein_id=gnl|Prokka|M1404_00822

gnl|Prokka|M1404_1 prokka gene 908301 909224 . + . ID=M1404_00823_gene;locus_tag=M1404_00823

gnl|Prokka|M1404_1 Prodigal:002006 CDS 908301 909224 . + 0 ID=M1404_00823;Parent=M1404_00823_gene;inference=ab initio prediction:Prodigal:002006,similar to AA sequence:UniProtKB:A0A0H3CEP9;locus_tag=M1404_00823;note=UPF0276 protein CCNA_03364;product=hypothetical protein;protein_id=gnl|Prokka|M1404_00823

gnl|Prokka|M1404_1 prokka gene 909214 909933 . + . ID=M1404_00824_gene;locus_tag=M1404_00824

gnl|Prokka|M1404_1 Prodigal:002006 CDS 909214 909933 . + 0 ID=M1404_00824;Parent=M1404_00824_gene;inference=ab initio prediction:Prodigal:002006;locus_tag=M1404_00824;product=hypothetical protein;protein_id=gnl|Prokka|M1404_00824

gnl|Prokka|M1404_1 prokka gene 910000 910200 . - . ID=M1404_00825_gene;locus_tag=M1404_00825

gnl|Prokka|M1404_1 Prodigal:002006 CDS 910000 910200 . - 0 ID=M1404_00825;Parent=M1404_00825_gene;inference=ab initio prediction:Prodigal:002006;locus_tag=M1404_00825;product=hypothetical protein;protein_id=gnl|Prokka|M1404_00825

gnl|Prokka|M1404_1 prokka gene 910201 910752 . - . ID=M1404_00826_gene;Name=algU;gene=algU;locus_tag=M1404_00826

gnl|Prokka|M1404_1 Prodigal:002006 CDS 910201 910752 . - 0 ID=M1404_00826;Parent=M1404_00826_gene;Name=algU;db_xref=COG:COG1595;gene=algU;inference=ab initio prediction:Prodigal:002006,similar to AA sequence:UniProtKB:Q06198;locus_tag=M1404_00826;product=RNA polymerase sigma-H factor;protein_id=gnl|Prokka|M1404_00826

gnl|Prokka|M1404_1 prokka gene 910874 910959 . - . ID=M1404_00827_gene;locus_tag=M1404_00827

gnl|Prokka|M1404_1 Aragorn:001002 tRNA 910874 910959 . - . ID=M1404_00827;Parent=M1404_00827_gene;inference=COORDINATES:profile:Aragorn:001002;locus_tag=M1404_00827;product=tRNA-Leu(gag)

gnl|Prokka|M1404_1 prokka gene 910973 911320 . - . ID=M1404_00828_gene;Name=secG;gene=secG;locus_tag=M1404_00828

gnl|Prokka|M1404_1 Prodigal:002006 CDS 910973 911320 . - 0 ID=M1404_00828;Parent=M1404_00828_gene;Name=secG;db_xref=COG:COG1314;gene=secG;inference=ab initio prediction:Prodigal:002006,similar to AA sequence:UniProtKB:P0AG99;locus_tag=M1404_00828;product=Protein-export membrane protein SecG;protein_id=gnl|Prokka|M1404_00828

gnl|Prokka|M1404_1 prokka gene 911539 913491 . - . ID=M1404_00829_gene;Name=topB;gene=topB;locus_tag=M1404_00829

gnl|Prokka|M1404_1 Prodigal:002006 CDS 911539 913491 . - 0 ID=M1404_00829;Parent=M1404_00829_gene;eC_number=5.6.2.1;Name=topB;db_xref=COG:COG0550;gene=topB;inference=ab initio prediction:Prodigal:002006,similar to AA sequence:UniProtKB:P14294;locus_tag=M1404_00829;product=DNA topoisomerase 3;protein_id=gnl|Prokka|M1404_00829

gnl|Prokka|M1404_1 prokka gene 913501 914103 . - . ID=M1404_00830_gene;Name=recR;gene=recR;locus_tag=M1404_00830

gnl|Prokka|M1404_1 Prodigal:002006 CDS 913501 914103 . - 0 ID=M1404_00830;Parent=M1404_00830_gene;Name=recR;db_xref=COG:COG0353;gene=recR;inference=ab initio prediction:Prodigal:002006,similar to AA sequence:UniProtKB:Q9I3H9;locus_tag=M1404_00830;product=Recombination protein RecR;protein_id=gnl|Prokka|M1404_00830

gnl|Prokka|M1404_1 prokka gene 914305 914634 . - . ID=M1404_00831_gene;Name=ybaB;gene=ybaB;locus_tag=M1404_00831

gnl|Prokka|M1404_1 Prodigal:002006 CDS 914305 914634 . - 0 ID=M1404_00831;Parent=M1404_00831_gene;Name=ybaB;db_xref=COG:COG0718;gene=ybaB;inference=ab initio prediction:Prodigal:002006,similar to AA sequence:UniProtKB:P0A8B5;locus_tag=M1404_00831;product=Nucleoid-associated protein YbaB;protein_id=gnl|Prokka|M1404_00831

gnl|Prokka|M1404_1 prokka gene 914863 917469 . + . ID=M1404_00832_gene;Name=acnB;gene=acnB;locus_tag=M1404_00832

gnl|Prokka|M1404_1 Prodigal:002006 CDS 914863 917469 . + 0 ID=M1404_00832;Parent=M1404_00832_gene;eC_number=4.2.1.3;Name=acnB;db_xref=COG:COG1049;gene=acnB;inference=ab initio prediction:Prodigal:002006,similar to AA sequence:UniProtKB:P36683;locus_tag=M1404_00832;product=Aconitate hydratase B;protein_id=gnl|Prokka|M1404_00832

gnl|Prokka|M1404_1 prokka gene 917571 918599 . + . ID=M1404_00833_gene;locus_tag=M1404_00833

gnl|Prokka|M1404_1 Prodigal:002006 CDS 917571 918599 . + 0 ID=M1404_00833;Parent=M1404_00833_gene;db_xref=COG:COG1840;inference=ab initio prediction:Prodigal:002006,similar to AA sequence:UniProtKB:P43951;locus_tag=M1404_00833;product=putative protein;protein_id=gnl|Prokka|M1404_00833

gnl|Prokka|M1404_1 prokka gene 918650 919621 . - . ID=M1404_00834_gene;Name=epmA;gene=epmA;locus_tag=M1404_00834

gnl|Prokka|M1404_1 Prodigal:002006 CDS 918650 919621 . - 0 ID=M1404_00834;Parent=M1404_00834_gene;eC_number=6.3.1.-;Name=epmA;db_xref=COG:COG2269;gene=epmA;inference=ab initio prediction:Prodigal:002006,similar to AA sequence:UniProtKB:Q9ZJ12;locus_tag=M1404_00834;product=Elongation factor P--(R)-beta-lysine ligase;protein_id=gnl|Prokka|M1404_00834

gnl|Prokka|M1404_1 prokka gene 919974 921773 . + . ID=M1404_00835_gene;Name=frdA;gene=frdA;locus_tag=M1404_00835

gnl|Prokka|M1404_1 Prodigal:002006 CDS 919974 921773 . + 0 ID=M1404_00835;Parent=M1404_00835_gene;eC_number=1.3.5.4;Name=frdA;gene=frdA;inference=ab initio prediction:Prodigal:002006,similar to AA sequence:UniProtKB:V3TQ67;locus_tag=M1404_00835;product=Fumarate reductase flavoprotein subunit;protein_id=gnl|Prokka|M1404_00835

gnl|Prokka|M1404_1 prokka gene 921766 922536 . + . ID=M1404_00836_gene;Name=frdB;gene=frdB;locus_tag=M1404_00836

gnl|Prokka|M1404_1 Prodigal:002006 CDS 921766 922536 . + 0 ID=M1404_00836;Parent=M1404_00836_gene;eC_number=1.3.5.1;Name=frdB;db_xref=COG:COG0479;gene=frdB;inference=ab initio prediction:Prodigal:002006,similar to AA sequence:UniProtKB:P0AC47;locus_tag=M1404_00836;product=Fumarate reductase iron-sulfur subunit;protein_id=gnl|Prokka|M1404_00836

gnl|Prokka|M1404_1 prokka gene 922547 922945 . + . ID=M1404_00837_gene;Name=frdC;gene=frdC;locus_tag=M1404_00837

gnl|Prokka|M1404_1 Prodigal:002006 CDS 922547 922945 . + 0 ID=M1404_00837;Parent=M1404_00837_gene;Name=frdC;db_xref=COG:COG3029;gene=frdC;inference=ab initio prediction:Prodigal:002006,similar to AA sequence:UniProtKB:P0A8Q0;locus_tag=M1404_00837;product=Fumarate reductase subunit C;protein_id=gnl|Prokka|M1404_00837

gnl|Prokka|M1404_1 prokka gene 922960 923310 . + . ID=M1404_00838_gene;Name=frdD;gene=frdD;locus_tag=M1404_00838

gnl|Prokka|M1404_1 Prodigal:002006 CDS 922960 923310 . + 0 ID=M1404_00838;Parent=M1404_00838_gene;Name=frdD;db_xref=COG:COG3080;gene=frdD;inference=ab initio prediction:Prodigal:002006,similar to AA sequence:UniProtKB:P0A8Q3;locus_tag=M1404_00838;product=Fumarate reductase subunit D;protein_id=gnl|Prokka|M1404_00838

gnl|Prokka|M1404_1 prokka gene 923596 924666 . - . ID=M1404_00839_gene;Name=lptG;gene=lptG;locus_tag=M1404_00839

gnl|Prokka|M1404_1 Prodigal:002006 CDS 923596 924666 . - 0 ID=M1404_00839;Parent=M1404_00839_gene;Name=lptG;db_xref=COG:COG0795;gene=lptG;inference=ab initio prediction:Prodigal:002006,similar to AA sequence:UniProtKB:P0ADC6;locus_tag=M1404_00839;product=Lipopolysaccharide export system permease protein LptG;protein_id=gnl|Prokka|M1404_00839

gnl|Prokka|M1404_1 prokka gene 924673 925779 . - . ID=M1404_00840_gene;Name=lptF;gene=lptF;locus_tag=M1404_00840

gnl|Prokka|M1404_1 Prodigal:002006 CDS 924673 925779 . - 0 ID=M1404_00840;Parent=M1404_00840_gene;Name=lptF;db_xref=COG:COG0795;gene=lptF;inference=ab initio prediction:Prodigal:002006,similar to AA sequence:UniProtKB:P0AF98;locus_tag=M1404_00840;product=Lipopolysaccharide export system permease protein LptF;protein_id=gnl|Prokka|M1404_00840

gnl|Prokka|M1404_1 prokka gene 925898 927382 . + . ID=M1404_00841_gene;Name=pepA;gene=pepA;locus_tag=M1404_00841

gnl|Prokka|M1404_1 Prodigal:002006 CDS 925898 927382 . + 0 ID=M1404_00841;Parent=M1404_00841_gene;eC_number=3.4.11.1;Name=pepA;db_xref=COG:COG0260;gene=pepA;inference=ab initio prediction:Prodigal:002006,similar to AA sequence:UniProtKB:P68767;locus_tag=M1404_00841;product=Cytosol aminopeptidase;protein_id=gnl|Prokka|M1404_00841

gnl|Prokka|M1404_1 prokka gene 927474 928163 . - . ID=M1404_00842_gene;Name=mtnN;gene=mtnN;locus_tag=M1404_00842

gnl|Prokka|M1404_1 Prodigal:002006 CDS 927474 928163 . - 0 ID=M1404_00842;Parent=M1404_00842_gene;eC_number=3.2.2.9;Name=mtnN;db_xref=COG:COG0775;gene=mtnN;inference=ab initio prediction:Prodigal:002006,similar to AA sequence:UniProtKB:A6T4W3;locus_tag=M1404_00842;product=5'-methylthioadenosine/S-adenosylhomocysteine nucleosidase;protein_id=gnl|Prokka|M1404_00842

gnl|Prokka|M1404_1 prokka gene 928163 928831 . - . ID=M1404_00843_gene;locus_tag=M1404_00843

gnl|Prokka|M1404_1 Prodigal:002006 CDS 928163 928831 . - 0 ID=M1404_00843;Parent=M1404_00843_gene;inference=ab initio prediction:Prodigal:002006;locus_tag=M1404_00843;product=hypothetical protein;protein_id=gnl|Prokka|M1404_00843

gnl|Prokka|M1404_1 prokka gene 928839 930560 . - . ID=M1404_00844_gene;Name=recJ;gene=recJ;locus_tag=M1404_00844

gnl|Prokka|M1404_1 Prodigal:002006 CDS 928839 930560 . - 0 ID=M1404_00844;Parent=M1404_00844_gene;eC_number=3.1.-.-;Name=recJ;db_xref=COG:COG0608;gene=recJ;inference=ab initio prediction:Prodigal:002006,similar to AA sequence:UniProtKB:P21893;locus_tag=M1404_00844;product=Single-stranded-DNA-specific exonuclease RecJ;protein_id=gnl|Prokka|M1404_00844

gnl|Prokka|M1404_1 prokka gene 930623 931306 . - . ID=M1404_00845_gene;Name=dsbC;gene=dsbC;locus_tag=M1404_00845

gnl|Prokka|M1404_1 Prodigal:002006 CDS 930623 931306 . - 0 ID=M1404_00845;Parent=M1404_00845_gene;Name=dsbC;db_xref=COG:COG1651;gene=dsbC;inference=ab initio prediction:Prodigal:002006,similar to AA sequence:UniProtKB:P45111;locus_tag=M1404_00845;product=Thiol:disulfide interchange protein DsbC;protein_id=gnl|Prokka|M1404_00845

gnl|Prokka|M1404_1 prokka gene 931607 932569 . + . ID=M1404_00846_gene;Name=prfB;gene=prfB;locus_tag=M1404_00846

gnl|Prokka|M1404_1 Prodigal:002006 CDS 931607 932569 . + 0 ID=M1404_00846;Parent=M1404_00846_gene;Name=prfB;db_xref=COG:COG1186;gene=prfB;inference=ab initio prediction:Prodigal:002006,similar to AA sequence:UniProtKB:P07012;locus_tag=M1404_00846;product=Peptide chain release factor RF2;protein_id=gnl|Prokka|M1404_00846

gnl|Prokka|M1404_1 prokka gene 932622 934127 . + . ID=M1404_00847_gene;Name=lysU;gene=lysU;locus_tag=M1404_00847

gnl|Prokka|M1404_1 Prodigal:002006 CDS 932622 934127 . + 0 ID=M1404_00847;Parent=M1404_00847_gene;eC_number=6.1.1.6;Name=lysU;db_xref=COG:COG1190;gene=lysU;inference=ab initio prediction:Prodigal:002006,similar to AA sequence:UniProtKB:P0A8N5;locus_tag=M1404_00847;product=Lysine--tRNA ligase%2C heat inducible;protein_id=gnl|Prokka|M1404_00847

gnl|Prokka|M1404_1 prokka gene 934183 935421 . + . ID=M1404_00848_gene;locus_tag=M1404_00848

gnl|Prokka|M1404_1 Prodigal:002006 CDS 934183 935421 . + 0 ID=M1404_00848;Parent=M1404_00848_gene;inference=ab initio prediction:Prodigal:002006;locus_tag=M1404_00848;product=hypothetical protein;protein_id=gnl|Prokka|M1404_00848

gnl|Prokka|M1404_1 prokka gene 935441 936112 . + . ID=M1404_00849_gene;Name=neuA_1;gene=neuA_1;locus_tag=M1404_00849

gnl|Prokka|M1404_1 Prodigal:002006 CDS 935441 936112 . + 0 ID=M1404_00849;Parent=M1404_00849_gene;eC_number=2.7.7.43;Name=neuA_1;db_xref=COG:COG1083;gene=neuA_1;inference=ab initio prediction:Prodigal:002006,similar to AA sequence:UniProtKB:P0A0Z8;locus_tag=M1404_00849;product=N-acylneuraminate cytidylyltransferase;protein_id=gnl|Prokka|M1404_00849

gnl|Prokka|M1404_1 prokka gene 936259 936945 . + . ID=M1404_00850_gene;Name=queC_1;gene=queC_1;locus_tag=M1404_00850

gnl|Prokka|M1404_1 Prodigal:002006 CDS 936259 936945 . + 0 ID=M1404_00850;Parent=M1404_00850_gene;eC_number=6.3.4.20;Name=queC_1;db_xref=COG:COG0603;gene=queC_1;inference=ab initio prediction:Prodigal:002006,similar to AA sequence:UniProtKB:O31675;locus_tag=M1404_00850;product=7-cyano-7-deazaguanine synthase;protein_id=gnl|Prokka|M1404_00850

gnl|Prokka|M1404_1 prokka gene 936938 937363 . + . ID=M1404_00851_gene;Name=queD;gene=queD;locus_tag=M1404_00851

gnl|Prokka|M1404_1 Prodigal:002006 CDS 936938 937363 . + 0 ID=M1404_00851;Parent=M1404_00851_gene;eC_number=4.1.2.50;Name=queD;db_xref=COG:COG0720;gene=queD;inference=ab initio prediction:Prodigal:002006,similar to AA sequence:UniProtKB:P65870;locus_tag=M1404_00851;product=6-carboxy-5%2C6%2C7%2C8-tetrahydropterin synthase;protein_id=gnl|Prokka|M1404_00851

gnl|Prokka|M1404_1 prokka gene 937369 937998 . + . ID=M1404_00852_gene;Name=queE;gene=queE;locus_tag=M1404_00852

gnl|Prokka|M1404_1 Prodigal:002006 CDS 937369 937998 . + 0 ID=M1404_00852;Parent=M1404_00852_gene;eC_number=4.3.99.3;Name=queE;gene=queE;inference=ab initio prediction:Prodigal:002006,similar to AA sequence:UniProtKB:A0A0H3KB22;locus_tag=M1404_00852;product=7-carboxy-7-deazaguanine synthase;protein_id=gnl|Prokka|M1404_00852

gnl|Prokka|M1404_1 prokka gene 938236 938991 . + . ID=M1404_00853_gene;locus_tag=M1404_00853

gnl|Prokka|M1404_1 Prodigal:002006 CDS 938236 938991 . + 0 ID=M1404_00853;Parent=M1404_00853_gene;db_xref=COG:COG0327;inference=ab initio prediction:Prodigal:002006,similar to AA sequence:UniProtKB:Q57354;locus_tag=M1404_00853;product=GTP cyclohydrolase 1 type 2 ;protein_id=gnl|Prokka|M1404_00853

gnl|Prokka|M1404_1 prokka gene 939079 939861 . + . ID=M1404_00854_gene;Name=fabI;gene=fabI;locus_tag=M1404_00854

gnl|Prokka|M1404_1 Prodigal:002006 CDS 939079 939861 . + 0 ID=M1404_00854;Parent=M1404_00854_gene;eC_number=1.3.1.9;Name=fabI;db_xref=COG:COG0623;gene=fabI;inference=ab initio prediction:Prodigal:002006,similar to AA sequence:UniProtKB:P0AEK4;locus_tag=M1404_00854;product=Enoyl-[acyl-carrier-protein] reductase [NADH] FabI;protein_id=gnl|Prokka|M1404_00854

gnl|Prokka|M1404_1 prokka gene 939922 941898 . + . ID=M1404_00855_gene;Name=rnb;gene=rnb;locus_tag=M1404_00855

gnl|Prokka|M1404_1 Prodigal:002006 CDS 939922 941898 . + 0 ID=M1404_00855;Parent=M1404_00855_gene;eC_number=3.1.13.1;Name=rnb;db_xref=COG:COG4776;gene=rnb;inference=ab initio prediction:Prodigal:002006,similar to AA sequence:UniProtKB:P30850;locus_tag=M1404_00855;product=Exoribonuclease 2;protein_id=gnl|Prokka|M1404_00855

gnl|Prokka|M1404_1 prokka gene 941952 943229 . - . ID=M1404_00856_gene;Name=murA;gene=murA;locus_tag=M1404_00856

gnl|Prokka|M1404_1 Prodigal:002006 CDS 941952 943229 . - 0 ID=M1404_00856;Parent=M1404_00856_gene;eC_number=2.5.1.7;Name=murA;db_xref=COG:COG0766;gene=murA;inference=ab initio prediction:Prodigal:002006,similar to AA sequence:UniProtKB:P45025;locus_tag=M1404_00856;product=UDP-N-acetylglucosamine 1-carboxyvinyltransferase;protein_id=gnl|Prokka|M1404_00856

gnl|Prokka|M1404_1 prokka gene 943250 943507 . - . ID=M1404_00857_gene;locus_tag=M1404_00857

gnl|Prokka|M1404_1 Prodigal:002006 CDS 943250 943507 . - 0 ID=M1404_00857;Parent=M1404_00857_gene;db_xref=COG:COG5007;inference=ab initio prediction:Prodigal:002006,similar to AA sequence:UniProtKB:P45026;locus_tag=M1404_00857;product=putative protein;protein_id=gnl|Prokka|M1404_00857

gnl|Prokka|M1404_1 prokka gene 943489 943866 . - . ID=M1404_00858_gene;locus_tag=M1404_00858

gnl|Prokka|M1404_1 Prodigal:002006 CDS 943489 943866 . - 0 ID=M1404_00858;Parent=M1404_00858_gene;inference=ab initio prediction:Prodigal:002006;locus_tag=M1404_00858;product=hypothetical protein;protein_id=gnl|Prokka|M1404_00858

gnl|Prokka|M1404_1 prokka gene 943871 944509 . - . ID=M1404_00859_gene;Name=mlaC;gene=mlaC;locus_tag=M1404_00859

gnl|Prokka|M1404_1 Prodigal:002006 CDS 943871 944509 . - 0 ID=M1404_00859;Parent=M1404_00859_gene;Name=mlaC;db_xref=COG:COG2854;gene=mlaC;inference=ab initio prediction:Prodigal:002006,similar to AA sequence:UniProtKB:P45028;locus_tag=M1404_00859;product=Intermembrane phospholipid transport system binding protein MlaC;protein_id=gnl|Prokka|M1404_00859

gnl|Prokka|M1404_1 prokka gene 944554 945060 . - . ID=M1404_00860_gene;Name=mlaD;gene=mlaD;locus_tag=M1404_00860

gnl|Prokka|M1404_1 Prodigal:002006 CDS 944554 945060 . - 0 ID=M1404_00860;Parent=M1404_00860_gene;Name=mlaD;db_xref=COG:COG1463;gene=mlaD;inference=ab initio prediction:Prodigal:002006,similar to AA sequence:UniProtKB:P64604;locus_tag=M1404_00860;product=Intermembrane phospholipid transport system binding protein MlaD;protein_id=gnl|Prokka|M1404_00860

gnl|Prokka|M1404_1 prokka gene 945098 945883 . - . ID=M1404_00861_gene;Name=mlaE;gene=mlaE;locus_tag=M1404_00861

gnl|Prokka|M1404_1 Prodigal:002006 CDS 945098 945883 . - 0 ID=M1404_00861;Parent=M1404_00861_gene;Name=mlaE;db_xref=COG:COG0767;gene=mlaE;inference=ab initio prediction:Prodigal:002006,similar to AA sequence:UniProtKB:P64606;locus_tag=M1404_00861;product=Intermembrane phospholipid transport system permease protein MlaE;protein_id=gnl|Prokka|M1404_00861

gnl|Prokka|M1404_1 prokka gene 945880 946674 . - . ID=M1404_00862_gene;Name=mlaF;gene=mlaF;locus_tag=M1404_00862

gnl|Prokka|M1404_1 Prodigal:002006 CDS 945880 946674 . - 0 ID=M1404_00862;Parent=M1404_00862_gene;eC_number=3.6.3.-;Name=mlaF;db_xref=COG:COG1127;gene=mlaF;inference=ab initio prediction:Prodigal:002006,similar to AA sequence:UniProtKB:P45031;locus_tag=M1404_00862;product=Intermembrane phospholipid transport system ATP-binding protein MlaF;protein_id=gnl|Prokka|M1404_00862

gnl|Prokka|M1404_1 prokka gene 946905 947483 . + . ID=M1404_00863_gene;Name=lptC;gene=lptC;locus_tag=M1404_00863

gnl|Prokka|M1404_1 Prodigal:002006 CDS 946905 947483 . + 0 ID=M1404_00863;Parent=M1404_00863_gene;Name=lptC;db_xref=COG:COG3117;gene=lptC;inference=ab initio prediction:Prodigal:002006,similar to AA sequence:UniProtKB:P0ADV9;locus_tag=M1404_00863;product=Lipopolysaccharide export system protein LptC;protein_id=gnl|Prokka|M1404_00863

gnl|Prokka|M1404_1 prokka gene 947467 947979 . + . ID=M1404_00864_gene;Name=lptA;gene=lptA;locus_tag=M1404_00864

gnl|Prokka|M1404_1 Prodigal:002006 CDS 947467 947979 . + 0 ID=M1404_00864;Parent=M1404_00864_gene;Name=lptA;db_xref=COG:COG1934;gene=lptA;inference=ab initio prediction:Prodigal:002006,similar to AA sequence:UniProtKB:P45074;locus_tag=M1404_00864;product=Lipopolysaccharide export system protein LptA;protein_id=gnl|Prokka|M1404_00864

gnl|Prokka|M1404_1 prokka gene 947988 948713 . + . ID=M1404_00865_gene;Name=lptB;gene=lptB;locus_tag=M1404_00865

gnl|Prokka|M1404_1 Prodigal:002006 CDS 947988 948713 . + 0 ID=M1404_00865;Parent=M1404_00865_gene;eC_number=3.6.3.-;Name=lptB;db_xref=COG:COG1137;gene=lptB;inference=ab initio prediction:Prodigal:002006,similar to AA sequence:UniProtKB:P45073;locus_tag=M1404_00865;product=Lipopolysaccharide export system ATP-binding protein LptB;protein_id=gnl|Prokka|M1404_00865

gnl|Prokka|M1404_1 prokka gene 948716 949234 . + . ID=M1404_00866_gene;Name=ptsN;gene=ptsN;locus_tag=M1404_00866

gnl|Prokka|M1404_1 Prodigal:002006 CDS 948716 949234 . + 0 ID=M1404_00866;Parent=M1404_00866_gene;Name=ptsN;db_xref=COG:COG1762;gene=ptsN;inference=ab initio prediction:Prodigal:002006,similar to AA sequence:UniProtKB:P69829;locus_tag=M1404_00866;product=Nitrogen regulatory protein;protein_id=gnl|Prokka|M1404_00866

gnl|Prokka|M1404_1 prokka gene 949264 950130 . + . ID=M1404_00867_gene;Name=rapZ;gene=rapZ;locus_tag=M1404_00867

gnl|Prokka|M1404_1 Prodigal:002006 CDS 949264 950130 . + 0 ID=M1404_00867;Parent=M1404_00867_gene;Name=rapZ;db_xref=COG:COG1660;gene=rapZ;inference=ab initio prediction:Prodigal:002006,similar to AA sequence:UniProtKB:P0A894;locus_tag=M1404_00867;product=RNase adapter protein RapZ;protein_id=gnl|Prokka|M1404_00867

gnl|Prokka|M1404_1 prokka gene 950132 951469 . - . ID=M1404_00868_gene;Name=xseA;gene=xseA;locus_tag=M1404_00868

gnl|Prokka|M1404_1 Prodigal:002006 CDS 950132 951469 . - 0 ID=M1404_00868;Parent=M1404_00868_gene;eC_number=3.1.11.6;Name=xseA;db_xref=COG:COG1570;gene=xseA;inference=ab initio prediction:Prodigal:002006,similar to AA sequence:UniProtKB:P04994;locus_tag=M1404_00868;product=Exodeoxyribonuclease 7 large subunit;protein_id=gnl|Prokka|M1404_00868

gnl|Prokka|M1404_1 prokka gene 951550 952743 . - . ID=M1404_00869_gene;locus_tag=M1404_00869

gnl|Prokka|M1404_1 Prodigal:002006 CDS 951550 952743 . - 0 ID=M1404_00869;Parent=M1404_00869_gene;eC_number=1.14.11.-;db_xref=COG:COG2850;inference=ab initio prediction:Prodigal:002006,similar to AA sequence:UniProtKB:P44683;locus_tag=M1404_00869;product=putative ribosomal oxygenase;protein_id=gnl|Prokka|M1404_00869

gnl|Prokka|M1404_1 prokka gene 952797 953096 . - . ID=M1404_00870_gene;locus_tag=M1404_00870

gnl|Prokka|M1404_1 Prodigal:002006 CDS 952797 953096 . - 0 ID=M1404_00870;Parent=M1404_00870_gene;inference=ab initio prediction:Prodigal:002006,similar to AA sequence:UniProtKB:P43994;locus_tag=M1404_00870;note=UPF0125 protein HI_0395;product=hypothetical protein;protein_id=gnl|Prokka|M1404_00870

gnl|Prokka|M1404_1 prokka gene 953083 953523 . - . ID=M1404_00871_gene;Name=pasT;gene=pasT;locus_tag=M1404_00871

gnl|Prokka|M1404_1 Prodigal:002006 CDS 953083 953523 . - 0 ID=M1404_00871;Parent=M1404_00871_gene;Name=pasT;db_xref=COG:COG2867;gene=pasT;inference=ab initio prediction:Prodigal:002006,similar to AA sequence:UniProtKB:Q8FEY4;locus_tag=M1404_00871;product=Persistence and stress-resistance toxin PasT;protein_id=gnl|Prokka|M1404_00871

gnl|Prokka|M1404_1 prokka gene 953679 954263 . + . ID=M1404_00872_gene;Name=pth;gene=pth;locus_tag=M1404_00872

gnl|Prokka|M1404_1 Prodigal:002006 CDS 953679 954263 . + 0 ID=M1404_00872;Parent=M1404_00872_gene;eC_number=3.1.1.29;Name=pth;gene=pth;inference=ab initio prediction:Prodigal:002006,similar to AA sequence:UniProtKB:C3LPI9;locus_tag=M1404_00872;product=Peptidyl-tRNA hydrolase;protein_id=gnl|Prokka|M1404_00872

gnl|Prokka|M1404_1 prokka gene 954297 955388 . + . ID=M1404_00873_gene;Name=ychF;gene=ychF;locus_tag=M1404_00873

gnl|Prokka|M1404_1 Prodigal:002006 CDS 954297 955388 . + 0 ID=M1404_00873;Parent=M1404_00873_gene;Name=ychF;db_xref=COG:COG0012;gene=ychF;inference=ab initio prediction:Prodigal:002006,similar to AA sequence:UniProtKB:P44681;locus_tag=M1404_00873;product=Ribosome-binding ATPase YchF;protein_id=gnl|Prokka|M1404_00873

gnl|Prokka|M1404_1 prokka gene 955474 956886 . + . ID=M1404_00874_gene;Name=ydgI;gene=ydgI;locus_tag=M1404_00874

gnl|Prokka|M1404_1 Prodigal:002006 CDS 955474 956886 . + 0 ID=M1404_00874;Parent=M1404_00874_gene;Name=ydgI;db_xref=COG:COG0531;gene=ydgI;inference=ab initio prediction:Prodigal:002006,similar to AA sequence:UniProtKB:P0AAE5;locus_tag=M1404_00874;product=Putative arginine/ornithine antiporter;protein_id=gnl|Prokka|M1404_00874

gnl|Prokka|M1404_1 prokka gene 957055 957783 . + . ID=M1404_00875_gene;locus_tag=M1404_00875

gnl|Prokka|M1404_1 Prodigal:002006 CDS 957055 957783 . + 0 ID=M1404_00875;Parent=M1404_00875_gene;inference=ab initio prediction:Prodigal:002006;locus_tag=M1404_00875;product=hypothetical protein;protein_id=gnl|Prokka|M1404_00875

gnl|Prokka|M1404_1 prokka gene 957933 959171 . - . ID=M1404_00876_gene;locus_tag=M1404_00876

gnl|Prokka|M1404_1 Prodigal:002006 CDS 957933 959171 . - 0 ID=M1404_00876;Parent=M1404_00876_gene;eC_number=3.-.-.-;db_xref=COG:COG0624;inference=ab initio prediction:Prodigal:002006,similar to AA sequence:UniProtKB:Q57051;locus_tag=M1404_00876;product=putative hydrolase;protein_id=gnl|Prokka|M1404_00876

gnl|Prokka|M1404_1 prokka gene 959364 959918 . - . ID=M1404_00877_gene;Name=ytfJ;gene=ytfJ;locus_tag=M1404_00877

gnl|Prokka|M1404_1 Prodigal:002006 CDS 959364 959918 . - 0 ID=M1404_00877;Parent=M1404_00877_gene;Name=ytfJ;db_xref=COG:COG3054;gene=ytfJ;inference=ab initio prediction:Prodigal:002006,similar to AA sequence:UniProtKB:P39187;locus_tag=M1404_00877;product=putative protein YtfJ;protein_id=gnl|Prokka|M1404_00877

gnl|Prokka|M1404_1 prokka gene 960061 960393 . + . ID=M1404_00878_gene;locus_tag=M1404_00878

gnl|Prokka|M1404_1 Prodigal:002006 CDS 960061 960393 . + 0 ID=M1404_00878;Parent=M1404_00878_gene;inference=ab initio prediction:Prodigal:002006;locus_tag=M1404_00878;product=hypothetical protein;protein_id=gnl|Prokka|M1404_00878

gnl|Prokka|M1404_1 prokka gene 960437 960649 . - . ID=M1404_00879_gene;locus_tag=M1404_00879

gnl|Prokka|M1404_1 Prodigal:002006 CDS 960437 960649 . - 0 ID=M1404_00879;Parent=M1404_00879_gene;inference=ab initio prediction:Prodigal:002006;locus_tag=M1404_00879;product=hypothetical protein;protein_id=gnl|Prokka|M1404_00879

gnl|Prokka|M1404_1 prokka gene 960843 961262 . + . ID=M1404_00880_gene;Name=rbsD;gene=rbsD;locus_tag=M1404_00880

gnl|Prokka|M1404_1 Prodigal:002006 CDS 960843 961262 . + 0 ID=M1404_00880;Parent=M1404_00880_gene;eC_number=5.4.99.62;Name=rbsD;db_xref=COG:COG1869;gene=rbsD;inference=ab initio prediction:Prodigal:002006,similar to AA sequence:UniProtKB:P04982;locus_tag=M1404_00880;product=D-ribose pyranase;protein_id=gnl|Prokka|M1404_00880

gnl|Prokka|M1404_1 prokka gene 961274 962770 . + . ID=M1404_00881_gene;Name=rbsA_1;gene=rbsA_1;locus_tag=M1404_00881

gnl|Prokka|M1404_1 Prodigal:002006 CDS 961274 962770 . + 0 ID=M1404_00881;Parent=M1404_00881_gene;eC_number=7.5.2.7;Name=rbsA_1;db_xref=COG:COG1129;gene=rbsA_1;inference=ab initio prediction:Prodigal:002006,similar to AA sequence:UniProtKB:P04983;locus_tag=M1404_00881;product=Ribose import ATP-binding protein RbsA;protein_id=gnl|Prokka|M1404_00881

gnl|Prokka|M1404_1 prokka gene 962787 963755 . + . ID=M1404_00882_gene;Name=rbsC_1;gene=rbsC_1;locus_tag=M1404_00882

gnl|Prokka|M1404_1 Prodigal:002006 CDS 962787 963755 . + 0 ID=M1404_00882;Parent=M1404_00882_gene;Name=rbsC_1;db_xref=COG:COG1172;gene=rbsC_1;inference=ab initio prediction:Prodigal:002006,similar to AA sequence:UniProtKB:P0AGI1;locus_tag=M1404_00882;product=Ribose import permease protein RbsC;protein_id=gnl|Prokka|M1404_00882

gnl|Prokka|M1404_1 prokka gene 963783 964670 . + . ID=M1404_00883_gene;Name=rbsB_1;gene=rbsB_1;locus_tag=M1404_00883

gnl|Prokka|M1404_1 Prodigal:002006 CDS 963783 964670 . + 0 ID=M1404_00883;Parent=M1404_00883_gene;Name=rbsB_1;db_xref=COG:COG1879;gene=rbsB_1;inference=ab initio prediction:Prodigal:002006,similar to AA sequence:UniProtKB:P44737;locus_tag=M1404_00883;product=Ribose import binding protein RbsB;protein_id=gnl|Prokka|M1404_00883

gnl|Prokka|M1404_1 prokka gene 964754 965674 . + . ID=M1404_00884_gene;Name=rbsK;gene=rbsK;locus_tag=M1404_00884

gnl|Prokka|M1404_1 Prodigal:002006 CDS 964754 965674 . + 0 ID=M1404_00884;Parent=M1404_00884_gene;eC_number=2.7.1.15;Name=rbsK;db_xref=COG:COG0524;gene=rbsK;inference=ab initio prediction:Prodigal:002006,similar to AA sequence:UniProtKB:P0A9J6;locus_tag=M1404_00884;product=Ribokinase;protein_id=gnl|Prokka|M1404_00884

gnl|Prokka|M1404_1 prokka gene 965757 966770 . + . ID=M1404_00885_gene;Name=rbsR;gene=rbsR;locus_tag=M1404_00885

gnl|Prokka|M1404_1 Prodigal:002006 CDS 965757 966770 . + 0 ID=M1404_00885;Parent=M1404_00885_gene;Name=rbsR;db_xref=COG:COG1609;gene=rbsR;inference=ab initio prediction:Prodigal:002006,similar to AA sequence:UniProtKB:P0ACQ0;locus_tag=M1404_00885;product=Ribose operon repressor;protein_id=gnl|Prokka|M1404_00885

gnl|Prokka|M1404_1 prokka gene 966785 967948 . - . ID=M1404_00886_gene;Name=pheA;gene=pheA;locus_tag=M1404_00886

gnl|Prokka|M1404_1 Prodigal:002006 CDS 966785 967948 . - 0 ID=M1404_00886;Parent=M1404_00886_gene;Name=pheA;db_xref=COG:COG0077;gene=pheA;inference=ab initio prediction:Prodigal:002006,similar to AA sequence:UniProtKB:P0A9J8;locus_tag=M1404_00886;product=Bifunctional chorismate mutase/prephenate dehydratase;protein_id=gnl|Prokka|M1404_00886

gnl|Prokka|M1404_1 prokka gene 968003 969154 . - . ID=M1404_00887_gene;Name=yqhD;gene=yqhD;locus_tag=M1404_00887

gnl|Prokka|M1404_1 Prodigal:002006 CDS 968003 969154 . - 0 ID=M1404_00887;Parent=M1404_00887_gene;eC_number=1.1.1.-;Name=yqhD;db_xref=COG:COG1979;gene=yqhD;inference=ab initio prediction:Prodigal:002006,similar to AA sequence:UniProtKB:Q46856;locus_tag=M1404_00887;product=Alcohol dehydrogenase YqhD;protein_id=gnl|Prokka|M1404_00887

gnl|Prokka|M1404_1 prokka gene 969307 970224 . - . ID=M1404_00888_gene;Name=lpxC;gene=lpxC;locus_tag=M1404_00888

gnl|Prokka|M1404_1 Prodigal:002006 CDS 969307 970224 . - 0 ID=M1404_00888;Parent=M1404_00888_gene;eC_number=3.5.1.108;Name=lpxC;db_xref=COG:COG0774;gene=lpxC;inference=ab initio prediction:Prodigal:002006,similar to AA sequence:UniProtKB:P0A725;locus_tag=M1404_00888;product=UDP-3-O-acyl-N-acetylglucosamine deacetylase;protein_id=gnl|Prokka|M1404_00888

gnl|Prokka|M1404_1 prokka gene 970270 971574 . - . ID=M1404_00889_gene;Name=ftsZ;gene=ftsZ;locus_tag=M1404_00889

gnl|Prokka|M1404_1 Prodigal:002006 CDS 970270 971574 . - 0 ID=M1404_00889;Parent=M1404_00889_gene;Name=ftsZ;gene=ftsZ;inference=ab initio prediction:Prodigal:002006,protein motif:HAMAP:MF_00909;locus_tag=M1404_00889;product=Cell division protein FtsZ;protein_id=gnl|Prokka|M1404_00889

gnl|Prokka|M1404_1 prokka gene 971659 972939 . - . ID=M1404_00890_gene;Name=ftsA;gene=ftsA;locus_tag=M1404_00890

gnl|Prokka|M1404_1 Prodigal:002006 CDS 971659 972939 . - 0 ID=M1404_00890;Parent=M1404_00890_gene;Name=ftsA;db_xref=COG:COG0849;gene=ftsA;inference=ab initio prediction:Prodigal:002006,similar to AA sequence:UniProtKB:P0ABH0;locus_tag=M1404_00890;product=Cell division protein FtsA;protein_id=gnl|Prokka|M1404_00890

gnl|Prokka|M1404_1 prokka gene 972965 973741 . - . ID=M1404_00891_gene;Name=ftsQ;gene=ftsQ;locus_tag=M1404_00891

gnl|Prokka|M1404_1 Prodigal:002006 CDS 972965 973741 . - 0 ID=M1404_00891;Parent=M1404_00891_gene;Name=ftsQ;gene=ftsQ;inference=ab initio prediction:Prodigal:002006,protein motif:HAMAP:MF_00911;locus_tag=M1404_00891;product=Cell division protein FtsQ;protein_id=gnl|Prokka|M1404_00891

gnl|Prokka|M1404_1 prokka gene 973738 974667 . - . ID=M1404_00892_gene;Name=ddlB;gene=ddlB;locus_tag=M1404_00892

gnl|Prokka|M1404_1 Prodigal:002006 CDS 973738 974667 . - 0 ID=M1404_00892;Parent=M1404_00892_gene;eC_number=6.3.2.4;Name=ddlB;db_xref=COG:COG1181;gene=ddlB;inference=ab initio prediction:Prodigal:002006,similar to AA sequence:UniProtKB:P07862;locus_tag=M1404_00892;product=D-alanine--D-alanine ligase B;protein_id=gnl|Prokka|M1404_00892

gnl|Prokka|M1404_1 prokka gene 974676 976124 . - . ID=M1404_00893_gene;Name=murC;gene=murC;locus_tag=M1404_00893

gnl|Prokka|M1404_1 Prodigal:002006 CDS 974676 976124 . - 0 ID=M1404_00893;Parent=M1404_00893_gene;eC_number=6.3.2.8;Name=murC;db_xref=COG:COG0773;gene=murC;inference=ab initio prediction:Prodigal:002006,similar to AA sequence:UniProtKB:P45066;locus_tag=M1404_00893;product=UDP-N-acetylmuramate--L-alanine ligase;protein_id=gnl|Prokka|M1404_00893

gnl|Prokka|M1404_1 prokka gene 976135 977199 . - . ID=M1404_00894_gene;Name=murG;gene=murG;locus_tag=M1404_00894

gnl|Prokka|M1404_1 Prodigal:002006 CDS 976135 977199 . - 0 ID=M1404_00894;Parent=M1404_00894_gene;eC_number=2.4.1.227;Name=murG;db_xref=COG:COG0707;gene=murG;inference=ab initio prediction:Prodigal:002006,similar to AA sequence:UniProtKB:P17443;locus_tag=M1404_00894;product=UDP-N-acetylglucosamine--N-acetylmuramyl-(pentapeptide) pyrophosphoryl-undecaprenol N-acetylglucosamine transferase;protein_id=gnl|Prokka|M1404_00894

gnl|Prokka|M1404_1 prokka gene 977277 978467 . - . ID=M1404_00895_gene;Name=ftsW;gene=ftsW;locus_tag=M1404_00895

gnl|Prokka|M1404_1 Prodigal:002006 CDS 977277 978467 . - 0 ID=M1404_00895;Parent=M1404_00895_gene;eC_number=2.4.1.129;Name=ftsW;db_xref=COG:COG0772;gene=ftsW;inference=ab initio prediction:Prodigal:002006,similar to AA sequence:UniProtKB:P0ABG4;locus_tag=M1404_00895;product=putative peptidoglycan glycosyltransferase FtsW;protein_id=gnl|Prokka|M1404_00895

gnl|Prokka|M1404_1 prokka gene 978479 979783 . - . ID=M1404_00896_gene;Name=murD;gene=murD;locus_tag=M1404_00896

gnl|Prokka|M1404_1 Prodigal:002006 CDS 978479 979783 . - 0 ID=M1404_00896;Parent=M1404_00896_gene;eC_number=6.3.2.9;Name=murD;db_xref=COG:COG0771;gene=murD;inference=ab initio prediction:Prodigal:002006,similar to AA sequence:UniProtKB:P14900;locus_tag=M1404_00896;product=UDP-N-acetylmuramoylalanine--D-glutamate ligase;protein_id=gnl|Prokka|M1404_00896

gnl|Prokka|M1404_1 prokka gene 979802 980884 . - . ID=M1404_00897_gene;Name=mraY;gene=mraY;locus_tag=M1404_00897

gnl|Prokka|M1404_1 Prodigal:002006 CDS 979802 980884 . - 0 ID=M1404_00897;Parent=M1404_00897_gene;eC_number=2.7.8.13;Name=mraY;db_xref=COG:COG0472;gene=mraY;inference=ab initio prediction:Prodigal:002006,similar to AA sequence:UniProtKB:P0A6W3;locus_tag=M1404_00897;product=Phospho-N-acetylmuramoyl-pentapeptide-transferase;protein_id=gnl|Prokka|M1404_00897

gnl|Prokka|M1404_1 prokka gene 980878 982260 . - . ID=M1404_00898_gene;Name=murF;gene=murF;locus_tag=M1404_00898

gnl|Prokka|M1404_1 Prodigal:002006 CDS 980878 982260 . - 0 ID=M1404_00898;Parent=M1404_00898_gene;eC_number=6.3.2.10;Name=murF;db_xref=COG:COG0770;gene=murF;inference=ab initio prediction:Prodigal:002006,similar to AA sequence:UniProtKB:P11880;locus_tag=M1404_00898;product=UDP-N-acetylmuramoyl-tripeptide--D-alanyl-D-alanine ligase;protein_id=gnl|Prokka|M1404_00898

gnl|Prokka|M1404_1 prokka gene 982253 983737 . - . ID=M1404_00899_gene;Name=murE;gene=murE;locus_tag=M1404_00899

gnl|Prokka|M1404_1 Prodigal:002006 CDS 982253 983737 . - 0 ID=M1404_00899;Parent=M1404_00899_gene;eC_number=6.3.2.13;Name=murE;db_xref=COG:COG0769;gene=murE;inference=ab initio prediction:Prodigal:002006,similar to AA sequence:UniProtKB:P22188;locus_tag=M1404_00899;product=UDP-N-acetylmuramoyl-L-alanyl-D-glutamate--2%2C6-diaminopimelate ligase;protein_id=gnl|Prokka|M1404_00899

gnl|Prokka|M1404_1 prokka gene 983747 985552 . - . ID=M1404_00900_gene;Name=ftsI;gene=ftsI;locus_tag=M1404_00900

gnl|Prokka|M1404_1 Prodigal:002006 CDS 983747 985552 . - 0 ID=M1404_00900;Parent=M1404_00900_gene;eC_number=3.4.16.4;Name=ftsI;db_xref=COG:COG0768;gene=ftsI;inference=ab initio prediction:Prodigal:002006,similar to AA sequence:UniProtKB:P0AD68;locus_tag=M1404_00900;product=Peptidoglycan D%2CD-transpeptidase FtsI;protein_id=gnl|Prokka|M1404_00900

gnl|Prokka|M1404_1 prokka gene 985570 985887 . - . ID=M1404_00901_gene;Name=ftsL;gene=ftsL;locus_tag=M1404_00901

gnl|Prokka|M1404_1 Prodigal:002006 CDS 985570 985887 . - 0 ID=M1404_00901;Parent=M1404_00901_gene;Name=ftsL;db_xref=COG:COG3116;gene=ftsL;inference=ab initio prediction:Prodigal:002006,similar to AA sequence:UniProtKB:P0AEN4;locus_tag=M1404_00901;product=Cell division protein FtsL;protein_id=gnl|Prokka|M1404_00901

gnl|Prokka|M1404_1 prokka gene 985887 986852 . - . ID=M1404_00902_gene;Name=rsmH;gene=rsmH;locus_tag=M1404_00902

gnl|Prokka|M1404_1 Prodigal:002006 CDS 985887 986852 . - 0 ID=M1404_00902;Parent=M1404_00902_gene;eC_number=2.1.1.199;Name=rsmH;db_xref=COG:COG0275;gene=rsmH;inference=ab initio prediction:Prodigal:002006,similar to AA sequence:UniProtKB:P45057;locus_tag=M1404_00902;product=Ribosomal RNA small subunit methyltransferase H;protein_id=gnl|Prokka|M1404_00902

gnl|Prokka|M1404_1 prokka gene 986974 987432 . - . ID=M1404_00903_gene;Name=mraZ;gene=mraZ;locus_tag=M1404_00903

gnl|Prokka|M1404_1 Prodigal:002006 CDS 986974 987432 . - 0 ID=M1404_00903;Parent=M1404_00903_gene;Name=mraZ;db_xref=COG:COG2001;gene=mraZ;inference=ab initio prediction:Prodigal:002006,similar to AA sequence:UniProtKB:P22186;locus_tag=M1404_00903;product=Transcriptional regulator MraZ;protein_id=gnl|Prokka|M1404_00903

gnl|Prokka|M1404_1 prokka gene 987715 989259 . - . ID=M1404_00904_gene;locus_tag=M1404_00904

gnl|Prokka|M1404_1 Prodigal:002006 CDS 987715 989259 . - 0 ID=M1404_00904;Parent=M1404_00904_gene;inference=ab initio prediction:Prodigal:002006;locus_tag=M1404_00904;product=hypothetical protein;protein_id=gnl|Prokka|M1404_00904

gnl|Prokka|M1404_1 prokka gene 989562 990458 . + . ID=M1404_00905_gene;Name=fecB;gene=fecB;locus_tag=M1404_00905

gnl|Prokka|M1404_1 Prodigal:002006 CDS 989562 990458 . + 0 ID=M1404_00905;Parent=M1404_00905_gene;Name=fecB;db_xref=COG:COG4594;gene=fecB;inference=ab initio prediction:Prodigal:002006,similar to AA sequence:UniProtKB:P15028;locus_tag=M1404_00905;product=Fe(3+) dicitrate-binding periplasmic protein;protein_id=gnl|Prokka|M1404_00905

gnl|Prokka|M1404_1 prokka gene 990458 991441 . + . ID=M1404_00906_gene;Name=fecC;gene=fecC;locus_tag=M1404_00906

gnl|Prokka|M1404_1 Prodigal:002006 CDS 990458 991441 . + 0 ID=M1404_00906;Parent=M1404_00906_gene;Name=fecC;db_xref=COG:COG0609;gene=fecC;inference=ab initio prediction:Prodigal:002006,similar to AA sequence:UniProtKB:P15030;locus_tag=M1404_00906;product=Fe(3+) dicitrate transport system permease protein FecC;protein_id=gnl|Prokka|M1404_00906

gnl|Prokka|M1404_1 prokka gene 991441 992433 . + . ID=M1404_00907_gene;Name=fecD;gene=fecD;locus_tag=M1404_00907

gnl|Prokka|M1404_1 Prodigal:002006 CDS 991441 992433 . + 0 ID=M1404_00907;Parent=M1404_00907_gene;Name=fecD;db_xref=COG:COG0609;gene=fecD;inference=ab initio prediction:Prodigal:002006,similar to AA sequence:UniProtKB:P15029;locus_tag=M1404_00907;product=Fe(3+) dicitrate transport system permease protein FecD;protein_id=gnl|Prokka|M1404_00907

gnl|Prokka|M1404_1 prokka gene 992433 993206 . + . ID=M1404_00908_gene;Name=fecE;gene=fecE;locus_tag=M1404_00908

gnl|Prokka|M1404_1 Prodigal:002006 CDS 992433 993206 . + 0 ID=M1404_00908;Parent=M1404_00908_gene;Name=fecE;db_xref=COG:COG1120;gene=fecE;inference=ab initio prediction:Prodigal:002006,similar to AA sequence:UniProtKB:P15031;locus_tag=M1404_00908;product=Fe(3+) dicitrate transport ATP-binding protein FecE;protein_id=gnl|Prokka|M1404_00908

gnl|Prokka|M1404_1 prokka gene 993296 993526 . + . ID=M1404_00909_gene;locus_tag=M1404_00909

gnl|Prokka|M1404_1 Prodigal:002006 CDS 993296 993526 . + 0 ID=M1404_00909;Parent=M1404_00909_gene;inference=ab initio prediction:Prodigal:002006;locus_tag=M1404_00909;product=hypothetical protein;protein_id=gnl|Prokka|M1404_00909

gnl|Prokka|M1404_1 prokka gene 993585 994268 . - . ID=M1404_00910_gene;Name=artM;gene=artM;locus_tag=M1404_00910

gnl|Prokka|M1404_1 Prodigal:002006 CDS 993585 994268 . - 0 ID=M1404_00910;Parent=M1404_00910_gene;Name=artM;db_xref=COG:COG4160;gene=artM;inference=ab initio prediction:Prodigal:002006,similar to AA sequence:UniProtKB:P0AE30;locus_tag=M1404_00910;product=Arginine ABC transporter permease protein ArtM;protein_id=gnl|Prokka|M1404_00910

gnl|Prokka|M1404_1 prokka gene 994268 994939 . - . ID=M1404_00911_gene;Name=artQ;gene=artQ;locus_tag=M1404_00911

gnl|Prokka|M1404_1 Prodigal:002006 CDS 994268 994939 . - 0 ID=M1404_00911;Parent=M1404_00911_gene;Name=artQ;db_xref=COG:COG4215;gene=artQ;inference=ab initio prediction:Prodigal:002006,similar to AA sequence:UniProtKB:P0AE34;locus_tag=M1404_00911;product=Arginine ABC transporter permease protein ArtQ;protein_id=gnl|Prokka|M1404_00911

gnl|Prokka|M1404_1 prokka gene 994946 995668 . - . ID=M1404_00912_gene;Name=artI;gene=artI;locus_tag=M1404_00912

gnl|Prokka|M1404_1 Prodigal:002006 CDS 994946 995668 . - 0 ID=M1404_00912;Parent=M1404_00912_gene;Name=artI;db_xref=COG:COG0834;gene=artI;inference=ab initio prediction:Prodigal:002006,similar to AA sequence:UniProtKB:P45091;locus_tag=M1404_00912;product=ABC transporter arginine-binding protein;protein_id=gnl|Prokka|M1404_00912

gnl|Prokka|M1404_1 prokka gene 995685 996416 . - . ID=M1404_00913_gene;Name=artP;gene=artP;locus_tag=M1404_00913

gnl|Prokka|M1404_1 Prodigal:002006 CDS 995685 996416 . - 0 ID=M1404_00913;Parent=M1404_00913_gene;eC_number=3.6.3.-;Name=artP;db_xref=COG:COG4161;gene=artP;inference=ab initio prediction:Prodigal:002006,similar to AA sequence:UniProtKB:P0AAF6;locus_tag=M1404_00913;product=Arginine transport ATP-binding protein ArtP;protein_id=gnl|Prokka|M1404_00913

gnl|Prokka|M1404_1 prokka gene 996592 997203 . - . ID=M1404_00914_gene;Name=gmhA_2;gene=gmhA_2;locus_tag=M1404_00914

gnl|Prokka|M1404_1 Prodigal:002006 CDS 996592 997203 . - 0 ID=M1404_00914;Parent=M1404_00914_gene;eC_number=5.3.1.28;Name=gmhA_2;db_xref=COG:COG0279;gene=gmhA_2;inference=ab initio prediction:Prodigal:002006,similar to AA sequence:UniProtKB:P63224;locus_tag=M1404_00914;product=Phosphoheptose isomerase;protein_id=gnl|Prokka|M1404_00914

gnl|Prokka|M1404_1 prokka gene 997209 997748 . - . ID=M1404_00915_gene;Name=hpt;gene=hpt;locus_tag=M1404_00915

gnl|Prokka|M1404_1 Prodigal:002006 CDS 997209 997748 . - 0 ID=M1404_00915;Parent=M1404_00915_gene;eC_number=2.4.2.8;Name=hpt;db_xref=COG:COG0634;gene=hpt;inference=ab initio prediction:Prodigal:002006,similar to AA sequence:UniProtKB:O33799;locus_tag=M1404_00915;product=Hypoxanthine phosphoribosyltransferase;protein_id=gnl|Prokka|M1404_00915

gnl|Prokka|M1404_1 prokka gene 997983 999338 . - . ID=M1404_00916_gene;Name=pmbA;gene=pmbA;locus_tag=M1404_00916

gnl|Prokka|M1404_1 Prodigal:002006 CDS 997983 999338 . - 0 ID=M1404_00916;Parent=M1404_00916_gene;eC_number=3.4.-.-;Name=pmbA;db_xref=COG:COG0312;gene=pmbA;inference=ab initio prediction:Prodigal:002006,similar to AA sequence:UniProtKB:P0AFK0;locus_tag=M1404_00916;product=Metalloprotease PmbA;protein_id=gnl|Prokka|M1404_00916

gnl|Prokka|M1404_1 prokka gene 999433 999972 . + . ID=M1404_00917_gene;locus_tag=M1404_00917

gnl|Prokka|M1404_1 Prodigal:002006 CDS 999433 999972 . + 0 ID=M1404_00917;Parent=M1404_00917_gene;inference=ab initio prediction:Prodigal:002006,similar to AA sequence:UniProtKB:P0A8X0;locus_tag=M1404_00917;note=UPF0307 protein YjgA;product=hypothetical protein;protein_id=gnl|Prokka|M1404_00917

gnl|Prokka|M1404_1 prokka gene 1000029 1000916 . - . ID=M1404_00918_gene;Name=hflC;gene=hflC;locus_tag=M1404_00918

gnl|Prokka|M1404_1 Prodigal:002006 CDS 1000029 1000916 . - 0 ID=M1404_00918;Parent=M1404_00918_gene;Name=hflC;db_xref=COG:COG0330;gene=hflC;inference=ab initio prediction:Prodigal:002006,similar to AA sequence:UniProtKB:P0ABC3;locus_tag=M1404_00918;product=Modulator of FtsH protease HflC;protein_id=gnl|Prokka|M1404_00918

gnl|Prokka|M1404_1 prokka gene 1000916 1002163 . - . ID=M1404_00919_gene;Name=hflK;gene=hflK;locus_tag=M1404_00919

gnl|Prokka|M1404_1 Prodigal:002006 CDS 1000916 1002163 . - 0 ID=M1404_00919;Parent=M1404_00919_gene;Name=hflK;db_xref=COG:COG0330;gene=hflK;inference=ab initio prediction:Prodigal:002006,similar to AA sequence:UniProtKB:P0ABC7;locus_tag=M1404_00919;product=Modulator of FtsH protease HflK;protein_id=gnl|Prokka|M1404_00919

gnl|Prokka|M1404_1 prokka gene 1002360 1003082 . - . ID=M1404_00920_gene;locus_tag=M1404_00920

gnl|Prokka|M1404_1 Prodigal:002006 CDS 1002360 1003082 . - 0 ID=M1404_00920;Parent=M1404_00920_gene;inference=ab initio prediction:Prodigal:002006;locus_tag=M1404_00920;product=hypothetical protein;protein_id=gnl|Prokka|M1404_00920

gnl|Prokka|M1404_1 prokka gene 1003195 1004469 . - . ID=M1404_00921_gene;Name=thrC;gene=thrC;locus_tag=M1404_00921

gnl|Prokka|M1404_1 Prodigal:002006 CDS 1003195 1004469 . - 0 ID=M1404_00921;Parent=M1404_00921_gene;eC_number=4.2.3.1;Name=thrC;db_xref=COG:COG0498;gene=thrC;inference=ab initio prediction:Prodigal:002006,similar to AA sequence:UniProtKB:P00934;locus_tag=M1404_00921;product=Threonine synthase;protein_id=gnl|Prokka|M1404_00921

gnl|Prokka|M1404_1 prokka gene 1004489 1005445 . - . ID=M1404_00922_gene;Name=thrB;gene=thrB;locus_tag=M1404_00922

gnl|Prokka|M1404_1 Prodigal:002006 CDS 1004489 1005445 . - 0 ID=M1404_00922;Parent=M1404_00922_gene;eC_number=2.7.1.39;Name=thrB;gene=thrB;inference=ab initio prediction:Prodigal:002006,similar to AA sequence:UniProtKB:Q1CMW6;locus_tag=M1404_00922;product=Homoserine kinase;protein_id=gnl|Prokka|M1404_00922

gnl|Prokka|M1404_1 prokka gene 1005457 1007904 . - . ID=M1404_00923_gene;Name=thrA;gene=thrA;locus_tag=M1404_00923

gnl|Prokka|M1404_1 Prodigal:002006 CDS 1005457 1007904 . - 0 ID=M1404_00923;Parent=M1404_00923_gene;Name=thrA;db_xref=COG:COG0460;gene=thrA;inference=ab initio prediction:Prodigal:002006,similar to AA sequence:UniProtKB:P00561;locus_tag=M1404_00923;product=Bifunctional aspartokinase/homoserine dehydrogenase 1;protein_id=gnl|Prokka|M1404_00923

gnl|Prokka|M1404_1 prokka gene 1008261 1008962 . + . ID=M1404_00924_gene;locus_tag=M1404_00924

gnl|Prokka|M1404_1 Prodigal:002006 CDS 1008261 1008962 . + 0 ID=M1404_00924;Parent=M1404_00924_gene;db_xref=COG:COG0325;inference=ab initio prediction:Prodigal:002006,similar to AA sequence:UniProtKB:P44506;locus_tag=M1404_00924;product=Pyridoxal phosphate homeostasis protein;protein_id=gnl|Prokka|M1404_00924

gnl|Prokka|M1404_1 prokka gene 1008996 1009559 . + . ID=M1404_00925_gene;locus_tag=M1404_00925

gnl|Prokka|M1404_1 Prodigal:002006 CDS 1008996 1009559 . + 0 ID=M1404_00925;Parent=M1404_00925_gene;inference=ab initio prediction:Prodigal:002006;locus_tag=M1404_00925;product=hypothetical protein;protein_id=gnl|Prokka|M1404_00925

gnl|Prokka|M1404_1 prokka gene 1009801 1010502 . - . ID=M1404_00926_gene;locus_tag=M1404_00926

gnl|Prokka|M1404_1 Prodigal:002006 CDS 1009801 1010502 . - 0 ID=M1404_00926;Parent=M1404_00926_gene;inference=ab initio prediction:Prodigal:002006;locus_tag=M1404_00926;product=hypothetical protein;protein_id=gnl|Prokka|M1404_00926

gnl|Prokka|M1404_1 prokka gene 1010989 1011726 . - . ID=M1404_00927_gene;locus_tag=M1404_00927

gnl|Prokka|M1404_1 Prodigal:002006 CDS 1010989 1011726 . - 0 ID=M1404_00927;Parent=M1404_00927_gene;inference=ab initio prediction:Prodigal:002006;locus_tag=M1404_00927;product=hypothetical protein;protein_id=gnl|Prokka|M1404_00927

gnl|Prokka|M1404_1 prokka gene 1012137 1012976 . + . ID=M1404_00928_gene;locus_tag=M1404_00928

gnl|Prokka|M1404_1 Prodigal:002006 CDS 1012137 1012976 . + 0 ID=M1404_00928;Parent=M1404_00928_gene;inference=ab initio prediction:Prodigal:002006;locus_tag=M1404_00928;product=hypothetical protein;protein_id=gnl|Prokka|M1404_00928

gnl|Prokka|M1404_1 prokka gene 1013040 1013504 . - . ID=M1404_00929_gene;Name=rnhA;gene=rnhA;locus_tag=M1404_00929

gnl|Prokka|M1404_1 Prodigal:002006 CDS 1013040 1013504 . - 0 ID=M1404_00929;Parent=M1404_00929_gene;eC_number=3.1.26.4;Name=rnhA;db_xref=COG:COG0328;gene=rnhA;inference=ab initio prediction:Prodigal:002006,similar to AA sequence:UniProtKB:P0A7Y4;locus_tag=M1404_00929;product=Ribonuclease HI;protein_id=gnl|Prokka|M1404_00929

gnl|Prokka|M1404_1 prokka gene 1013573 1014334 . + . ID=M1404_00930_gene;Name=dnaQ;gene=dnaQ;locus_tag=M1404_00930

gnl|Prokka|M1404_1 Prodigal:002006 CDS 1013573 1014334 . + 0 ID=M1404_00930;Parent=M1404_00930_gene;eC_number=2.7.7.7;Name=dnaQ;db_xref=COG:COG0847;gene=dnaQ;inference=ab initio prediction:Prodigal:002006,similar to AA sequence:UniProtKB:P03007;locus_tag=M1404_00930;product=DNA polymerase III subunit epsilon;protein_id=gnl|Prokka|M1404_00930

gnl|Prokka|M1404_1 prokka gene 1014428 1014504 . + . ID=M1404_00931_gene;locus_tag=M1404_00931

gnl|Prokka|M1404_1 Aragorn:001002 tRNA 1014428 1014504 . + . ID=M1404_00931;Parent=M1404_00931_gene;inference=COORDINATES:profile:Aragorn:001002;locus_tag=M1404_00931;product=tRNA-Asp(gtc)

gnl|Prokka|M1404_1 prokka gene 1014520 1014596 . + . ID=M1404_00932_gene;locus_tag=M1404_00932

gnl|Prokka|M1404_1 Aragorn:001002 tRNA 1014520 1014596 . + . ID=M1404_00932;Parent=M1404_00932_gene;inference=COORDINATES:profile:Aragorn:001002;locus_tag=M1404_00932;product=tRNA-Asp(gtc)

gnl|Prokka|M1404_1 prokka gene 1014782 1016314 . + . ID=M1404_00933_gene;Name=der;gene=der;locus_tag=M1404_00933

gnl|Prokka|M1404_1 Prodigal:002006 CDS 1014782 1016314 . + 0 ID=M1404_00933;Parent=M1404_00933_gene;Name=der;db_xref=COG:COG1160;gene=der;inference=ab initio prediction:Prodigal:002006,similar to AA sequence:UniProtKB:Q9XCI8;locus_tag=M1404_00933;product=GTPase Der;protein_id=gnl|Prokka|M1404_00933

gnl|Prokka|M1404_1 prokka gene 1016378 1016767 . + . ID=M1404_00934_gene;locus_tag=M1404_00934

gnl|Prokka|M1404_1 Prodigal:002006 CDS 1016378 1016767 . + 0 ID=M1404_00934;Parent=M1404_00934_gene;inference=ab initio prediction:Prodigal:002006;locus_tag=M1404_00934;product=hypothetical protein;protein_id=gnl|Prokka|M1404_00934

gnl|Prokka|M1404_1 prokka gene 1016820 1018226 . - . ID=M1404_00935_gene;Name=ftsP;gene=ftsP;locus_tag=M1404_00935

gnl|Prokka|M1404_1 Prodigal:002006 CDS 1016820 1018226 . - 0 ID=M1404_00935;Parent=M1404_00935_gene;Name=ftsP;db_xref=COG:COG2132;gene=ftsP;inference=ab initio prediction:Prodigal:002006,similar to AA sequence:UniProtKB:P26648;locus_tag=M1404_00935;product=Cell division protein FtsP;protein_id=gnl|Prokka|M1404_00935

gnl|Prokka|M1404_1 prokka gene 1018230 1018955 . - . ID=M1404_00936_gene;Name=plsC;gene=plsC;locus_tag=M1404_00936

gnl|Prokka|M1404_1 Prodigal:002006 CDS 1018230 1018955 . - 0 ID=M1404_00936;Parent=M1404_00936_gene;eC_number=2.3.1.51;Name=plsC;db_xref=COG:COG0204;gene=plsC;inference=ab initio prediction:Prodigal:002006,similar to AA sequence:UniProtKB:P26647;locus_tag=M1404_00936;product=1-acyl-sn-glycerol-3-phosphate acyltransferase;protein_id=gnl|Prokka|M1404_00936

gnl|Prokka|M1404_1 prokka gene 1019066 1019785 . + . ID=M1404_00937_gene;Name=lpxH;gene=lpxH;locus_tag=M1404_00937

gnl|Prokka|M1404_1 Prodigal:002006 CDS 1019066 1019785 . + 0 ID=M1404_00937;Parent=M1404_00937_gene;eC_number=3.6.1.54;Name=lpxH;db_xref=COG:COG2908;gene=lpxH;inference=ab initio prediction:Prodigal:002006,similar to AA sequence:UniProtKB:P44046;locus_tag=M1404_00937;product=UDP-2%2C3-diacylglucosamine hydrolase;protein_id=gnl|Prokka|M1404_00937

gnl|Prokka|M1404_1 prokka gene 1019789 1020295 . + . ID=M1404_00938_gene;locus_tag=M1404_00938

gnl|Prokka|M1404_1 Prodigal:002006 CDS 1019789 1020295 . + 0 ID=M1404_00938;Parent=M1404_00938_gene;inference=ab initio prediction:Prodigal:002006;locus_tag=M1404_00938;product=hypothetical protein;protein_id=gnl|Prokka|M1404_00938

gnl|Prokka|M1404_1 prokka gene 1020344 1020910 . - . ID=M1404_00939_gene;Name=efp;gene=efp;locus_tag=M1404_00939

gnl|Prokka|M1404_1 Prodigal:002006 CDS 1020344 1020910 . - 0 ID=M1404_00939;Parent=M1404_00939_gene;Name=efp;db_xref=COG:COG0231;gene=efp;inference=ab initio prediction:Prodigal:002006,similar to AA sequence:UniProtKB:P64036;locus_tag=M1404_00939;product=Elongation factor P;protein_id=gnl|Prokka|M1404_00939

gnl|Prokka|M1404_1 prokka gene 1020946 1021959 . + . ID=M1404_00940_gene;Name=epmB;gene=epmB;locus_tag=M1404_00940

gnl|Prokka|M1404_1 Prodigal:002006 CDS 1020946 1021959 . + 0 ID=M1404_00940;Parent=M1404_00940_gene;eC_number=5.4.3.-;Name=epmB;db_xref=COG:COG1509;gene=epmB;inference=ab initio prediction:Prodigal:002006,similar to AA sequence:UniProtKB:P39280;locus_tag=M1404_00940;product=L-lysine 2%2C3-aminomutase;protein_id=gnl|Prokka|M1404_00940

gnl|Prokka|M1404_1 prokka gene 1022053 1023219 . + . ID=M1404_00941_gene;Name=oapA;gene=oapA;locus_tag=M1404_00941

gnl|Prokka|M1404_1 Prodigal:002006 CDS 1022053 1023219 . + 0 ID=M1404_00941;Parent=M1404_00941_gene;Name=oapA;db_xref=COG:COG3061;gene=oapA;inference=ab initio prediction:Prodigal:002006,similar to AA sequence:UniProtKB:P44415;locus_tag=M1404_00941;product=Opacity-associated protein OapA;protein_id=gnl|Prokka|M1404_00941

gnl|Prokka|M1404_1 prokka gene 1023279 1024022 . - . ID=M1404_00942_gene;locus_tag=M1404_00942

gnl|Prokka|M1404_1 Prodigal:002006 CDS 1023279 1024022 . - 0 ID=M1404_00942;Parent=M1404_00942_gene;eC_number=3.5.2.20;db_xref=COG:COG1878;inference=ab initio prediction:Prodigal:002006,similar to AA sequence:UniProtKB:A0NLY7;locus_tag=M1404_00942;product=Isatin hydrolase;protein_id=gnl|Prokka|M1404_00942

gnl|Prokka|M1404_1 prokka gene 1024175 1025083 . - . ID=M1404_00943_gene;Name=rdgC;gene=rdgC;locus_tag=M1404_00943

gnl|Prokka|M1404_1 Prodigal:002006 CDS 1024175 1025083 . - 0 ID=M1404_00943;Parent=M1404_00943_gene;Name=rdgC;db_xref=COG:COG2974;gene=rdgC;inference=ab initio prediction:Prodigal:002006,similar to AA sequence:UniProtKB:P44628;locus_tag=M1404_00943;product=Recombination-associated protein RdgC;protein_id=gnl|Prokka|M1404_00943

gnl|Prokka|M1404_1 prokka gene 1025177 1026004 . + . ID=M1404_00944_gene;Name=proC;gene=proC;locus_tag=M1404_00944

gnl|Prokka|M1404_1 Prodigal:002006 CDS 1025177 1026004 . + 0 ID=M1404_00944;Parent=M1404_00944_gene;eC_number=1.5.1.2;Name=proC;db_xref=COG:COG0345;gene=proC;inference=ab initio prediction:Prodigal:002006,similar to AA sequence:UniProtKB:P22008;locus_tag=M1404_00944;product=Pyrroline-5-carboxylate reductase;protein_id=gnl|Prokka|M1404_00944

gnl|Prokka|M1404_1 prokka gene 1026004 1027161 . + . ID=M1404_00945_gene;Name=hcaT;gene=hcaT;locus_tag=M1404_00945

gnl|Prokka|M1404_1 Prodigal:002006 CDS 1026004 1027161 . + 0 ID=M1404_00945;Parent=M1404_00945_gene;Name=hcaT;gene=hcaT;inference=ab initio prediction:Prodigal:002006,similar to AA sequence:UniProtKB:Q47142;locus_tag=M1404_00945;product=putative 3-phenylpropionic acid transporter;protein_id=gnl|Prokka|M1404_00945

gnl|Prokka|M1404_1 prokka gene 1027164 1028057 . + . ID=M1404_00946_gene;Name=xerD;gene=xerD;locus_tag=M1404_00946

gnl|Prokka|M1404_1 Prodigal:002006 CDS 1027164 1028057 . + 0 ID=M1404_00946;Parent=M1404_00946_gene;Name=xerD;db_xref=COG:COG4974;gene=xerD;inference=ab initio prediction:Prodigal:002006,similar to AA sequence:UniProtKB:P0A8P8;locus_tag=M1404_00946;product=Tyrosine recombinase XerD;protein_id=gnl|Prokka|M1404_00946

gnl|Prokka|M1404_1 prokka gene 1028115 1028432 . - . ID=M1404_00947_gene;Name=yfiA;gene=yfiA;locus_tag=M1404_00947

gnl|Prokka|M1404_1 Prodigal:002006 CDS 1028115 1028432 . - 0 ID=M1404_00947;Parent=M1404_00947_gene;Name=yfiA;db_xref=COG:COG1544;gene=yfiA;inference=ab initio prediction:Prodigal:002006,similar to AA sequence:UniProtKB:P71346;locus_tag=M1404_00947;product=Ribosome-associated factor Y;protein_id=gnl|Prokka|M1404_00947

gnl|Prokka|M1404_1 prokka gene 1028677 1030050 . - . ID=M1404_00948_gene;Name=alsT_2;gene=alsT_2;locus_tag=M1404_00948

gnl|Prokka|M1404_1 Prodigal:002006 CDS 1028677 1030050 . - 0 ID=M1404_00948;Parent=M1404_00948_gene;Name=alsT_2;db_xref=COG:COG1115;gene=alsT_2;inference=ab initio prediction:Prodigal:002006,similar to AA sequence:UniProtKB:Q45068;locus_tag=M1404_00948;product=Amino-acid carrier protein AlsT;protein_id=gnl|Prokka|M1404_00948

gnl|Prokka|M1404_1 prokka gene 1030499 1030768 . - . ID=M1404_00949_gene;locus_tag=M1404_00949

gnl|Prokka|M1404_1 Prodigal:002006 CDS 1030499 1030768 . - 0 ID=M1404_00949;Parent=M1404_00949_gene;inference=ab initio prediction:Prodigal:002006;locus_tag=M1404_00949;product=hypothetical protein;protein_id=gnl|Prokka|M1404_00949

gnl|Prokka|M1404_1 prokka gene 1030768 1030971 . - . ID=M1404_00950_gene;Name=yacG;gene=yacG;locus_tag=M1404_00950

gnl|Prokka|M1404_1 Prodigal:002006 CDS 1030768 1030971 . - 0 ID=M1404_00950;Parent=M1404_00950_gene;Name=yacG;db_xref=COG:COG3024;gene=yacG;inference=ab initio prediction:Prodigal:002006,similar to AA sequence:UniProtKB:P0A8H8;locus_tag=M1404_00950;product=DNA gyrase inhibitor YacG;protein_id=gnl|Prokka|M1404_00950

gnl|Prokka|M1404_1 prokka gene 1030982 1031602 . - . ID=M1404_00951_gene;Name=coaE;gene=coaE;locus_tag=M1404_00951

gnl|Prokka|M1404_1 Prodigal:002006 CDS 1030982 1031602 . - 0 ID=M1404_00951;Parent=M1404_00951_gene;eC_number=2.7.1.24;Name=coaE;db_xref=COG:COG0237;gene=coaE;inference=ab initio prediction:Prodigal:002006,similar to AA sequence:UniProtKB:P44920;locus_tag=M1404_00951;product=Dephospho-CoA kinase;protein_id=gnl|Prokka|M1404_00951

gnl|Prokka|M1404_1 prokka gene 1031617 1032339 . - . ID=M1404_00952_gene;locus_tag=M1404_00952

gnl|Prokka|M1404_1 Prodigal:002006 CDS 1031617 1032339 . - 0 ID=M1404_00952;Parent=M1404_00952_gene;inference=ab initio prediction:Prodigal:002006;locus_tag=M1404_00952;product=hypothetical protein;protein_id=gnl|Prokka|M1404_00952

gnl|Prokka|M1404_1 prokka gene 1032339 1033550 . - . ID=M1404_00953_gene;Name=epsF;gene=epsF;locus_tag=M1404_00953

gnl|Prokka|M1404_1 Prodigal:002006 CDS 1032339 1033550 . - 0 ID=M1404_00953;Parent=M1404_00953_gene;Name=epsF;db_xref=COG:COG1459;gene=epsF;inference=ab initio prediction:Prodigal:002006,similar to AA sequence:UniProtKB:P45780;locus_tag=M1404_00953;product=Type II secretion system protein F;protein_id=gnl|Prokka|M1404_00953

gnl|Prokka|M1404_1 prokka gene 1033550 1034932 . - . ID=M1404_00954_gene;locus_tag=M1404_00954

gnl|Prokka|M1404_1 Prodigal:002006 CDS 1033550 1034932 . - 0 ID=M1404_00954;Parent=M1404_00954_gene;inference=ab initio prediction:Prodigal:002006;locus_tag=M1404_00954;product=hypothetical protein;protein_id=gnl|Prokka|M1404_00954

gnl|Prokka|M1404_1 prokka gene 1034932 1035366 . - . ID=M1404_00955_gene;locus_tag=M1404_00955

gnl|Prokka|M1404_1 Prodigal:002006 CDS 1034932 1035366 . - 0 ID=M1404_00955;Parent=M1404_00955_gene;inference=ab initio prediction:Prodigal:002006;locus_tag=M1404_00955;product=hypothetical protein;protein_id=gnl|Prokka|M1404_00955

gnl|Prokka|M1404_1 prokka gene 1035526 1036092 . + . ID=M1404_00956_gene;Name=ampD;gene=ampD;locus_tag=M1404_00956

gnl|Prokka|M1404_1 Prodigal:002006 CDS 1035526 1036092 . + 0 ID=M1404_00956;Parent=M1404_00956_gene;eC_number=3.5.1.28;Name=ampD;db_xref=COG:COG3023;gene=ampD;inference=ab initio prediction:Prodigal:002006,similar to AA sequence:UniProtKB:P13016;locus_tag=M1404_00956;product=1%2C6-anhydro-N-acetylmuramyl-L-alanine amidase AmpD;protein_id=gnl|Prokka|M1404_00956

gnl|Prokka|M1404_1 prokka gene 1036678 1037274 . + . ID=M1404_00957_gene;Name=rppH;gene=rppH;locus_tag=M1404_00957

gnl|Prokka|M1404_1 Prodigal:002006 CDS 1036678 1037274 . + 0 ID=M1404_00957;Parent=M1404_00957_gene;eC_number=3.6.1.-;Name=rppH;gene=rppH;inference=ab initio prediction:Prodigal:002006,protein motif:HAMAP:MF_00298;locus_tag=M1404_00957;product=RNA pyrophosphohydrolase;protein_id=gnl|Prokka|M1404_00957

gnl|Prokka|M1404_1 prokka gene 1037277 1038071 . + . ID=M1404_00958_gene;locus_tag=M1404_00958

gnl|Prokka|M1404_1 Prodigal:002006 CDS 1037277 1038071 . + 0 ID=M1404_00958;Parent=M1404_00958_gene;inference=ab initio prediction:Prodigal:002006;locus_tag=M1404_00958;product=hypothetical protein;protein_id=gnl|Prokka|M1404_00958

gnl|Prokka|M1404_1 prokka gene 1038081 1038884 . + . ID=M1404_00959_gene;Name=lgt;gene=lgt;locus_tag=M1404_00959

gnl|Prokka|M1404_1 Prodigal:002006 CDS 1038081 1038884 . + 0 ID=M1404_00959;Parent=M1404_00959_gene;eC_number=2.5.1.145;Name=lgt;db_xref=COG:COG0682;gene=lgt;inference=ab initio prediction:Prodigal:002006,similar to AA sequence:UniProtKB:P60955;locus_tag=M1404_00959;product=Phosphatidylglycerol--prolipoprotein diacylglyceryl transferase;protein_id=gnl|Prokka|M1404_00959

gnl|Prokka|M1404_1 prokka gene 1038890 1039741 . + . ID=M1404_00960_gene;Name=thyA;gene=thyA;locus_tag=M1404_00960

gnl|Prokka|M1404_1 Prodigal:002006 CDS 1038890 1039741 . + 0 ID=M1404_00960;Parent=M1404_00960_gene;eC_number=2.1.1.45;Name=thyA;db_xref=COG:COG0207;gene=thyA;inference=ab initio prediction:Prodigal:002006,similar to AA sequence:UniProtKB:P0A884;locus_tag=M1404_00960;product=Thymidylate synthase;protein_id=gnl|Prokka|M1404_00960

gnl|Prokka|M1404_1 prokka gene 1039756 1040256 . + . ID=M1404_00961_gene;Name=tadA;gene=tadA;locus_tag=M1404_00961

gnl|Prokka|M1404_1 Prodigal:002006 CDS 1039756 1040256 . + 0 ID=M1404_00961;Parent=M1404_00961_gene;eC_number=3.5.4.33;Name=tadA;db_xref=COG:COG0590;gene=tadA;inference=ab initio prediction:Prodigal:002006,similar to AA sequence:UniProtKB:P68398;locus_tag=M1404_00961;product=tRNA-specific adenosine deaminase;protein_id=gnl|Prokka|M1404_00961

gnl|Prokka|M1404_1 prokka gene 1040473 1041213 . - . ID=M1404_00962_gene;Name=pflA;gene=pflA;locus_tag=M1404_00962

gnl|Prokka|M1404_1 Prodigal:002006 CDS 1040473 1041213 . - 0 ID=M1404_00962;Parent=M1404_00962_gene;eC_number=1.97.1.4;Name=pflA;db_xref=COG:COG1180;gene=pflA;inference=ab initio prediction:Prodigal:002006,similar to AA sequence:UniProtKB:P0A9N4;locus_tag=M1404_00962;product=Pyruvate formate-lyase 1-activating enzyme;protein_id=gnl|Prokka|M1404_00962

gnl|Prokka|M1404_1 prokka gene 1041376 1043415 . - . ID=M1404_00963_gene;Name=lip-1;gene=lip-1;locus_tag=M1404_00963

gnl|Prokka|M1404_1 Prodigal:002006 CDS 1041376 1043415 . - 0 ID=M1404_00963;Parent=M1404_00963_gene;eC_number=3.1.1.3;Name=lip-1;gene=lip-1;inference=ab initio prediction:Prodigal:002006,similar to AA sequence:UniProtKB:P40601;locus_tag=M1404_00963;product=Lipase 1;protein_id=gnl|Prokka|M1404_00963

gnl|Prokka|M1404_1 prokka gene 1043588 1045912 . - . ID=M1404_00964_gene;Name=pflB;gene=pflB;locus_tag=M1404_00964

gnl|Prokka|M1404_1 Prodigal:002006 CDS 1043588 1045912 . - 0 ID=M1404_00964;Parent=M1404_00964_gene;eC_number=2.3.1.54;Name=pflB;db_xref=COG:COG1882;gene=pflB;inference=ab initio prediction:Prodigal:002006,similar to AA sequence:UniProtKB:P09373;locus_tag=M1404_00964;product=Formate acetyltransferase 1;protein_id=gnl|Prokka|M1404_00964

gnl|Prokka|M1404_1 prokka gene 1045998 1046849 . - . ID=M1404_00965_gene;Name=focA;gene=focA;locus_tag=M1404_00965

gnl|Prokka|M1404_1 Prodigal:002006 CDS 1045998 1046849 . - 0 ID=M1404_00965;Parent=M1404_00965_gene;Name=focA;db_xref=COG:COG2116;gene=focA;inference=ab initio prediction:Prodigal:002006,similar to AA sequence:UniProtKB:P0AC23;locus_tag=M1404_00965;product=putative formate transporter 1;protein_id=gnl|Prokka|M1404_00965

gnl|Prokka|M1404_1 prokka gene 1047237 1047587 . + . ID=M1404_00966_gene;locus_tag=M1404_00966

gnl|Prokka|M1404_1 Prodigal:002006 CDS 1047237 1047587 . + 0 ID=M1404_00966;Parent=M1404_00966_gene;eC_number=3.9.1.-;db_xref=COG:COG0537;inference=ab initio prediction:Prodigal:002006,similar to AA sequence:UniProtKB:P44956;locus_tag=M1404_00966;product=Purine nucleoside phosphoramidase;protein_id=gnl|Prokka|M1404_00966

gnl|Prokka|M1404_1 prokka gene 1047587 1047937 . + . ID=M1404_00967_gene;locus_tag=M1404_00967

gnl|Prokka|M1404_1 Prodigal:002006 CDS 1047587 1047937 . + 0 ID=M1404_00967;Parent=M1404_00967_gene;inference=ab initio prediction:Prodigal:002006;locus_tag=M1404_00967;product=hypothetical protein;protein_id=gnl|Prokka|M1404_00967

gnl|Prokka|M1404_1 prokka gene 1047946 1049004 . + . ID=M1404_00968_gene;Name=nagZ;gene=nagZ;locus_tag=M1404_00968

gnl|Prokka|M1404_1 Prodigal:002006 CDS 1047946 1049004 . + 0 ID=M1404_00968;Parent=M1404_00968_gene;eC_number=3.2.1.52;Name=nagZ;db_xref=COG:COG1472;gene=nagZ;inference=ab initio prediction:Prodigal:002006,similar to AA sequence:UniProtKB:P75949;locus_tag=M1404_00968;product=Beta-hexosaminidase;protein_id=gnl|Prokka|M1404_00968

gnl|Prokka|M1404_1 prokka gene 1049007 1050182 . + . ID=M1404_00969_gene;Name=rlmC;gene=rlmC;locus_tag=M1404_00969

gnl|Prokka|M1404_1 Prodigal:002006 CDS 1049007 1050182 . + 0 ID=M1404_00969;Parent=M1404_00969_gene;eC_number=2.1.1.189;Name=rlmC;db_xref=COG:COG2265;gene=rlmC;inference=ab initio prediction:Prodigal:002006,similar to AA sequence:UniProtKB:P75817;locus_tag=M1404_00969;product=23S rRNA (uracil(747)-C(5))-methyltransferase RlmC;protein_id=gnl|Prokka|M1404_00969

gnl|Prokka|M1404_1 prokka gene 1050275 1051240 . - . ID=M1404_00970_gene;Name=pfkA;gene=pfkA;locus_tag=M1404_00970

gnl|Prokka|M1404_1 Prodigal:002006 CDS 1050275 1051240 . - 0 ID=M1404_00970;Parent=M1404_00970_gene;eC_number=2.7.1.11;Name=pfkA;db_xref=COG:COG0205;gene=pfkA;inference=ab initio prediction:Prodigal:002006,similar to AA sequence:UniProtKB:P0A796;locus_tag=M1404_00970;product=ATP-dependent 6-phosphofructokinase isozyme 1;protein_id=gnl|Prokka|M1404_00970

gnl|Prokka|M1404_1 prokka gene 1051335 1052249 . - . ID=M1404_00971_gene;Name=fieF;gene=fieF;locus_tag=M1404_00971

gnl|Prokka|M1404_1 Prodigal:002006 CDS 1051335 1052249 . - 0 ID=M1404_00971;Parent=M1404_00971_gene;Name=fieF;db_xref=COG:COG0053;gene=fieF;inference=ab initio prediction:Prodigal:002006,similar to AA sequence:UniProtKB:P69380;locus_tag=M1404_00971;product=Ferrous-iron efflux pump FieF;protein_id=gnl|Prokka|M1404_00971

gnl|Prokka|M1404_1 prokka gene 1052250 1052816 . - . ID=M1404_00972_gene;locus_tag=M1404_00972

gnl|Prokka|M1404_1 Prodigal:002006 CDS 1052250 1052816 . - 0 ID=M1404_00972;Parent=M1404_00972_gene;inference=ab initio prediction:Prodigal:002006;locus_tag=M1404_00972;product=hypothetical protein;protein_id=gnl|Prokka|M1404_00972

gnl|Prokka|M1404_1 prokka gene 1052818 1053594 . - . ID=M1404_00973_gene;Name=yaaA;gene=yaaA;locus_tag=M1404_00973

gnl|Prokka|M1404_1 Prodigal:002006 CDS 1052818 1053594 . - 0 ID=M1404_00973;Parent=M1404_00973_gene;Name=yaaA;db_xref=COG:COG3022;gene=yaaA;inference=ab initio prediction:Prodigal:002006,similar to AA sequence:UniProtKB:P0A8I3;locus_tag=M1404_00973;product=Peroxide stress resistance protein YaaA;protein_id=gnl|Prokka|M1404_00973

gnl|Prokka|M1404_1 prokka gene 1053622 1054290 . - . ID=M1404_00974_gene;Name=ung;gene=ung;locus_tag=M1404_00974

gnl|Prokka|M1404_1 Prodigal:002006 CDS 1053622 1054290 . - 0 ID=M1404_00974;Parent=M1404_00974_gene;eC_number=3.2.2.27;Name=ung;db_xref=COG:COG0692;gene=ung;inference=ab initio prediction:Prodigal:002006,similar to AA sequence:UniProtKB:Q9KPK8;locus_tag=M1404_00974;product=Uracil-DNA glycosylase;protein_id=gnl|Prokka|M1404_00974

gnl|Prokka|M1404_1 prokka gene 1054566 1054949 . + . ID=M1404_00975_gene;Name=grcA;gene=grcA;locus_tag=M1404_00975

gnl|Prokka|M1404_1 Prodigal:002006 CDS 1054566 1054949 . + 0 ID=M1404_00975;Parent=M1404_00975_gene;Name=grcA;db_xref=COG:COG3445;gene=grcA;inference=ab initio prediction:Prodigal:002006,similar to AA sequence:UniProtKB:P44455;locus_tag=M1404_00975;product=Autonomous glycyl radical cofactor;protein_id=gnl|Prokka|M1404_00975

gnl|Prokka|M1404_1 prokka gene 1055181 1056977 . + . ID=M1404_00976_gene;Name=lepA;gene=lepA;locus_tag=M1404_00976

gnl|Prokka|M1404_1 Prodigal:002006 CDS 1055181 1056977 . + 0 ID=M1404_00976;Parent=M1404_00976_gene;eC_number=3.6.5.-;Name=lepA;db_xref=COG:COG0481;gene=lepA;inference=ab initio prediction:Prodigal:002006,similar to AA sequence:UniProtKB:P60785;locus_tag=M1404_00976;product=Elongation factor 4;protein_id=gnl|Prokka|M1404_00976

gnl|Prokka|M1404_1 prokka gene 1056996 1058018 . + . ID=M1404_00977_gene;Name=lepB;gene=lepB;locus_tag=M1404_00977

gnl|Prokka|M1404_1 Prodigal:002006 CDS 1056996 1058018 . + 0 ID=M1404_00977;Parent=M1404_00977_gene;eC_number=3.4.21.89;Name=lepB;db_xref=COG:COG0681;gene=lepB;inference=ab initio prediction:Prodigal:002006,similar to AA sequence:UniProtKB:P00803;locus_tag=M1404_00977;product=Signal peptidase I;protein_id=gnl|Prokka|M1404_00977

gnl|Prokka|M1404_1 prokka gene 1058025 1058702 . + . ID=M1404_00978_gene;Name=rnc;gene=rnc;locus_tag=M1404_00978

gnl|Prokka|M1404_1 Prodigal:002006 CDS 1058025 1058702 . + 0 ID=M1404_00978;Parent=M1404_00978_gene;eC_number=3.1.26.3;Name=rnc;db_xref=COG:COG0571;gene=rnc;inference=ab initio prediction:Prodigal:002006,similar to AA sequence:UniProtKB:P0A7Y0;locus_tag=M1404_00978;product=Ribonuclease 3;protein_id=gnl|Prokka|M1404_00978

gnl|Prokka|M1404_1 prokka gene 1058699 1059619 . + . ID=M1404_00979_gene;Name=era;gene=era;locus_tag=M1404_00979

gnl|Prokka|M1404_1 Prodigal:002006 CDS 1058699 1059619 . + 0 ID=M1404_00979;Parent=M1404_00979_gene;Name=era;db_xref=COG:COG1159;gene=era;inference=ab initio prediction:Prodigal:002006,similar to AA sequence:UniProtKB:P06616;locus_tag=M1404_00979;product=GTPase Era;protein_id=gnl|Prokka|M1404_00979

gnl|Prokka|M1404_1 prokka gene 1060077 1073309 . - . ID=M1404_00980_gene;locus_tag=M1404_00980

gnl|Prokka|M1404_1 Prodigal:002006 CDS 1060077 1073309 . - 0 ID=M1404_00980;Parent=M1404_00980_gene;inference=ab initio prediction:Prodigal:002006;locus_tag=M1404_00980;product=hypothetical protein;protein_id=gnl|Prokka|M1404_00980

gnl|Prokka|M1404_1 prokka gene 1073339 1075057 . - . ID=M1404_00981_gene;Name=fhaC;gene=fhaC;locus_tag=M1404_00981
[truncated: 3,144,738 more chars]
