# Supplementary material for: Comparative analysis of shared and unique mechanisms important for diverse strains of Pasteurella multocida to cause systemic infection in mice
Source: PLoS Pathog. 2025 Dec 22;21(12):e1013398. doi: 10.1371/journal.ppat.1013398 (PMC12721544; doi:10.1371/journal.ppat.1013398)
Supplement: S1 Table — (DOCX) [file ppat.1013398.s008.docx]

**S1 Table**. Infectious dose and number of mice used for *P. multocida* systemic infections performed in this study

| Strain | Strain number | Mice used for experiment | Infectious dose in CFU^1^ |
| --- | --- | --- | --- |
| VP161 *Himar1* mutant library | NA^2^ | Two males and two females | 1.44 x 10^7^ |
| M1404 *Himar1* mutant library | NA | Two males and two females | 1.95 x 10^7^ |
| Wild-type M1404 harbouring empty vector | AL4221 | Three females | 3.30 x 10^4^ |
| Wild-type M1404 harbouring empty vector | AL4221 | Three males | 2.50 x 10^4^ |
| M1404 *alsT_1* mutant harbouring empty vector | AL4853 | Three females | 1.75 x 10^5^ |
| M1404 *alsT_1* mutant harbouring empty vector | AL4853 | Three males | 1.15 x 10^5^ |
| M1404 *crp* mutant harbouring empty vector | AL4855 | Three females | 1.70 x 10^5^ |
| M1404 *crp* mutant harbouring empty vector | AL4855 | Three males | 1.48 x 10^5^ |
| M1404 *cyaA* mutant harbouring empty vector | AL4857 | Three females | 1.87 x 10^5^ |
| M1404 *cyaA* mutant harbouring empty vector | AL4857 | Three males | 1.40 x 10^5^ |

^1^ CFU – Colony forming units

^2^ NA – Not applicable
