# Supplementary material for: Comparative analysis of shared and unique mechanisms important for diverse strains of Pasteurella multocida to cause systemic infection in mice
Source: PLoS Pathog. 2025 Dec 22;21(12):e1013398. doi: 10.1371/journal.ppat.1013398 (PMC12721544; doi:10.1371/journal.ppat.1013398)
Supplement: S2 Table — (DOCX) [file ppat.1013398.s009.docx]

**S2 Table.** TraDIS mapping statistics for the input transposon mutant libraries

| Library | Transposon-DNA junction reads | Mapped reads | Number of unique *Himar1* insertion sites (UIS^1^) | Resolution (Distance between UIS) |
| --- | --- | --- | --- | --- |
| Input libraries |  |  |  |  |
| VP161 |  |  |  |  |
| Library 1 | 1,757,473 | 1,211,338 | 42,771 | 52.51 |
| Library 2 | 1,764,365 | 1,240,274 | 42,803 | 52.47 |
| Library 3 | 1,757,577 | 1,222,848 | 42,518 | 52.83 |
| Library 4 | 1,764,661 | 1,218,068 | 42,060 | 53.40 |
| Total |  |  | 71,190 | 31.55 |
| M1404 |  |  |  |  |
| Library 1 | 6,981,977 | 5,807,837 | 38,852 | 62.30 |
| Library 2 | 6,648,491 | 5,590,017 | 37,424 | 64.68 |
| Library 3 | 7,569,978 | 6,337,831 | 39,834 | 60.77 |
| Library 4 | 6,784,647 | 5,698,351 | 38,252 | 63.28 |
| Total |  |  | 60,416 | 40.07 |
| Mice infected with VP161 |  |  |  |  |
| Blood |  |  |  |  |
| Mouse 1 | 1,770,340 | 1,313,856 | 40,159 | 55.93 |
| Mouse 2 | 1,734,955 | 1,254,692 | 40,924 | 54.88 |
| Mouse 3 | 1,775,359 | 1,298,576 | 38,852 | 57.81 |
| Mouse 4 | 1,767,708 | 1,302,992 | 39,768 | 56.48 |
| Total |  |  | 69,813 | 32.17 |
| Liver |  |  |  |  |
| Mouse 1 | 1,770,960 | 1,317,312 | 39,695 | 56.58 |
| Mouse 2 | 1,782,436 | 1,326,244 | 40,916 | 54.89 |
| Mouse 3 | 1,776,693 | 1,330,889 | 39,400 | 57.01 |
| Mouse 4 | 1,773,297 | 1,300,166 | 40,860 | 54.97 |
| Total |  |  | 71,057 | 31.61 |
| Spleen |  |  |  |  |
| Mouse 1 | 1,732,783 | 1,327,824 | 39,165 | 57.35 |
| Mouse 2 | 1,775,808 | 1,317,514 | 41,124 | 54.62 |
| Mouse 3 | 1,777,345 | 1,299,770 | 40,803 | 55.05 |
| Mouse 4 | 1,779,576 | 1,307,655 | 41,408 | 54.24 |
| Total |  |  | 73,400 | 30.60 |
| Mice infected with M1404 |  |  |  |  |
| Blood |  |  |  |  |
| Mouse 1 | 6,511,884 | 5,524,455 | 31,048 | 77.96 |
| Mouse 2 | 6,106,712 | 5,195,119 | 32,709 | 74.01 |
| Mouse 3 | 7,248,536 | 6,114,855 | 33,175 | 72.97 |
| Mouse 4 | 6,832,751 | 5,786,335 | 32,246 | 75.07 |
| Total |  |  | 56,761 | 42.65 |
| Liver |  |  |  |  |
| Mouse 1 | 7,954,019 | 6,748,110 | 31,770 | 76.19 |
| Mouse 2 | 5,330,169 | 4,430,316 | 30,228 | 80.08 |
| Mouse 3 | 9,066,531 | 7,847,303 | 36,144 | 66.97 |
| Mouse 4 | 6,347,637 | 5,32,2350 | 32,737 | 73.94 |
| Total |  |  | 56,948 | 42.51 |
| Spleen |  |  |  |  |
| Mouse 1 | 7,059,436 | 6,025,578 | 30,034 | 80.60 |
| Mouse 2 | 6,674,658 | 5,671,969 | 32,796 | 73.81 |
| Mouse 3 | 7,936,658 | 6,713,440 | 34,763 | 69.63 |
| Mouse 4 | 7,998,355 | 6,788,619 | 33,347 | 72.59 |
| Total |  |  | 57,371 | 42.19 |

^1^UIS – unique *Himar1* insertion sites
