# Supplementary material for: Comparative analysis of shared and unique mechanisms important for diverse strains of Pasteurella multocida to cause systemic infection in mice
Source: PLoS Pathog. 2025 Dec 22;21(12):e1013398. doi: 10.1371/journal.ppat.1013398 (PMC12721544; doi:10.1371/journal.ppat.1013398)
Supplement: S3 Table — (DOCX) [file ppat.1013398.s010.docx]

**S3 Table.** Genes in the 100% *P. multocida* core genome (1,564 genes) that encode proteins with matches to the curated database of *P. multocida* virulence factor and antibiotic resistance genes [1] at >75% amino acid identity.

| General function | Genes |
| --- | --- |
| Iron receptors | *hbpA*^1^, *hgbA*, PM0741, PM1081, PM1428 |
| Iron transporters | *tonB-exbBD*, *afuABC*, *afuA_2*, *afuA_3*, *fecCDE*, *fbpB*, *fbpC*, *yfeABCD* |
| Outer membrane proteins and fimbriae | *comE1*, *oma87*^1^, *ompH_2*, *ptfA* |
| Sialic acid uptake and utilisation | *nanP*, *nanU*, *nanB* |
| Methionine uptake | *metQ* |
| Transcriptional regulators, two component systems, and the stringent response | *spoT*^1^, *fis*, *fur*^1^, *hfq*, *qseB*, *qseC*, PM0442^1^ |
| Quorum sensing | *lsrABCDFGKR-luxS* |
| LPS biosynthesis | *hptA*, *hptB*, *hptC*, *hptD*, *lpt-3*, *gtcB*, *kdtA*^1^ |

^1^Identified as essential in VP161 and M1404 for growth in rich media

1. Smallman TR, Perlaza-Jiménez L, Wang X, Korman TM, Kotsanas D, Gibson JS, et al. Pathogenomic analysis and characterization of *Pasteurella multocida* strains recovered from human infections. Microbiol Spectr. 2024;12(4):e0380523. doi: 10.1128/spectrum.03805-23.
