# Supplementary material for: Comparative analysis of shared and unique mechanisms important for diverse strains of Pasteurella multocida to cause systemic infection in mice
Source: PLoS Pathog. 2025 Dec 22;21(12):e1013398. doi: 10.1371/journal.ppat.1013398 (PMC12721544; doi:10.1371/journal.ppat.1013398)
Supplement: S4 Table — (DOCX) [file ppat.1013398.s011.docx]

**S4 Table.** Genes that result in decreased *in vivo* fitness when disrupted in *P. multocida* strain VP161 or M1404, as identified by TraDIS analysis of mutants recovered from either the blood, liver or spleen following systemic infections in BALB/c mice.

|  |  |  |  |  | VP161 | | | | M1404 | | | |
| --- | --- | --- | --- | --- | --- | --- | --- | --- | --- | --- | --- | --- |
| VP161 locus tag^1^ | VP161 gene | M1404 locus tag^2^ | M1404 gene | Function | Rich media | Blood | Liver | Spleen | Rich media | Blood | Liver | Spleen |
| 0005 | *crp* | 00005 | *crp* | cAMP-activated global transcriptional regulator | Yes | NA^3^ | NA | NA | No | Yes | Yes | Yes |
| NA | *NA* | 00018 | *lex1* | Lipooligosaccharide biosynthesis protein | NA | NA | NA | NA | No | Yes | Yes | Yes |
| 0075 | *dus* | 00072 | *dusB* | tRNA-dihydrouridine synthase B | No | Yes | Yes | Yes | No | No | No | No |
| 0076 | *fis* | 00073 | *fis* | DNA-binding protein | No | Yes | Yes | Yes | No | NA | NA | NA |
| 0077 | *purL* | 00074 | *purL* | Phosphoribosylformylglycinamidine synthase | No | Yes | Yes | Yes | No | Yes | Yes | Yes |
| 0089 | *pgm* | 00086 | *pgm* | Phosphoglucomutase, capsule monomer biosynthesis | No | Yes | Yes | Yes | No | Yes | Yes | Yes |
| 0094 | 0094 | 00091 | 00091 | 47 kDa outer membrane protein | No | Yes | Yes | Yes | No | No | No | No |
| 0183 | 0183 | 00293 | 00293 | hypothetical protein | No | Yes | Yes | Yes | No | No | No | No |
| 0184 | *rnt* | 00294 | *rnt* | Ribonuclease T | Yes | No | No | No | No | Yes | No | No |
| 0191 | *nudB* | 00301 | *nudB* | Dihydroneopterin triphosphate diphosphatase | No | No | No | No | No | No | No | Yes |
| 0207 | *pal* | 00317 | *pal* | Peptidoglycan-associated lipoprotein | No | No | Yes | No | Yes | NA | NA | NA |
| 0219 | *holD* | 00327 | *holD* | DNA polymerase III subunit psi | No | No | No | No | No | Yes | No | No |
| 0242 | *purA* | 00350 | *purA* | Adenylosuccinate synthetase | No | Yes | Yes | Yes | No | Yes | Yes | Yes |
| 0252 | 0252 | 00360 | 00360 | tRNA-Met | Yes | NA | NA | NA | No | No | Yes | No |
| 0261 | *rpoZ* | 00369 | *rpoZ* | DNA-directed RNA polymerase subunit omega | No | No | No | No | No | No | No | Yes |
| 0281 | *hutZ_1* | 01072 | *hutZ_1* | Heme oxygenase | No | Yes | Yes | Yes | No | No | No | No |
| 0490 | 0490 | 00853 | 00853 | hypothetical protein | No | No | No | No | No | Yes | No | Yes |
| 0496 | 0496 | 00849 | 00849 | hypothetical protein | No | No | No | No | No | Yes | Yes | Yes |
| 0514 | *kdsC* | 00831 | *kdsC* | 3-deoxy-D-manno-octulosonate 8-phosphate phosphatase | Yes | NA | NA | NA | No | Yes | Yes | No |
| 0519 | *alsT_1* | 00826 | *alsT_1* | Amino-acid carrier protein | No | Yes | Yes | Yes | No | Yes | Yes | Yes |
| 0543 | *mdh* | 00805 | *mdh* | Malate dehydrogenase | No | No | No | No | No | Yes | No | No |
| 0549 | *prmC* | 00799 | *prmC* | Release factor glutamine methyltransferase | No | Yes | Yes | Yes | No | Yes | Yes | Yes |
| 0607 | *purE* | 00736 | *purE* | N5-carboxyaminoimidazole ribonucleotide mutase | No | Yes | Yes | Yes | No | Yes | Yes | Yes |
| 0608 | *purK* | 00735 | *purK* | N5-carboxyaminoimidazole ribonucleotide synthase | No | Yes | Yes | No | No | Yes | Yes | Yes |
| 0609 | *aspC* | 00734 | *aspC* | Aspartate aminotransferase | No | Yes | No | Yes | No | Yes | Yes | Yes |
| 0693 | *ubiX* | 00652 | *ubiX* | Flavin prenyltransferase | No | Yes | Yes | Yes | No | NA | NA | NA |
| 0694 | *purF* | 00651 | *purF* | Amidophosphoribosyltransferase | No | Yes | Yes | Yes | No | Yes | Yes | Yes |
| 0695 | *cvpA* | 00650 | *cvpA* | Colicin V production protein | No | Yes | Yes | Yes | No | No | No | Yes |
| 0727 | *tyrP_1* | 00619 | *tyrP_2* | Tyrosine-specific transport protein | No | Yes | Yes | Yes | No | No | No | No |
| 0773 | *phyB* | 00576 | *lipB* | Homology to capsule phospholipid substitution proteins | No | Yes | Yes | Yes | No | Yes | Yes | Yes |
| 0774 | *phyA* | NA | *NA* | Homology to capsule phospholipid substitution proteins | No | Yes | Yes | Yes | NA | NA | NA | NA |
| 0775 | *hyaE* | NA | *NA* | Hyaluronic acid capsule biosynthesis protein | No | Yes | Yes | Yes | NA | NA | NA | NA |
| 0776 | *hyaD* | NA | *NA* | Hyaluronic acid synthase | No | Yes | Yes | Yes | NA | NA | NA | NA |
| 0777 | *hyaC* | NA | *NA* | UDP-glucose 6-dehydrogenase | No | Yes | Yes | Yes | NA | NA | NA | NA |
| 0778 | *hyaB* | NA | *NA* | Hyaluronic acid capsule biosynthesis | No | Yes | Yes | Yes | NA | NA | NA | NA |
| 0779 | *hexD* | 00565 | *cexD* | Capsule export | Yes | Yes | Yes | No | No | Yes | Yes | Yes |
| 0780 | *hexC* | 00564 | *cexC* | Capsule export | Yes | Yes | Yes | Yes | No | Yes | Yes | Yes |
| 0781 | *hexB* | 00563 | *cexB* | Capsule transport protein | Yes | NA | NA | NA | No | Yes | Yes | Yes |
| 0782 | *hexA* | 00562 | *cexA* | Capsule transport ATP-binding protein | No | NA | NA | NA | No | Yes | Yes | Yes |
| NA | *NA* | 00566 | *lipA* | Capsule attachment | NA | NA | NA | NA | No | Yes | Yes | Yes |
| NA | *NA* | 00567 | *bcbI* | Capsule biosynthesis | NA | NA | NA | NA | No | Yes | Yes | Yes |
| NA | *NA* | 00568 | *bcbH* | Capsule biosynthesis | NA | NA | NA | NA | No | Yes | Yes | Yes |
| NA | *NA* | 00569 | *bcbG* | Capsule biosynthesis | NA | NA | NA | NA | No | Yes | Yes | Yes |
| NA | *NA* | 00570 | *bcbF* | Capsule biosynthesis | NA | NA | NA | NA | No | Yes | Yes | Yes |
| NA | *NA* | 00571 | *bcbE* | Capsule biosynthesis | NA | NA | NA | NA | No | Yes | Yes | Yes |
| NA | *NA* | 00572 | *bcbD* | Capsule biosynthesis | NA | NA | NA | NA | No | Yes | Yes | Yes |
| NA | *NA* | 00573 | *bcbC* | Capsule synthase | NA | NA | NA | NA | No | Yes | Yes | Yes |
| NA | *NA* | 00574 | *bcbB* | UDP-N-acetyl-D-mannosamine dehydrogenase | NA | NA | NA | NA | No | Yes | Yes | Yes |
| NA | *NA* | 00575 | *bcbA* | UDP-N-acetylglucosamine 2-epimerase | NA | NA | NA | NA | No | Yes | Yes | Yes |
| 0819 | *purC* | 00529 | *purC* | Phosphoribosylaminoimidazole-succinocarboxamide synthase | No | Yes | Yes | Yes | No | Yes | Yes | Yes |
| 0845 | *aroA* | 00506 | *aroA* | 3-phosphoshikimate 1-carboxyvinyltransferase | No | Yes | Yes | Yes | No | Yes | Yes | Yes |
| 0846 | *ubiG* | 00505 | *ubiG* | Ubiquinone biosynthesis O-methyltransferase | No | Yes | Yes | No | Yes | NA | NA | NA |
| 0872 | *folK* | 00431 | *folK* | 2-amino-4-hydroxy-6- hydroxymethyldihydropteridine pyrophosphokinase | Yes | NA | NA | NA | Yes | Yes | Yes | Yes |
| 0878 | 0878 | 00425 | 00425 | Putative Na/H antiporter | Yes | Yes | Yes | No | Yes | Yes | No | Yes |
| 0891 | *hldE* | 00412 | *hldE* | Bifunctional protein, LPS biosynthesis | No | Yes | Yes | Yes | No | Yes | Yes | Yes |
| 0914 | *hfq* | 00389 | *hfq* | RNA-binding protein | No | Yes | Yes | Yes | No | No | No | No |
| 0919 | *sapA* | 00384 | *sapA* | Peptide transport periplasmic protein | No | No | No | No | No | Yes | Yes | Yes |
| 0920 | *sapB_1* | 00383 | *sapB_1* | Putrescine export system permease protein | No | Yes | No | No | Yes | NA | NA | NA |
| 0921 | *sapC* | 00382 | *sapC* | Peptide transport system permease protein | Yes | No | Yes | No | Yes | NA | NA | NA |
| 0932 | *guaB* | 01083 | *guaB* | Inosine-5'-monophosphate dehydrogenase | Yes | NA | NA | NA | No | Yes | Yes | Yes |
| 0942 | *galE* | 01093 | *galE* | UDP-glucose 4-epimerase | No | No | No | Yes | No | Yes | Yes | Yes |
| 0994 | *trhP* | 01144 | *trhP* | tRNA wobble base hydroxylation protein | No | No | No | No | No | Yes | Yes | No |
| 1003 | *purD* | 01153 | *purD* | Phosphoribosylamine--glycine ligase | No | Yes | Yes | Yes | No | Yes | Yes | Yes |
| 1006 | *purH* | 01156 | *purH* | Bifunctional purine biosynthesis protein | No | Yes | Yes | Yes | No | Yes | Yes | Yes |
| 1029 | *epmA* | 01179 | *epmA* | Elongation factor P--(R)-beta-lysine ligase | No | Yes | Yes | Yes | No | Yes | No | No |
| 1044 | *neuA_1* | 01194 | *neuA_1* | N-acylneuraminate cytidylyltransferase | No | No | No | No | No | Yes | Yes | Yes |
| 1053 | *mlaB* | 01203 | *mlaB* | Phospholipid ABC transporter protein | Yes | No | No | No | No | Yes | No | No |
| 1055 | *mlaD* | 01205 | *mlaD* | Phospholipid ABC transporter-binding protein | No | No | No | No | No | Yes | No | No |
| 1056 | *mlaE* | 01206 | *mlaE* | Phospholipid ABC transporter permease protein | No | No | No | No | No | Yes | No | No |
| 1057 | *mlaF* | 01207 | *mlaF* | Phospholipid import ATP-binding protein | No | No | No | No | No | Yes | Yes | No |
| 1061 | *ptsN* | 01211 | *ptsN* | Nitrogen regulatory protein | No | No | No | No | Yes | No | Yes | No |
| 1068 | *ychF* | 01218 | *ychF* | Ribosome-binding ATPase | No | No | No | No | No | Yes | No | Yes |
| 1109 | *gmhA* | 01259 | *gmhA* | Phosphoheptose isomerase | No | Yes | Yes | Yes | No | Yes | Yes | Yes |
| 1136 | *epmB* | 01285 | *epmB* | L-lysine 2,3-aminomutase | No | Yes | Yes | Yes | No | No | No | No |
| 1172 | *lepA* | 01321 | *lepA* | Elongation factor 4 | Yes | No | No | No | Yes | No | No | Yes |
| 1217 | *purM* | 01364 | *purM* | Phosphoribosylformylglycinamidine cyclo-ligase | No | Yes | Yes | Yes | No | Yes | Yes | Yes |
| 1218 | *purN* | 01365 | *purN* | Phosphoribosylglycinamide formyltransferase | No | Yes | Yes | Yes | No | Yes | Yes | Yes |
| 1273 | *lon* | 01420 | *lon* | Lon protease | Yes | No | No | No | No | Yes | Yes | Yes |
| 1281 | *srlB* | 01428 | *srlB* | PTS system glucitol/sorbitol-specific EIIA component | No | No | No | No | No | Yes | Yes | Yes |
| 1294 | *mreB* | 01441 | *mreB* | Rod shape-determining protein | Yes | No | No | No | No | No | Yes | No |
| 1310 | *mrdA* | 01462 | *mrdA* | Peptidoglycan D,D-transpeptidase | No | No | No | No | Yes | Yes | Yes | No |
| 1321 | *plsX* | 01473 | *plsX* | Phosphate acyltransferase | No | Yes | Yes | Yes | Yes | NA | NA | NA |
| 1346 | *cpxA* | 01498 | *cpxA* | Sensor histidine kinase | No | No | No | No | No | Yes | No | Yes |
| 1359 | *rph* | 01511 | *rph* | Ribonuclease PH | No | No | No | No | No | Yes | No | Yes |
| 1376 | 1376 | 01528 | 01528 | putative ferredoxin-like protein | Yes | NA | NA | NA | No | Yes | No | No |
| 1383 | *purB* | 01535 | *purB* | Adenylosuccinate lyase | No | No | No | No | No | Yes | Yes | Yes |
| 1384 | *hflD* | 01536 | *hflD* | High frequency lysogenization protein | No | No | No | No | No | Yes | Yes | Yes |
| 1390 | *hptC* | 01542 | *hptC* | ADP-heptose--LPS heptosyltransferase-adds Hep II to Hep I in LPS | No | No | No | No | No | Yes | Yes | Yes |
| 1422 | *cyaA* | 01575 | *cyaA* | Adenylate cyclase | No | No | No | No | No | Yes | Yes | No |
| 1431 | *1431* | 01584 | *01584* | hypothetical protein | No | No | No | No | No | Yes | Yes | Yes |
| 1432 | *dsbA* | 01585 | *dsbA* | Thiol:disulfide interchange protein DsbA | No | No | No | No | No | No | No | Yes |
| 1480 | *tufA_1* | 01630 | *tufB_1* | Elongation factor Tu | No | No | No | No | No | Yes | Yes | Yes |
| 1572 | *gpsA* | 01723 | *gpsA* | Glycerol-3-phosphate dehydrogenase (NAD(P)+) | No | Yes | Yes | Yes | Yes | NA | NA | NA |
| 1604 | *pabA* | 01755 | *pabA* | Aminodeoxychorismate/anthranilate synthase component 2 | No | Yes | Yes | Yes | No | No | No | No |
| 1605 | *pabB* | 01756 | *pabB* | Aminodeoxychorismate synthase component 1 | No | Yes | Yes | Yes | No | No | No | No |
| NA | *NA* | 01811 | 01811 | Surface lipoprotein PmSLP-3 | NA | NA | NA | NA | No | Yes | Yes | Yes |
| 1663 | 1663 | 01812 | 01812 | Slam exporter | No | No | No | No | No | Yes | Yes | Yes |
| 1664 | *rsmD* | 01813 | *rsmD* | Ribosomal RNA small subunit methyltransferase D | No | No | No | No | No | Yes | No | Yes |
| 1720 | *asnC* | 01872 | *asnC* | Regulatory protein AsnC | No | Yes | Yes | Yes | No | Yes | Yes | Yes |
| 1721 | *asnA* | 01873 | *asnA* | Aspartate--ammonia ligase | No | Yes | Yes | Yes | No | Yes | Yes | Yes |
| 1798 | *thiE* | 01958 | *thiE* | Thiamine-phosphate synthase | Yes | NA | NA | NA | Yes | Yes | Yes | Yes |
| 1809 | *aroE* | 01969 | *aroE* | Shikimate dehydrogenase (NADP(+)) | No | Yes | Yes | Yes | Yes | NA | NA | NA |
| 1828 | *galU* | 01988 | *galU* | UTP--glucose-1-phosphate uridylyltransferase | No | Yes | Yes | Yes | No | Yes | Yes | Yes |
| 1833 | *hptD* | 01993 | *hptD* | Addition of Hep III to the inner core | No | No | No | No | No | Yes | Yes | Yes |
| 1844 | *hptA* | 02001 | *hptA* | Heptosyltransferase-adds Hep I to glycoform A LPS | No | Yes | Yes | No | No | Yes | Yes | Yes |
| 1848 | *gctB* | 02005 | *gctB* | Galactosyltransferase-adds to Hep I in LPS | No | No | No | No | No | Yes | Yes | Yes |
| 1886 | *hldD* | 02046 | *hldD* | ADP-L-glycero-D-manno-heptose-6-epimerase | No | Yes | No | Yes | No | Yes | Yes | Yes |
| 1902 | *tufA_2* | 02062 | *tufB_2* | Elongation factor Tu | No | No | No | No | No | Yes | Yes | Yes |
| 1919 | *plpB* | 02078 | *plpB* | putative D-methionine-binding lipoprotein | No | Yes | Yes | Yes | No | Yes | Yes | Yes |
| 1920 | *metP* | 02079 | *metP* | Methionine import system permease protein | No | Yes | Yes | Yes | No | Yes | Yes | Yes |
| 1921 | *metN* | 02080 | *metN* | Methionine import ATP-binding protein | No | Yes | Yes | Yes | No | No | Yes | No |
| 1922 | *gmhB* | 02081 | *gmhB* | D-glycero-beta-D-manno-heptose-1,7-bisphosphate 7-phosphatase | No | No | Yes | No | Yes | NA | NA | NA |
| 1931 | *ubiH* | 02090 | *ubiH* | 2-octaprenyl-6-methoxyphenol hydroxylase | No | Yes | No | No | No | No | No | No |
| 1945 | *nanE* | 02104 | *nanE* | Putative N-acetylmannosamine-6-phosphate 2-epimerase | Yes | No | No | No | No | Yes | Yes | No |
| 1947 | *nanP* | 02106 | *nanP* | Sialic acid-binding periplasmic protein | No | No | No | No | No | Yes | Yes | Yes |
| 1948 | *nanU* | 02107 | *nanU* | Sialic acid TRAP transporter permease protein | No | No | No | No | No | Yes | Yes | Yes |
| 1971 | *ubiE_3* | 02130 | *ubiE* | Ubiquinone/menaquinone biosynthesis C-methyltransferase UbiE | No | Yes | No | Yes | No | NA | NA | NA |
| 1986 | *serA* | 02145 | *serA* | D-3-phosphoglycerate dehydrogenase | No | Yes | Yes | Yes | No | No | No | No |
| 2000 | *serB* | 02159 | *serB* | Phosphoserine phosphatase | No | Yes | Yes | Yes | No | No | No | No |
| 2079 | *tonB* | 02253 | *tonB* | TonB-ExbBD system | No | Yes | Yes | No | No | Yes | Yes | Yes |
| 2080 | *exbD* | 02254 | *exbD* | TonB-ExbBD system | No | Yes | Yes | No | No | Yes | Yes | Yes |
| 2081 | *exbB* | 02255 | *exbB* | TonB-ExbBD system | No | Yes | Yes | No | No | Yes | Yes | Yes |

^1^The number represents the VP161 locus tag without the PmVP161_ prefix

^2^The number represents the M1404 locus tag without the M1404_ prefix

^3^NA – Not applicable
