# Supplementary material for: Comparative analysis of shared and unique mechanisms important for diverse strains of Pasteurella multocida to cause systemic infection in mice
Source: PLoS Pathog. 2025 Dec 22;21(12):e1013398. doi: 10.1371/journal.ppat.1013398 (PMC12721544; doi:10.1371/journal.ppat.1013398)
Supplement: S5 Table — (DOCX) [file ppat.1013398.s012.docx]

**S5 Table.** Genes that result in increased *in vivo* fitness when disrupted in *P. multocida* strain VP161 or M1404, as identified by TraDIS analysis of mutants recovered from either the blood, liver or spleen following systemic infections in BALB/c mice.

|  |  |  |  |  | VP161 | | | | M1404 | | | |
| --- | --- | --- | --- | --- | --- | --- | --- | --- | --- | --- | --- | --- |
| VP161 locus tag^1^ | VP161 gene | M1404 locus tag^2^ | M1404 gene | Function | Rich media | Blood | Liver | Spleen | Rich media | Blood | Liver | Spleen |
| 0233 | 0233 | 00341 | 00341 | Hypothetical protein | No | No | No | No | No | Yes | No | No |
| 0256 | 0256 | 00364 | 00364 | Long-chain-fatty-acid--CoA ligase FadD15 | No | No | Yes | Yes | No | No | No | No |
| 0818 | *yeeZ* | 00530 | *yeeZ* | Protein YeeZ | Yes | Yes | Yes | Yes | Yes | Yes | Yes | Yes |
| NA^3^ | NA | 00860 | 00860 | Hypothetical protein | NA | NA | NA | NA | No | No | Yes | No |
| 0959 | *proQ* | 01109 | *proQ* | RNA chaperone | Yes | NA | NA | Yes | Yes | NA | NA | NA |
| 1146 | *yacG* | 01295 | *yacG* | DNA gyrase inhibitor | Yes | NA | NA | NA | Yes | Yes | No | No |
| 1492 | *gpt* | 01642 | *gpt* | Xanthine phosphoribosyltransferase | Yes | Yes | Yes | Yes | Yes | NA | NA | NA |
| 1514 | *speB* | 01672 | *speB* | Agmatinase | Yes | Yes | Yes | Yes | Yes | Yes | Yes | Yes |
| 1565 | 1565 | 01716 | 01716 | Hypothetical protein | No | No | No | No | No | Yes | Yes | Yes |
| 1804 | *ssuB* | 01964 | *ssuB* | Aliphatic sulfonates import ATP-binding protein | Yes | No | Yes | No | No | No | No | No |
| 1863 | *mltC* | 02022 | *mltC* | Membrane-bound lytic murein transglycosylase C | Yes | Yes | Yes | Yes | Yes | NA | NA | NA |
| 2098 | *rraA* | 02274 | *rraA* | Regulator of ribonuclease activity A | No | No | No | No | No | Yes | Yes | Yes |

^1^The number represents the VP161 locus tag without the PmVP161_ prefix

^2^The number represents the M1404 locus tag without the M1404_ prefix

^3^NA – Not applicable
