# Supplementary material for: Comparative analysis of shared and unique mechanisms important for diverse strains of Pasteurella multocida to cause systemic infection in mice
Source: PLoS Pathog. 2025 Dec 22;21(12):e1013398. doi: 10.1371/journal.ppat.1013398 (PMC12721544; doi:10.1371/journal.ppat.1013398)
Supplement: S6 Table — (DOCX) [file ppat.1013398.s013.docx]

**S6 Table.** Strains and plasmids used in this study.

| Strain or plasmid | Description | Source or reference |
| --- | --- | --- |
| Strains |  |  |
| *P. multocida* |  |  |
| M1404 | Bison haemorrhagic septicaemia isolate, serotype B:L2 | K. R. Rhoades, National Animal Disease Center, Ames, Iowa |
| VP161 | Chicken fowl cholera isolate, serotype A:1 | [1] |
| VP161-Tn*7* | VP161 with Tn*7* insertion downstream of *glmS*; Kan^R^ | [2] |
| AL2188 | VP161 harbouring pAL99S; Spec^R^ | [3] |
| AL4221 | M1404 harbouring pAL99S; Spec^R^ | This study |
| AL4853 | M1404 *alsT_1* insertional mutant harbouring pAL99S; Kan^R^ Spec^R^ | This study |
| AL4855 | M1404 *crp*  insertional mutant harbouring pAL99S; Kan^R^ Spec^R^ | This study |
| AL4857 | M1404 *cyaA*  insertional mutant harbouring pAL99S; Kan^R^ Spec^R^ | This study |
| AL4870 | M1404 *alsT_1* insertional mutant harbouring pAL2045; Kan^R^ Spec^R^ | This study |
| AL4871 | M1404 *cyaA*  insertional mutant harbouring pAL2046; Kan^R^ Spec^R^ | This study |
| AL4872 | VP161 *cyaA* insertional mutant harbouring pAL99S; Kan^R^ Spec^R^ | This study |
| AL4873 | VP161 *cyaA* insertional mutant harbouring pAL2047; Kan^R^ Spec^R^ | This study |
| AL4874 | M1404 *crp* insertional mutant harbouring pAL2048; Kan^R^ Spec^R^ | This study |
|  |  |  |
| *E. coli* |  |  |
| DH5α | F^-^ *deoR endA1* *gyrA96 hsdR17* (r_K_^-^ m_K_^-^) *recA1 relA1* *supE44 thi-1* Δ(*lacZYAargFV169*) ᶲ80*lacZ*ΔM15 | Bethesda Research Laboratories |
| JKE201 | MFDpir Δ*mcrA* Δ(*mrr-hsdRMS-mcrBC*) *aac(3)IV*::*lacI^q^*; Erm^R^ | [4] |
| AL1296 | DH5α harbouring pAL99S; Spec^R^ | [3] |
| AL1995 | DH5α harbouring pAL953; Kan^R^ Spec^R^ | [3] |
| AL4487 | JKE201 harbouring pAL614; Amp^R^ Spec^R^ | This study |
|  |  |  |
| Plasmids |  |  |
| pAL99S | *P. multocida­*-*E. coli* expression plasmid; Spec^R^ P*tpiA* | [3] |
| pAL614 | RP4 mobilisable plasmid containing *Himar1*::Spec; RP4 Mob^+^ oriR6K Amp^R^ Spec^R^ tnpC9 |  |
| pAL953 | *P. multocida* vector containing ClosTron group II intron; Spec^R^ Kan^R^ | [3] |
| pAL2019 | pAL953 with the group II intron targeted to *alsT_1*; Kan^R^ Spec^R^ | This study |
| pAL2022 | pAL953 with the group II intron targeted to *crp*; Kan^R^ Spec^R^ | This study |
| pAL2023 | pAL953 with the group II intron targeted to *cyaA*; Kan^R^ Spec^R^ | This study |
| pAL2045 | Wild-type copy of *alsT_1* from M1404 cloned into pAL99S; Spec^R^ | This study |
| pAL2046 | Wild-type copy of *cyaA* from M1404 cloned into pAL99S; Spec^R^ | This study |
| pAL2047 | Wild-type copy of *cyaA* from VP161 cloned into pAL99S; Spec^R^ | This study |
| pAL2048 | Wild-type copy of *crp* from M1404 cloned into pAL99S; Spec^R^ | This study |

1. Wilkie IW, Grimes SE, O'Boyle D, Frost AJ. The virulence and protective efficacy for chickens of *Pasteurella multocida* administered by different routes. Vet Microbiol. 2000;72(1-2):57-68. doi: 10.1016/s0378-1135(99)00187-x.

2. Smallman TR, Williams GC, Harper M, Boyce JD. Genome-wide investigation of *Pasteurella multocida* identifies the stringent response as a negative regulator of hyaluronic acid capsule production. Microbiol Spectr. 2022;10(2):e0019522. doi: 10.1128/spectrum.00195-22.

3. Harper M, St Michael F, John M, Vinogradov E, Steen JA, van Dorsten L, et al. *Pasteurella multocida* Heddleston serovar 3 and 4 strains share a common lipopolysaccharide biosynthesis locus but display both inter- and intrastrain lipopolysaccharide heterogeneity. J Bacteriol. 2013;195(21):4854-64. doi: 10.1128/jb.00779-13.

4. Harms A, Liesch M, Körner J, Québatte M, Engel P, Dehio C. A bacterial toxin-antitoxin module is the origin of inter-bacterial and inter-kingdom effectors of *Bartonella*. PLoS Genet. 2017;13(10):e1007077. doi: 10.1371/journal.pgen.1007077.
