# Supplementary material for: Comparative analysis of shared and unique mechanisms important for diverse strains of Pasteurella multocida to cause systemic infection in mice
Source: PLoS Pathog. 2025 Dec 22;21(12):e1013398. doi: 10.1371/journal.ppat.1013398 (PMC12721544; doi:10.1371/journal.ppat.1013398)
Supplement: S7 Table — (DOCX) [file ppat.1013398.s014.docx]

**S7 Table.** Oligonucleotides used in this study

| Oligonucleotide | Sequence (5’ – 3’)^1^ | Description |
| --- | --- | --- |
| Oligonucleotides used for TraDIS library production | | |
| BAP8034 | P‑G*ATCGGAAGAGCGGTTCAGCAGGTTTTTTTTTTCAAAAAAA*A | Splinkerette adapter top strand {Barquist, 2016 #32} |
| BAP8035 | G*AGATCGGTCTCGGCATTCCTGCTGAACCGCTCTTCCGATC*T | Splinkerette adapter bottom strand {Barquist, 2016 #32} |
| BAP8037 | CAAGCAGAAGACGGCATACGAGAT**TAAGGCGA**GAGATCGGTCTCGGCATTCC | Adapter-specific oligonucleotide used for TraDIS library amplification, contains an index sequence (in bold) and an Illumina P7 sequence at the 5’ |
| BAP8038 | CAAGCAGAAGACGGCATACGAGAT**CGTACTAG**GAGATCGGTCTCGGCATTCC | Adapter-specific oligonucleotide used for TraDIS library amplification, contains an index sequence (in bold) and an Illumina P7 sequence at the 5’ |
| BAP8039 | CAAGCAGAAGACGGCATACGAGAT**AGGCAGAA**GAGATCGGTCTCGGCATTCC | Adapter-specific oligonucleotide used for TraDIS library amplification, contains an index sequence (in bold) and an Illumina P7 sequence at the 5’ |
| BAP8040 | CAAGCAGAAGACGGCATACGAGAT**TCCTGAGC**GAGATCGGTCTCGGCATTCC | Adapter-specific oligonucleotide used for TraDIS library amplification, contains an index sequence (in bold) and an Illumina P7 sequence at the 5’ |
| BAP8042 | GGTTCTAGAGACCGGGGACTTATCAGC | Custom *Himar1* transposon-specific Illumina sequencing oligonucleotide |
| BAP8043 | TTCAGCAGGAATGCCGAGACCGATCTC | Custom adapter index-specific Illumina sequencing oligonucleotide |
| BAP8335 | CAAGCAGAAGACGGCATACGAGAT**GGACTCCT**GAGATCGGTCTCGGCATTCC | Adapter-specific oligonucleotide used for TraDIS library amplification, contains an index sequence (in bold) and an Illumina P7 sequence at the 5’ |
| BAP8336 | CAAGCAGAAGACGGCATACGAGAT**TAGGCATG**GAGATCGGTCTCGGCATTCC | Adapter-specific oligonucleotide used for TraDIS library amplification, contains an index sequence (in bold) and an Illumina P7 sequence at the 5’ |
| BAP8337 | CAAGCAGAAGACGGCATACGAGAT**CTCTCTAC**GAGATCGGTCTCGGCATTCC | Adapter-specific oligonucleotide used for TraDIS library amplification, contains an index sequence (in bold) and an Illumina P7 sequence at the 5’ |
| BAP8338 | CAAGCAGAAGACGGCATACGAGAT**CAGAGAGG**GAGATCGGTCTCGGCATTCC | Adapter-specific oligonucleotide used for TraDIS library amplification, contains an index sequence (in bold) and an Illumina P7 sequence at the 5’ |
| BAP8339 | CAAGCAGAAGACGGCATACGAGAT**GCTACGCT**GAGATCGGTCTCGGCATTCC | Adapter-specific oligonucleotide used for TraDIS library amplification, contains an index sequence (in bold) and an Illumina P7 sequence at the 5’ |
| BAP8350 | AATGATACGGCGACCACCGAGATCTACACCTCTAGAAAGTATAGGAACTTCGAACCG | *Himar1*-specific oligonucleotide for TraDIS library amplification, contains Illumina P5 at the 5’ end |
| BAP8356 | CAAGCAGAAGACGGCATACGAGAT**CGAGGCTG**GAGATCGGTCTCGGCATTCC | Adapter-specific oligonucleotide used for TraDIS library amplification, contains an index sequence (in bold) and an Illumina P7 sequence at the 5’ |
| BAP8357 | CAAGCAGAAGACGGCATACGAGAT**AAGAGGCA**GAGATCGGTCTCGGCATTCC | Adapter-specific oligonucleotide used for TraDIS library amplification, contains an index sequence (in bold) and an Illumina P7 sequence at the 5’ |
| BAP8358 | CAAGCAGAAGACGGCATACGAGAT**GTAGAGGA**GAGATCGGTCTCGGCATTCC | Adapter-specific oligonucleotide used for TraDIS library amplification, contains an index sequence (in bold) and an Illumina P7 sequence at the 5’ |
| BAP9946 | CAAGCAGAAGACGGCATACGAGAT**ATCACGAC**GAGATCGGTCTCGGCATTCC | Adapter-specific oligonucleotide used for TraDIS library amplification, contains an index sequence (in bold) and an Illumina P7 sequence at the 5’ |
| BAP9947 | CAAGCAGAAGACGGCATACGAGAT**ACAGTGGT**GAGATCGGTCTCGGCATTCC | Adapter-specific oligonucleotide used for TraDIS library amplification, contains an index sequence (in bold) and an Illumina P7 sequence at the 5’ |
| BAP9948 | CAAGCAGAAGACGGCATACGAGAT**ACCCAGCA**GAGATCGGTCTCGGCATTCC | Adapter-specific oligonucleotide used for TraDIS library amplification, contains an index sequence (in bold) and an Illumina P7 sequence at the 5’ |
| BAP10005 | CAAGCAGAAGACGGCATACGAGAT**CAGATCCA**GAGATCGGTCTCGGCATTCC | Adapter-specific oligonucleotide used for TraDIS library amplification, contains an index sequence (in bold) and an Illumina P7 sequence at the 5’ |
| BAP10006 | CAAGCAGAAGACGGCATACGAGAT**ACAAACGG**GAGATCGGTCTCGGCATTCC | Adapter-specific oligonucleotide used for TraDIS library amplification, contains an index sequence (in bold) and an Illumina P7 sequence at the 5’ |
| BAP10007 | CAAGCAGAAGACGGCATACGAGAT**AACCCCTC**GAGATCGGTCTCGGCATTCC | Adapter-specific oligonucleotide used for TraDIS library amplification, contains an index sequence (in bold) and an Illumina P7 sequence at the 5’ |
| BAP10008 | CAAGCAGAAGACGGCATACGAGAT**CCCAACCT**GAGATCGGTCTCGGCATTCC | Adapter-specific oligonucleotide used for TraDIS library amplification, contains an index sequence (in bold) and an Illumina P7 sequence at the 5’ |
| BAP10009 | CAAGCAGAAGACGGCATACGAGAT**CACCACAC**GAGATCGGTCTCGGCATTCC | Adapter-specific oligonucleotide used for TraDIS library amplification, contains an index sequence (in bold) and an Illumina P7 sequence at the 5’ |
| BAP10010 | CAAGCAGAAGACGGCATACGAGAT**GAAACCCA**GAGATCGGTCTCGGCATTCC | Adapter-specific oligonucleotide used for TraDIS library amplification, contains an index sequence (in bold) and an Illumina P7 sequence at the 5’ |
| BAP10011 | CAAGCAGAAGACGGCATACGAGAT**TGTGACCA**GAGATCGGTCTCGGCATTCC | Adapter-specific oligonucleotide used for TraDIS library amplification, contains an index sequence (in bold) and an Illumina P7 sequence at the 5’ |
| BAP10012 | CAAGCAGAAGACGGCATACGAGAT**AGGGTCAA**GAGATCGGTCTCGGCATTCC | Adapter-specific oligonucleotide used for TraDIS library amplification, contains an index sequence (in bold) and an Illumina P7 sequence at the 5’ |
| BAP10013 | CAAGCAGAAGACGGCATACGAGAT**AGGAGTGG**GAGATCGGTCTCGGCATTCC | Adapter-specific oligonucleotide used for TraDIS library amplification, contains an index sequence (in bold) and an Illumina P7 sequence at the 5’ |
| BAP10014 | CAAGCAGAAGACGGCATACGAGAT**TAGATCGC**GAGATCGGTCTCGGCATTCC | Adapter-specific oligonucleotide used for TraDIS library amplification, contains an index sequence (in bold) and an Illumina P7 sequence at the 5’ |
| BAP10015 | CAAGCAGAAGACGGCATACGAGAT**CTCTCTAT**GAGATCGGTCTCGGCATTCC | Adapter-specific oligonucleotide used for TraDIS library amplification, contains an index sequence (in bold) and an Illumina P7 sequence at the 5’ |
| BAP10016 | CAAGCAGAAGACGGCATACGAGAT**TATCCTCT**GAGATCGGTCTCGGCATTCC | Adapter-specific oligonucleotide used for TraDIS library amplification, contains an index sequence (in bold) and an Illumina P7 sequence at the 5’ |
| BAP10017 | CAAGCAGAAGACGGCATACGAGAT**AGAGTAGA**GAGATCGGTCTCGGCATTCC | Adapter-specific oligonucleotide used for TraDIS library amplification, contains an index sequence (in bold) and an Illumina P7 sequence at the 5’ |
| BAP10018 | CAAGCAGAAGACGGCATACGAGAT**GTAAGGAG**GAGATCGGTCTCGGCATTCC | Adapter-specific oligonucleotide used for TraDIS library amplification, contains an index sequence (in bold) and an Illumina P7 sequence at the 5’ |
| BAP10019 | CAAGCAGAAGACGGCATACGAGAT**ACTGCATA**GAGATCGGTCTCGGCATTCC | Adapter-specific oligonucleotide used for TraDIS library amplification, contains an index sequence (in bold) and an Illumina P7 sequence at the 5’ |
| BAP10020 | CAAGCAGAAGACGGCATACGAGAT**AAGGAGTA**GAGATCGGTCTCGGCATTCC | Adapter-specific oligonucleotide used for TraDIS library amplification, contains an index sequence (in bold) and an Illumina P7 sequence at the 5’ |
| BAP10021 | CAAGCAGAAGACGGCATACGAGAT**CTAAGCCT**GAGATCGGTCTCGGCATTCC | Adapter-specific oligonucleotide used for TraDIS library amplification, contains an index sequence (in bold) and an Illumina P7 sequence at the 5’ |
| ClosTron mutagenesis | | |
| BAP6544 | CGAAATTAGAAACTTGCGTTCAGTAAAC | EBS universal primer, used to retarget the group II intron to a specific target and for Sanger sequencing to confirm a single insertion |
| BAP8243 | TACGAGTACTCCGTACCCTTGCAAG | Oligonucleotide for Sanger sequencing to confirm correct retargeting of pAL953 |
| BAP10089 | AAAAAAGCTTATAATTATCCTTAGTGGCCGTCCAGGTGCGCCCAGATAGGGTG | IBS ClosTron oligonucleotide for retargeting the group II intron targeting region to *alsT_1*, contains a *Hin*dIII restriction site |
| BAP10090 | CAGATTGTACAAATGTGGTGATAACAGATAAGTCGTCCAGCCTAACTTACCTTTCTTTGT | EBS1d ClosTron oligonucleotide for retargeting the group II intron targeting region to *alsT_1*, contains a *Bsr*GI restriction site |
| BAP10091 | TGAACGCAAGTTTCTAATTTCGATTGCCACTCGATAGAGGAAAGTGTCT | EBS2 ClosTron oligonucleotide for retargeting the group II intron targeting region to *alsT_1* |
| BAP10109 | AAAAAAGCTTATAATTATCCTTAGGCAACGAAATGGTGCGCCCAGATAGGGTG | IBS ClosTron oligonucleotide for retargeting the group II intron targeting region to *crp*, contains a *Hin*dIII restriction site |
| BAP10110 | CAGATTGTACAAATGTGGTGATAACAGATAAGTCGAAATGATTAACTTACCTTTCTTTGT | EBS1d ClosTron oligonucleotide for retargeting the group II intron targeting region to *crp*, contains a *Bsr*GI restriction site |
| BAP10111 | TGAACGCAAGTTTCTAATTTCGATTTTGCCTCGATAGAGGAAAGTGTCT | EBS2 ClosTron oligonucleotide for retargeting the group II intron targeting region to *crp* |
| BAP10112 | AAAAAAGCTTATAATTATCCTTAAATCTCCCCGGCGTGCGCCCAGATAGGGTG | IBS ClosTron oligonucleotide for retargeting the group II intron targeting region to *cyaA*, contains a *Hin*dIII restriction site |
| BAP10113 | CAGATTGTACAAATGTGGTGATAACAGATAAGTCCCCGGCTATAACTTACCTTTCTTTGT | EBS1d ClosTron oligonucleotide for retargeting the group II intron targeting region to *cyaA*, contains a *Bsr*GI restriction site |
| BAP10114 | TGAACGCAAGTTTCTAATTTCGGTTAGATTCCGATAGAGGAAAGTGTCT | EBS2 ClosTron oligonucleotide for retargeting the group II intron targeting region to *cyaA* |
| Complementation plasmid production | | |
| BAP10092 | AAAAAACCCGGGAGGAGGAATAATGAGCATATTTTCTAC | Forward oligonucleotide flanking *alsT_1*, contains an *Xma*I restriction site |
| BAP10093 | AAAAAAAAGCTTTTAAGACCAAATATCGTTATCGAC | Reverse oligonucleotide flanking *alsT_1*, contains an *Hin*dIII restriction site |
| BAP10115 | AAAAAAGGATCCGTGAGTGGAATATCATTTGAATTAC | Forward oligonucleotide flanking *cyaA*, contains an *Bam*HI restriction site |
| BAP10116 | AAAAAAAAGCTTTTATGACATCGCTAATCGACTG | Reverse oligonucleotide flanking *cyaA*, contains an *Hin*dIII restriction site |
| BAP10117 | AAAAAAGGATCCATGGAGGTCTTCCGTGCAAG | Forward oligonucleotide flanking *crp*, contains an *Bam*HI restriction site |
| BAP10118 | AAAAAAAAGCTTGATTATCTTGTACCGTAAACGAC | Reverse oligonucleotide flanking *crp*, contains an *Hin*dIII restriction site |

^1^P- represents a 5’ phosphate and * represents a phosphorothioate bond
