# Supplementary material for: Comparative analysis of shared and unique mechanisms important for diverse strains of Pasteurella multocida to cause systemic infection in mice
Source: PLoS Pathog. 2025 Dec 22;21(12):e1013398. doi: 10.1371/journal.ppat.1013398 (PMC12721544; doi:10.1371/journal.ppat.1013398)
Supplement: S8 Table — (DOCX) [file ppat.1013398.s015.docx]

**S8 Table.** Prophage regions identified in the *de novo* assembled M1404 genome

| Region | Length | PHASTER score | Coding sequences | Genome position in nucleotides | Homolog | GC % |
| --- | --- | --- | --- | --- | --- | --- |
| M1404_1 |  |  |  |  |  |  |
| 1 | 73.5Kb | 150 | 99 | 95,238-168,755 | NC_000929 | 42.35 |
| 2 | 33.5Kb | 150 | 48 | 222,212-255,789 | NC_027382 | 42.44 |
| 3 | 36.7Kb | 150 | 49 | 403,519-440,233 | NC_027382 | 42.11 |
| 4 | 15.6Kb | 110 | 18 | 2,243,343-2,259,040 | NC_031940 | 38.06 |
| M1404_2 |  |  |  |  |  |  |
| 5 | 34.5Kb | 150 | 51 | 24,192-58,714 | NC_000929 | 42.36 |
