# Supplementary material for: Comparative analysis of shared and unique mechanisms important for diverse strains of Pasteurella multocida to cause systemic infection in mice
Source: PLoS Pathog. 2025 Dec 22;21(12):e1013398. doi: 10.1371/journal.ppat.1013398 (PMC12721544; doi:10.1371/journal.ppat.1013398)
Supplement: S1 Fig — Created in BioRender. Boyce, J. (2025) https://BioRender.com/iqy3pkk. (DOCX) [file ppat.1013398.s016.docx]

S1 Fig


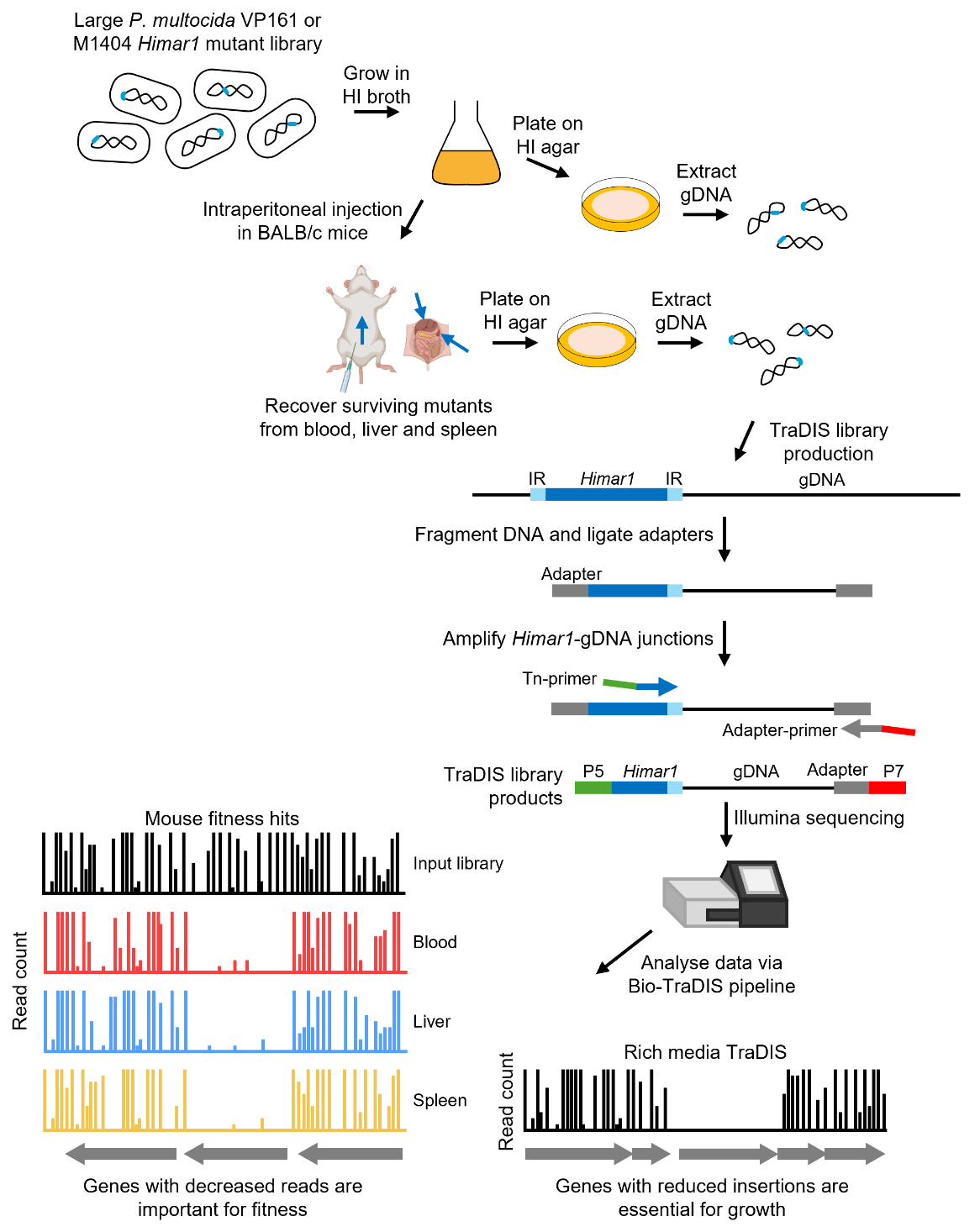


**S1 Fig.** Overview of the TraDIS methodology from this study. A large *P. multocida* strain M1404 *Himar1* mutant library was generated via conjugation. The M1404 *Himar1* library, along with a previously produced VP161 *Himar1* mutant library, were used to identify genes essential for growth in rich medium and also to perform systemic infections in BALB/c mice. Surviving mutants were recovered from the blood, liver, and spleen from mice, and plated onto heart infusion agar. Mutants were recovered, and genomic DNA (gDNA) extracted to produce TraDIS libraries. The gDNA was fragmented by sonication, end-repaired, and adapters ligated onto all fragments. Transposon-chromosome junctions were amplified using oligonucleotides specific to the *Himar1* inverted repeat and adapter, with oligonucleotides containing Illumina P5 and P7 sequences. The TraDIS libraries were then sequenced using an Illumina NextSeq, with data analysed using the Bio-TraDIS toolkit and related scripts. The normalized number of unique insertion sites per gene was compared to identify genes important for growth in rich media. Normalised read counts per gene were compared between the rich media TraDIS libraries, and each of the bloodstream, liver, and spleen TraDIS libraries to identify genes that result in either a fitness cost or benefit when disrupted by transposon insertions.
