# Supplementary material for: Comparative analysis of shared and unique mechanisms important for diverse strains of Pasteurella multocida to cause systemic infection in mice
Source: PLoS Pathog. 2025 Dec 22;21(12):e1013398. doi: 10.1371/journal.ppat.1013398 (PMC12721544; doi:10.1371/journal.ppat.1013398)
Supplement: S2 Fig — (DOCX) [file ppat.1013398.s017.docx]

S2 Fig


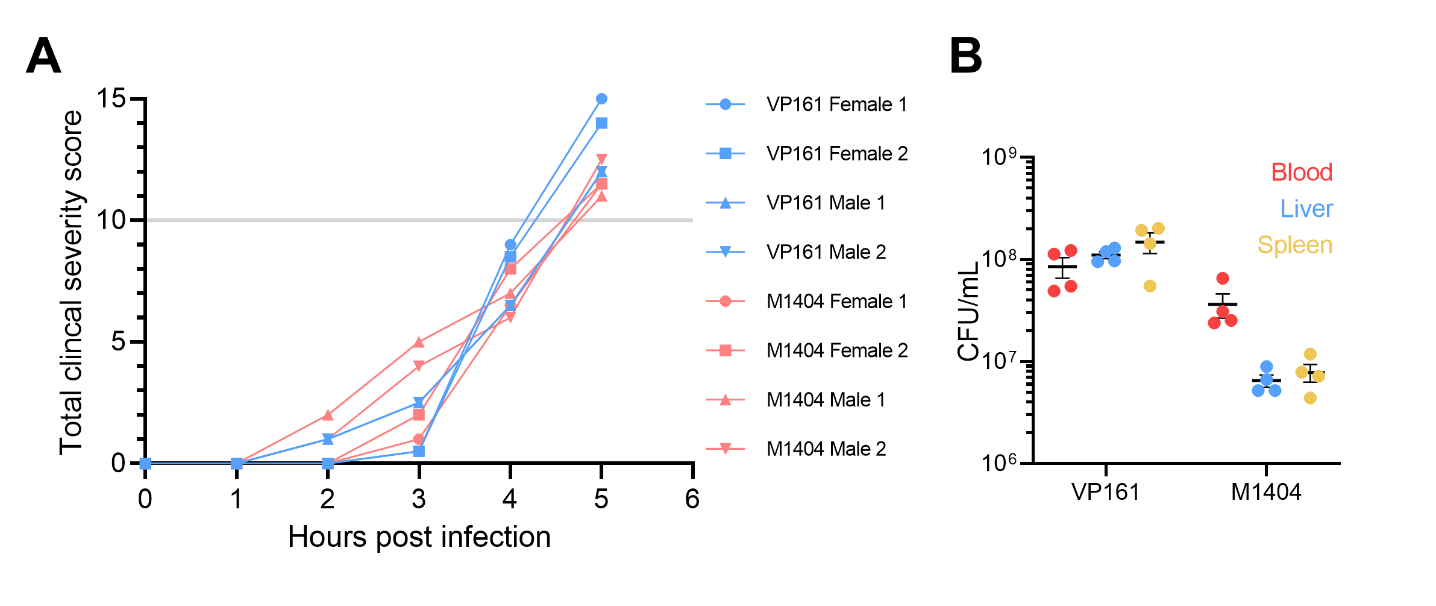


**S2 Fig.** Mouse systemic infections with the *P. multocida* strain VP161 and M1404 *Himar1* mutant libraries. For each library, two male and two female 6-10-week-old BALB/c mice were injected intraperitoneally with ~ 2 x 10^7^ CFU. **A.** Mice were monitored for clinical signs of systemic infection, with mice reaching the humane endpoint when they had a total score above 10 (grey line). **B.** Surviving VP161 or M1404 *Himar1* mutants were recovered from the bloodstream, liver, and spleen. Blood was resuspended in a total volume of 1 mL, and liver and spleen samples were homogenized in 1 mL of 1 x PBS, before being plated onto heart infusion agar.
