# Supplementary material for: Comparative analysis of shared and unique mechanisms important for diverse strains of Pasteurella multocida to cause systemic infection in mice
Source: PLoS Pathog. 2025 Dec 22;21(12):e1013398. doi: 10.1371/journal.ppat.1013398 (PMC12721544; doi:10.1371/journal.ppat.1013398)
Supplement: S3 Fig — (DOCX) [file ppat.1013398.s018.docx]

S3 Fig


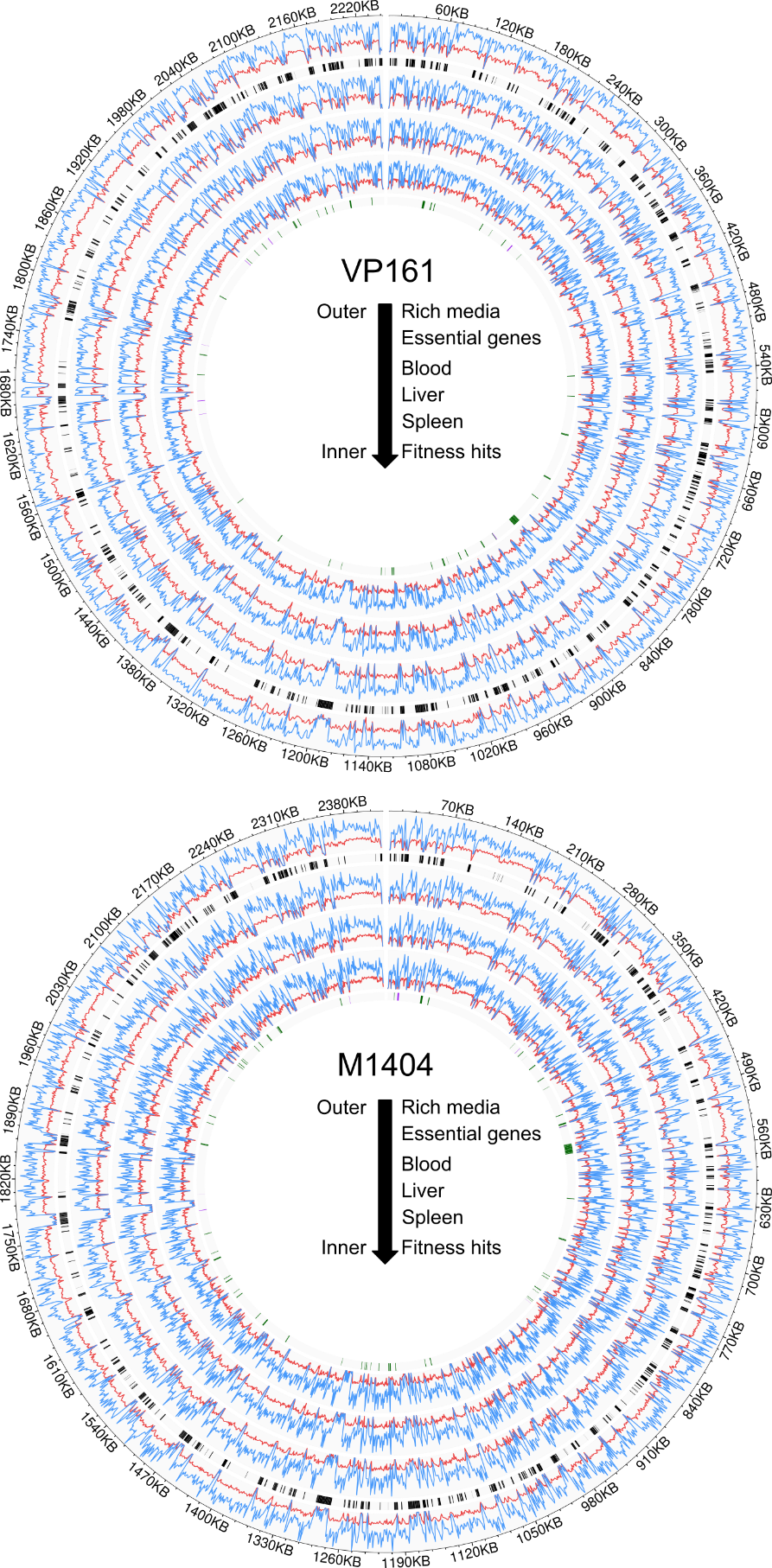


**S3 Fig.** Log_2_ read count (blue) and unique insertion sites (red) per 1 kb across the *P. multocida* VP161 and M1404 genome. Genes identified as essential for growth in rich media are shown as black bars in the second from outer ring, while fitness-cost genes are shown as green bars and fitness-benefit genes shown as purple bars in the inner ring.
