# Supplementary material for: Comparative analysis of shared and unique mechanisms important for diverse strains of Pasteurella multocida to cause systemic infection in mice
Source: PLoS Pathog. 2025 Dec 22;21(12):e1013398. doi: 10.1371/journal.ppat.1013398 (PMC12721544; doi:10.1371/journal.ppat.1013398)
Supplement: S4 Fig — (DOCX) [file ppat.1013398.s019.docx]

S4 Fig


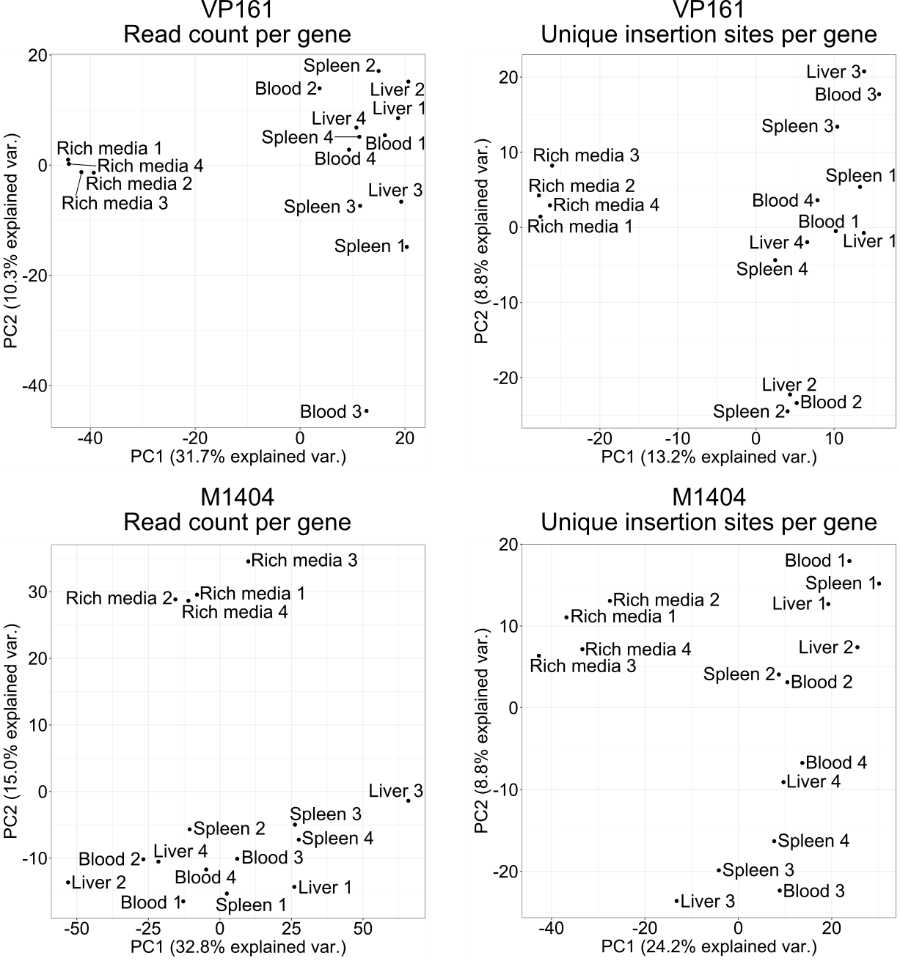


**S4 Fig.** Principal component analysis (PCA) plots for all VP161 and M1404 TraDIS libraries in this study. PCA was performed using prcomp in R, using either the number of unique insertion sites per gene or the number of reads per gene.
