# Supplementary material for: Comparative analysis of shared and unique mechanisms important for diverse strains of Pasteurella multocida to cause systemic infection in mice
Source: PLoS Pathog. 2025 Dec 22;21(12):e1013398. doi: 10.1371/journal.ppat.1013398 (PMC12721544; doi:10.1371/journal.ppat.1013398)
Supplement: S5 Fig — (DOCX) [file ppat.1013398.s020.docx]

S5 Fig


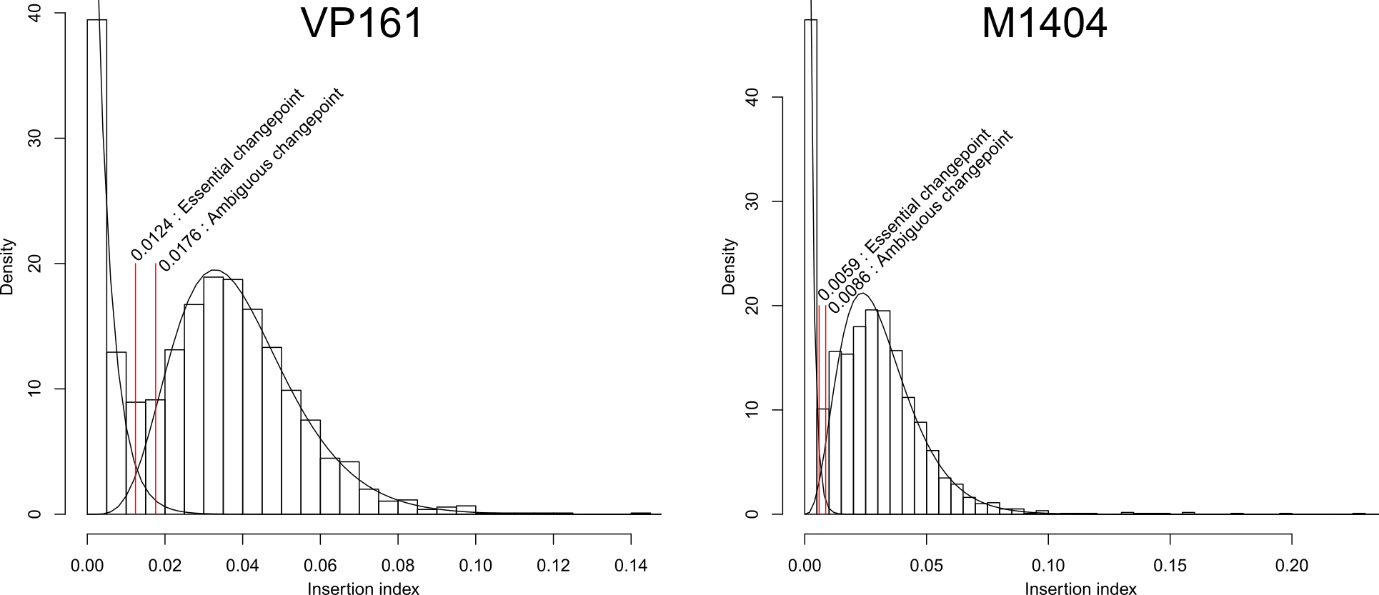


**S5 Fig.** Histograms of unique insertion sites (UIS) per gene and identification of essential gene cut-offs in the *P. multocida* strain VP161 and M1404 rich media TraDIS libraries. The number of UIS is divided by gene length to identify the insertion index, a normalized UIS count per gene. Histograms are generated using the insertion index from all genes, generating a bimodal distribution of essential and non-essential genes. Normal curves are drawn for the two sets, and the intersect between the normal curves is taken as the cut-off for gene essentiality.
