# Supplementary material for: Comparative analysis of shared and unique mechanisms important for diverse strains of Pasteurella multocida to cause systemic infection in mice
Source: PLoS Pathog. 2025 Dec 22;21(12):e1013398. doi: 10.1371/journal.ppat.1013398 (PMC12721544; doi:10.1371/journal.ppat.1013398)
Supplement: S6 Fig — (DOCX) [file ppat.1013398.s021.docx]

S6 Fig


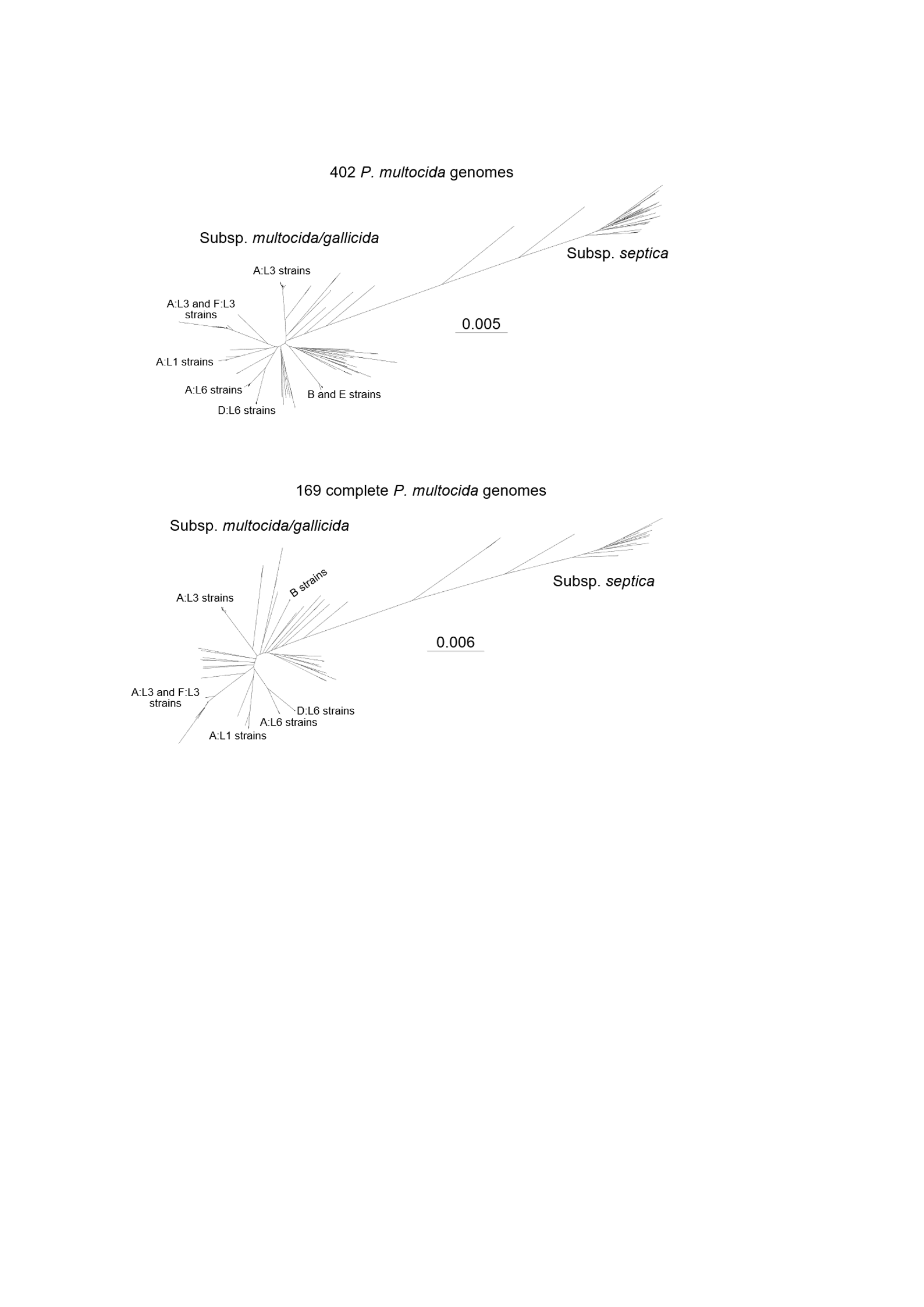


**S6 Fig.** Maximum-likelihood core-genome phylogeny of the 169 complete *P. multocida* genomes used to identify the 100% *P. multocida* core genome. Roary was used to generate a core-genome alignment using 850,282 sites, and IQ-TREE was used to generate the phylogeny (using model GTR+F+R10), with 1,000 bootstrap replicates (see supplemental material 6 for bootstrap values). Scale bar represents number of nucleotide substitutions per site. The maximum-likelihood core-genome phylogeny of 402 *P. multocida* strains, including incomplete genomes, was included as a comparison.
