# Supplementary material for: Comparative analysis of shared and unique mechanisms important for diverse strains of Pasteurella multocida to cause systemic infection in mice
Source: PLoS Pathog. 2025 Dec 22;21(12):e1013398. doi: 10.1371/journal.ppat.1013398 (PMC12721544; doi:10.1371/journal.ppat.1013398)
Supplement: S7 Fig — (DOCX) [file ppat.1013398.s022.docx]

S7 Fig


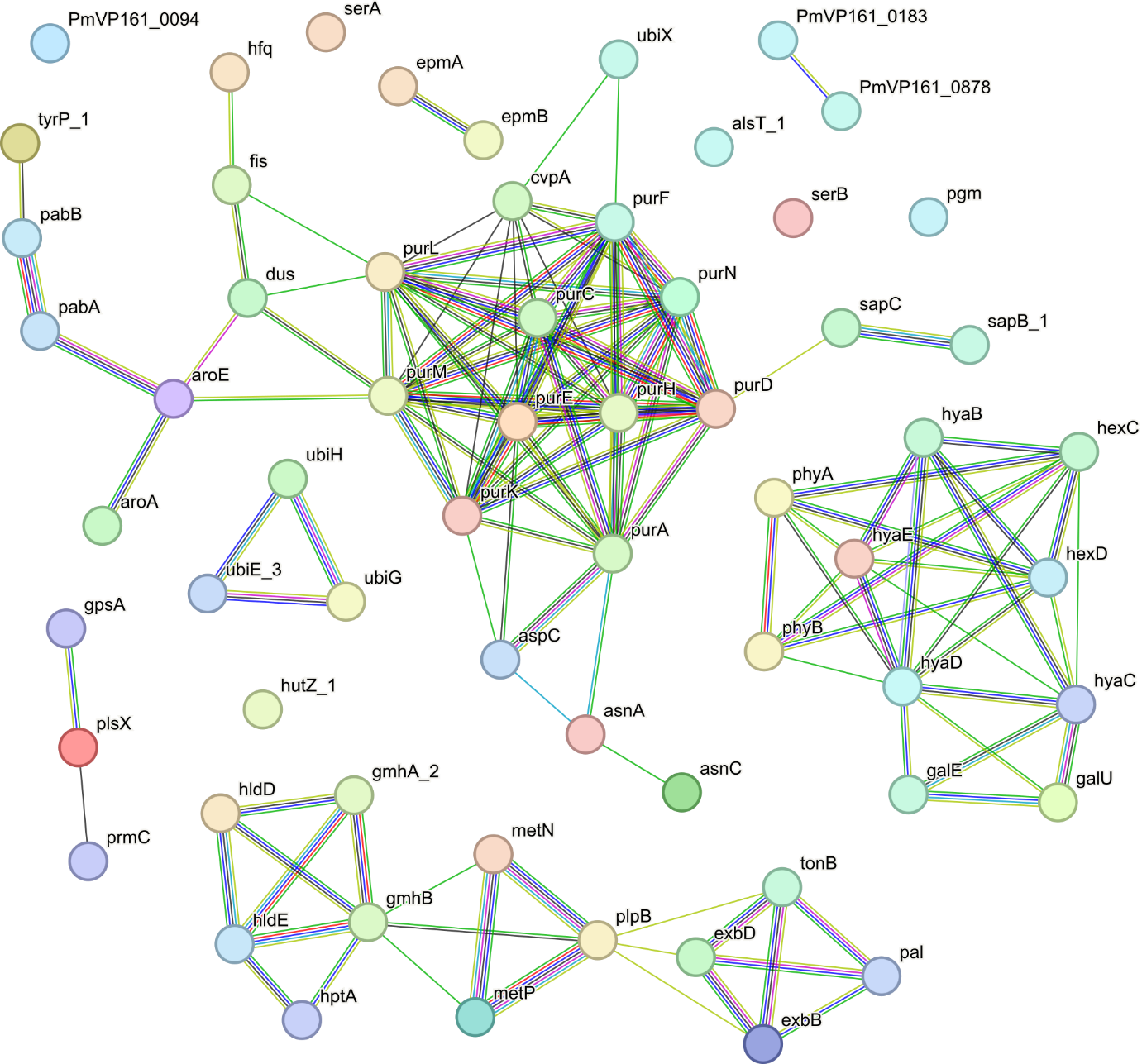


**S7 Fig.** STRING interaction map for *P. multocida* strain VP161 *in vivo* fitness genes. Genes were included if they were identified as important for VP161 survival in the bloodstream, liver, or spleen of BALB/c mice. The protein sequences of *in vivo* fitness genes were matched to *P. multocida* strain Pm70 in string, with all 63 genes matching to a Pm70 protein. The lines between genes show the type of evidence for interaction; red line - fusion evidence, green line - neighbourhood evidence, blue line - co-occurrence evidence, purple line - experimental evidence, yellow line - text-mining evidence, light blue line - database evidence, black line - co-expression evidence.
