# Supplementary material for: Comparative analysis of shared and unique mechanisms important for diverse strains of Pasteurella multocida to cause systemic infection in mice
Source: PLoS Pathog. 2025 Dec 22;21(12):e1013398. doi: 10.1371/journal.ppat.1013398 (PMC12721544; doi:10.1371/journal.ppat.1013398)
Supplement: S9 Fig — (DOCX) [file ppat.1013398.s024.docx]

S9 Fig


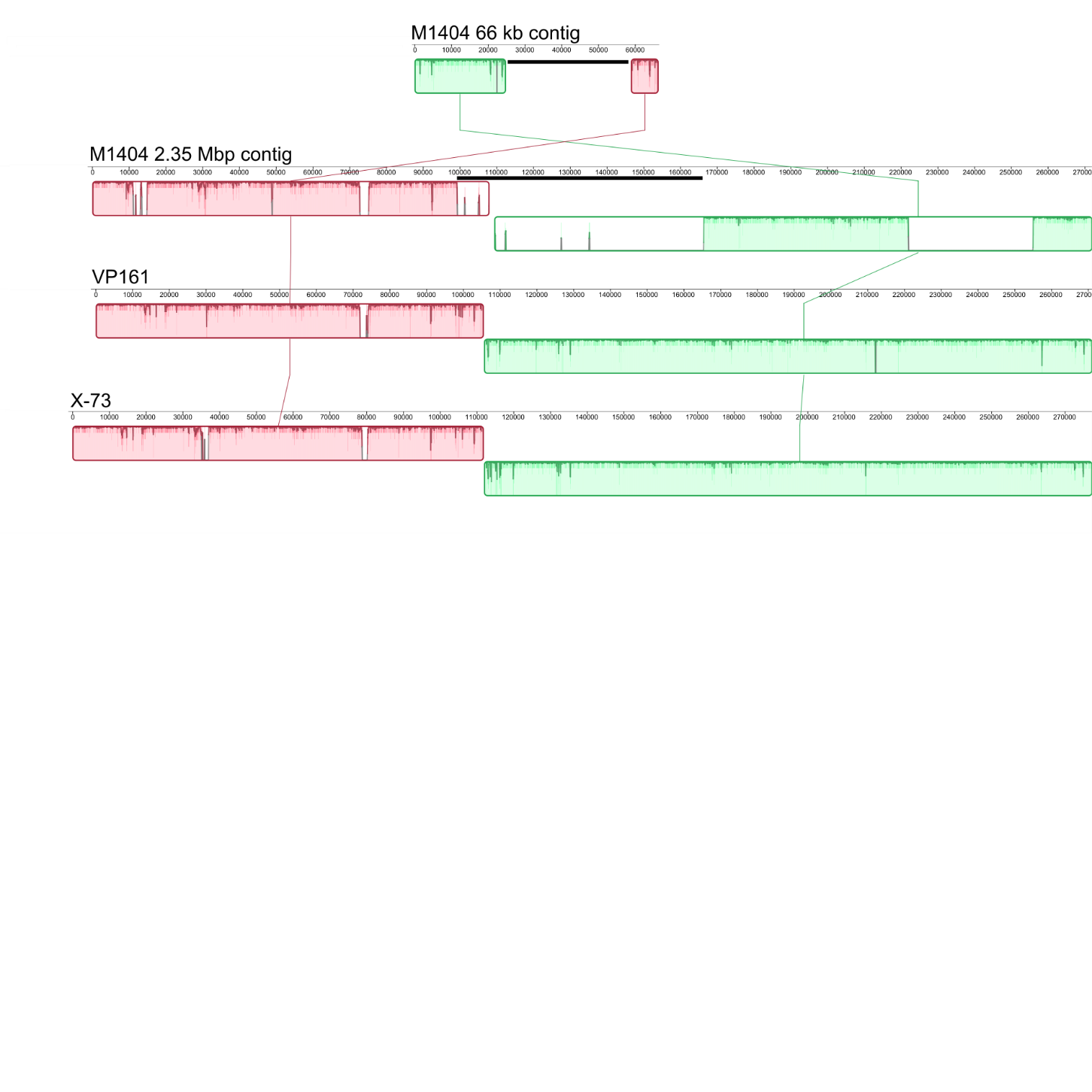


**S9 Fig.** Mauve alignment showing colinear parts of the 2.35 Mbp and 66 kb contigs generated in the *P. multocida* strain M1404 assembly with strains VP161 and X-73. The prophage regions are highlighted by black lines.
