# Supplementary material for: Comparative analysis of shared and unique mechanisms important for diverse strains of Pasteurella multocida to cause systemic infection in mice
Source: PLoS Pathog. 2025 Dec 22;21(12):e1013398. doi: 10.1371/journal.ppat.1013398 (PMC12721544; doi:10.1371/journal.ppat.1013398)
Supplement: Attachment — Submitted filename: Plos_pathogens_response_to_reviewers_comments_v3.docx [file ppat.1013398.s026.docx]

**Response to reviewers comments**

We thank the reviewers for their comments and suggestions. In this document the reviewers’ comments are in bold, and our responses and any changes to the manuscript are in normal text under the reviewers’ comments.

**Reviewers' Comments:**

**Part I – Summary**

**Reviewer #2: The study would also be more impactful if placed in the context of a broader Gram-negative systemic infection field in addition to the Pasteurella field.**

See response to reviewer #2 question 3

**Part II – Major Issues: Key Experiments Required for Acceptance**

**Reviewer #1: I have no major issues.**

**Reviewer #2:**

**1. Genetic complementation throughout the study is not sufficient. In Figure 4, the experimental design should include complementation to ensure that the shift in virulence is specifically due to the described gene and not an operon off-target effect.**

We agree that complementation is important to confirm observed phenotypes. We have constructed complemented strains and assessed these strains for restoration of phenotypes in the *in vitro* assays. Monash University is committed to the ethical, humane and responsible use of animals and expects all researchers to prioritize replacement, reduction and refinement for all animal experiments conducted. Our *in vitro* experiments using the complemented strains clearly show that *crp* and *cyaA* are essential for capsule production. Moreover, there is an extensive body of literature already showing that capsule is a major virulence determinant for *Pasteurella multocida*. Furthermore, the expression plasmid used can be lost in up to 10% of the population when grown for 16 h without selection. We believe it is not appropriate to use this complementation system *in vivo* without further refinement. We therefore consider that the use of additional animals to test these complemented strains outside our allowable animal ethics requirements. However, we have modified the text as follows to reflect that complemented mutants have not been tested for virulence.

- Removed “selected” on line 224
- Changed “confirming” to “agreeing with” on line 235
- Changed “confirming” to “suggesting” on line 310
- Added “Complementation was not performed to reduce the number of mice used in this study” on lines 310-311

Additionally, there is strong previous evidence that the mutagenesis system we are using is target gene-specific and does not result in polar effects on downstream genes. The mutants used in this study were generated by insertion of a group II intron, using the ClosTron (also known as TargeTron) system. This group II intron does not contain any transcriptional terminators, so insertion into a target gene should not impact expression of downstream genes. Indeed, we have successfully utilised this system previously for inactivation of both capsule and lipopolysaccharide biosynthesis genes, where genes are encoded in large operons (10.1128/jb.00779-13, 10.1128/spectrum.00195-22). In these studies, we found that insertion of the intron into target genes within the operon did not disrupt expression of other capsule or lipopolysaccharide genes within the region, as confirmed by the restoration of phenotype using single gene complementation.

**In Figure 5A, the crp complementation is marginal. Is this within the reliable reading range of the plate reader? There appears to only be a ~0.002 difference in absorbance which is considered well beyond the linear detectable range for most absorbance-based approaches.**

We apologise for this error. Re-examination of the graph following this reviewer’s comment has revealed that we did not label the y-axis of this graph correctly. This graph should be displaying absorbance/mL of culture. Therefore, the raw absorbance values are more than 10-fold higher. These values are well within the reliable reading range of the spectrophotometer, and this is confirmed by the tight clustering of readings and the small error bars. We have modified the Y-axis title of Fig 5A to “Absorbance (640nm) per mL of culture” and the Y-axis title of Fig 5B to “Hyaluronic acid amounts (μg) per mL of culture” and modified the text in the accompanying figure legend.

Additionally, we have added additional text to the manuscript:

- “(OD_600_ ~15)” to the materials and methods section on line 570
- “For both VP161 and M1404, hyaluronic acid or absorbance values were normalised by the optical density of the input culture used to give hyaluronic acid or absorbance per 1 mL of culture” on lines 577-579

Partial restoration of a phenotype in a complementation strain (i.e. increasing but not restoring capsule production to wild-type levels) is not uncommon for these types of experiments in bacteria. For our experiments in *P. multocida* we typically clone the wild-type gene into pAL99- or pPBA1100-based plasmids (10.1371/journal.ppat.1000750, 10.1128/spectrum.00195-22, 10.1128/spectrum.03805-23, 10.1128/iai.00122-16). The strengths of the plasmid-encoded promoters as well as the copy number of the plasmid, and thus the amount of the recombinant protein provided to the strain during complementation studies, is variable. In this specific instance, CRP activity requires cAMP and in the complemented *crp* mutant, it is likely that the strain has increased CRP production compared to the wild-type, but the corresponding amount of cAMP available in the cell may be insufficient to fully stimulate CRP activity in all CRP proteins.

**2. It is exciting that many hits in this TnSeq study have been identified in other Gram-negative species. This indicates larger trends in the bacterial pathogenesis field that specific mechanisms may be conserved across species in systemic infection. In the Discussion, the authors should place their findings in the context of those from other groups who have used similar approaches in different species. A few examples could include: PMID35762751, PMID39178317, PMID37463183, PMID30087402, PMID31009518.**

We were mindful of the length of this research article. Given the amount of data presented, we focused our discussions on results primarily relevant to *P. multocida* research. However, the reviewer makes a good point to further contextualise our results with similar results from Gram negative species. We agree that comparison with other research showing *gmhB* being important for *in vivo* fitness in multiple other Gram-negative pathogens is a key comparison that should be included. We have added “Identification of *gmhB*, involved in ADP-heptose synthesis, was identified as important for *in vivo* fitness of both strains. Previous studies have shown that *gmhB* is important for fitness of several Enterobacterales species in mice [71, 72], indicating that ADP-heptose synthesis is likely important for virulence in several Gammaproteobactera species.” into the discussion on lines 419-423. We have also added into the discussion that *crp* was identified as important for *K. pneumoniae* fitness by TraDIS on lines 378-380.

**Reviewer #3: 1. Line 117: The authors employed normalized UIS counts, which is a commonly used method for identifying essential genes. However, considering that transposon insertions may exhibit certain positional biases, is there a risk of misclassification, such as false positives or false negatives? To my understanding, essential genes are typically defined as those whose knockout results in lethality. Therefore, directly defining genes identified through this non-targeted knockout approach as essential genes may not be entirely appropriate.**

It is true that essentiality as defined by TraDIS screens is not identical to essentiality as defined by the inability to delete the gene. In this study, a gene is called as essential when the gene has reduced or no transposon mutants recovered. The VP161 and M1404 *Himar1* mutant libraries used in this study had, on average, 31 and 24 unique insertion sites (UIS) per kb, respectively. Importantly, we saw no positional bias of unique insertion sites or number of insertions per kb across the entire genome (S3 Fig). As such, we are confident that we are not getting false positives or false negatives due to *Himar1* insertion bias.

We used the Bio-TraDIS toolkit for identification of essential genes. The Bio-TraDIS pipeline identifies essential genes by plotting normalised unique insertion sites (called insertion indexes) as a histogram. This histogram generates a bimodal distribution; essential genes that have no, or very few, transposon insertions cluster to the left, and non-essential genes that have several unique insertions cluster to the right. Two normal curves are drawn, with the intersect used as the cut-off value to determine essentiality. Comparing a histogram of insertion indexes in this way is a very commonly used method for identifying essential genes via TraDIS (10.1101/gr.097097.109, 10.1038/s41598-017-05795-y, 10.1073/pnas.1519220112, 10.1128/jb.00432-20, 10.1128/jb.00630-18, 10.1186/s12866-023-02835-8). Even for essential genes, transposon insertion sites near the end of the gene may be tolerated and even for inactivating insertion sites, some transposon mutants may be recovered at very low levels due to the different times to death for different classes of essential genes (see [10.1073/pnas.2001507117](https://doi.org/10.1073/pnas.2001507117)). For both strains, most of the genes identified as essential clustered far to the left. For VP161, the insertion index cut-off for essentiality was 0.0124, and 468 of the 585 genes identified as essential had an insertion index less than half that of the cut off (representing ≤ 5 unique insertion sites in a 1 kb gene). For M1404, the insertion index cut-off for essentiality was 0.0059, and 458 of the 585 genes identified as essential had an insertion index less than half that of the cut off (representing ≤ 2 unique insertion sites in a 1 kb gene). In our previous TraDIS study, using an identical method to call essential genes, we compared protein encoding genes identified as essential to the database of essential genes (DEG), which contains genes identified as essential by TraDIS-based methods and by individual knockouts, finding that 93% of genes identified as essential in our study had a homolog in the DEG. While there may be some false positives near the insertion index cut-off, we are confident that this method produces very few false positives.

However, for clarity regarding how we have called essential genes, we have modified the following:

- Added “identifying genes that resulted in lethality or a severe fitness defect when insertionally inactivated” on lines 121-122 in the results
- Modified the materials and methods on line 543-546 to say “Briefly, genes essential for growth in rich media were identified by comparing normalized UIS per gene excluding insertions in the final 10% of the gene. Insertion indexes for all genes were plotted on a histogram that generates a bimodal distribution. Two normal curves are drawn from this histogram, and the intersect between the two normal curves is taken to be the cut-off to determine essentiality.”

**2. The authors identified 94 essential genes important for the in vivo adaptability of M1404 through mouse infection experiments. I am interested in understanding the rationale for selecting alsT_1, crp, and cyaA for the construction of individual mutant strains for further investigation. Since many other candidate genes were also identified, were targeted mutants of these genes constructed and analyzed as well? Did mutants of other genes exhibit similar infection phenotypes in mice, or did them fail to display significant phenotypic changes following screening? The authors focused their subsequent research on the crp and cyaA genes after gene screening. However, the function of the crp gene in Pasteurella multocida has already been reported in a previous studies (Identification of the crp gene in avian Pasteurella multocida and evaluation of the effects of crp deletion on its phenotype, virulence and immunogenicity. DOI: 10.1186/s12866-016-0739-y), and it is reasonable that cyaA, as an upstream gene, would affect the crp pathway. Therefore, conducting knockout studies on other screened genes that have not yet been reported may have greater scientific value.**

For *P. multocida* mutagenesis, we utilise a group II intron system to inactivate target genes (10.1016/j.mimet.2009.10.018). For the intron to be targeted to a gene of interest it must be precisely modified so that the LtrA can insert intron RNA into the target gene. We utilise ClosTron to identify potential intron target sites (10.1016/j.mimet.2009.10.018), which uses an algorithm to find potential target sites based on a weighted positional matrix (generated using successful intron insertion sites in *E. coli*, see 10.1016/j.jmb.2003.12.009). In our experience. target sites identified by ClosTron with a score > 6 have an approximately 50% success rate for generating an insertional mutant in *P. multocida*. We selected four genes to inactivate;  *alsT_1* and *prmC* in bothVP161 and M1404, PmVP161_0878 and *tyrP_1* in VP161, and *crp* and *cyaA* in M1404. These genes were identified as important for fitness *in vivo* and we planned to do systemic infections with each individual mutant, but unfortunately only introns targeted to the genes *alsT_1*, *crp* and *cyaA* successfully inserted into the M1404 genome. We were able to obtain PmVP161_0878 intron mutants, but these mutants always had a secondary intron insertion elsewhere in the genome. The *alsT_1* gene had previously been identified as important for VP161 virulence using an *in vivo* population of pooled random mutants (signature tagged mutagenesis) but had not been shown to impact virulence in an individual infection (10.1128/IAI.71.9.5440-5446.2003). *prmC*, PmVP161_0878 and *tyrP_1* had not been previously identified as associated with *P. multocida* virulence and were therefore of high interest to investigate in this study. The reviewer is correct that there has been some investigation of the effect of *crp* inactivation in *P. multocida* strain 0818 (10.1186/s12866-016-0739-y). That study reported on the effect of CRP loss on complement-mediated killing, antibody response and vaccine efficacy. The study also reported that the 0818 *crp* mutant had reduced expression of some of the capsule genes but no capsule quantification was reported. Our study has important points of difference as we examine two different *P. multocida* strains and show that CRP levels affect capsule production differently depending on the strain type. We agree that there were several genes identified in this study that warrant further investigation. However, given the CRP-family proteins are involved in regulation of virulence factors in other Gram-negative pathogens (10.1128/jb.00363-10, 10.1371/journal.ppat.1012529), we felt it was appropriate to further investigate and report on the role of CRP in *P. multocida*.

**3. Line 267: The authors state that inactivation of cpdA only slightly reduces the adaptability of VP161, yet targeted deletion of cpdA in VP161 could not be achieved, which appears somewhat contradictory. According to the Ecocyc database, cpdA is considered a non-essential gene in Escherichia coli, although the situation may differ in Pasteurella.**

We agree with the reviewer that it is unlikely that *cpdA* is essential. Indeed, our TraDIS data strongly support the prediction that *cpdA* is not essential, as we identified 31 unique insertion sites in *cpdA* following growth in rich media. Our extensive use of ClosTron mutagenesis (> 15 years) has revealed that it sometimes fails in *P. multocida* because it is dependent on the presence of the appropriate nucleotide sequence in the gene for the Group II intron. We predict our inability to obtain a *cpdA* mutant by this method is an issue with the intron targeting, rather than *cpdA* being essential or having a significant fitness cost when disrupted in VP161. Homologous recombination-based methods have rarely been successful in these *P. multocida* strains, so currently we have no other reliable mutagenesis methods with which to attempt mutagenesis of *cpdA*.

**4. Lines 461–472: The authors constructed the corresponding mutants using a method similar to targetron and provided detailed tables of strains and primers. However, the manuscript lacks detailed descriptions of the procedures for vector construction. If possible, could the authors provide comprehensive methods for vector and mutant construction in the supplementary materials**

We apologise for the omission and have added more detailed methods as follows:

- Added “(see S1 Text for more detail on pAL953 modification)” in the materials and methods on lines 476-477
- Added a section in S1 text describing the process of modifying our base group II intron to a gene of interest on lines 38-53
- Added in the restriction sites used to clone the retargeted region into pAL953 in S7 Table for the IBS and EBS1d primers
- Added BAP8243 to S7 Table

**5. I am curious about how the capsule was quantified in this study. In line 542, please specify the measurement method used, including the equipment (e.g., enzyme-linked immunosorbent assay (ELISA) reader, spectrophotometer), as well as the approach for quantifying hyaluronic acid. Were commercial kits, ELISA, or mass spectrometry used? As far as I know, polysaccharides are generally analyzed by SDS-PAGE silver staining or mass spectrometry. Please provide detailed experimental methods for capsule quantification as used in this study, as I could not find the relevant methodology in reference 7.**

Capsule was quantified using a colorimetric assay that has been used in numerous past studies to quantitatively measure *P. multocida* capsule (10.1128/IAI.69.4.2487-2492.2001, 10.1371/journal.ppat.1000750, 10.1128/spectrum.00195-22, 10.1016/j.micpath.2021.105145, 10.1128/spectrum.03805-23, 10.1186/s13567-021-01009-6, 10.1128/iai.00122-16). In our slightly modified assay (described in reference 7 of manuscript), surface capsule is extracted from mildly heated cells, then partially purified by ethanol precipitation before adding to a Stains-All solution. The absorbance at 640 nm of each sample is then measured using a Tecan. Absorbance of unknown VP161 samples were compared against values obtained for hyaluronic acid standards (VP161 capsule is composed of hyaluronic acid), which gives a linear regression of 0.99. As strain M1404 capsule is not composed of hyaluronic acid, we report only an absorbance value (normalised to culture OD). For clarity we have modified the materials and methods section on lines 572-579 to briefly explain the assay.

**Part III – Minor Issues: Editorial and Data Presentation Modifications**

**Please use this section for editorial suggestions as well as relatively minor modifications of existing data that would enhance clarity.**

**Reviewer #1: It would be helpful if some of the key common genes identified by TraDIS in both strains are labelled in Fig 2A. I accept this will be limited, but it would be helpful to readers.**

We agree that additional information in Figure 2 would be useful. However, we found that addition of genes either in Fig 2 A or Fig 2 C overcrowded the figure and impeded legibility; Fig 2 A has the fold-changes for the three TraDIS libraries in a single panel so there are three dots representing each gene, and there were too many genes identified in Fig 2 C to keep the figure manageable. As such, we opted to give full details in the supplementary materials to reduce figure complexity and size. We have given a list of genes identified as important for *in vivo* fitness in S4 Table with full data given in S4 data, and a list of genes that increased fitness when disrupted in S5 Table with full data given in S5 data. Furthermore, all genes that had either a fitness increase or decrease *in vivo* when disrupted are given in Figure 3, which is a good graphical representation of genes identified by the TraDIS analysis.

**The animal ethics should be a stand-alone section.**

The line regarding animal ethics that was given under the heading mouse systemic infections has been moved to a stand-alone section in the materials and methods on lines 503-506

**Reviewer #2: Minor Concerns:**

**1. The authors state the P. multocida strains cause infection across many different types of animals (from mammals to birds). Can the authors add a brief sentence or two about how the infection manifests in the wild (symptomatically) vs. what is observed in the mouse model? This will help to contextualize the model for those not in the field since the affected species vary so widely.**

We agree that this is important information. We have added “To cause disease, *P. multocida* often colonises the upper respiratory tract before spreading to the lower respiratory tract, and can rapidly enter the blood stream via tissue damage or the lymphatic system [14-16]. Entry directly into the bloodstream via skin has also been observed [4].” on lines 57-60 in the introduction

**2. To follow up Figure 1B, it would be helpful if the authors included a Venn diagram/Donut Chart/Heat Map or some other type of display that showed gene types which were distinct or conserved across the VP161 and M1404 strains. It is listed in the text (Lines 123-131) but may help the readers to see these displayed next to one another with relative differences in the number of genes belonging to each category.**

We appreciate that additional figures would aid in readability for this dataset but in the interest of brevity we have opted to list the most salient points of the rich media TraDIS data and give the rest of the data as S2 Data, which has homologs for all genes, predicted function and eggNOG ID matches if present.

**3. While the Venn diagrams in Figure 1B, 2B-C are nice display items because they demonstrate proportionality of overlap, they are quite difficult to read. Could the authors modify colors so that distinct and overlapping sections are more obvious? Perhaps the authors could also display the Venn diagram data as a table?**

We have modified the colours of the Venn diagrams for legibility. We feel that given the data is shown entirely in Figure 3, that for brevity we should not add a table.

**Reviewer #3: 1. Line 172, The authors described the identification of dozens of genes associated with bacterial in vivo adaptability. Ultimately, the deletion of seven genes was found to enhance the strain’s in vivo adaptability. Does this mean that the presence of these genes is beneficial for adaptability, or rather, that their deletion improves adaptability? Please confirm whether this interpretation of the conclusion is correct.**

We are not completely clear on the interpretation of this question relating to in vivo “adaptability”. TraDIS measures survival under particular conditions, which indicates essentiality or relative fitness in a particular niche. Transposon mutants in the genes listed in S5 Table were recovered at a significantly higher number compared to the input mutant library, indicating that insertional inactivation of these genes provides a fitness advantage compared to the rest of the population. We are not convinced that this would necessarily imply increased adaptability, as we have not directly measured adaptation to the host environment via transcriptional or genetic changes. We apologise if we have misinterpreted this question.

**2. In lines 175–199, the authors describe that multiple lipopolysaccharide or capsule synthesis-related genes are important for the in vivo adaptability of strains, mentioning many genes involved in polysaccharide synthesis. My question is whether all the genes mentioned have been reported in previous studies, or if they are novel discoveries made by this research group. If existing reports are involved, appropriate citations should be provided. For example, line 208 includes a relevant citation [28].**

In the results we have listed in text several of the genes identified as important for virulence and their potential functions. For the most part, we decided to reference previous work associated with these genes in the discussion, rather than throughout the results. We could add references to the results but feel for the sake of brevity that having these only in the discussion is most appropriate; however, we are happy to leave this to the discretion of the editor. Regarding polysaccharide synthesis, many studies have identified the importance of specific genes involved in capsule or LPS synthesis. However, we believe our study comprehensively shows all of the genes involved in these pathways are required for survival in mice. Additionally, the importance of the first heptose addition by *hptA* has been shown to be important for *P. multocida* virulence, but the synthesis system required for production of the modified heptose added to the core-polysaccharide was additionally shown here to be required for survival *in vivo*.

**Other modifications**

- Added Peracute at the start of line 60 in the intro
- Change most to almost all on line 177

**Journal Requirements:**

**1) Please upload all main figures as separate Figure files in .tif or .eps format. For more information about how to convert and format your figure files please see our guidelines:** 
[**https://journals.plos.org/plospathogens/s/figures**](https://journals.plos.org/plospathogens/s/figures)

Done

**2) Some material included in your submission may be copyrighted. According to PLOSu2019s copyright policy, authors who use figures or other material (e.g., graphics, clipart, maps) from another author or copyright holder must demonstrate or obtain permission to publish this material under the Creative Commons Attribution 4.0 International (CC BY 4.0) License used by PLOS journals. Please closely review the details of PLOSu2019s copyright requirements here: PLOS Licenses and Copyright. If you need to request permissions from a copyright holder, you may use PLOS's Copyright Content Permission form.
Please respond directly to this email and provide any known details concerning your material's license terms and permissions required for reuse, even if you have not yet obtained copyright permissions or are unsure of your material's copyright compatibility. Once you have responded and addressed all other outstanding technical requirements, you may resubmit your manuscript within Editorial Manager. 
Potential Copyright Issues:**

**- Figure S1. Please confirm whether you drew the images / clip-art within the figure panels by hand. If you did not draw the images, please provide (a) a link to the source of the images or icons and their license / terms of use; or (b) written permission from the copyright holder to publish the images or icons under our CC BY 4.0 license. Alternatively, you may replace the images with open source alternatives. See these open source resources you may use to replace images / clip-art:
-** [**https://commons.wikimedia.org**](https://commons.wikimedia.org) **-** [**https://openclipart.org/**](https://openclipart.org/)**.**

Everything in S1 Figure was drawn by hand except for the mouse intraperitoneal and organ diagram. These diagrams were obtained from BioRender, of which we have a paid subscription which allows us to use material for publications. BioRender is acknowledged in the figure legend.

**3) Please ensure that the funders and grant numbers match between the Financial Disclosure field and the Funding Information tab in your submission form. Note that the funders must be provided in the same order in both places as well.
- State the initials, alongside each funding source, of each author to receive each grant. For example: "This work was supported by the National Institutes of Health (####### to AM; ###### to CJ) and the National Science Foundation (###### to AM)."
- State what role the funders took in the study. If the funders had no role in your study, please state: "The funders had no role in study design, data collection and analysis, decision to publish, or preparation of the manuscript.".
If you did not receive any funding for this study, please simply state: u201cThe authors received no specific funding for this work.u201d**

Done
