# Supplementary material for: Comparative analysis of shared and unique mechanisms important for diverse strains of Pasteurella multocida to cause systemic infection in mice
Source: PLoS Pathog. 2025 Dec 22;21(12):e1013398. doi: 10.1371/journal.ppat.1013398 (PMC12721544; doi:10.1371/journal.ppat.1013398)
Supplement: Attachment — Submitted filename: Response to reviewers_Dec04_2025.docx [file ppat.1013398.s027.docx]

**Additional Editor Comments:**

**Please incorporate in the main text your answers to the reviewers when feasible (see comment from Reviewer 2)**

Reviewer 2 had previously made three to four suggestions for additional information to be given in the main text. In the previous revision we had made some additions into the main text; however, we have now added some more and for two of the suggestions given more detailed reasoning on why we believe further additions in the main text are not appropriate.

Note: all line numbers given below refer to the track changes version

**Reviewer 2 major comment 2 (in original review)**

We had already added into the discussion that *gmhB* and *crp* have been found in other *in vivo* TraDIS studies (lines 425-427 and 383-385) and included two of the studies suggested by the reviewer (10.1128/iai.00224-22 and 10.1371/journal.ppat.1012495). We did not find many additional individual genes when looking at other *in vivo* TraDIS/Tn-Seq studies, but did find a few biological systems that overlap, such as methionine uptake and purine biosynthesis in *Yersinia pestis* (10.1128/mbio.01385-14), and part of the shikimate pathway in *Salmonella enterica* subsp. Typhimurium (10.1371/journal.pgen.1003456). To take account of this, we have now added

“Several biological processes were identified as important for *P. multocida* survival that have also been identified by TraDIS as important for pathogenesis or *in vivo* survival in other bacterial species, including methionine uptake and purine biosynthesis in *Yersinia pestis* [49], and the shikimate pathway in *Salmonella enterica* subsp. Typhimurium [50].” on lines 327-331 in the discussion.

**Reviewer 2 minor issue** 1 **(in original review)**

We had added in additional information in the previous revision, discussing the disease progression on lines 57-60. However, we see that we may not have contextualised the natural disease against the mouse model in sufficient detail, so we have now modified the text to say “During a natural systemic infection *P. multocida* colonises several different tissues [4, 14, 16]. *P. multocida* disease often progresses rapidly and in peracute cases death can occur within 48 h of infection [17, 18]. Mouse models of *P. multocida* systemic infection are initiated by intraperitoneal injection [19, 20]. In such mouse models, *P. multocida* rapidly enters the bloodstream and colonises several tissues, mimicking natural systemic infections [19, 20].” (lines 60-65). We have also corrected the spelling of rapidly on line 58.

**Reviewer #2 Minor concern 2 (in original review)**

**“To follow up figure 1B, it would be helpful if the authors included a Venn diagram/Donout chart/Heat Map or some other type of display that showed gene types which were distinct or conserved across the VP161 and M1404 strains.”**

This would be a very large dataset to include in the main text. This Venn diagram shown in Figure 1B has 461 genes of overlap (447 of which are in the core genome) as well as 75 genes with homologs that are uniquely essential to VP161 and 65 genes with homologs that are uniquely essential to M1404. All of the genes and their predicted functions are accessible and searchable in the full supplementary dataset S2 data, which we believe is the most efficient and appropriate way to present this data. Therefore, we have not added anything further here into the main text.

**Reviewer #2 Minor concern #3**

**“While the Venn diagrams in Figure 1B, 2B-C are nice display items because they demonstrate proportionality of overlap, they are quite difficult to read. Could the authors modify colours so that distinct and overlapping sections are more obvious? Perhaps the authors could also display the Venn diagram data as a table?”**

For the first question we did already change the colours in the previous revision to make the Venn diagrams easier to read.

For the second question, we were unsure of what was being asked. We think the reviewer was asking for a table with just gene numbers listed for each of the different parts of the Venn diagram. We think this would not be an appropriate use of space as it duplicates what is already shown and what should now be easier to read due to the improved colors. Importantly, the genes that make up the Fig. 2C data, are all shown with gene names and their relevant heatmaps (including replicates) in the main text as Fig 3. Additionally, the data is presented as S4 Table and also S3 data in searchable format. We think reviewer 2 may have missed that Fig 3 contains a visual representation of each of the genes shown in Fig. 2C, with gene names shown, so to make this clearer for the reader, we now explicitly call Fig 3 when we call Fig 2C (lines 177 and 179). Additionally, we realised that when we dicuss the genes that showed increased fitness when mutated, we previously only called S5 Table; however, these genes are also shown in Fig 3. So, we have now also called Fig 3 at this point in the main text (Line 181)
